# Health system lessons from community practice: a qualitative study rethinking the role of social prescribing for refugee populations

**DOI:** 10.3389/fpubh.2025.1739953

**Published:** 2026-01-26

**Authors:** Victoria Touzel, Annabell Duda, Luisa Bartz, Doreen Reifegerste

**Affiliations:** AG4 Prevention and Health Promotion, Faculty of Public Health, Bielefeld University, Bielefeld, Germany

**Keywords:** community-led support, dark logic, health equity, refugee health, social prescribing, voluntary sector

## Abstract

**Background:**

Social prescribing has become central to UK health policy, positioned as a mechanism for addressing social determinants of health through non-medical interventions. However, little is known about its relevance for refugee populations, whose experiences are shaped by poverty, legal precarity, and systemic exclusion. Limited evidence exists on which delivery models are used in health and refugee-serving initiatives. Consequently, this study explores how different delivery models of social prescribing and community-led refugee support projects intersect and how this could inform health systems.

**Methods:**

Twenty-three UK practitioners participated in semi-structured online interviews, including social prescribers, community project leads, health professionals, and volunteers. Participants represented diverse projects, including arts, sports, language learning, peer mentoring, health advocacy, and General Practitioner (GP)-based social prescribing. Data were analysed iteratively to generate conceptual insights into delivery models, resources, challenges, and practitioner perspectives on social prescribing by using reflexive thematic analysis informed by realist principles.

**Results:**

Practitioner accounts revealed diverse delivery models across community-led and NHS-commissioned settings, converging around trauma-informed, person-centred approaches. Community projects frequently evolved into multi-service community centres combining social, legal, and health-related activities, sustained through precarious funding and reliance on volunteers. Social prescribers described exceeding their short-term signposting remit to meet complex needs. Key challenges included systemic underfunding, workforce burnout, language and cultural barriers, immigration status-based exclusions, and hostile policy environments. Resources identified included dedicated staff, volunteers, and community partnerships. Practitioners identified relational trust, safety, and empowerment as key mechanisms of impact for refugees. While social prescribing delivery models were viewed as conceptually aligned with refugee-serving community organisations, practitioners identified several potential harms around capacity, accountability, and suitability of referral models.

**Conclusion:**

Based on practitioner perspectives, community-led refugee projects offer important lessons for social prescribing: the importance of trauma-informed practice, cultural safety, relational outcomes, and sustained accompaniment. Findings suggest that for social prescribing to be effective and safe for refugee populations, systemic adaptations may be needed, including: long-term funding, prescriber training in trauma and asylum contexts, improved service mapping, and evaluation frameworks valuing relational outcomes. These insights require validation through research with refugee populations to establish whether identified challenges and harms manifest as described.

## Graphical abstract

**Figure:**
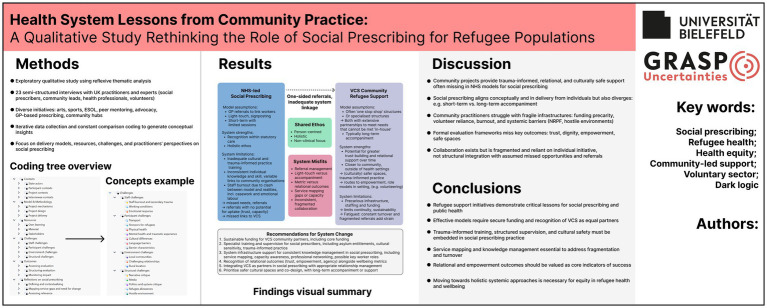

## Highlights

- First qualitative UK study exploring how social prescribing delivery models operate in refugee support contexts from practitioner perspectives.
- Identifies community-led projects as central yet under-recognised actors that practitioners describe as providing trauma-informed, culturally safe, and relational support.
- Highlights systemic challenges identified by practitioners: funding precarity, workforce strain, ‘hostile environment’ policies and exclusion by immigration status.
- Applies dark logic framework to reveal potential unintended harms and paradoxical effects in referral systems, particularly around continuity, accountability, and evaluation, based on practitioner accounts.
- Proposes practical recommendations for sustainable funding, prescriber training in trauma and asylum contexts, recognition of relational outcomes, and stronger integration with voluntary sector partners, requiring validation through research with refugee populations.

## Introduction

Social prescribing is an established approach in the UK, introduced in the 1990s and now increasingly central to health policy (1, 2). Typically, social prescribing takes place through a referral pathway, often one where healthcare professionals refer patients to social prescribers who then connect them to community-led activities and support services in a time-limited support service (typically 6–12 sessions) (3). These may include arts, exercise, or advice services, and social groups. Through these connections, social prescribing seeks to reduce loneliness, strengthen social support networks, and promote holistic wellbeing (4, 5).

The evidence base for social prescribing has grown substantially, with studies indicating improvements in wellbeing, social connectedness, and reductions in primary care attendance (1, 6–9). Social prescribing informs national policy initiatives such as the Fit for the Future: 10 Year Health Plan, where Neighbourhood Health Centres providing a ‘one stop shop’ service with multidisciplinary are viewed as central to improving health equity and reducing demand on overstretched clinical services (10, 11). It has been framed as part of wider public health systems seeking to improve population health equity (10, 12, 13), but this is also a framing which is being interrogated (14–17).

Currently, the literature largely reflects mainstream healthcare perspectives and general population use (16, 18). Relatively little attention has been paid to how social prescribing models function for marginalised or migrated groups (6, 19). More specifically, we still have yet to understand how they intersect with the lived realities of poverty, trauma, and legal precarity that characterise many refugees’ and asylum seekers’ experiences.

In this article, the term ‘refugee’ is used in a broad and inclusive sense, encompassing people who have sought protection outside their country of origin, including those formally recognised as refugees, individuals awaiting decisions on asylum applications, and others with precarious or temporary legal status (often described as asylum seekers or forced migrants) (20, 21). We use this terminology to reflect the diversity of legal, social, and lived realities within refugee populations, while recognising that experiences are shaped by intersecting factors such as poverty, trauma, displacement histories, and resettlement contexts. Consequently, refugee support is here defined as a wide range of community-led, voluntary and statutory initiatives that provide social, legal, cultural, or wellbeing assistance to people with refugee, asylum, or other precarious protection or migration statuses. We therefore use the term ‘community-led projects’ in this article to describe all kinds of initiatives and projects originating from and driven by community and voluntary sector organizations, in contrast to NHS-commissioned social prescribing services. Furthermore, we use the term “trauma-informed practice” to refer to approaches the prevalence and effects of trauma, prioritise physical and emotional safety, and allow people to engage in ways that support choice, agency, and trust.

To date, refugee health research has predominantly focused on clinical outcomes, health service access, and integration processes, with relatively little structural analysis of pathways to boost social integration and cultural safety (22–24). Although critically needed, these focuses give less consideration to community-led, informal, or social-capital-based approaches to wellbeing (25). Public health scholarship highlights the critical importance of social networks, cultural safety, and grassroots infrastructures in promoting health among marginalised groups (26–28). Yet, the accessibility, cultural appropriateness, and adaptability of social prescribing to refugee contexts has remained largely unexamined. This represents a pressing research gap at the intersection of public health, health equity, and refugee wellbeing.

## Background

Despite the prominence of social prescribing in UK health policy and practice, several significant gaps emerge when considering refugee populations. The following sections outline four interconnected gaps that inform this study’s research questions.

### Gap 1: limited understanding of delivery models and methodologies

Firstly, there is minimal peer-reviewed research documenting the delivery models used in diverse social projects for refugee populations and how this is relevant to social prescribing. The term ‘delivery models’ here describe the organizational approaches and structures behind services, defining what is designed, delivered and evaluated and how this is organised. This working definition is based on a suggested framework from Piña et al. (2015) comprising sections on capacity, organizational structure, finances, patients, care processes and infrastructure, and culture (29). The only UK systematic review of social prescribing for migrants broadly found very limited evidence and no evaluative studies detailing service adaptation processes (19). A qualitative investigation involving practitioners and migrants underscores the need for culturally tailored delivery and co-production, but highlights that such adaptations remain largely undocumented in formal evaluations (6). Neither study provides refugee-specific evidence. Other relevant social prescribing analyses concentrate on the roles of social prescribers within NHS primary care and systems-level integration (1, 7, 8, 18, 30). While voluntary and community sector organisations are acknowledged as referral partners, their on-the-ground experiences and expertise outside statutory systems are largely absent from published analyses.

### Gap 2: Challenges and resources in practice

Secondly, very little is known about whether or how refugees and asylum seekers access social prescribing services in the UK, or what challenges and resources refugee-serving practitioners encounter in practice or service access. Evaluations seldom disaggregate data by migration status, leaving blind spots around equity of access and outcomes (16, 19, 25). Furthermore, no clear or specific evidence base exists on the cultural, linguistic, or material appropriateness of social prescribing interventions for refugees (19, 27). Questions also remain around whether needs identified through social prescribing are addressed when they fall outside the remit of statutory services, including whether referrals function ‘successfully’ for both statutory and third sector stakeholders, and where final accountability lies to meet complex needs (31, 32). The challenges and resources refugee-serving community practitioners encounter while delivering support remain under-documented. This means there is no understanding of how these actors interoperate with NHS-led social prescribing schemes or bridge systemic gaps in refugee support (for instance at status change entitlements provision) by providing community capacities and resources (33). This creates a ‘black box’ in our system understanding of how social prescribing referrals may or may not function for refugee individuals, as we do not understand the capacity of the sector to meet and respond to referrals in from the health system.

### Gap 3: practitioner perspectives on social prescribing relevance

Thirdly, the role of the voluntary and community sector is central yet under-documented. These operational and system pressures highlight that delivery challenges cannot be separated from the practitioners navigating this environment. While charities and grassroots organisations often deliver support to refugee populations and collaborate with social prescribers, little peer-reviewed work examines practitioners’ perspectives on how social prescribing systems align with their refugee support work (28, 34). Informal and grassroots organisations, which are often the first point of contact for refugees, remain largely absent from the literature (19). Practitioners working in refugee support projects hold vital forms of practice-based knowledge that are rarely reflected in formal research or policy debates (35). Their expertise includes building trust with populations who may be marginalised or fearful of institutions, engaging communities in culturally and linguistically relevant ways, and delivering services in resource-constrained environments (36, 37). They also play a crucial role in promoting enabling factors that support participation, while addressing barriers linked to trauma, poverty, and precarious legal status (23, 33). Such practice wisdom is indispensable for understanding how interventions are experienced on the ground and whether social prescribing is perceived as relevant and feasible for refugee populations to access.

### Gap 4: best practices and lessons for future services

Finally, there is currently no synthesis of best practices or lessons learned that could inform the design of future social prescribing services for refugee populations. Without systematic documentation of what works, what does not, and why, there is a risk that interventions may be designed or delivered without adequate recognition of service and community capacity limitations, funding constraints, structural barriers, or the lived realities of displacement and traumatic experiences. As a result, even well-intentioned initiatives may inadvertently exclude or retraumatise the very populations they are meant to support. To assess this potential for harm, the key analytical approach for this work draws on Bonell and colleagues’ ‘dark logic’ framework (21). Here the authors theorised that public health interventions can produce unintended harmful consequences through complex interactions between agency, structure, and context. Their ‘dark logic’ framework emphasizes the importance of theorizing not only intended beneficial mechanisms but also potential pathways to harm – a relevant consideration when delivering services to populations experiencing trauma, systemic exclusion, and hostile policy environments.

### Research questions

This study responds directly to these gaps in understanding by purposefully sampling practitioners whose perspectives are under-represented, asking the following research questions (referred to later as RQ1, RQ2, RQ3, and RQ4):

(1) What delivery models and methodologies are used by practitioners in social projects for refugee populations?
(2) What are the primary challenges and available resources that practitioners identify in their work?
(3) How do practitioners perceive the relevance of social prescribing for refugee support?
(4) What lessons and best practices can inform future social prescribing services for refugee populations?

## Methodology

### Study design

This study adopted an exploratory qualitative design using reflexive thematic analysis following Braun and Clarke’s approach (38), with further analysis drawing on Bonell and colleagues’ dark logic framework (39), and adhered to the Consolidated criteria for Reporting Qualitative research (COREQ) guidelines (40). This methodology was chosen to provide a flexible and rigorous framework for identifying and interpreting patterns of meaning across diverse practitioner accounts, while recognising the active role of the researcher in knowledge generation. It is particularly suited to applied public health research where limited evidence exists and the aims is to develop conceptually rich insights grounded in participants’ lived experiences and practice-based knowledge (38). Rather than testing predefined hypotheses, the analysis proceeded inductively to explore how practitioners conceptualised delivery models, resources, challenges and social prescribing within refugee-support contexts. The approach was also informed by realist review principles, both in recognising practice knowledge as a critical form of evidence and in coding categorisation strategy (41, 42).

### Sampling strategy

The study focused on expert interviews with practitioners engaged in refugee-serving projects in the UK. A purposive sampling approach was adopted to ensure inclusion of participants with direct, relevant expertise in refugee social projects. Inclusion criteria required participants to:

- Be professionals or volunteers directly engaged in refugee-related projects within the UK.
- Have experience in project design, delivery, and/or evaluation.
- Be involved in projects addressing information needs, skill-building, social support, or social integration.
- Be willing and able to provide informed consent.

Exclusion criteria included lack of direct refugee project experience or inability to provide informed consent. Recruitment aimed for diversity across project types and settings (e.g., GP-based social prescribing, community projects, arts, sport, gardening, language learning, mental health, school-based and university projects in differing localities, both rural and urban).

### Recruitment

Participants were identified and recruited through:

- Respondents from the accompanying literature review who expressed interest in participating in expert interviews (*n* = 13) (Touzel et al., under review).
- Structured outreach to NGOs, community organisations, and statutory agencies aligned with the project representation criteria (*n* = 2).
- Snowballing from recommendations made by participants (*n* = 3).
- Reaching out to personal and professional networks (*n* = 5).

Potential participants were first contacted by email or phone, provided with an information sheet and consent form, and screened for eligibility through correspondence (see Data sheets 1 and 2).

### Data collection

Twenty-three practitioners took part in the study. Written informed consent (see information for participants below) was obtained prior to interviews, along with brief socio-demographic details (e.g., gender, role, years of experience). Twenty-two expert interviews were conducted online via Zoom at times convenient for participants with an anticipated length of 1 h. One interview was conducted in written form at the participant’s request, which was then directly anonymised without further alteration.

A semi-structured interview guide was used (see Data sheet 3), with sections addressing: project delivery models and organizational structures; evaluation practices and outcome measurements; stakeholder relationships and referral pathways into services or projects; perspectives on alignment with social prescribing models; challenges and resources; and lessons learned and best practice recommendations.

All Zoom interviews were audio-recorded using the designated Zoom function, with verbal explanation of data processing and privacy sought before the interview started to determine participants’ active consent. Interviews were transcribed verbatim using supporting software f4x. Transcripts were downloaded, read for accuracy and anonymised immediately by the lead researcher, who also conducted all interviews. No field notes were kept. Data collection continued until theoretical saturation was reached. Saturation was determined through a systematic process: after conducting 18 interviews, the research team independently reviewed the emerging codebook and discussed whether new conceptual categories were appearing. When the final 5 interviews yielded no new codes and only provided examples of existing themes, we concluded that saturation had been reached. This agreement was reached through research team discussion, with all researchers involved in the coding process and validation agreeing that the existing thematic framework adequately captured the range of perspectives and experiences from the planned purposive sampling. It is possible that further perspectives were not captured or addressed, which is addressed below under limitations.

### Data analysis

Data analysis involved a systematic process of familiarisation with the data, generating initial codes, constructing and reviewing themes, and refining thematic maps through an iterative and reflexive process (38). The analysis proceeded concurrently with data collection, allowing insights from earlier interviews to shape subsequent data gathering in an iterative manner. In addition to inductive thematic analysis, Bonell and colleagues’ dark logic framework (39) was applied deductively retrospectively to the finalized coding framework during later stages of analysis to systematically identify and theorize potential harmful pathways and unintended consequences emerging from practitioners’ accounts. Coding was conducted inductively to capture semantic and latent meanings in participants’ accounts, considering both shared experiences and contextual diversity across roles and settings. Reflective thematic analysis also recognises the active role of the researcher in knowledge construction, emphasising subjectivity and reflexivity in analysis. The analytic process therefore followed these six recursive phases:

1) Familiarisation—repeated reading of transcripts and initial noting of ideas.
2) Coding—systematic coding across the full dataset to identify meaningful themes.
3) Generating initial themes—grouping codes into potential themes and subthemes.
4) Reviewing themes—refining themes in relation to coded extracts and the dataset as a whole.
5) Defining and naming themes—identifying the essence of each theme and the relationships between them.
6) Producing the report—selecting extracts to illustrate analytic claims.

Coding was conducted using MaxQDA 2025 software. To enhance rigour, initial coding by the first author was discussed with the second and third co-authors, who independently assessed a subset of transcripts and exchanged as a group to discuss coding schemes, discrepancies and other valuable inductive coding categories until consensus was reached.

The final coding tree (see Data sheet 4) included 6 core themes (Contexts, Model and Methodology, Resources, Challenges, Outcomes, Reflections on Social Prescribing), with a further 20 sub-categories and 72 concepts.

### Researcher reflexivity

The research team brought diverse professional backgrounds to this study, including public health, experience in health and community project management in the UK voluntary sector, health communications and health inequity research. Our collective experience included both critical perspectives on health system inequities and recognition of social prescribing’s potential benefits. We acknowledge that the research focus on unintended harms could lead to overemphasis on identifying and discussing negative impacts. The original questionnaire is provided with this article, which shows the variety of questions posed to also identify positive experiences (e.g., resources perceived by interviewees, unexpected allies or sources of support, perception of social prescribing as a useful approach, aspects of social prescribing which may be particularly helpful for refugees). Codes for positive descriptions of social prescribing based on the above question areas were integrated into the coding framework from the outset. Following reflective exchange, further new thematic codes that broadly reflected positive resources or assets were also added to the coding framework, such as for environment (which was often used by interviewees to describe natural assets and the positive impact of time spent outdoors, directly relevant to social prescribing and nature on prescription). We also ensured that each researcher independently coded a subset of transcripts before collaborative discussion, allowing us to identify and interrogate divergent interpretations in ongoing exchange. The focus on unintended harms using a dark logic framework was then applied retrospectively to better contextualise and interpret results, as a methodological decision applied after reflexive exchange between the researchers and a wider research team also working on related topics (see Acknowledgements). We also reflected on overly deterministic coding related to unintended harms. An example of this would be attribution complexity, where we initially focused on interactions between social prescribing models and community services for described harms. Reflexive discussion led us to recognise that participants often described harms emerging from the interactions between social prescribing and community structures with pre-existing systemic issues (such as austerity-driven service cuts or longstanding NHS-community power imbalances). We revised our analytical approach to better distinguish between harms attributable to social prescribing design versus those where social prescribing amplified or made visible existing structural problems (see Discussion). However, further reflective work could have supported the thematic development and methodological decisions, which is reflected under limitations below.

### Ethical considerations

Ethical approval was obtained from the Ethics Commission from Bielefeld University (reference 2025–059). Data is stored securely in line with GDPR requirements.

### Positionality statement

The research team acknowledges the importance of reflexivity in qualitative research, as our backgrounds, professional experiences, and personal identities inevitably shape how we approach research, interact with participants, and interpret findings.

The team comprises researchers with backgrounds in public health, communication science and social policy, with experience of working with economically disadvantaged and migrated groups. The first author has a personal migration background and all researchers speak multiple languages. The first author also has experience of working within charity contexts with refugee groups in the UK, similar to many of the participants in the research. None of the researchers have experienced forced migration or identify as male, which are limitations we have sought to mitigate through foregrounding practitioners’ voices and critical reflection.

We recognise the potential for power imbalances between academic researchers and community practitioners, and we took steps to minimise these, such as ensuring limited demographic and personal information would be requested, leaving it open to participants to share only to the degree that they were comfortable with. We specifically conducted member checking for transcripts in individual cases and in discussing the emerging findings with interested interviewees. Additionally, feedback was sought from practitioners at the end of interviews, with emerging themes being shared back with this group for discussion and influencing future interview questioning.

## Results

### Participant demographics

All Zoom interviews varied between 43 and 100 min in length, with a mean length of 66 min. Table 1 presents the demographic characteristics of the 23 participants in the expert interviews. Variables captured included gender, professional role, area of expertise, years of experience, and project focus. These illustrate the diversity of expertise represented in the sample and help contextualise the subsequent qualitative findings:

**Table 1:** Demographic characteristics of participants.

Participant	Project focus	Gender	Role	Years	Areas of expertise
P1	Social prescribing	Female	Social prescriber	1	Social prescriber and therapist running creative art projects with marginalised groups
P2	Social prescribing	Female	Youth service manager	2	Social prescribing manager with background in youth work
P3	Diaspora charity	Male	Youth and sport manager	25	Sports and activities organisation and management within community-based support service hub, and current charity chair
P4	Peer mentoring	Female	Senior researcher	10	Researcher focused on person-centred care, background as speech and language therapist
P5	Diaspora charity	Male	Charity director	2	Acting within community-based support service hub including legal support, language learning, women’s groups, events, sports, employment, signposting
P6	Performing and arts	Male	Producer	13	Background in arts and migration, and events organisation
P7	Social prescribing	Female	Social prescriber	8	Social prescribing with background in legal advice as an immigration advisor, runs additional diaspora support and signposting group
P8	Diaspora charity	Female	Integration manager	3	ESOL and language learning within community-based support service hub, including legal advice, women’s groups, employment, activities, advocacy, and signposting
P9	Performing and arts	Female	Drama programme founder and producer/ director	9	Leading community arts and performance for women’s groups hosted by wider community support service hub, with a background in ESOL
P10	Performing and arts	Female	Writing project coordinator	22	Leading community arts and performance group hosted by wider community support service and advocacy hub
P11	Transport and activity	Male	Founder and CEO	8	Leading community project, with background as public health inequities expert
P12	Social work and signposting	Female	Founder and research coordinator	24	Now volunteering with community charity that pairs social care students with refugees for case work and signposting, background as social worker
P13	Language cafés and signposting	Female	Charity chair	6	Volunteer for ESOL and signposting needs, sports activity organisation, background as a teacher
P14	Health service improvement	Female	GP and lead for local health stream initiative	11	Expert on primary care and health system strengthening, social prescribing from GP perspective
P15	Regional charity supporting new arrivals	Female	Wellbeing manager	8	Coordinating activities and sport for wellbeing as part of a community-based hub offering legal, housing and social support, mental health support, signposting
P16	Local authority education	Female	Quality Assurance Teacher for post-16 education	3	Support for children in local authority care including unaccompanied refugee minors to ensure equitable access to education
P17	Social prescribing	Female	Social prescriber	3	Social prescribing in rural settings, background in mental health cares
P18	Regional charity for migrated populations	Female	CEO	6	Migrant and refugee support in a community-based hub leading public health initiatives, language learning, childcare, events and activities, translation and accompanying to appointments, signposting, background in childcare and education
P19	National programme for inclusion	Female	Schools coordinator	6	Supporting schools and other organisations to change policies, practice and delivery to foster inclusion, background as a teacher
P20	Regional public health delivery	Male	Regional manager for health inequities	37	Health inequities with focus on alcohol and substance abuse, mental health and suicide risk
P21	Healthcare provision and advocacy at international charity	Female	Advocacy manager and primary care lead	3	Public health expert on health inequities focusing on primary care system barriers and strengthening, health delivery in camp settings
P22	Social prescribing	Female	Social prescriber	2	Social prescriber for migrated populations including first arrivals still living in camp or hotel settings, background as a nurse
P23	Local charity and community space	Non-Binary	Volunteer	9	Local project with established community garden, offering particular group sessions and courses with a focus on green therapy

The sample was predominantly female (*n* = 16, 69.6%), with smaller representation of male (*n* = 6, 26.1%) and non-binary (*n* = 1, 4.3%) participants. Years of expertise in their current role ranged widely, with 8 participants (34.8%) in shorter-term contracts or roles (0–3 years), 11 (47.8%) in more established positions (4–13 years), and 4 (17.4%) in long-held roles, often due to long-term involvement as a founder, volunteer or staff member (14 + years). Professional roles spanned from frontline practitioners (e.g., social prescribers, project workers, volunteers) to senior managers, directors, researchers, and CEOs, reflecting a mix of operational and leadership perspectives. Many also contributed significant secondary experience from other professions (e.g., therapist, teacher). Areas of expertise clustered around social prescribing, community-led support hubs, arts and creative practices, public health, education, and advocacy, with the majority of participants combining professional expertise with direct engagement in migrant and refugee support (*n* = 15, 65.2%). Multiple participants described their own migration backgrounds within the contexts of the interview without this being directly requested (*n* = 7, 30.5%), of these with some specifying personal forced migration histories (*n* = 2, 8.7%).

Although not explicitly captured in the demographics survey for reasons of project identifiability, efforts were made through the systematised purposive sampling to ensure diversity in settings as well as project focus. Of the 23 participants, the largest proportion were from London (*n* = 8, 34.8%). Smaller clusters were based in Plymouth (*n* = 3, 13.0%), Glasgow (*n* = 2, 8.7%), Liverpool or the Wirral (*n* = 2, 8.7%), and West Yorkshire (*n* = 2, 8.7%). Single participants were recruited from Brighton, Aberdeenshire, Norwich, Greater Manchester, Essex, and Dublin (each *n* = 1, 4.3%). Due to the nature of this research and the voluntary and community sector landscape, multiple sub-clusters of organisations or individuals who worked with each other were identified, but this occurred as often by chance as by recommendation. Overlapping aspects for sub-clusters was seen in themes addressed but also specifically in stakeholders mentioned, where some interviewees referred directly to other projects’ work that were interviewed.

### RQ1: delivery models and methodologies

RQ1 examined the delivery models and methodologies used by practitioners in social projects for refugee populations, whereby practitioners described diverse delivery models across settings. Findings are reported under heading for project design, delivery, and evaluation, with reference to either community-led projects/community practitioners (which refers to all respondents who were not social prescribers), to social prescribing services/social prescribers, or to all interviewees.

### Project design

Most community-led projects reported being created in response to observed population needs. Some noted that they were not specifically responding to commissioning (e.g., where the NHS contracts services from providers). The projects varied significantly in design formality. Community-led projects often drew on trauma-informed, person-centred, and holistic wellbeing principles, defining these as prioritising safety, trustworthiness, and empowerment in service delivery, and prioritizing holistic individual needs. These principles were often reported as embedded through co-design or responsive processes ensuring cultural sensitivity and ownership.

NHS-commissioned social prescribing roles followed more formalised models and structures, though often delivered in hybrid form (managed by charities but embedded in health settings). Social prescribers described experiencing conflicts between their preferred approaches, defined as prioritizing trauma-informed, person-centred, holistic care and social prescribing delivery frameworks. Social prescribers described instances where what they felt individuals needed did not align with framework restrictions, particularly in terms of how long support services should be offered. All social prescribers framed social prescribing delivery models as short-term signposting services, typically six–eight sessions. However, all reported operating outside these frameworks to meet individual complex needs: “They call it light-touch, but these cases do not just end in eight weeks” (P1, Social Prescriber). All social prescribers stressed that their individual methodology was relational and trauma-informed in practice, but that this approach was inadequately recognised in NHS models and structures.

A minority of projects reported surviving early years informally by engaging volunteers and evolving into structured organisations over time. Multiple practitioners described operating on short-term contracts or insecure grants, and a few described projects sometimes closing or surviving only through volunteer support.

### Project delivery

Recruitment pathways differed by setting. Community-led projects relied heavily on self-referrals, primarily based on word of mouth and trusted contacts, while statutory and university projects cooperated more closely with other local authority teams. Social prescribers reported that referrals came mainly from GPs and allied health staff, with limited community self-referral.

Community-led projects described working predominantly with asylum seekers and refugees, often in contingency accommodation or hotels, alongside specific national groups such as Afghans, Syrians, and Ukrainians, as well as unaccompanied minors and women’s groups. All social prescribers reported highly diverse caseloads, including recent arrivals, long-settled migrants, single mothers, young adults, and frequent GP attenders, while community-led projects tended to emphasise targeted work with asylum seekers and ethnic minority communities.

Primary activities delivered varied widely, including physical activities; language, education, or employment; creative and arts activities; horticulture or outdoor activities; mothers’ or women’s groups; advice and signposting. The vast majority of community-led projects described developed or developing “one stop shop” (P8, Integration Manager) structures in response to need, combining primary activities with supporting services, e.g., childcare, legal aid, community kitchens, interpretation. Only one community-led project had a focus on delivering one activity with referrals outwards for other needs.

The majority of community-led projects had small teams of 1–3 paid staff members and described being heavily dependent on volunteers for delivery. While volunteers were valued as a strength in terms of community ownership, some practitioners described concerns about sustainability and continuity in delivery. Multiple community-led projects described cycles of staff loss whenever funding ended or contracts expired, while social prescribers highlighted burnout and exit from posts, which is discussed further in results for RQ2.

Partnerships in delivery were near-universal, with most projects collaborating with schools, NGOs, housing providers, legal advisers, health services, or cultural groups. Some projects had already strategically developed into extensive hubs that combined food, legal, health, and social activities, while others relied on partnerships primarily to extend capacity and provide referrals into services they could not deliver themselves. Community practitioners described developing ‘one stop shop’ structures that were sometimes linked to funding constraints, while others emphasized participants’ needs for safe and trusted spaces and practical considerations such as childcare and access to multiple supports under one roof. Social prescribers more often functioned as brokers into multiple agencies, including statutory services, with inconsistent partnerships with the voluntary and community sector determined by capacity and interest.

### Project evaluation

Evaluation practices were uneven across settings. Community-led projects most often evaluated through case studies, feedback forms or surveys, and informal reflection. In several instances, evaluation outputs were explicitly linked to advocacy and empowerment, for example by providing participation certificates to support asylum claims or by feeding case studies into campaigning and narrative change.

Some community practitioners described concerns about formal scales. They reported that tools were imposed by funders, were limited or poorly fitting for the population, or missed what mattered. Only one community practitioner describing blended formal tools with co-production in a more positive way. Social prescribers similarly described formalised but often inadequate tools that failed to capture complex realities. They instead described the importance of qualitative data in the form of feedback, case studies and vignettes that focus on sense of safety, dignity, and relational outcomes as part of individual growth as the real markers of success.

Community practitioners emphasised that project participants most likely to benefit were those who were socially isolated, young people with some stability (e.g., leave to remain), and women seeking safe spaces. Barriers were described as greatest for single mothers without childcare, unaccompanied minors with depression, and those facing language, poverty, or disability-related obstacles. Social prescribers similarly observed that refugees and migrants broadly could benefit as “high contributors in transition” (P12, Founder and Research Coordinator), but stressed that those most in need (people fearful of authorities, lacking English, or housebound through ill mental or physical health) were often the least able to access services.

Across all interviews, individual journeys, involving slow rebuilding of trust and agency, were considered the most meaningful indicators of success. Multiple participants emphasized agency, empowerment, and participant voice as important outcomes, with projects describing empowerment through feeling ‘seen’ or ‘heard: “Drama gave them a safe way to say things they could never have said otherwise” (P9, Drama Programme Founder). Some noted that these outcomes were not typically captured in formal NHS metrics.

Mechanisms of individual change most often centred on the creation of safe and trusted environments and relational trust-building between participants and community practitioners or social prescribers. Several projects described pathways from participation into volunteering and leadership with transformative effects: “certificates aren’t just paper, they can shift how authorities view someone” (P4, Senior Researcher). Public visibility through arts, education, or cultural programmes also served as an important mechanism for representation, building confidence and belonging: “Art lets people show who they are, not just what’s on their Home Office file” (P2, Youth Service Manager).

Mechanisms of wider societal change were less commonly mentioned but included narrative change through public visibility, advocacy, and partnership-building to institutionalise refugee inclusion:

“We have a sort of special, sort of secret weapon that we could use, which is that we can get to audiences that that no amount of op eds or campaigning or social media about asylum or the iniquities of the asylum system could possibly achieve… And that way you kind of slide behind their defences.” (P10, Writing Project Coordinator).

### RQ2: primary challenges and resources

RQ2 examined the primary challenges and available resources that practitioners identified in their work. Participants described substantially more challenges than resources. One stated: “For the social prescribing, well that’s not going to work, because there is not capacity out there at all.” (P6, Producer).

## Challenges

### Workforce and organizational challenges

Staff across community organisations described working conditions marked by fragility, with small teams stretched thin, heavy reliance on volunteers, and limited contracts. Burnout and compassion fatigue were frequently mentioned, with several highlighting secondary trauma from repeatedly hearing distressing stories in the absence of adequate supervision: “We carry their pain back home” (P16, Quality Assurance Teacher). Many also pointed to training gaps, noting that while trauma- or asylum-specific training was badly needed, funding and time rarely allowed it.

Interpretation and translation placed further pressure on staff, with community practitioners describing often stepping in themselves or relying on patchwork solutions. Social prescribers similarly emphasised the psychological burden of carrying complex trauma stories while being unable to resolve systemic barriers, the inadequacy of telephone and inconsistent interpreters, and lack of support despite the depth of mental health and safeguarding issues they encountered: “We’re listening like therapists but without the supervision” (P1, Social Prescriber).

### Population and community-level barriers

Across all interviewees, mental health and trauma-related needs were the most frequently cited individual challenge, with community practitioners linking ongoing legal uncertainty to heightened anxiety, re-triggering of trauma around Home Office interviews, and difficulty sustaining participation: “You can see depression shutting them down in front of you” (P23, Volunteer).

Language barriers were also widely reported, shaping access to GPs, trust, and health literacy: “this is a desperate need… you cannot possibly come into a country and be part of the community if you cannot speak the language” (P13, Charity chair). Several interviewees noted that even the term “social prescribing” or standard scales do not translate effectively and should be rethought or interpreted: “we never used ‘social prescribing’ as a term” (P2, Youth Services Manager).

Cultural difference and mistrust of institutions were often addressed alongside commentary on the hostile climate: “It’s not just about putting a logo on something and saying ‘safe’. It’s about who they see there, who is in the room, and whether they feel this is really for them.” (P18, CEO).

### Systemic and policy barriers

Participants described system-level stressors including hotel or camp isolation, dispersal, fear of the Home Office, and distance to services. The most commonly cited structural constraint concerned money and entitlements: extremely low asylum support and allowances (43), No Recourse to Public Funds status (NRPF) (44), transport poverty combined with geographical isolation and cliff-edges at status change. Participants linked these to attendance, continuity and feasibility challenges: “they are hungry, they are cold—that’s what NRPF means” (P7, Social Prescriber); “People in hotels are just stuck, miles from anything” (P11 Founder and CEO).

The precariousness of the transition period after being awarded leave to remain was stressed by multiple participants:

“At the point of leave to remain, people face destitution and homelessness and loss of income and resources because they are not eligible for benefits or work until everything has been processed and sorted out, which can take many weeks.” (P12, Founder and Research Coordinator).

Additional environmental challenges included adversarial or inconsistent relationships with statutory services, a hostile policy climate in which racist or stigmatizing narratives circulated, and direct experiences of racism, Islamophobia, and systemic violence: “Young men get stopped by police for nothing” (P3, Youth and Sport Manager). Social prescribers further described clients denied access to healthcare or burdened with NHS debts, which in turn could undermine immigration applications.

Some participants described concerns about policies including the ‘20-year rule’ for visa eligibility (45), the lack of safe migration routes, forced dispersal, and a wider policy environment described as “inhumane” (P7, Social Prescriber).

## Resources

### Organizational learning and staff commitment

Interviewees described their own learning as a critical resource. Community practitioners reported learning by adapting to language needs, by building culturally sensitive spaces that respected intersectional and lived experiences, and by developing trauma-informed approaches that emphasised dignity, pacing, and belonging. Many reported shifting their own focus from narrow education or work goals toward broader measures of empowerment and inclusion, stressing that this shaped daily practice.

Social prescribers likewise underscored their own learning, describing how experience taught them to adapt when formal services excluded clients (e.g., NRPF, mothers with childcare needs), to prioritise holistic assessments over rigid models, and to set boundaries, while acknowledging systemic limits.

Staff themselves were consistently described as a core resource. Community organisations highlighted how small but dedicated teams often worked beyond contracted hours, sometimes even continuing unpaid when funding lapsed, while their lived experience and facilitation skills were seen as irreplaceable in sustaining trust. Social prescribers similarly noted that their own commitment and peer-support networks among colleagues were vital in coping with heavy caseloads and emotional strain.

### Volunteers and community partnerships

Community-led projects viewed volunteers as a critical resource, who often sustained programmes during funding gaps and sometimes progressed from service users into leadership or employment. A smaller number of projects were entirely volunteer-led in their early stages. Supportive relationships with schools, councils, and other charities and in-kind donations from local businesses, retailers, and individuals were consistently described as vital stop-gaps.

Social prescribers emphasised the importance of supportive relationships with community organisations, food banks, and advice centres. They emphasized how small, often random donations could make a disproportionate difference to enhance dignity, where they felt unable to make a meaningful difference:

“There was a one-off donation of a box of pots and pans, that I gave to one of the asylum seekers I was working with. And in our very final session a few weeks ago, that was the thing that she came back to… I was like, you know, ‘what are the pockets of peace during your life at the moment?’ Because generally her life is just very fearful, very stressful, difficult. She's like, ‘well, I really like it when I cook, there's my moments of peace. And that's partly because those pots and pans are the only thing that actually are mine. I don't really own anything else’, and I'm just like ‘oh God, those pots and pans, like, thank God that that we had this random donation of a box of pots and pans’. So yeah, I'm glad I was able to do that for her.” (P1, Social Prescriber)

### RQ3: practitioners’ perspectives on social prescribing

RQ3 investigated how community practitioners perceived the relevance of social prescribing for refugee support. This section explores how practitioners understood social prescribing, whether they saw it as relevant to refugee support, and where they identified gaps or mismatches.

### Understanding and familiarity with social prescribing

Across the sample, definitions of social prescribing clustered most often around a holistic, link-working model described as a non-medical, person-centred approach connecting people to community assets to address wider determinants of health. Further definitions framed it primarily as “signposting” or “brokerage” from statutory services into community activities, with some community practitioners critiquing a perceived gap between the time-limited NHS model and day-to-day practice. One practitioner stressed its origins in relieving GP workload. Yet uncertainty was also common: some interviewees would not offer a definition or immediately redirected the question back to the interviewer and a larger group expressed doubts about their definition, including admission of having researched the term for the interview.

For community practitioners, familiarity with social prescribing was inconsistent. A large group described indirect or practical contact with social prescribing, such as receiving referrals or collaboration. An equally large group reported no (meaningful) contact. An interviewee showed interest in creating a link with a local GP surgery to act as a receiving organisation in future: “I thought, well, I could go and give them a leaflet to tell them about us and say, we are happy to be a point of contact to support people” (P13, charity chair). Of those who had had contact, several described challenges in engaging with local social prescribers, with some describing unsuccessful attempts or concerns about their experiences. One example included a more positive or neutral experience then balanced with interactions that were more difficult to manage from a capacity perspective:

“I mean sometimes it seems almost a bit sporadic, like there's one particularly social prescriber. He's contacted us sometimes, he asked us for example for someone from our organisation to call a client of his who speaks Kurdish and just like introduce the organisation, which I'm like, okay, that's kind of interesting. We do have Kurdish speakers among our like staff volunteers, but it's not a huge population… Just one recent example, another case of a social prescriber. She actually came to our office with one of her clients and introduced her to the work and just showed her around personally… Another example actually. And this particular prescriber contacts us quite a lot, but sometimes they're a bit, I don't know if the word is insensitive, but more a bit much. For example, she once contacted us and the subject was “urgent action required”. And it was about, I think, an Iranian client who was suicidal and needed urgent help. And it was just a little bit insensitive. And we didn't have that sort of capacity to help them.” (P8, Integration Manager).

One interviewee reported more consistently positive delivery and results with social prescribing. In this community-embedded social prescribing project embedded in community structures, the manager emphasized the deep meaning in witnessing individual growth. In this example, specific cultural changes to the model (e.g., changing project terminology, model and delivery expectations to focus solely on community referral to activities and long-term monitoring rather than one-to-one talking sessions with a social prescriber, providing budget for activity referrals) had led to community uptake, exceeding all initial expectations:

But with the social prescribing and the way that it was advertised from the beginning, parents didn't understand what that meant. So when you talk about health and well-being, they think that that's a financial thing. It doesn't translate. But when you talk about social prescribing, they feel that that's a health thing. And they're like, well, there's nothing physically wrong with me. So they don't identify mental health, emotional health and trauma as physical. We try and encapsulate the whole thing. Well, you know, you can be unwell mentally and that's the same as if you've got a tummy bug because we try and normalise mental health and emotional health and well-being as is if you were poorly in other respects. But… there's no understanding there. So it was really hard trying to explain to them what we wanted the project to do in this kind of therapeutic way and improve wellbeing because it just didn't translate… we talked about enjoyment and happiness and fun and those sort of words to show the parents that's what we wanted for their children. We wanted them to have fun. We felt it was important for them to enjoy themselves while they were here. And out of that, from a reporting point of view, ticked the boxes for recovery because that's how you recover from trauma is to then do something fun and become happier in your new environment. So, I think a lot of parents were caught up with housing, finances, what was going on with them, making sure their kids were placed in school. That activities bit, that wasn't a priority. So we kind of took that away from the parent and made it a priority. And then that meant that they didn't have to deal with it. So I think that was really important for them as well and they're all so grateful. I've got lots and lots of lovely quotes from all of the parents about how important it was for their child to be placed on this activity, to help them, and that they're smiling again. So we've kind of worked with the families to see what would be best for them for social prescribing. And then I've fed that back to County and said, this is the only way it's going to work. Because a traditional six meetings, sitting down talking to a child, a young person about their feelings, about what they've been through. What can we do to help isn’t talking, that isn’t doing anything for them. We need to just get them into an activity. Get them engaged, make sure they're happy again. And the rest of it speaks for itself. (P2, Youth Service Manager).

### Perceived relevance and fit

Concerning the relevance of social prescribing for refugee support, a large group of participants, including all social prescribers, described social prescribing as aligned with community-led support, emphasising shared practices of holistic support, non-clinical navigation, and community participation. However, many participants also described caveats, including misalignment with NHS record-keeping systems, knowledge gaps around organisations or entitlements, or limited capacity: “they… have a kind of directory… and they did not even have the 5 or 6 that support people seeking asylum on it” (P14, GP). Some questioned whether the model was consistently effective and described concerns about role drift into gap-filling casework: “plugging holes where services aren’t there” (P17, Social Prescriber). A further large group did not or would not comment explicitly on alignment, or remained neutral, occasionally stating inability to say based on limited familiarity.

A majority of interviewees communicated that social prescribing could be relevant and useful in refugee support. Many described potential benefits in the model, describing its holistic, person-centred ethos and its potential to address isolation, provide advocacy, build trust, and connect people with community activities and entitlements. Social prescribers described that social prescribing’s “whole person” (P22, Social Prescriber) approach resonated strongly with refugee needs and could reduce invisibility and increase opportunities:

“So, there's the degree of invisibility amongst migrant populations… when they feel brave enough to come through the GP or they feel safe, they have some level of status, or they're either waiting for an asylum claim to come through, or they're waiting on a case and they feel a little bit safer, legally speaking. And then they go to the health care setting and then there’s a degree of visibility and feeling heard and seen and knowing what's available, because most of them don't know that there are services available for them, there are advocacy services available for them. There are food banks that they can access. There's a lot. But if you don't come across those services that can point you in the right direction and signpost you or refer you then… in terms of finding the services that will help the person, that's where the role becomes really important, because part of the social prescribing role is to do that research and to have that relationship with community organisations.” (P7, Social Prescriber).

Community practitioners noted benefits in signposting, light-touch support, and family-centred approaches, provided quality standards and appropriate follow-up were in place. Both community practitioners and social prescribers described that social prescribing could be an opportunity for increased advocacy in housing, welfare, and health statutory access:

“I've seen letters that social prescribers have written to advocate… [for] asylum seekers when they have been struggling with housing actually, and as well when they've struggled with social housing, writing letters of support, but also for things like reporting requirements for the Home Office… So I think they can be really strong advocates and they have a bit more time than health professionals to really understand that person… You know, to know that they can get free bus tickets or that they can get free prescriptions. I can't change what the Home Office does to you. I can't change your accommodation necessarily. I can advocate for you. But I do think, you know, social prescribers could really do some small things that really support that person to exist in a really tough situation.” (P14, GP).

### Identified gaps and concerns

Many community practitioners raised doubts or cautions about social prescribing’s relevance for refugee populations. Practitioners described barriers including GP gatekeeping, mistrust of statutory systems, and refugees’ fears of contact with the Home Office. Others noted resourcing and capacity problems, such as waiting lists and service saturation, or inconsistent capacity among social prescribers to be able to activate referrals or advocate effectively. Several practitioners described ‘light-touch’ standard referral models as insufficient when refugees’ circumstances are considered. Referral uptake or attendance was described as unrealistic without intensive accompaniment or trust-building: “There’s zero outcome from a one-sided referral” (P15, Wellbeing Manager).

Interviewees described that poorly controlled activities and lack of accompaniment risk retraumatising refugees, and expressed concern that the social prescribing referrals are inadequately evaluated to see whether receiving organisations are a good fit, are trained or have capacity to support: “If you just drop people into activities without support, it can do more harm than good” (P15, Wellbeing Manager). This exact concern was then also illustrated in reflections from a social prescriber, who described the following referral for a client:

“She loves to dance and she took two buses and went to a dance class… on a very limited income. And she called me that evening and said I was the only black face there. You didn't tell me… [but] I think social prescribing has great worth if the other supports are there to facilitate.” (P22, Social Prescriber).

### RQ4: lessons and best practice for future services

RQ4 synthesized lessons and best practices that could inform future social prescribing for refugee populations. Findings are organised into systemic lessons, practice-level lessons, training and workforce development needs, and evaluation considerations.

### Systemic and structural lessons

Across the interviews, many participants described underfunding, workforce strain, and lack of integration between NHS structures and community realities. Funding insecurity was often identified as undermining continuity of care and sustainable cooperation with the third sector: “Every year we do not know if we’ll still exist” (P5, Charity Director). Calls for longer-term, core funding to reduce burnout and project collapse were communicated by multiple community practitioners. This concern was also described by social prescribers, who noted that lack of funding and resources across community organisations limits onward referral. Even where social prescribers provide emotional support, lack of capacity undermines long-term outcomes for individuals: “You can listen and hold the story, but then there’s nowhere to send them” (P17, Social Prescriber).

Several interviewees described fragmentation and service inconsistency of service alongside excessive caseloads, constant recruitment and turnover, and the risk of burnout. They described that social prescribing imposes “five jobs in one” (P7, Social Prescriber), and stressed that emotional support and relationship-building—not just signposting—should be recognised as core outcomes. Some participants described the NHS framing of social prescribing as indicative of “a very medical mindset” (P7, Social Prescriber), with timetables that did not account for the strong emotional bonds between individuals. Social prescribers called for realistic recognition of the emotional labour involved and systemic redesign to match expectations with practice.

A smaller cluster of community and statutory representatives stressed the need for stronger systems and data infrastructures for social prescribing practice. This reflected concerns over knowledge management with high staff turnover and fragmented commissioning structures: “Every time staff leave, all the knowledge goes with them, and the system starts again” (P20, Regional Manager for Health Inequities).

### Practice-level lessons and best practices

Many community practitioners emphasized the importance of designing projects around safe, accessible, and culturally sensitive spaces. Several participants described that “healthcare settings are frightening for people who have been through the system” (P19, Schools Coordinator), and trusted community-based spaces were noted as potentially better settings for entry points to care. Participants described trauma-informed practice as essential. Community practitioners stressed that without explicit awareness of trauma triggers and the adoption of humane, flexible, and safe approaches, social prescribing risks alienating or retraumatising individuals. Trauma-informed practice was described as including specialist training and supervision as well as the design of safe community environments and flexible activities that allowed people to engage at their own pace: “You have to slow down—trauma does not run on timetables” (P16, Quality Assurance Teacher). Oher participants described the importance of co-design and ownership. Refugee-led and community-led design were described as enhancing confidence, trust, and sustainability of projects or interventions, as “co-design means they feel it’s theirs, not something done to them” (P4, Senior Researcher).

### Training and workforce development

A further set of lessons concerns skills and training, as well as structural and working conditions for social prescribers. Several community practitioners described knowledge gaps among social prescribers regarding trauma-informed practice, asylum law, or cultural factors. They emphasized the need for specialist training and structured supervision as without this, social prescribers and activities risk alienating refugees, or missing needs. One practitioner elaborated:

“I mean, it's entirely possible to alienate, to distance, almost to retraumatize somebody by taking them on a gorgeous, beautiful walk out where you share local history, because maybe you've gone a little bit overboard and you share so much local history. And now this the whole walk has become about you and about the fact that you own the knowledge. You are the keeper of the keys and therefore you don't really understand because you probably didn't follow half the stuff that I was saying… I was using weird vocabulary, antiquated vocabulary and now what I've done is that I have kind of shown that this place isn't for you, and you don't quite understand it… And where and with whom does the responsibility lie or is there a responsibility? I'm not sure, but I think there is definitely room for a lot of practical and practitioner training.” (P15, Wellbeing Manager).

This echoed concerns from social prescribers interviewed, who described the mismatch between their job expectations and the emotional realities of the role. Social prescribers emphasized that the social prescribing service provided to refugee individuals was dependent on the training and interest of the social prescriber involved, which they described often led to inadequate need recognition and therefore service provision: “It depends entirely on the prescriber—if they care and if they are trained” (P22, Social Prescriber); “Some prescribers just do not see the needs, so nothing happens” (P1, Social Prescriber).

### Recommendations for systems and infrastructure

Participants suggested a range of recommendations for strengthening practice. Multiple participants focused on systemic thinking, infrastructures and support systems. Some proposed dedicated roles for mapping services, developing centralised databases, and fostering active professional networks to improve awareness: “There should be a proper map of what’s out there, otherwise it’s random who gets referred where” (P16, Quality Assurance Teacher).

**Figure 1:**
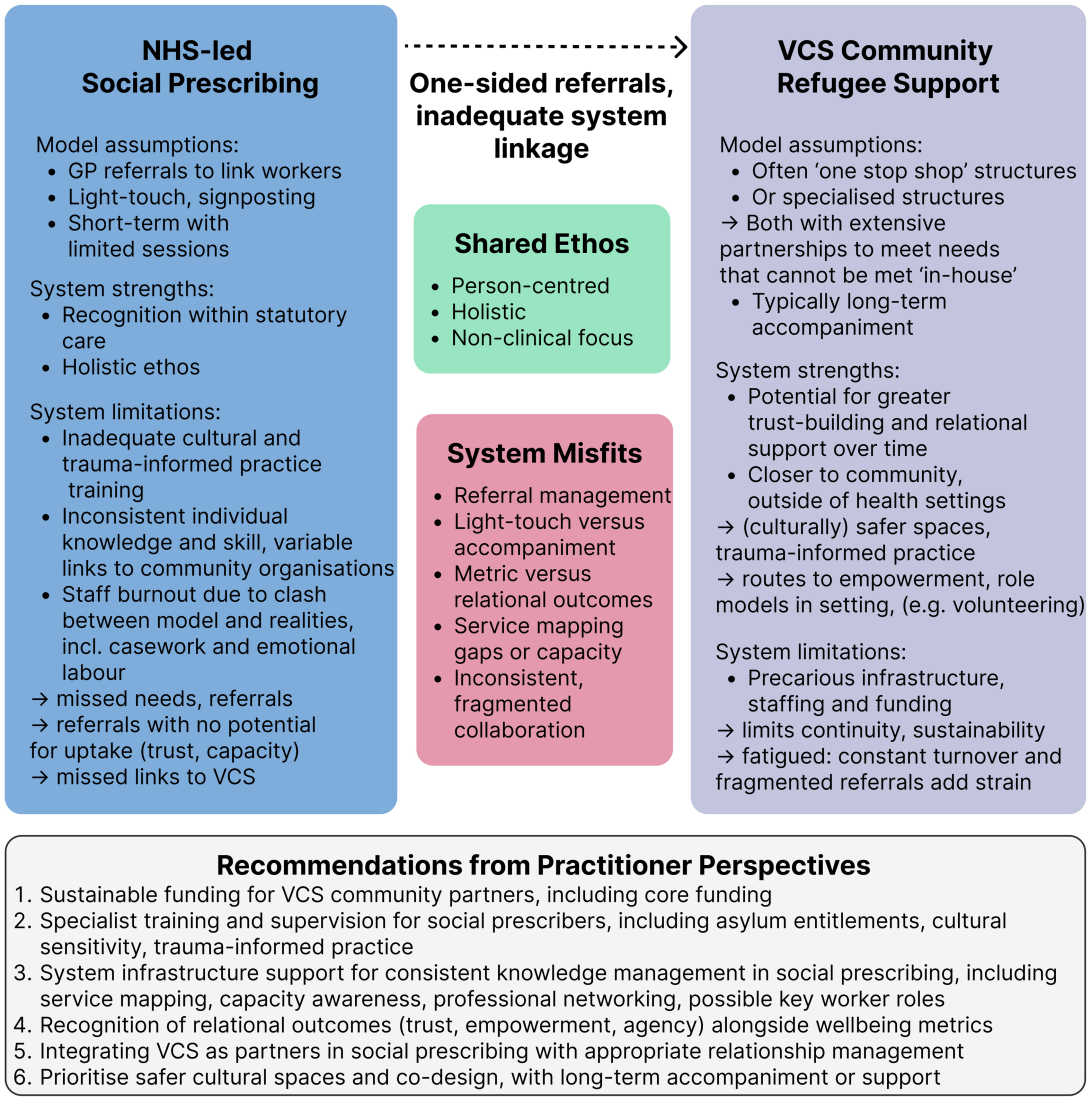
Findings summary visual.

Secure funding structures to ensure continuity of care and linkage with the community refugee support sector was also described within a holistic health system approach: “it’s systems on top of systems—nobody can see the whole picture” (P20, Regional Manager for Health Inequities). Social prescribers additionally suggested introducing key worker roles for longer-term support, and ensuring prescribers are not solely responsible for research, administration, and signposting (see Figure 1).

Finally, for all RQs, further direct quote examples can be found in Data sheet 5.

## Discussion

This study explored delivery models, challenges, resources, and practitioners’ perspectives related to social prescribing for refugee populations in the UK. Findings generated insights into how community-led refugee support and NHS-commissioned social prescribing services operate, where they converge and diverge, and what lessons can inform future practice in this space. The following discussion synthesizes findings in relations to each research question, examines using a dark logic framework, and considers broader implications for social prescribing and public health systems.

### Delivery models and methodologies

Findings revealed diverse delivery models across community-led and NHS social prescribing settings alongside key patterns in how support is organized and delivered across different settings. Community-led projects often emerged organically, rather than through formal commissioning, suggests organisational and structural growth in response to identified needs. Many developed “one-stop shop” models combining social, legal, educational, and health-related activities under one roof. This evolution appeared driven by three factors: individual needs for trusted and accessible spaces, practical considerations such as childcare availability, and funding constraints that pushed organisations to consolidate services rather than maintain narrow specialisations.

In contrast, social prescribing had more formalized structures centred on time-limited signposting through social prescriber. However, social prescribers consistently reported exceeding these parameters to provide sustained relational support in response to holistic needs, suggesting tension between prescribed models and practice realities. This finding aligns with previous research highlighting gaps between social prescribing policy frameworks and front-line implementation, particularly regarding the emotional labour and relationship-building that social prescribers undertake (32, 46).

### Challenges and resources in practice

Practitioners identified substantially more challenges than resources, painting a picture of a fragile refugee support ecosystem. Challenges operated at multiple levels: individual (trauma, language, mistrust), organizational (workforce burnout, funding precarity, training gaps), and systemic (hostile immigration policies, NRPF exclusions and entitlements at status transitions, dispersal). This multilevel analysis gives a first indication of how macro-level policy environments shape the conditions for meso-level organization capacities and micro-level service delivery and individual outcomes.

The finding that funding insecurity emerged as recurring across settings suggests structural rather than isolated problems and has important implications, affecting community organisations’ ability to retain staff, maintain services, and build sustainable partnerships. Short-term restricted funding creates conditions where organisations cannot invest in training, supervision, or evaluation infrastructure. This not only limits quality but also contributes to workforce burnout, furthering a cycle of instability. These findings resonate with broader critiques of commissioning practices in the UK NHS and voluntary sector, which increasingly require organisations to demonstrate specified measurable outcomes while providing inadequate resources for capacity-building or sustainability (47, 48). The overall picture reflects an under-resourced sector operating in a hostile policy environment, further suggesting that systemic underfunding exacerbates structural gaps.

Resources identified included staff commitment, volunteer contributions, organizational learning, and community partnerships. Notably, many of these resources were informal or precarious, representing individual goodwill rather than systematic infrastructure. That high turnover was described across settings suggests it is systemic rather than occasional. The reliance on volunteers, while clearly valued, raises questions about sustainability and whether unpaid labour is effectively subsidizing gaps in service provision. This represents a form of ‘shadow work’ that deserves greater policy recognition.

Participants’ critical views of formal scales and metrics suggests tensions between funder accountability and practitioner understandings of meaningful change. The emphasis on relational trust, safe spaces, and gradual empowerment as critical enablers aligns with established theory on therapeutic relationships and trauma-informed practice (49–51). However, findings reveal that these relational outcomes are rarely captured by standard evaluation metrics, rendering them invisible to commissioners and policymakers despite being central to practitioners’ and social prescribers’ understanding of impact.

### RQ3: practitioner perspectives on social prescribing relevance

Practitioners expressed mixed views on the relevance of social prescribing for refugee populations. Participants saw both alignment, particularly in terms of philosophical resonance in shared values, but also expressed caveats which suggest theoretical compatibility alongside structural and practical implementation barriers. These included GP gatekeeping (limiting access), short timeframes (mismatched with trust-building needs), inadequate social prescriber knowledge of refugee entitlements and services, capacity constraints in receiving organisations, and cultural mismatches in activities.

The finding that familiarity with social prescribing varied considerably, even among those on the frontline of refugee support and therefore most likely to receive referrals, suggests that social prescribing has not yet linked effectively with the third sector. Challenges in engaging often led practitioners to give up, suggesting structural barriers to effective partnership. Awareness of social prescribing as a relevant framework for refugee populations was broadly lacking. This may reflect how social prescribing has been communicated, implemented, and evaluated primarily within mainstream healthcare contexts.

Summarising across findings demonstrates that resourcing for social prescribing could create or exacerbate inequities. This is seen firstly in social prescriber allocation, as whether refugees access support depends on which prescriber they happen to encounter, particularly on their competencies, knowledge, and interest in refugee-specific topics. Practitioners’ concerns about inappropriate referrals – exemplified by a social prescriber who referred a Black woman to an all-white dance class – illustrate substantive risks where cultural competence is lacking. This connects directly to the dark logic framework discussed below: well-intentioned interventions can cause harm where they fail to account for power dynamics, cultural contexts, and lived experiences of marginalisation.

The tension between standardized protocols (six-twelve contact points in social prescribing) and actual practice (ongoing relational support) suggests a fundamental mismatch between commissioning assumptions and refugee support realities. This raises questions about whether existing social prescribing is adequate for refugee individuals but requires adaptation, or whether other approaches such as key worker systems, multi-agency statutory coordination, and long-term accompaniment and casework models are needed.

### RQ4: lessons and best practices for future services

Synthesizing across findings, several key lessons emerged. First trauma-informed presence was seen to require not just individual practitioner awareness but systemic design: flexible timeframes, safe physical environments, cultural competence, and supervision structures. Second, sustainable funding models are seen as essential—not just for service delivery but for organizational capacity, training, knowledge management, and partnership maintenance. Third, evaluation frameworks were seen to need to recognise relational and empowerment outcomes as legitimate measures of success, not merely proxies for ‘harder’ health outcomes.

Fourth, co-design and community ownership were seen to enhance trust, relevance and sustainability. Fifth, service mapping and knowledge management systems were felt needed to address information gaps that currently leave referrals to chance. Sixth, training in trauma-informed practice and asylum contexts were seen as required for social prescribers working with refugee populations. Finally structural integration between NHS social prescribing and voluntary refugee support was seen to require genuine partnership models, not just outward referral pathways, recognizing community organisations as partners in health delivery.

These lessons contribute to growing evidence that social prescribing effectiveness depends heavily on contextual adaptation and adequate resourcing of community infrastructures. For refugee populations specifically, they suggest that social prescribing delivery must centre on trust-building, sustained accompaniment, and cultural safety.

### Dark logic: systematic analysis of unintended harms

Drawing on Bonell and colleagues’ framework (39) for theorizing harmful consequences of public health interventions, our findings also suggest several potential ‘dark logic’ pathways through which social prescribing may generate unintended harms for refugee populations. We structure this analysis using Context-Mechanism-Outcome (CMO) configurations to systematically identify how specific features of social prescribing implementation interact with structural conditions to produce unintended harms. This approach also distinguishes between unintended harms directly attributable to social prescribing design choices and those arising from broader structural barrier that social prescribing interacts with (but does not create).

### CMO configuration 1: cultural mismatch and compounded isolation

Context: Social prescribing services operate without refugee-specific training infrastructure or consistent and comprehensive service mapping of culturally appropriate organisations. Mainstream community activities are designed for general populations without adaptation for refugee experiences.

Mechanism: Without adequate cultural competence and service mapping, social prescribers make referrals based on limited knowledge of refugee-serving organisations or the cultural appropriateness of activities. Referral processes may lack safety-checking mechanisms or protocols for cultural matching.

Outcome: Referrals can compound isolation rather than addressing it through inadequate referrals and cultural mechanisms. The example of the Black woman referred to an all-white dance class illustrates this example: “She loves to dance and she took two buses and went to a dance class… on a very limited income. And she called me that evening and said I was the only black face there. You did not tell me.” (P22, Social Prescriber). Such experiences not only fail to address isolation but actively erode trust in statutory services, potentially deterring future help-seeking. In this case, financial cost of attendance also compounds material deprivation. In sum, when social prescribers lack knowledge of refugee-specific services, referrals function as a lottery with potential for significant unintended harm.

Attribution: This harm is directly attributable to social prescribing’s referral mechanisms where this operates without adequate cultural competence and service mapping, structural issues which are both remedial through system-level investment in training and information infrastructures. The specific mechanism through which social prescribing reproduces unintended harm stems from infrastructure design, which interacts with wider structural issues of racism and social exclusion and may also be relevant to other statutory roles involving referrals (e.g., social workers, health visitors, asylum support caseworkers, housing officers, ESOL coordinators).

### CMO configuration 2: time-limited models and risk of retraumatisation or relationship ruptures

Context: NHS commissioning frameworks mandate short-term social prescribing interventions, typically six to twelve sessions, which may be accessed by refugee populations experiencing ongoing trauma, legal precarity, and systemic exclusions which requires more sustained accompaniment.

Mechanism: Standard social prescribing activities requiring personal disclosure, physical proximity with strangers, or engagement with authority figures may risk triggering retraumatisation if delivered within rigid timeframes that emphasizes brief signposting. When practitioners lack training in trauma-informed approaches, they may inadvertently create unsafe environments or push individuals to engage at paces that are harmful to them. This situation arises through tension between standardised framework protocols and the perceived sustained relational engagement needed for trust-building: “They call it light-touch, but these cases do not just end in eight weeks” (P1, Social Prescriber). If social prescribers must terminate relationships according to protocol despite perceived need for longer accompaniment, this creates relationship ruptures.

Outcome: Premature termination of support relationships may recreate refugees’ experiences of institutional abandonment and forced separation, potentially retraumatising individuals. For refugees who have experienced institutional violence or neglect, these failures reinforce cynicism and withdrawal with implications for future help-seeking and willingness to engage with health systems.

Attribution: This harm is directly attributable to social prescribing service design rather than to pre-existing structural barriers, specifically commissioning frameworks that impose standardise timeframes with inadequate trauma-informed training provision. These are remediable through flexible protocols for complex cases and specialist training. The conditions that create the risk for retraumatisation are defined by structural factors including migration experience, racism and systemic violence, and are relevant to other statutory services (e.g., Home Office interviews, police encounters, court proceedings, housing assessments, health or social services interventions).

### CMO configuration 3: capacity overwhelm and cascading service erosion

Context: Community organisations operate with precarious funding, small teams and heavy volunteer dependence. Social prescribing pathways generate referrals into these organisations without capacity assessment, resource allocation, or accountability mechanisms for ensuring organisations can respond.

Mechanism: When underfunded community organisations receive referrals they lack capacity to serve, cascading harms occur through capacity overwhelm as social prescribers make referrals where demand cannot be absorbed. This may also occur in the absence of capacity from appropriate statutory care services, as is likely in the earlier example from a receiving organisation: “the subject was ‘urgent action required’. And it was about, I think, an Iranian client who was suicidal and needed urgent help… And we did not have that sort of capacity to help them.” (P8, Integration Manager). Referral processes lack accountability mechanisms for ensuring organisations or services can meet needs or for managing expectations when they cannot.

Outcome: Cascading harms amplify ongoing structural issues that accelerate staff burnout in receiving organisations, service degradation or closure to new participants. In terms of individual experience, this also creates unintended harm through the experience of long waiting lists leaving individuals without support and trust erosion through one-sided referrals that cannot be served: “you can listen and hold the story, but then there’s nowhere to send them” (P17, Social Prescriber).

Attribution: The underfunding of the voluntary sector and availability of specific crisis and non-crisis statutory services (e.g., specialist mental health) represents a broader structural problem predating social prescribing. However social prescribing system design that generates demand without corresponding capacity investment in receiving organisations amplifies wider structural pressures that contribute to capacity overwhelm. This represents an amplification effect where social prescribing interacts with but does not solely cause the harm, and is also an effect broadly relevant to many other statutory services.

### CMO configuration 4: workforce burnout and inequitable access

Context: Social prescribers work within NHS structures that frame their role as light-touch signposting, while the emotional realities of supporting refugees with complex needs necessitates sustained relational work. Formal adequate supervision and trauma support infrastructure may be inadequate or absent.

Mechanism: Social prescribers described feeling burdened by emotional labour without adequate support systems: “we are listening like therapists but without the supervision” (P1, Social Prescriber). They emphasized that the social prescribing service provided to refugee individuals was dependent on individual prescriber training and interest, which often led to inadequate need recognition: “It depends entirely on the prescriber – if they care and if they are trained” (P22, Social Prescriber). Social prescribing is described as imposing “five jobs in one” (P7, Social Prescriber) without support or recognition for the emotional labour involved.

Outcome: Social prescriber burnout creates turnover that disrupts relationships, depletes organisational knowledge and networks to community organisations, and amplifies inconsistent access to support depending on which prescriber a refugee encounters. This variability means social prescribing effectiveness becomes a lottery rather than representing a consistent and more equitable service. For service users, inconsistent support and relationship disruptions reinforce institutional disinterest.

Attribution: Workforce burnout represents a widespread challenge across statutory and community sector roles involving emotional labour with populations who have complex needs, which is not a problem unique to or created by social prescribing. However, specific design features of social prescribing amplify burnout risk and its unintended harmful consequences: light-touch signposting framing compared to complex relational work realities; inadequate supervision; and lack of mandatory training requirements. While refugee needs stem from pre-existing conditions, the mechanism inequitable service—where quality depends on individual prescriber capacity rather than systematic infrastructure—is attributable to social prescribing design choices that are remediable through realistic role expectations, mandatory supervision, specialist training, and workload management recognising emotional labour as core work.

This CMO analysis suggests that addressing identified unintended harms requires distinguishing between design-attributable problems remediable through reform (training and supervision infrastructure, flexible protocols for complex cases), amplification effects requiring both social prescribing reform and broader structural reform (capacity assessments, sustainable voluntary sector investment), and pre-existing conditions requiring cross-governmental and societal action (hostile immigration policies, systemic racism). These distinctions are critical to understanding that the challenges are not unique to social prescribing and for understanding its role in either reproducing or addressing health inequities for refugee populations.

### Strengths and limitations

The strength of this study lies in capturing under-represented practitioner knowledge across varied contexts through an exploratory qualitative framework. Analytical rigor was pursued through purposive sampling aimed at maximum diversity, iterative data collection and analysis, and consensus-building with other authors. The central inclusion of many practitioner voices enabled grounded insights while minimizing researcher over-interpretation. Triangulation was supported through convergence of findings across diverse project types and practitioner roles, suggesting conceptual saturation.

The study followed COREQ guidelines for reporting qualitative research, enhancing transparency and reproducibility (40). The integration of Bonell and colleagues’ dark logic framework represents a methodological strength, enabling systematic theorization of potential harms alongside benefits (39). This addresses a critical gap, where social prescribing delivery is also analysed for potential harm rather than exclusively intended positive outcomes.

However, several significant limitations must be acknowledged, which constrain the scope and generalizability of our interpretations. First, the study primarily reflects perspectives of project leads and social prescribers, with missing voices from the statutory sector, commissioners, and most importantly, refugee individuals themselves. Without direct inclusion of refugee participants, we cannot verify whether practitioners’ accounts align with refugees’ lived experiences of social prescribing services or whether potential harms manifest in the ways described. Future research should prioritize direct inclusion of refugee participants to understand their lived experience of social prescribing services and validate or challenge practitioner interpretations. The positionality of the research team as a female team without direct experience of forced migration requires reflexive acknowledgement, particularly given that the majority of asylum seekers are young men. This may have shaped our interpretation of practitioner accounts and limited our ability to probe certain aspects of gendered experiences.

Second, despite extensive targeted recruitment attempts, various viewpoints could not be represented as planned in the purposive sampling, including religious or faith-based community services, food banks, homeless shelters, and social workers in statutory settings. Our sample may have been inadvertently skewed towards practitioners with problematic experiences of social prescribing. We may be missing perspectives from organisations with more positive experiences of receiving social prescribing referrals, from commissioners who design services, or from social prescribers working in better-resourced settings. This potential sampling bias may mean that our findings may overrepresent challenges and underrepresent effective practice.

Third, the non-generalizable qualitative design further limits transferability and findings are not statistically generalizable. While theoretical saturation was reached within our sample, the purposive nature of sampling and convergence of themes suggests conceptual relevance rather than empirical representativeness across all social prescribing contexts or all refugee support settings. Caution is therefore warranted in extrapolating findings beyond the contexts studied. The breadth of settings and convergence of themes across diverse practitioner roles supports the plausibility and potential transferability of insights, but empirical validation through larger-scale studies would be needed to establish prevalence or frequency of identified patterns. Additionally, while this study provides important insights for the UK context where social prescribing is well-established, transferability to countries where social prescribing is not delivered or where refugee support systems differ substantially is limited. Findings may, however, offer broader conceptual lessons about community-led approaches to refugee health and wellbeing that could inform practice in diverse international contexts, subject to contextual adaptation.

Fourth, regarding reflexive practice for thematic development and methodological decision-making, while team members met to hold structured reflection meetings, more systematic documentation of how our positionality might be shaping our interpretation of emerging themes through artefacts such as reflective journalling could have further strengthened methodological transparency. Additionally, ongoing reflexive dialogue to identify whether positive descriptions of social prescribing were being underweighted, to actively search for disconfirming cases, or to continue recruitment specifically targeting organisations with positive social prescribing experiences would have strengthened the analytic process and rigour.

Fifth, the retrospective application of the dark logic framework means we theorized potential harms based on practitioner accounts rather than directly observing harmful outcomes in refugee populations. While CMO configurations are grounded in practitioners’ descriptions of mechanisms and consequences they observed, these remain practitioner interpretations rather than empirically documented harms experienced by refugees. The plausibility of identified dark logic pathways is supported by convergence across multiple practitioners’ accounts and consistency with theoretical frameworks, but empirical verification would require direct research with refugee services users documenting their experiences of harm or benefit.

Finally, the cross-sectional nature of interviews means we captured practitioners’ perspectives at a single time point rather than tracking how social prescribing implementation and impacts evolve over time. Longitudinal research would be needed to understand whether identified challenges represent implementation problems or persistent structural features and whether reported harms manifest as short-term disruptions or long-term consequences for refugee populations.

These limitations collectively mean that while our findings provide valuable practitioner-based insights into potential challenges and harms, they represent hypotheses and conceptual frameworks requiring empirical validation through direct research with refugee populations, larger-scale studies, and longitudinal designs. Interpretations should be understood as exploratory and practice-informed rather than definitively establishing prevalence, causality, or generalizability of identified patterns.

## Conclusion

This exploratory qualitative study provides practice-informed insights into how social prescribing delivery models intersect with refugee support contexts. Practitioner accounts suggest that community refugee support work emphasizes principles of potential relevance to social prescribing: trauma-informed design; culturally safe spaces; recognition of relational and empowerment outcomes; and sustained accompaniment. However, practitioners also identified significant challenges and potential harms when social prescribing systems encounter refugee populations without adequate adaptation.

Based on practitioner perspectives, findings suggest that for social prescribing to be effective and safe for refugee populations, systemic adaptations may be needed: long-term funding models enabling organisational sustainability and workforce support; training in trauma-informed practice and asylum contexts for social prescribers; comprehensive service mapping and knowledge management infrastructures; evaluation frameworks validating relational, empowerment, and cultural safety outcomes; and policy environments that mitigate rather than exacerbate barriers to health and wellbeing. Practitioners’ accounts indicate that without such adaptations, there is a risk that social prescribing may inadvertently reproduce rather than address health inequities. However, these interpretations are based on practitioner perspectives rather than direct documentation of refugee experiences and require empirical validation through research directly involving refugee service users.

The study’s contribution lies is foregrounding practitioners’ practice-based knowledge about how existing systems may inadequately respond to refugee populations’ needs when operating in silos or without adequate cultural and trauma-informed adaptation. By applying Bonell and colleagues’ dark logic framework to practitioner accounts, the analysis demonstrates a methodology for theorizing potential harms alongside intentional benefits when adapting interventions for populations experiencing trauma, legal precarity, and systemic exclusion. The identified CMO configurations provide testable hypotheses for future research examining whether and how these potential harm pathways manifest in refugee experiences of social prescribing.

Further research is urgently needed to validate these practitioner-informed insights through direct engagement with refugee populations, to examine whether identified challenges and harms are experienced as described, and to document examples of effective practice where social prescribing successfully supports refugee health and wellbeing. Such research should employ participatory methods centering refugee voices and agency, include larger and more diverse samples across different social prescribing models and refugee support contexts, and use longitudinal designs to understand how impacts evolve over time. Only through such empirical validation can the field move from practitioner-based hypotheses to evidence-based guidance for equitable social prescribing implementation.

## Data Availability

The datasets presented in this article are not readily available because this is not aligned with written consent given by participants for excerpt data usage in publications and presentations only. Requests to access the datasets should be directed to Victoria Touzel, victoria.touzel@uni-bielefeld.de.
