# Supplementary material for: Health system lessons from community practice: a qualitative study rethinking the role of social prescribing for refugee populations
Source: Front Public Health. 2026 Jan 26;13:1739953. doi: 10.3389/fpubh.2025.1739953 (PMC12883642; doi:10.3389/fpubh.2025.1739953)
Supplement: Supplementary file 1 [file Data_Sheet_1.PDF]

UNIVERSITÄT BIELEFELD

Faculty of Public Health
Prof. Dr. Doreen Reifegerste

Named contact for this study:
Victoria Touzel
victoria.touzel@uni-bielefeld.de

1. INFORMATION FOR PARTICIPANTS

Thank you for your interest in our study "Bridging Social Support and Social Prescribing: Exploring Methodologies and Insights from Refugee Projects."

Please read the following information carefully.

Study Purpose

This study examines the methodologies used in social projects supporting refugee populations, with a particular focus on how these initiatives address key challenges and barriers. It explores how practitioners align their work with social prescribing models and assesses the relevance of such approaches in refugee support. Additionally, the study seeks to identify best practices and lessons learned from existing social projects to inform future initiatives. By engaging experts working directly with refugee communities, the research aims to highlight effective strategies for improving access to social support, integration services, and overall well-being.

Responsible for the Study

The study is being conducted by the University of Bielefeld, Faculty of Public Health, AG 4 Prevention and Health Promotion, under the direction of Prof. Dr. Doreen Reifegerste and Victoria Touzel.

Study Procedure – What to Expect

As part of this study, you will participate in an expert interview exploring methodologies, challenges, and best practices in social projects that support refugee populations. The interview will focus on your professional experiences, particularly in relation to social prescribing and other structured interventions aimed at improving refugee well-being. Your insights will contribute to identifying effective approaches to refugee support, informing future initiatives, and strengthening the role of social projects in this context.

The interview will last approximately 45 to 60 minutes and will be conducted online via Zoom. You may choose to keep your camera off if preferred; although the Zoom

function records both video and sound, the video track will be deleted after the interview and only the audio will be saved as a mp3 file.

We will collect limited personal data, such as your professional role, demographic details and bank details for the purpose of renumeration, in accordance with Articles 6 of the GDPR. Detailed information about data processing and protection measures is provided in the attached privacy policy. As compensation for your participation, you will receive a transfer of £50. This can also be made as a donation to the organisation.

Your contact details will be securely stored until December 31, 2025. The anonymized research data will be retained for up to 10 years.

Benefits and Risks

By participating in this study, you will contribute to a deeper understanding of effective methodologies and best practices in social projects that support refugee populations. Your insights will help improve future initiatives by identifying key challenges, innovative approaches, and the role of social prescribing in refugee support. This research aims to inform policymakers, practitioners, and organizations working in this field, ultimately enhancing the quality and accessibility of services for refugees.

While the interview will focus on professional experiences, discussing challenges in refugee support work may bring up sensitive topics. If any questions make you uncomfortable, you are free to skip them, pause, or discontinue the interview at any time. Additionally, you may contact the research team if you have any concerns.

Voluntary Participation

Participation in this study is voluntary. You can withdraw from the study at any time without providing a reason, and you will not face any negative consequences for doing so.

Data Usage

The data collected in this study will be treated confidentially. The handling of your personal data is explained in detail in the attached privacy policy.

The research data will be published in a summarized form, such as in academic journals. After the completion of the study, the anonymized data will be securely stored in an archive that is not publicly accessible. The potential for future use of the data is currently uncertain. The data will only be published in a form that ensures no conclusions can be drawn about specific individuals. This process follows the guidelines of the German Research Foundation (DFG) for ensuring the quality and integrity of the research.

Faculty of Public Health
Prof. Dr. Doreen Reifegerste

Named contact for this study:
Victoria Touzel
victoria.touzel@uni-bielefeld.de

2. CONSENT TO PARTICIPATION AND DATA PROCESSING

I have read and understood the participation information and the accompanying data protection declaration for the study *“Bridging Social Support and Social Prescribing: Exploring Methodologies and Insights from Refugee Projects”.* I have received sufficient information and had the opportunity to ask questions via email before the study began.

I understand that my participation in this study is voluntary and that I will not face any disadvantages if I choose not to give my consent. I also acknowledge that I can withdraw my consent—either fully or partially—at any time, with future effect. No justification is required for my withdrawal, and no negative consequences will result. The lawful processing of my data prior to my withdrawal remains unaffected. If I revoke my consent before my data is anonymized, my personal data will be deleted immediately.

I confirm that I have downloaded and received a copy of the information for participants document, which includes the data privacy statement, and this consent form.

By signing below, I voluntarily consent to participate in the study and to the processing of my personal data as described.

Name (in block letters):

Place, Date, Signature:

Thank you for your cooperation and trust.

Faculty of Public Health
Prof. Dr. Doreen Reifegerste

Named contact for this study:
Victoria Touzel
victoria.touzel@uni-bielefeld.de

3. INFORMATION ON THE PROCESSING OF PERSONAL DATA (DATA PRIVACY STATEMENT)

Information on the Processing of Personal Data in the Research Project:

"Bridging Social Support and Social Prescribing: Exploring Methodologies and Insights from Refugee Projects"

In accordance with Articles 13 and 14 of the EU General Data Protection Regulation (EU-GDPR), Bielefeld University is providing this data privacy notice to inform participants about the processing of their personal data within the research project.

For definitions of terms such as "personal data," "processing," "data controller," or "third party," please refer to Article 4 of the EU-GDPR.

1 Contact Details

Data Controller:

Universität Bielefeld
Universitätsstraße 25 D-
33615 Bielefeld Tel:
0521 / 106 – 00
E-Mail: post@uni-bielefeld.de
Web: https://www.uni-bielefeld.de

Project Team:

Prof. Dr. Doreen Reifegerste
E-Mail: doreen.reifegerste@uni-bielefeld.de
Tel.: 0521-106-67807

Victoria Touzel
E-Mail: victoria.touzel@uni-bielefeld.de

Data Protection Officer:

The Data Protection Officer can be reached by mail at the address of the responsible institution.
Tel.: 0521 106-5225
E-Mail: datenschutzbeauftragte@uni-bielefeld.de

2 Legal Basis for Data Processing

The processing of personal data in this research project is based on the explicit consent of the participants, in accordance with Article 6(1)(a) of the EU-GDPR.

3 Purpose of Data Collection and Processing

Personal data will be collected and processed for the following purposes:

For interview invitations and scheduling:

- Name, email address, and, if applicable, phone number
- Bank details for the purpose of renumeration

For analysis of research data:

- Audio recordings of interviews (voices)
- Demographic information related to professional background, sector of work, and experience in refugee support

Personal data will only be collected and used for the purposes described in this study and will be handled exclusively within the research project at Bielefeld University.

Some personal data will be requested in advance and received and held in Outlook (e.g., name, organisation name, bank details for transfer, gender, role within the organisation, length of service in the organisation, area of work). Once the interview has been completed, relevant information will be transferred to the administration office for payments at Bielefeld University. Once the audio files (voice) have been transcripted, the anonymised relevant information will be added to the beginning of the transcript (referring to: gender, role within the organisation, length of service, area of work).

The interview data will be collected online via the Zoom platform account with Bielefeld University. By using Zoom, audio and video recordings will be made, and participants may choose to keep their camera off during online interviews. This file is saved as an mp4 file by Zoom. The video recordings will then be deleted immediately using DaVinci video editing software, where the video track will be deleted and only the audio track kept, finally exporting and saving the file as an mp3 (audio only, including voice recordings) file. DaVinci only saves on the local server, so this process does not involve uploading this content or sharing it with another provider to delete the video track. The audio recordings will be transferred to Sciebo. Sciebo is a GDPR-compliant cloud storage service for universities in Nordrhein-Westfalen in

Germany. After these recordings are transferred, they will be deleted on the terminal device and in the Zoom account used for the interview.

Bielefeld University's data protection information on the use of Sciebo can be viewed here: https://www.uni-bielefeld.de/einrichtungen/bits/services/kuz/sciebo/datenschutz/index.xml

Bielefeld University's data protection information on the use of Zoom can be viewed here: https://www.uni-bielefeld.de/einrichtungen/bits/elearningmedien/lernplattformen/zoom/datenschutz.xml

The audio recordings will either be transcribed by a research assistant working on the project using MaxQDA, or automatically transcribed using f4x and then checked in MaxQDA. During the transcription process, all personally identifiable information will be anonymized. After transcription, all audio recordings will be permanently deleted. The transcripts will be stored on Sciebo.

Personal contact details and the requested information above will be held in Outlook only for as long as is necessary to arrange the interviews, transcripts and renumeration, after which time correspondence relating to the interviews will be deleted.

4 Data Access and Transfer

Access to the data is strictly limited to the researchers and research assistants working on the project at Bielefeld University. The collected personal data will not be shared with third parties or transferred outside the EU.

A contract may be agreed with f4x for the purpose of transcribing data (audio files e.g. voice). If this is the case, a contract will be agreed with f4x that meets the processing requirements for GDPR.

F4x'S contact details are as follows:

dr. dresing & pehl GmbH

Deutschhausstrasse 22a

35037 Marburg

Deutschland

https://www.audiotranskription.de/en/imprint/

The details of how f4x ensures their services are GDPR-compliant can be found here:

https://www.audiotranskription.de/en/f4-features/f4-gdpr/

5 Retention and Anonymisation

Contact information will be deleted after study completion, no later than **December 31, 2025**. The anonymized research data from the interviews will be securely stored for **10 years**, in accordance with the data retention guidelines of Bielefeld University.

6 Publishing Research Findings

Research findings will be presented in a summarized format, such as in academic publications or conference presentations. If individual cases are referenced, they will be presented in a way that does not allow for identification of any specific person.

This research project follows the recommendations of Bielefeld University and the European General Data Protection Regulation (EU-GDPR) regarding research quality and data security.

7 Withdrawing Consent

Participants have the right to withdraw their consent at any time. The withdrawal of consent does not affect the legality of data processing conducted prior to the withdrawal (Article 7 EU-GDPR).

8 Rights of Participants as Data Subjects

As a data subject, you have the right to:

- Request information about your stored personal data (Article 15 EU-GDPR).
- Request correction of inaccurate personal data (Article 16 EU-GDPR).
- Request deletion or restriction of processing, or object to data processing (Articles 17 and 18 EU-GDPR).
- If applicable, request data portability for any data processed through automated means (Article 20 EU-GDPR).

To exercise these rights, please contact the research team using the contact details provided above. Complaints and concerns can also be directed to the university's Data Protection Officer. Furthermore, you have the right to lodge a complaint with the relevant data protection supervisory authority (Article 77 EU-GDPR).

Important Notice: After December 31st 2025, due to the complete anonymization of the data, **it will no longer be possible to modify, access, or delete individual records.** As a result, rights related to data access, correction, restriction of processing, or objection under Articles 15–21 of the GDPR will no longer be applicable beyond this date.

For any further questions regarding participation, voluntary withdrawal, or data protection, please contact:

Lead Researcher:

Victoria Touzel
E-Mail: victoria.touzel@uni-bielefeld.de
