# Supplementary material for: Health system lessons from community practice: a qualitative study rethinking the role of social prescribing for refugee populations
Source: Front Public Health. 2026 Jan 26;13:1739953. doi: 10.3389/fpubh.2025.1739953 (PMC12883642; doi:10.3389/fpubh.2025.1739953)
Supplement: Supplementary file 2 [file Data_Sheet_2.pdf]

Touzel, Victoria Nastassja

Subject: Request for grey literature or contacts for expert interviews

Dear Team at (Charity Name),

I am an international researcher and doctoral student (profile here), focusing on researching social prescribing as a system approach for refugee populations. Within this work, I am leading a rapid realist review evaluating social prescribing and social-capital based interventions (which have similar mechanisms to social prescribing and can be reproduced) for refugee populations internationally. The final included social interventions may take many forms that still fit under the umbrella of social prescribing depending on their methodology; think here of arts and music, sport, signposting and awareness raising, employment and volunteering, befriending, mentoring, nature-based activities for a start. As you may imagine, this is a challenging review process, and myself and my team imagine that the most valuable evidence may be available from “grey” sources, meaning outside of university settings. We are therefore proactively reaching out to people who may be able to support particularly with grey literature recommendations (grey literature means literature that isn’t formally published, and may include project reports, evaluation reports, and articles in non-scientific contexts).

I’m getting in touch with yourselves as part of my review process, where I’m contacting the most relevant organisations based on a search in charity registers for England, Wales, Scotland and Northern Ireland. I would be delighted to hear from you if there is grey literature from your projects with refugee populations that you can share with me, whether publicly published or not. In addition, in case you have heard of other relevant projects, interventions and ongoing work, I also wanted to ask if you may have any further literature or organisation recommendations for me?

It may be that you don’t specifically have any written evidence that you can share, but are interested in sharing your learnings as colleagues and practitioners in an expert interview. I will be conducting conversations with diverse organisations working with or delivering social projects for refugee populations, and would be delighted to hear from you if this is the case so we can start that conversation.

I would be very grateful to hear from you and wish you all the best in your very needed work!

With very best wishes,

Vikki Touzel
