# Supplementary material for: Health system lessons from community practice: a qualitative study rethinking the role of social prescribing for refugee populations
Source: Front Public Health. 2026 Jan 26;13:1739953. doi: 10.3389/fpubh.2025.1739953 (PMC12883642; doi:10.3389/fpubh.2025.1739953)
Supplement: Supplementary file 3 [file Data_Sheet_3.PDF]

Bridging Social Support and Social Prescribing: Exploring Methodologies and Insights from Refugee Projects

Preamble (5 minutes)

Thank you very much for taking the time for this interview. My name is Vikki Touzel, and I would like to talk with you about your experiences and perspectives as a practitioner working in social projects with refugees.

About the Study

This study explores the approaches used in social projects that assist refugees, with a focus on understanding how these initiatives work and what lessons we can learn from them. Specifically, it examines how these projects do or do not align with social prescribing as part of the UK's health system. Additionally, the research aims to identify best practices and lessons learned from existing social projects to help shape future initiatives. By engaging experts like you who work directly with refugee communities, we seek to highlight system strategies for improving access to services, social support, and overall well-being.

Data Handling and Your Rights

Your participation in this study is entirely voluntary. You have the right to withdraw at any time without providing a reason, and doing so will have no negative consequences. With your consent, this interview will be recorded, and all data will be handled in strict compliance with data protection regulations. Any personally identifiable information will be anonymized, and recordings will be deleted after transcription. The anonymized research data will be securely stored and used solely for academic purposes, such as publications and presentations, in a manner that ensures no individual can be identified. More information about this handling is included in the "Information for Participants" document previously shared with you.

Do you have any questions before we begin to discuss the interview process?

Okay. During our conversation, I will ask you a series of questions. Please feel free to share whatever comes to mind and what you consider important. There are no "right" or "wrong" answers—your insights and experiences are what matter most. Take as much time as you need to respond. If I use a term you do not understand, please let me know and I'll rephrase.

During the interview, you are welcome to take a break at any time. If you feel uncomfortable with any topic or question, please let me know.

If you agree, I would like to record the interview using the built-in recording function of Zoom. You will see an indicator in the meeting window once the recording starts. We can also record the session using only audio, if you prefer this – in which case, please turn off your camera.

Are you comfortable with this approach?

Thank you!

If you have no further questions, I will now begin the recording.

Introduction (5 minutes)

1. **Personal Context and Motivation**:
    - "Can you start by sharing a bit about your background and about how you first became involved in projects that support refugees?"

Understanding project approaches (30 minutes)

Think of a refugee support project that you are actively involved in or one that you were very closely involved with in the recent past—one that addressed information needs, such as signposting, or helped build resources like connections or skills for long-term security:

2. **Project context building**:
    - "Could you walk me through the story of this project from the beginning? How did it come about?
        - "What was its main purpose in that context?"
        - "How was it first and later financed?"
    - "Which groups of individuals – refugees and others – were involved and how did they become involved?"
    - "Who delivered within the project and what were their roles? How did those partnerships work?"
    - "What did the delivery of the project look like day-to-day? Can you describe the main activities?"
    - "What types of person benefited the most from the project and why?"
    - "What types of person faced greater challenges to participating and why?"
    - "Did language or cultural differences, or lived traumatic experiences, come up as challenges for individuals in the project?"
    - "Were the activities tailored to different groups within the refugee community? If so, how did you adjust the approach?"
3. **Measuring impact**:
    - "Could you describe the journey of an individual through the project over time?"
    - "What changed for them during this time and how could you tell those changes took place?"
    - "How was the project evaluated, if it has been?"
    - "Were there any unexpected effects from the evaluation or other experiences?"
4. **Community and stakeholder challenges:**
    - (If not already asked/covered): "Who was involved in first initiating the project from the wider context (e.g., local governments, health services, community groups)?"
    - "How did collaboration with these other stakeholders shape the project's direction?"
    - "Were there any other unexpected allies or sources of support that helped move the project forward?"
    - "Have you faced resistance or lack of support from within the project, or from other organisations or individuals?"

Reflections on social prescribing (20 minutes)

After talking about your specific project, now I‘d like to reflect a bit with you on the potential role of social prescribing:

5. **Model identification**:
   - “How do you understand social prescribing?”
   - “Is this a term you would you to describe your work?”
   - “What framework or model does your work align with, if any?”
     - “If no specific model was used, what guided your approach?”
   - “Do you see social prescribing as a useful approach in the contexts you work in, or are there other approaches you think are more fitting?"
6. **Model comparison and appropriateness of social prescribing:**
   - "Which aspects of social prescribing do you think might be especially helpful for refugees?”
   - "In your experience, are there parts of social prescribing that might be less suitable when working with refugee groups? Why do you think that?"
   - "If you could adapt or improve social prescribing models to better fit the needs of refugee groups you work with, what changes would you make?”
7. **Lessons learned and looking forward:**
   - “If you could redesign a social project entirely based on your experiences, what would you do differently?”
   - “Looking ahead, what do you see as the most promising future directions for social projects addressing refugees’ needs?

Wrap Up (5 minutes)

8. **Reflection:**
   - “Based on your experiences, what do you think are the most important factors behind impactful project work with refugee populations?”
   - “Is there anything else you’d like to share about your work or views that we haven’t covered?”
