# Supplementary material for: Health system lessons from community practice: a qualitative study rethinking the role of social prescribing for refugee populations
Source: Front Public Health. 2026 Jan 26;13:1739953. doi: 10.3389/fpubh.2025.1739953 (PMC12883642; doi:10.3389/fpubh.2025.1739953)
Supplement: Supplementary file 4 [file Data_Sheet_4.pdf]

Code System

Code System	Frequency
Code System	3428
Contexts	0
State actors	0
Social workers	25
Schools	40
GPs	63
Participant contexts	0
Populations involved	45
Children	61
Family	65
Men	28
Women	68
Project contexts	0
Financing	83
Project structures	40
Project purpose or objective	29
Need for project	34
Project background	63
Interviewee contexts	0

Personal motivation	19
Decision-making and values	50
Work experience	51
Migration history	6
Education	23
Model & Methodology	0
Project mechanisms	0
Language barrier solutions	59
Cultural barrier solutions	70
Mental health and trauma solutions	81
Trauma-informed practice	68
Enabling characteristics and benefits	34
Activity tailouring	21
Delivery model	124
Project design	0
Recruitment and referral	116
Methodology for project	15
Activities delivered	148
Project delivery	0
Stakeholders involved	24
Volunteers	63
Partnerships in delivery	97

Staffing in delivery	77
Resources	0
Own learning	0
Priorities if starting new project	36
Lessons learned from own project	163
Learning from work	55
Material	0
Environment	12
Donations	22
Stakeholders	0
Stakeholder strategies	79
Supportive relationships	37
Challenges	0
Staff challenges	0
Staff burnout and secondary trauma	43
Working conditions	71
Emotional response	6
Participant challenges	0
Transport	18
Stressors for refugees	26
Physical health	19
Mental health and traumatic experience	73

Cultural differences	46
Language barriers	63
Barrier characteristics	49
Environment challenges	0
Local communities	26
Challenging relationships	60
Rural location	20
Structural challenges	0
Narrative critique	41
Media	10
Politics and system critique	60
Refugee allowances	29
Hostile environment	4
Outcomes	0
Assessing evaluation	0
Role of funders	9
Reflection on evaluation	65
Structuring evaluation	0
Informal	15
Formal/validated scales	23
Monitoring impact	0
Project impact or changes brought about	73

Individual journey	59
Reflections on social prescribing	0
Defining and contextualising	0
Social prescribing definition	27
Familiarity with social prescribing	28
Alignment with project work	45
Mapping service gaps and need for change	0
SP aspects - recommendations for improvement	54
SP aspects - what needs to change	66
Assessing relevance	0
SP aspects - less fitting	39
SP aspects - especially fitting	18
SP evaluation - SP as useful	44
SP evaluation - other approaches as useful	5
