# Supplementary material for: Health system lessons from community practice: a qualitative study rethinking the role of social prescribing for refugee populations
Source: Front Public Health. 2026 Jan 26;13:1739953. doi: 10.3389/fpubh.2025.1739953 (PMC12883642; doi:10.3389/fpubh.2025.1739953)
Supplement: Supplementary file 5 [file Data_Sheet_5.PDF]

24/11/2025

Quote examples from all code categories

Table of contents

1. Social workers ... 5
2. Schools ... 6
3. GPs ... 8
4. Populations involved ... 9
5. Children ... 11
6. Family ... 13
7. Men ... 14
8. Women ... 16
9. Financing ... 18
10. Project structures ... 20
11. Project purpose or objective ... 21
12. Need for project ... 23
13. Project background ... 25
14. Personal motivation ... 26
15. Decision-making and values ... 28
16. Work experience ... 29
17. Migration history ... 31
18. Education ... 31
19. Language barrier solutions ... 33
20. Cultural barrier solutions ... 35
21. Mental health and trauma solutions ... 37
22. Trauma-informed practice ... 39
23. Enabling characteristics and benefits ... 41
24. Activity tailouring ... 42
25. Delivery model ... 44
26. Recruitment and referral ... 46
27. Methodology for project ... 48
28. Activities delivered ... 50
29. Stakeholders involved ... 52
30. Volunteers ... 53
31. Partnerships in delivery ... 55
32. Staffing in delivery ... 57
33. Priorities if starting new project ... 58
34. Lessons learned from own project ... 60

35. Learning from work 62
36. Environment 64
37. Donations 65
38. Stakeholder strategies 66
39. Supportive relationships 68
40. Staff burnout and secondary trauma 69
41. Working conditions 71
42. Emotional response 72
43. Transport 74
44. Stressors for refugees 75
45. Physical health 76
46. Mental health and traumatic experience 78
47. Cultural differences 79
48. Language barriers 81
49. Barrier characteristics 83
50. Local communities 84
51. Challenging relationships 87
52. Rural location 88
53. Narrative critique 90
54. Media 92
55. Politics and system critique 93
56. Refugee allowances 96
57. Hostile environment 98
58. Role of funders 99
59. Reflection on evaluation 100
60. Informal 102
61. Formal/validated scales 104
62. Project impact or changes brought about 105
63. Individual journey 106
64. Social prescribing definition 110
65. Familiarity with social prescribing 112
66. Alignment with project work 113
67. SP aspects - recommendations for improvement 115
68. SP aspects - what needs to change 118
69. SP aspects - less fitting 120
70. SP aspects - especially fitting 122

71. SP evaluation - SP as useful 123
72. SP evaluation - other approaches as useful 125

1. Social workers

1.

"I mean, social prescribing is like full on caseworker role or you just have to like, get deep into social welfare, racism or all sorts of complexities come at you from left, right and centre. So I think there's a gap between what the NHS thinks social prescribing is and what the reality of this, of it actually is."

Code: Social workers
P7, Social prescriber

2.

"So yeah raising some of the issues, I personally had the meeting with the housing, the head of the housing department here and raised some of the issue of our community members, and he was very happy to meet me. And, uh, so, uh, if there is something, definitely we are contacting other and going to represent them, but uh, in terms of, uh, referring. So most of them, they're referring people, like I said this morning, uh, the social service, uh, referred the family to us and they're saying they're closing their case. And uh, if it's an issue that the (charity), uh, can help and deal with it."

Code: Social workers
P5, Charity director

3.

"Depending on the individual, there are some who do the bare minimum. Um, and there are others who will go above and beyond for every single child, because they want what's best for them. They are all overloaded and overwhelmed. Um, so I think a lot of them get jaded, even if they've gone in with the best intentions. And the overwhelm also means that our kids, there's a specific date before they're, there's a specific number of months before their 18th birthday that they're supposed to be allocated their leaving care workers so they can build that relationship, but it doesn't happen because there aren't enough workers. So some of them are meeting the leading care worker weeks before their 18th birthday. So it's something they really don't know very well. Um, but if you get a good one of those you're pretty much all right. We work fairly closely with them. And that's kind of the level of our support post 18 is we'll get advice to leaving in care because there are too many effectively too many looked after children between the ages of 18 and 25 for us to be personally involved in all of their cases. So, but we will become personally involved, if they ask us to. We just won't go out looking for opportunities to get involved. Um, and the leaving care workers have basically fed back that in terms of education, the main issue is that they've been forced to do ESOL and they've got no qualifications, because of that. On the other hand, they do then have access to adult education, which I think it's probably, I mean, I don't know enough to say for sure that I would think it's a better offer than 16 to 18. So. Yeah."

Code: Social workers
P16, Quality Assurance Teacher for post-16 education

4.

"Okay, well, I'm a registered social worker still. Um, and that's an important part of my professional identity. And I became-, I joined social work and qualified, well I joined social work in 1971 and qualified in 75 and have worked in local authority, um, most of my, um, practice career. Um, so doing statutory social work, which in those days was generic, it was everything. And so I see social work as a holistic generic. It's, um, intervening whether it's at the individual, group or community level. And that is my belief in social work. You know, however much that's changed over the years, um, which is why I remain registered."

Code: Social workers
P12, Founder and research coordinator

5.

Person 2: "And I'm also, uh, the supervisor for the international students that they have to have a social worker to support their learning. And so I can tell you that a Swiss student who was here on a four month, might be a five month placement proudly told me that she'd managed to get 12 people housed by just never stopping ringing private sector landlords and pushing them and so forth. Um, and, um and another student that, uh, French student said that she, she would be leaving in, she'd been working with a woman and she was going to be finishing her placement again, it was about five months placement, and that she had been told that she should close the case before she goes.

Person 1: Right.

Person 2: And she felt that she really didn't want to. And it was a really interesting moment because that was right in terms of the management. Of the case, you know, because that person had was capable then of getting whatever help she needed, when she needed it. #00:30:27-5#

Person 1: Yeah.

Person 2: Uh and it was the, it was the student not wanting to let go. Um, but you know, she felt kind of attached to this case. Anything other than, "I'm leaving" felt like a rejection, which was a wonderful learning moment for her."

Code: Social workers
P12, Founder and research coordinator

2. Schools

1.

"So first priority is to find them a school, a mainstream school where GCSEs will be being taught and in year 11, no one wants to take any new students, because a student not being entered for exams or being entered for exams and failing them is, obviously has a negative impact on the school statistics and the school's Ofsted and the school's reputation and everything. So schools don't want to take these young people. The (name) school being statutory, we have the right to direct schools, um, to take our young people, um, to avoid discrimination. However, while we still can direct academies, up until this year, they haven't had the same responsibility as local authority maintained schools to respond immediately to our direction. We, in fact have to take it effectively to the DfE, and get the DfE to, on our behalf, direct the academies, which usually means quite a long period out of education for that young person, while we are going through the direction process to make sure that they have a school place. So then they'll get a school place, they'll, we'll get them their uniform, they'll have a look around, they'll go to school."

Code: Schools
P16, Quality Assurance Teacher for post-16 education

2.

"You just, the child is there and they are in lessons and they're in lessons not understanding things, and teachers have to manage and they have to manage and that's it. Um, so that's where they come in, and because they're in care, they'll have PEPs. So personal education plans once every term. I don't do year 11, so I can't tell you so much what they look like in

year 11, but I would imagine a lot of it is college planning. And I kind of missed this out when I was talking about the direction process. So the direction is kind of the stick, the carrot is, we the (name) school gives £6,000 to every school that accepts one of our unaccompanied asylum-seeking children because of the resistance, we kind of have to, what they say effectively is we don't have any of the resources that we need to effectively educate this child. So give us them and we do. And that's the money that paid for the centre that I'm describing. Unfortunately, other schools will take that money, and often they'll use it to get a teaching assistant for a very short space of time. And it's completely ineffective."

Code: Schools
P16, Quality Assurance Teacher for post-16 education

3.

"And in fact, Ukrainians have been very interesting because they brought schools into our network from wealthier white areas. If I'm honest, that weren't wouldn't have really been interested in that as much before, because there was lots of the Syrian Afghan families get placed more in central (city), um, when they move on from their hotel accommodation. Um, and the schools there are quite multicultural anyway. When I say multicultural Pakistani, lots of them, but, but as well multicultural. Yeah. Um, whereas others go just um, on the outskirts of (city) has sort of maybe 80% white. And they had a few Ukrainians because the hosting scheme was different for Ukrainians. They lived on the whole with white, wealthier families. You had space for them. Um, but that and that's been really interesting and really brought a different demographic of schools into the network."

Code: Schools
P19, Schools coordinator

4.

"As you were talking, I was like, oh schools are a bit like social prescribers. So, I mean, got a school that became a school of sanctuary a few years ago, I'm actually now a governor for. They were the ones with the Afghan families, actually. They have, um, a hub, at the hub, which is separate sort of building next to the school, but run, run by the school. And, um, they have a women's English group. They have a slow cooker group. Um, they have, like a trip to the park to do laps around the park group. And actually they find lots of the mums who are too scared to go out, particularly when the Afghan mums were there a few years ago who were in the hotel and just the school gate was their safe place. That was it. Um, they used to take them to the park to just walk around and introduce them to the park, which gave them the confidence to do it. But it was also physical exercise but also help build community. Um, because they, they were also saying it is a very, very deprived areas, one of the most like high level, highest levels of unemployment and lowest life expectancy areas in the whole country. There's no, um, there's no community cafe. So they were saying, you know, school parents drop off kids and then want to talk, but there's no space to build those relationships. So the school had the hub where you can drop in for coffee afterwards and you can you can meet other people. And that for some parents, it's been a lifeline of meeting other people and then doing, doing activities. So yeah, I guess that's some kind of informal, it's social prescribing in a way, because it has really had a massive impact on because we, I was talking about whether that, that has an impact on pupil outcomes, which is the ultimate goal and actually does because it breaks the barriers down between them. Then they might be more likely to go into a reading session in school, you know, where parents are invited in because they've built confidence in the hub, which then has the impact on whether they might read to the child at home, and that improves outcomes very much."

Code: Schools
P19, Schools coordinator

5.

"Yeah, I'd say funding is a big issue in schools. But and that that's been the brand, but yeah, many of our schools who particularly who are schools of sanctuary um will have clothing banks, food banks. Another school who became a school of sanctuary this year, they have a community little centre next to the school where they are open every day for parents to drop in. And when we interviewed parents on the appraisal day, they were talking about what a lifeline that had been. Um, just an informal setting to talk about anxiety. And they run parenting courses. And I feel like it's rolling out this kind of extra support into schools that aren't quite so have such a needy, that sounds, I don't mean to use that word derogatorily, but population because so for my children's school just up the road, yeah, we aren't central (city) um, and I always think, oh, they do nothing. They don't have community things with the parents and they don't, but we don't really need it as much. Um, but they have started doing like a drop-in thing for anybody that wants to for a session, which I think is quite new and ground-breaking for, for them. But that's not ground-breaking for some of the, you know, inner city schools, um, where schools are a lifeline. So I think that's why, that's what makes me so passionate about schools. I feel like schools are filling the gaps. Yeah, that community has been lost. And where, you know, small businesses, small cafes, perhaps have struggled in Covid and disappeared and community centres have disappeared… um, you know, the school where I am governor, they open their kitchens every holiday to let families who are living in hotels, use their cooking facilities because they can't cook. And three out of five of the weeks that are the summer holidays, they're running, um, holiday, sort of free holiday clubs. So in the most deprived area, taking them on beach trips and the whole families too, they're taking the families to like, Whitby, um, which is amazing. They've applied for a grant to do it, and that's how they're doing it. But it's still teachers getting holidays and getting paid a bit. But yeah. #00:49:28-0#"

Code: Schools
P19, Schools coordinator

3. GPs

1.

"We all agree that the main outcome of this job is making people feel seen, because when they go to the GP, they're looking for a space to talk about the most vulnerable side of their lives, which is their health, their mental health, their feelings, their emotions, what's going on for them. And the, there's no time for, the GP has ten minutes they give you okay, but and then they're left feeling like, okay, not even the space where I am being, if I am supposed to get care from them, not even that place wants me. So then now having a role like this in a setting like that, because primary care is very communal. It's a very, it's a community space, one of the few community spaces. So to me, it's so important to have something in sort of embedded in that system where people feel like they have that space to talk about what's underlying that stress and that anxiety that they come to the GP talking about."

Code: GPs
P7, Social prescriber

2.

"Um, I think I think there are many I think there are many aspects, as I've just said, that could work well. I think the referral process would need to be much less formalised because GPs, for example, quite often don't recognise what's going on for the person. Um, and often what's brought to light are the medical issues. They perhaps don't always see the social issues. I mean, that could be said for a lot, you know, for the population they work with generally, but I think they probably, well, I know people who are GPs who worry a lot when suddenly, um, a hotel, an asylum hotel is opened up on their patch and suddenly there's an influx of 250 young men between the ages of 18 and 25. Suddenly their case numbers, you

know, their practice list goes up and they've got no idea how to work with people. They don't have language to understand. They can do basic screenings, but they often don't don't know what services are available, don't know how to work with interpreters in a genuine and helpful way."

Code: GPs
P4, Senior researcher

3.

"I mean, every child has a GP, and any child who is not getting out of bed is going to have a GP appointment made for them to talk about their mood. Um, but if they're not getting out of bed it's whether they'll make it to that appointment. Even though a staff member will, obviously there's a GP appointment like that will take them. It's not always guaranteed that they will get out of bed when the staff member comes to get them."

Code: GPs
P16, Quality Assurance Teacher for post-16 education

4.

"So for example, even accessing social prescribing, if someone's not registered with a GP, then how do you access the social prescriber? But also the kind of longer term barriers in accessing support and appointments and interpreters? Um, so exclusion, those kinds of things that are across the health system. Um, yeah, I think probably the biggest challenge really is, is the circumstances that people are in. So you can only have so much, you can only support someone so much without changing the circumstances that people are in. And I think to have a, a significant impact on someone's health and wellbeing, they need to not be within that system. And that's often the issue that we have in delivering our services is actually they just need to be moved out of (town), for example. And that's causing a lot of those issues. So, um, it's yeah, it's working within the context of the kind of wider structure of problems and the complexities of the asylum system. #00:43:31-7#"

Code: GPs
P21, Advocacy manager and primary care lead

5.

"I think that's fair. I think that, schools are a safe place for so many families. And actually the health care system often isn't. People don't understand. It's so different. You know, they have a headache. They end up going to A&E because they don't really understand the GP set up, but they don't have that, and they've got, you know, having lived abroad myself, you don't have a GP, you go straight to the hospital if you need antibiotics. Um and just and, and as we all know, it's a nightmare getting a GP. You spend hours on the phone, you'll get asked if you want a ten-minute phone consultation. The next step, you know, it's, it's confusing. Um, and it's a language issue as well. Whereas in school they often have many people that speak different, you know, they have teaching assistants that might speak the different languages. They usually have a member of staff that can translate. Um, so that's, that's a massive factor. Um, but I think, I think it's the trusted person thing, and that's where schools are really valuable."

Code: GPs
P19, Schools coordinator

4. Populations involved

1.

"Um, and as part of that remit, we work with unaccompanied asylum-seeking children. The city in which I work has, I don't want to say, the largest number in the UK of any local authority, but it's definitely up there in the top five, um, unaccompanied, uh, asylum-seeking

children. And obviously because they are children and they are unaccompanied, they immediately come into local authority care the second they arrive in (city), which means we're responsible for ensuring that they have an education and for removing barriers to that education, which means any kind of physical or mental health problem that is going to serve as a barrier to education is, uh, in our remit. Um, and I work with post 16 as well. And I would say that the majority of young people who arrive are around 15, 16. So they'll be in year 11 or moving on into year 12."

Code: Populations involved
P16, Quality Assurance Teacher for post-16 education

2.

"In terms of where people come from. Yeah. Um, yeah. I mean, (city), I think we have about 1300 asylum seekers currently in sort of contingency accommodation. But we were the first city to have like Syrian refugees in 2014. Rohingya refugees in 2011. Yeah. Um, a lot of Afghans got sent here to, um, and Ukrainians actually. And in fact, Ukrainians have been very interesting because they brought schools into our network from wealthier white areas. If I'm honest, that weren't wouldn't have really been interested in that as much before, because there was lots of the Syrian Afghan families get placed more in central (city), um, when they move on from their hotel accommodation. Um, and the schools there are quite multicultural anyway. When I say multicultural Pakistani, lots of them, but, but as well multicultural. Yeah. Um, whereas others go just um, on the outskirts of (city) has sort of maybe 80% white. And they had a few Ukrainians because the hosting scheme was different for Ukrainians. They lived on the whole with white, wealthier families. You had space for them. Um, but that and that's been really interesting and really brought a different demographic of schools into the network"

Code: Populations involved
P19, Schools coordinator

3.

"Definitely. Um, but also um, culturally, yeah, there's been there's been some barriers because we have Russian heritage Ukrainian families, but we also have Polish heritage Ukrainian families. It's such a large country, you know like we have our different dialects within the UK. There's so many in Ukraine, you know, I think you can fit the UK into Ukraine like 30 times or something ridiculous like that. It's it's such a big country with so many different provinces and so many different, you know, kind of, um, heritage, dual heritage. And there are a lot of Russian heritage Ukrainians because of the border. Um, and that obviously creates, um, you know, a kind of, uh, they're all against what happened to them in the war. You know, they are Ukrainian, but they have got a Russian heritage, so there's that kind of, yeah, you have to be really mindful with, like how you speak to them about their experience, war, and where their loyalties lie."

Code: Populations involved
P2, Youth service manager

4.

"Um, and so yeah, that's like you'll find in that pockets of the Native American population, um, but more so, I'd say like we had a lot of, um, Bengali people, um, lots of people from different parts of sub-Saharan sub-Saharan Africa, um, probably mostly like pockets of West Africa and East Africa, lots of, um, Caribbean people from the Caribbean. Um. But then in terms of like people seeking asylum, um, people from Somalia, Pakistan, um, different countries from the Middle East. Um. It it's really hard to say because honestly, we will see so many different people, um, and like I said that I would often know who's coming through my door like five minutes before. (Person 1: Yeah). And had to be like, oh God, I need to get an interpreter like this and (Person 1: Yeah) just be like, okay let's let's see who we are. Um, but

it's quite hard to, like, keep tabs on it all. But it's a very diverse population in the area, um, just generally."

Code: Populations involved
P1, Social Prescriber

5.

"And I mean, but despite our name, we don't just work with Afghans, we support refugees, from all over the world, like Ukrainians, Syrians, Iranians, Somalis. Yeah, yeah. Our our, um, organisation is a very diverse place and we appreciate that. It's like a, you know, kind of like a cultural, like hub."

Code: Populations involved
P8, Integration manager

5. Children

1.

"Yeah, yeah. Um, so some of the barriers I have are the language, but as I say, people are really trying very hard to overcome that. Yeah. Um, together we can. The other barrier, I have two barriers I can think of are, um, parenting support. So if I want to refer someone for English classes, a woman who's parenting alone. Um, I haven't had met a dad who's parenting alone, but if I did. There isn't an English class system available where you can bring a baby or bring a child. So that is, that's a barrier. So that means we have to look at alternative things like a mother baby group or a, you know a, a women's chat group. Um but a lot of times centres like this insurance wouldn't cover having, you know, a baby or a toddler in the, in the area. Um, so that is a barrier. Um, you know, I've, I've met some very good people who are robust in trying to accommodate, you know, they say, well, if the baby stays in the stroller or the baby stays quiet, we can, you know, let her come and she can be there and we won't have anyone make a fuss, you know, things like that. Um, so that, that is a, that is a difficulty. And then, you know, if you don't have English and you're trying to join a local mother baby grip. You've got the issue where, you know, people have come together. I don't want to use the word cliques, but they've come as a group unit and it's hard to break into us. And it's hard to, you know, keep on smiling when you haven't got the language. So, so that that that is an issue. Um, but in fairness, the people do persevere with it and they're willing to, you know, take one experience at that, another experience of something else, another experience or something else."

Code: Children
P22, Social prescriber

2.

"Uh, and then the, the young, uh, the young generation, which is, oh so vulnerable, uh, and we have to get them involved as some, uh, uh, meaningful activities. Otherwise they're going to be targeted in the streets, uh, of, uh, by, uh, inappropriate people, to be honest, either religiously or either, uh, other, other, uh, illegal activities. So, uh, it's so vulnerable to that as well. And uh, families, uh, which they are not familiar with the state of the, the, uh, this country, uh, not being integrated into country also cannot have, uh, full control of their children as well otherwise. So they are the other targeted, which is the, uh, police on the council and, and other and, uh, by record, the Afghan community members within the police, uh, uh, a very, very low in illegal activities within the UK and, uh, the activities that being illegal or violence, the Afghan community are less, less involved even not coming in numbers as well. So this all because of the, the community such as us to working with our young generation and put them in meaningful activities, put them in sport activities, uh, providing all the uh, all the information about the, uh, legal and other, other stuff of the country, get,

uh, involving their parents, how to, to, uh, control their children, how to behave with the children. I have to control their family members with them. It doesn't mean that we are interfering in their life, but we are just providing the information of the country or updating them with all other stuff. Or, uh, I mean, to be honest, the, the family issues of the Afghan community is another big, big target of the community as well that there's, uh, we can admit that there's a family, uh, issue, uh, is appearing within the family as well, and that we are referring to the women's group or speaking to the family and speaking to their husband or wives. And, uh, I mean, there's a lot of aspect of that coming to this country and, and being disagreement between the families. So this is another issue that been, uh, targeted by the Afghan community, not this thing to happen and, uh, get to the stage of violence. So sort of things within the family."

Code: Children
P5, Charity director

3.

"We came across a single mom, you know, with, with two young children who had not stepped outside her door for 48 hours, uh, because, because of fear, because of this, this, this virus that she was hearing about, that was, you know, that was, you know, that was killing people. And the food banks were, food banks were leaving bags of food at her door and, you know, and running away because that's what we all did at that time, you know. Uh, however, the food bank never thought to check whether she had, they just presumed that she had a, you know, access to hot to hot water. Uh, or and she had a tin opener. So all the foods that she was, that she was being left with was tinned food and dried food, she didn't have a, she didn't have a kettle, and she didn't even have a tin opener. So that's one of our workers, you know, using Google translate, you know, using Google Translate, found out that she hadn't eaten for two days, the children hadn’t eaten for two days because she was too scared to go out. She didn't have any access to information about, about Covid and what was, what was going on, what was happening. I mean, for a couple of days. One of our workers went right down to the local shop and got a tin opener for £0.50 and, uh, you know, and you know, and, you know, some pots and pans and she was able to able to eat. So that was, you know, that was that was the kind of, you know, we were able to be really flexible and responsive and we were able to do that, you know, just really, really, really quickly in a way, you know, that statutory agencies, you know, just, just wouldn't, wouldn’t be able to do"

Code: Children
P11, Founder and CEO

4.

"Two of the people turned out to have written about a time when they had to tell somebody that their child had died. And that was really, I mean, I had no idea what to do. And, and one of them, they both were one of them actually broke down and cried. And the other one was obviously very near to it. And I thought, oh, my God, you know, this is a disaster. What's going to happen? The group, nobody's going to want to come back to the group. Whereas it turned out one of them went out of the room and somebody else went out and sort of comforted them, and they came back. And it was okay."

Code: Children
P10, Writing project coordinator

5.

"Um, break that down and, and, and, and it also explained to the women themselves, you know or not explain but discuss, you know, have you heard of, of these things that happen when you've had traumatic experiences? And it's really good for the women, especially those with kids, to for them to understand, or them to have the option of looking at a model of what might be happening in their brain of that no, they're not a bad person shouting at their

kids. It's because you know, so, so I think the, the value of for me of studying while I'm doing the practice because I benefit so much from the research in the study, but also being able to share that with the women. So they also understand what might be going on or can, can reflect on if they want to, to, to, to to choose a way of looking at it from what I've been learning about. So that sort of sharing of, of that knowledge together."

Code: Children
P9, Drama programme founder and producer/director

6. Family

1.

"(City) has a significant population of asylum seekers and refugees. When they arrive in the UK, folk are often traumatised and isolated. There is this harmful right-wing narrative that people seeking sanctuary are living a life of luxury in hotels funded by tax payers. In reality, families are often confined to one room, do not have access to any food that they like/are familiar with, and have to subsist on £7 per week. Alternatively they are in shared accommodation e.g. with single mothers who do not speak their language."

Code: Family
P23, Volunteer

2.

"All the time with social services. I had a family from Nigeria terrified. They were terrified. Um, here years and finally we were doing their applications because they finally met the rules. And they had four children. Five children, and three of them were very small. And I referred them to social services under section 17 because they were in a one bedroom, like not even one bedroom, a studio flat cramped. So when I submitted the application then um, I was able to um refer them to social services because they, they only will assess if there's a legal representative involved. So they're always um doing this, Section 17 refers to social services once we started the case. So I did that and they got an interview with the social worker. And they separated the parents from the children to interview them. That's unlawful. That's just not okay. And, but obviously my client, she didn't know that she could say no. She thought that they were taking the children away because they were documented. And that was bad. And because she's doing something bad, they're taking the children away from her. So she and her husband stayed for, like, an hour on their own, waiting for the children to come back. Nobody told them if the children were coming back, why they had been taken away. And they were interviewing the children. Um, I don't know what for. Just to find out whether they had support systems somewhere else so they could send them away. But they were separated. And then, uh, yeah. Days after I saw that client again, she was completely traumatized. She thought that she'd never see her children again. Yeah, things like that."

Code: Family
P7, Social prescriber

3.

"Enjoyable something I've personally when I support a family which otherwise been in big trouble and, uh, I'm coming home, I'm so relaxed and just feel that I help someone, um, uh, which otherwise going to be in so difficulty. Uh, I saw people were on the street with their two young children, and I was sitting in an office until, uh, he was given an emergency accommodation, and I didn't go home because I said, if I go home, what will happen with this family, just on the street? So when I heard that, the council called me and I said, we're going to send them to emergency accommodation, and they are so relaxed. And I slept very well.

And I said I, someone received support from me, so I was, I was feeling so proud of myself that I support someone."

Code: Family
P5, Charity director

4.

"One of them was housing, then supporting the families to their home. So when it originally started, it was the responsibility of the council to make sure that anyone coming in through (area name), and it was predominantly (city) off the ferry, would be picked up by a host family and taken. And (area name) made sure that those connections happened at the port. So because our charity is based in (city), we were commissioned to do that work from the very beginning, as soon as the refugees started coming in. So we'd meet the families as soon as they come off the ferry, connect them with their host and then they'd go off to the homes that they were staying at. None of it was really that local, but obviously the ones that remained in (area name), they were the ones that retained with the council. And then once that started happening the flow was happening they needed to be supported while they were here."

Code: Family
P2, Youth service manager

5.

"If you are a family whether that is a dad with a child, or a mom with children or a couple with children, if there is not a temporary housing for them, they then put them up in things like travel lodges. And the problem for those families is that, uh, just working with one family. And just to give you an example. So mom, dad and the oldest daughter came to talk to us. They are in a, um, travel lodge. Quite on the edges of the of (town) if you like. It's quite a peninsula But because it's so close to the school holidays. Their children are all in different schools and one of the children is way down south. That family spends £84 a week on travel."

Code: Family
P18, CEO

7. Men

1.

"You know, and I'm just thinking of a client in this recently who was a man, I, I and I say that because I have 25% men and the rest are women, that the, the gender breakdown. Um, so but he's here earning or sorry, getting €38 a week. Um, feeling responsible for, you know, his mom and six siblings, um, in his country of origin. That's a that's a lot of stress. It's a lot of pressure."

Code: Men
P22, Social prescriber

2.

"And that's where a lot of the stumbling blocks are in social prescribing is in these circumstances like the Arab scheme, I know there's a massive army camp that's been turned over to refugees from Afghanistan who come under the Arab scheme, and the GPs have no idea. The numbers of them are so vast that they don't know, you know, how to work with those people. And the Arab scheme does provide somebody who's sort of a caseworker or a key worker, but that person themselves is totally overwhelmed by having to deal with not only the 250 men who've come through that scheme, but their families, because the whole family comes under that scheme. Totally overwhelmed."

Code: Men
P4, Senior researcher

3.

"I was reading, let's say (region), for example, because (region), there were people, um, because there is a lot of single males there. Um, you know, they were saying, our kids aren't safe and blah, blah blah and all the rest of it."

Code: Men
P18, CEO

4.

"For social prescribing huge, huge mix of genders, ages, everything. And that's one of the great things I think about social prescribing, unlike in my other work as a therapist, where, you know, you unfortunately do see less men. (Person 1: Yeah) Um probably see a smaller range of ages as well, because it's still not like, as socially acceptable to go to a therapist, whereas for a social prescriber, um, because it's in the GP surgery, I think, you really do get to see everyone, um, and a lot of clients I was working with, there's no way they would see a therapist, but they would come see me and they would come and chat to me for a long time."

Code: Men
P1, Social Prescriber

5.

"So I worked with a young lad, um, he had no family here. He was living in a home with, um, three other men that didn't speak his language. He really, really wanted to work, but he had to wait for his, um, his immigration status to change over, essentially. Um, and he, um, we tried to get him access to these services. He didn't know how to get there, so I ended up having, um., say having like it's a bad thing, but we got the bus together, and I took him because I'm from the town where this service is. So I, you know, showed him the route together. We walked over to the service, um, he we went in together. He had a conversation with, um, a volunteer there in his native tongue, which was I personally find it really interesting when people speak in their native tongue. And even though I don't know what's going on, I just kind of look at them in awe. Um, from there, he just owns, you know, a WhatsApp group. So we had a support worker he can contact any time of day. He got a free SIM card. Um, he then got access to education and college, too. Yeah. Um, so it can work quite well. It's just if they are unaware of the service, it works a lot better. If they're aware it's about trying to find out where those gaps are and what else they can sort of slot into, to a point where it's not going to overwhelm them and also isolate them further."

Code: Men
P17, Social prescriber

6.

Person 2: "Uh, there was a, uh, a men's group that sprung up. Uh, a swimming group. And the first that came, just a couple of people within the local, a couple of men, a couple of guys within the local community who were struggling bit with mental health. And they get into this whole, you know, whole sort of sort of cold swimming, swimming kind of kind of thing. And a Sunday morning at 9:30 on a Sunday morning, you know, see anything up to about 40 men, 50 or 40 men. All all swimming.

Person 1: Yeah.

Person 2: But they did more than swimming. You know, they're also supporting each other and they're talking to each other and they're developing relationships and, you know, and, uh, and they're signposting each other to other, other supports that, uh, you know, that costs nothing. It costs absolutely nothing. It's, you know, it's a grassroots project, you know, (charity) was very much a grass, a grassroots project."

Code: Men

P11, Founder and CEO

7.

Person 2: "Because I can hear (staff member) in in my head, yeah, that's how it would work with the family or with. Yeah. If it's a single young male, they will, they will certainly have to be street homeless for a period. So when then, when, when you know, that conversation is, it's not just about, you know, what do you want, what you hope you know, let's support you in doing that. It's like, this is the situation as it is here now in (city). You knew when you have to leave your accommodation there will be nothing else and we can show you where the safe places are. We can give you a sleeping bag. We can put you in touch with the Rough Sleepers team. Uh, but unless you have been recorded as being out on the street for three nights, you won't be treated as homeless. It's that level of information that people need, because, uh, the numbers, I mean, with, you know, I know that the (charity) team is thrilled to have, um, so many people being granted, but it's, it's, there's no resource for them. There's nothing, you know, so people with additional…. What people are largely looking for isn't, you know, that kind of soft, gentle, you know, what do you hope you know, do you want to be a chef or, or you, you know, you want to get to university or that it's like, okay, have you got do you know anybody in the city that could put you up? And this is the way the system work, and we'll help you with it as much as we can."

Code: Men
P12, Founder and research coordinator

8.

"We have a man who came to the group from a country in sub-Saharan Africa, a francophone country. So he arrived in this country with literally not a word of English and no money, and sort of met somebody at the mosque and slept on his sofa for a while, and eventually was referred to (charity). A very bright man. He'd been a teacher, but a science teacher in his own country and, um, absolutely passionate about it and no idea about literature and no idea about creative writing. He'd never done any creative writing, even at school. And so the first piece of writing I asked him to do, I said, can you describe your ideal day? And his ideal day consisted of, um, marking homework and planning the next day's lessons, and it was written in a very plain style. And so time went on, and a few weeks later we did a workshop with, I think about poetry, and I think the, the model that we use was Kipling's If, something like that, which was great, "If you could do, if you can do that", it's very easy to kind of grasp and follow. And so he wrote a poem in response to that, and he was like, "Oh, is that me? Does that come out of me? Is that in there somewhere?" Yeah, it was just amazing. And he then became this extraordinary poet. And it was like, it was like watching him growing into a new being. It was like midwifing a whole new side of his personality into being. It was such a privilege to, to help that happen."

Code: Men
P10, Writing project coordinator

8. Women

1.

"Initially single mums and young men, so we made it so children could come to the sessions. I found that increased contact outside of sessions with men particularly helped them to access the sessions. The men who attend our sessions are often extremely traumatised and more likely to have travelled by boat, experiencing unimaginable trauma on their journey to the UK."

Code: Women
P23, Volunteer

2.

"Um, so I met a woman from the (city) or, sorry, from the Democratic Republic of Congo. So I did my master's, um, um, rape services in, um, DRC. Um, compared to the (city) Rape Crisis Centre. That was that was my thesis. Um, so, um, I suppose I, I have an interest in, in, um, in that area. So I, uh, met a client, um, she was living in a she's living in a, in an IPAS centre. Um, it's a it's a comfortable, um, well-run centre. Uh, that was a hotel. Um, but clients cannot cook. So what they do, what they're, what's happening is they're, they're given a voucher. They buy pre-cooked or ready meals, um, reheat them and, uh, microwaves. That's all that's available at the moment. Um, she has eight children. Some are adults, some aren't. Um, and she was, um, captured, taken to the bush, um, you know, gang raped, repeatedly raped for a long time, and then eventually, you know, got away. So I met her at her time where she wanted to become. Her English was, um, little, and she wanted to become involved in, um, the community, but she didn't really know what that looked like. Um, so she was, I have to say, she was a little different in that she had a lot of family support. I don't often see that. Um, so she, um. Yeah, I think that family support was, was quite significant. She has a partner and she's very well supported. They were all separated, all got together again, which is a good outcome in terms of her story. Um, because that doesn't often happen. We have a lot of people who are, um, who have lost family. Yeah. Um, and so we, we met, we talked, she joined the English classes, she joined a women's group, she joined a jewellery making group. And she has then gone off on her own route. So she, she was she had never been to school. So that literacy problem. Um, so she also did a English and basic computer skills course here, and she was thrilled with herself. But in the midst of all of that, we talked about (city) Rape Crisis Centre and getting some support from there. And she said no, but she wanted to have better English. Um, she wants to be able to express herself more from the heart. Um, and that she would consider it later in life so that. Yeah, that was a journey uh for her."

Code: Women
P22, Social prescriber

3.

"Um, and there are people. You know, we're working with the family, and although the lady is disabled, she doesn't want to go out. And so she's actually a refugee. And she doesn't speak a lot of English. But she came over with her brother and he's done everything all day, even the personal care he did for her. So we've managed to get her out of the flat a couple of times just to have a little bit. But he's now married and he's got just a little bit more going on and so we went to visit her in the week and she changed. You know, I've never seen her smile so broadly."

Code: Women
P18, CEO

4.

"So there's no point me, um, referring, you know, um, um, a mother who was recently given birth to a, um, a baby and tots group, a mom and tots group, if she can't speak English and is going to feel more isolated in a crowded room. Um, so it can be a little bit challenging in that sense if they, if they have already accessed and aware of those, um, places in, it's a bit well, where do we go from here?"

Code: Women
P17, Social prescriber

50.

"Um, I would say women who haven't had experience of being listened to or even physically using their voices in the past, for example, women who've grown up in very traditional households where their girlhood was relatively free, and then as soon as they hit puberty or

11, 12, that was it. They were at home just listening to men or older people. And that's and you could, it's a, it's a physical, physiological thing I've noticed and a psychological. It's both. It's like um they don't believe they can speak out. And then that's what's lovely about the drama. It's more like a public speaking thing. You know, you start very slowly and perhaps in a performance. Well, to begin with, the general pattern is that a woman would come on and be holding something on stage or just be on stage, you know? Um, yeah, I'd have to say anything. And the next one, they might say something like, take part in one of the songs we sing or something. And then but I would say several of the women who made most marked what we would call impact on their or, you know, something that's measurable, that they can demonstrate what has happened to them as being part of a drama group, but perhaps wouldn't have happened. You can never guarantee that. But is there's I would say that it's women having confidence to speak out publicly. Um, and, and the experience of being listened to."

Code: Women
P9, Drama programme founder and producer/director

6.

"But I use that quote a lot. That's funny. You use that one. Okay. A man walks across the stage. Yeah, great. He's, he's, he's used to being listened to. A woman that in fact that's, that's good, what happens when a refugee woman walks on the stage? We don't know another world, Mr. Brook. You know, this is, it's not, that's just not the reality that I'm working with here. You know, it's that sense that things, you know, it's like the border. You're a fish in water, you know? Brook is the fish in water on the stage. You know, where actually I'm working. I'm trying to make a space. How do we how do we get that habitat? So that environment that those women are in that will enable them to be able to express themselves, and what does that look like? And I'm not going to tell them, you know, it's just it's going to come out what that looks like."

Code: Women
P9, Drama programme founder and producer/director

9. Financing

1.

"Um, but the wider context is that a lot of organisations are really struggling. Um, funding is reduced in lots of ways, including from trust and foundations. It's sort of universally accepted that Arts Council funding, I know, it hasn't really gone down, it's just that other funding has gone down. So demands on Arts Council has gone up. So it's very, it's, it's a very challenging environment. We also, I guess, benefit from working across different kind of thematics. There's some funders who are a bit more interested in arts, some who are a bit more interested in social change. We also are being funded for a sort of strategic positioning. But also we've been active in taking the work forward in areas like relation to climate. Mental health. Trying to be you know we don't want to be led by funders, but we also have to be aware of what priorities funders are thinking about and if they align with what makes sense for us."

Code: Financing
P6, Producer

2.

"So the woman groups, although I create even, uh, our staff in the other side as well. Uh, recently me and my colleagues had a meeting with woman group leaders and said, because of the funder, uh, going to, uh, ending on the end of this month, uh, before luckily, we get another fund from other. And what are you, we're going to, we're going to give you the option that you are free if you want to go somewhere else, so you can go, because we cannot

give you provide you with the with the salary from the funders. And they said no until you're getting another funding. We're going to work as a volunteer because we build the relationship with these women, which otherwise going to lose all of the, the, these women not coming to the activities. So that's the sort of, uh, dedication from uh, the side of our, our staff as well. And they say if there is a matter of month to two months, we can wait for another fund, but we still want to run these activities because of their relationship with the women's group. And they're so worried if the, uh, they leave the project at this, this stage, the, all the hard work that they have built in the last 3 or 4 years is going to disappear. And the women are also, who's going to be so disappointed. So bridging the relationship is also a big things with the community members."

Code: Financing
P5, Charity director

38.

"And, and we work through local health improvement teams and other partnerships in the local patch. So trying to add value to what they're doing rather than trying to go out and do lots of community based things, which is, what is to some extent, I'm less involved these days directly in working with individual community projects. We don't have the means of doing that, with a few exceptions. Um, and in (city), which is obviously the biggest of the areas, there are three sectoral and that's North East, North West and south health improvement teams currently under enormous financial pressure. We have to make massive cuts this year, which is the reality of the financial year. And we've got, I think, got a 13% cut to make in one year from the entire service, which is huge. So a difficult year."

Code: Financing
P20, Regional manager for health inequities

4.

"But funding's a big challenge for us as it is nationally for City of Sanctuary. Um, and we, you know, we're currently more squeezed than we've ever been. Um, big, big funders, funders from before just haven't had the capacity to fund us again this year. And they've said it's nothing to do with the project. It's just they're oversubscribed and the need is greater, there's more charities going for the same pot. Um, and donor funding has dropped off too. And you see that reflected across all charities like individual giving. That's, I mean, cost of living and all kinds of issues around Um. So. Yeah."

Code: Financing
P19, Schools coordinator

5.

"So. So all the (institution) contracts are from year on year, right? For example, we are now in July. Uh, although they've rolled over, the (institution), rolled over our interpretation contracts. But we haven't had the contract yet because the (institution) has to save loads of money. So they're now saying why are you paying your interpreters for the whole hour you know. And I'm going well first of all they're self-employed. They have to travel to the appointment. They, the actual appointment might be five minutes. However they may have sat there for 20 minutes before the doctor was available, and then they have to drive back. And so, not only that, they're on insurance and everything. So they're now trying to push GP surgeries to only in exceptional cases use face to face and the rest of the time use, uh, telephone. And, um, you only need to pay for the quarter of an hour, and I'm going, we're going to lose all those interpreters because they won't be able to afford to work. So, uh, or the people that don't say, well, then if there is a, if they then are being asked for face to face, they will just let you down, um, because they can't afford not to. Yeah. So we're in negotiations with them."

Code: Financing
P18, CEO

6.

Person 2: "Uh, so it's… the volunteers we've got at the moment are for them, the language cafe is, is the big thing. And that is a huge commitment to every Wednesday. Um, in your own time, and paying the car parking because now we have to pay for car parking and. Actually, I say that as though it's a joke. It's not a joke.

Person 1: It's not a joke because the that's directly out of pocket, isn't it? You know, and it's. Yeah, I, I don't know how much it's going to be, but that is still something that adds up. And I don't know if your Wednesday session, did you say it was an evening or an afternoon?

Person 2: It's, because it's two hours. So what is it? I think it's is it £2? There is one car park behind the church and if you get it earlier you could still go there for free. So I usually try and go there towards.

Person 1: Yeah. Um, sure.

Person 2: But it is something that I am. Since that change came, I think we should really try and get some funds to pay the volunteers something for their, their expenses. Yeah. Um, but all our funds are, um. What is it? It's not designated. It's, um, uh, restricted. Because they're grant funded. Yeah. So, um, that's the tricky thing."

Code: Financing
P13, Charity chair

10. Project structures

1.

"So I work with the health and Well-Being team in a company called the (name) Partnership. There are limited company that host, um, different funds for (organisation). And, uh, you know, they're linked to the (region), um, county council or local authority. Um, so I joined an existing team of three social prescribers who were already supporting the migrant community. Uh, and so then this, um, role was carved out to, to, uh, support the migrant community specifically. Um, so because I work alongside a migrant integration team, um, um, I used their criteria for a while. I can get to that later. And then we expanded it as time went on."

Code: Project structures
P22, Social prescriber

2.

"But, uh, I lead for the health board, (region) Health Board on public mental health and the prevention strands of alcohol and drugs work. I would say the mental health part of that is by far away the biggest proportion of it. One of the specific elements of that side is suicide prevention and self-harm work. So I am the (city) City Suicide Prevention coordinator in my second stint, uh, of that and uh, having spent six years doing that role earlier, and my predecessor took over, and then I took over from her when she retired because nobody else basically wanted the post. So I'm doing that job on top of my day job, if you like, but I'm delighted to be able to do that. So suicide prevention is quite a big part of my work. Um, two small teams that work with me and alcohol and drug health team and a mental health team as well. Uh, but the vast majority works with two tiny teams, is creating partnerships of various kinds with multiple partners. Voluntary sector has always been an important part of that. But many other partners as well, police, close colleagues were on suicide prevention and city council colleagues of various kinds, um, slightly unusual partners like roads, engineering companies from point of view, things like bridge safety and suicide prevention

work, um, along, along this whole list. But, uh, and as you might imagine, also mental health and allied charities for a big part of the work we do, uh, community groups of various kinds, youth projects, schools."

Code: Project structures
P20, Regional manager for health inequities

3.

"Um, we we became overwhelmed with the amount of referrals we had. So we had to hand over our rest of the world refugees. To social services team. Because anyone that comes in as a refugee, that's 16 to 25 from the rest of the world automatically gets a social worker. Whereas the Ukranian refugees don't. They get host families and they get placed. There's no social worker. So all of the delivery around social prescribing and the, um, the measurement tools to measure the journey travelled, that's already done with the social workers. So they already do that with their young people. So it kind of makes sense to hand that pot of money to them that we received from the council. And now they've got that directly. And in the locality that I work in, I think they're working with around 77, 78 young people under the same project. It's just not managed by us anymore."

Code: Project structures
P2, Youth service manager

4.

"So, um, the referral process for (charity) has changed. So a doctor has to speak to a patient and they have to fill out a form, which then gets emailed to (charity) and like filtered through there in case the patient's fallen for any of their projects, then it will get sent to us. So for example, if there's someone that needs social prescribing but is, um, has a drug or alcohol addiction, they will go to a separate team because they have the cab social prescribers within them instead of coming to us, even if it falls in our GP surgery. Originally, uh, you'd be surprised. Um, oh gosh, I'd say about 30% of maybe referrals that would come in, you would call and, you know, they may have, um, the doctor may have mentioned it in passing, or the doctor may have, um, been potentially a junior doctor and then gone to a supervisor and the doctor will say, oh, you know, send them to the social prescribing team. So a lot of the, um, patients you would call and they would have no clue about this referral."

Code: Project structures
P17, Social prescriber

5.

"And there's a certain level of that at the organisation, which is a good thing about the organisation and they have all the other classes and support and legal support and health care support and English and knitting and yoga. So, so women that have some, if they're not in drama, it's not that I'm just going saying go away. There is something else that can do. And when they come to me in drama, it means they're kind of ready for a bit more of a challenge, you know?"

Code: Project structures
P9, Drama programme founder and producer/director

11. Project purpose or objective

1.

"The (project name) programme initially used permaculture principles and horticulture to increase mental well-being, foster the development of self-management skills and the development of work-based skills. Over the years we have moved away from the focus being on getting people into work, as we recognise this is not what everyone needs (and that people are worth more than one they do; everyone is inherently valuable) - many people

attending our sessions simply need somewhere to be, share, learn and connect, but of course the sessions impact their lives in a myriad of ways. An overriding value is that people are welcome as they are… I feel like (project name) - now called (project name) - has had many different purposes over the years. Ultimately we wanted to create a safe space that felt like home for people who have often been traumatised, discriminated against and estranged from their family and friends.""

Code: Project purpose or objective
P23, Volunteer

2.

"It can be self-expression. Freedom to have self-expression, shared experience of creative moments can be incredibly powerful, even if the creative moment is, uh, as it were abstract. It doesn't have to be to do with the experience, or to do with a policy, or to do with a social change. If you have people sharing creative experiences together. And of course, artists are very skilled at designing those creative experiences so that they become that, so that they welcome people in, so they're open, so that they're they become very meaningful, create these points of connection and also wider stories out to the public."

Code: Project purpose or objective
P6, Producer

3.

"Advocating across different levels for, I guess not just refugees and people seeking asylum, but also people who are undocumented and basically anyone who's excluded from mainstream NHS services in a way. And that's really the aim or the purpose of our organisation"

Code: Project purpose or objective
P21, Advocacy manager and primary care lead

4.

Person 2: "And I just thought, what can I do? Uh, you know, I'm a single parent. I've got elders. I didn't even live in (city). And I thought, well, I've got students though.

Person 1: Yeah. Yeah.

Person 2: So what I thought about was that refugees were treated as a burden on the host community. You know “Oh, dear. We've got, you know, all this money spent managing their- “, you know controlling them rather than letting them get on with their lives. Money spent on housing, just making sure that the government could decide their futures when it was ready. Um, but that was what made them a burden. It was the structures that were set up around them. It wasn't the people themselves. But the professional community often treats students as a burden. Um, and I'd often heard people go, “Oh, well, um, you know, oh, you know, we can't have students at the moment. We're too busy, you know, it's too hard. It's too, you know.”

Person 1: Yeah.

Person 2: And actually both groups are high contributors in transition.

Person 1: Yeah.

Person 2: Give them a bit of support and look what happens, you know. So I thought “Okay, um, what about we bring this together?”"

Code: Project purpose or objective
P12, Founder and research coordinator

5.

"And the aim of those is usually to try and spread awareness of (charity) beyond our natural supporter base, because as a writing and performing group, we have a sort of special, sort of secret weapon that we could use, which is that we can get to audiences that that no amount of op eds or campaigning or social media about asylum or the iniquities of the asylum system could possibly achieve. So we can say to people here this amazing concert, or look at this beautiful film, or listen to this marvellous song. And that way you kind of slide behind their defences. Before they know it, they're hearing about all these things they thought they didn't want to know about."

Code: Project purpose or objective
P10, Writing project coordinator

12. Need for project

1.

"Uh, these days, uh, after, uh, when the Afghan evacuation on August 19th, 2021, I personally was involved in going to the hotels that where the Afghan were based, uh, in 14 hotels. Uh, and that was more than 3400 people, Afghan community. So, uh, 2 or 3 weeks a day, I just went to those community members to, uh, provide them with the services that to see the GP, getting their children to school to be enrolled and, uh, with the housing or for the immigrations. So those people are very, very the most needed right now. And they're slowly being settled as well. I mean, some of them have been relocated to other hotels or other area. But, uh, right now the people, uh, the women are very vulnerable within the community. So they're, they're the most targeted groups that from the organisation to get to, to provide the services."

Code: Need for project
P5, Charity director

2.

"Um, we also formed um partnerships with uh, um, migrant [geographical area A] councils, which were, which were overseeing the displaced, the, um, dispersal of asylum seekers and refugees as they come in, and the numbers were increasing, I think. I mean, the data's really, has always been really difficult to define. I mean, that's another conversation entirely. But who is a refugee and who isn't? How long are they a refugee for? So how are they counted? All those numbers were really slippery and really difficult to get, and we realised we kind of needed them to make the argument for why would you do this type of research in the [geographical area A], where the population, the numbers of people who are displaced is so much smaller than [city C], [city D], [city E], [city F]. Any other major conurbation. But I think our argument was, which eventually won through, was that exactly that. You know, because it's a very high white population. So [city B] you know the percentage of white national population varies, but, um, the estimates go from 93 to 96%. So incredibly difficult to be a displaced person within that environment. And, um, you know, the whole agenda around integration and what does that mean and, was rising, I guess. And [city G] in the [geographical area A] is where a lot of people arrive too, but then they're often dispersed from [city G] down to [city B] because housing is cheaper."

Code: Need for project
P4, Senior researcher

3.

"So I guess we developed this service in response to the use of (town) and concerns that we've heard from other organisations about the impact of the site, you know, the isolated nature, the impact on people's mental health, but also the kind of unmet health needs that

were, were there. So it's likely, uh, the health care commissioning side of it is slightly different to in hotel accommodation, although there's problems with that. Um, this was kind of a private provider, um, who delivered health care on site kind of, back in the middle of the day. No evening kind of service. Um, yeah. So quite a limited service. Well, better than nothing but no links to local secondary care, no like specialised mental health services... Um, and yeah, from hearing concerns from other organisations that provided other services on the site, um, we set up this service, which was a mobile clinic. So we didn't have it inside, it was outside the gates of the site. We didn't have permission to, to go into the site. And that's because there is the on-site medical centre. Um, but what we found really was that there, there was this massive level of unmet health need. The impact of the site in particular, um, resulted in deteriorating mental and physical health of the, of the people living at the site. So ultimately, the I guess, the aim of the service was to provide medical care, I guess short term medical care, um, referring to other services to other organisations, documenting, uh, human rights abuses, but also, I guess, collecting data or collecting evidence to advocate for the policy change, to basically close the site and encourage the government to stop housing people in these large sites."

Code: Need for project
P21, Advocacy manager and primary care lead

4.

Person 2: "We got wind of the fact that the authority were going to stop all language classes.

Person 1: Oh.

Person 2: Um, yeah. Um, so that was community education. Who delivered on behalf of (region) Council. Okay. They were going to stop delivering because of funding issues. Um, w-, yeah, this is happening all over (country), actually, but, um, I think. Yeah. Um, (institution) were continuing with the online lessons.

Person 1: Mhm.

Person 2: Um, but we thought, well this, this is, this is a desperate need. I mean, you cannot possibly come into a country and be part of the community if you can't speak the language. Um, I, I am actually incandescent with rage. Uh, the fact that it is so hard for people to learn English, from what I hear, Germany is very different in this regard.

Person 1: That would be correct. Germany has a very different approach to it. Yeah. Okay. Yeah.

Person 2: And, and it's just absolutely awful. Now I think community learning did in the end do some kind of lessons, but we never quite understood what they were or who could access them. And basically, um, community learning isn't what it used to be. Um, you know, and it's a shame anyway."

Code: Need for project
P13, Charity chair

5.

"So, anyway, um in 2001, um, we heard that refugees were being dispersed to (city), because of changes in law that meant that people were being sent around the country. They were accommodated in privately, uh, managed housing, on Home Office contracts. (City) had quite a lot of low-quality housing and (business name), the robbers, got the contract and I thought, actually if people coming to (city) they're not going to be entitled to benefits to any kind of social care support. It's all meant to be done by the housing provider until the point where

they were given leave to remain or deported. So I thought, really, people will need some sort of support, and that sat very well alongside a determination to teach white West Country students how to, how to recognise their whiteness and work in a multi-national setting. So students used to say, well how can we do work anti-racist work? Because there aren't any black people here. And I say no, but there are plenty of racists."

Code: Need for project
P12, Founder and research coordinator

13. Project background

1.

"So it's a military site in, in or an ex-military site in a very remote location in rural (region), um, which was opened as kind of asylum accommodation, um, not strictly detention because people technically can leave, um, but it's very isolated. So by, by nature people are kind of trapped in, in the site. Um, so it was kind of new government policy to open these large sites to, to reduce the, the use of hotel accommodation for people seeking asylum in, in the UK. Um, the site has capacity of about, I think 800 people. It's probably between about 500, 600 at the moment. Um, and people can stay there for a maximum of nine months, um, um, although that wasn't initially known when we started the project there. Um, so yeah."

Code: Project background
P21, Advocacy manager and primary care lead

2.

"So we have a hotel that is being used and that is for families. Okay, so we've got quite a few families that have come. Yes, we have uh, uh, quite a number of single males, but also single females. Um, the, the countries or the, the languages that we provide we'll determine that they are from places like Yemen. Uh, Syria. They've come via loads of other countries. And whenever they get refused in one country to go to the next one, some of the people have been travelling for four years."

Code: Project background
P18, CEO

3.

"Students, well, had to use their own abilities. They had no, literally no resources. We kind of… postage or anything like that, um, went through the university, we'd get, buy dictionaries. We've got, you know, they just there was no Google Translate or was it just the beginnings of it in those days. And they, they just they just made it work, they and some of the refugees together made it work. Because it was part of my job, it became the most interesting thing that I was doing, as part of my job. Um, because a lot of it was, you know, academic politics. And so it meant that I could actually feel like I was doing something, doing social work. So in the first year, uh, we, we set up a kind of steering group. It closed the first summer because there were no students, but otherwise we had, we found, you know, locally there was a Master's as well as an undergraduate course. And so those students kind of, uh, their placements overlapped. So, you know, they, they provided the service between October and May, June, and then it closed in the summer. And then we started with them. And all the students passed through the academic boards, even though they only worked with maybe three families each. Something like that, but they were, they were doing holistic social work that people needed. And, um, the second year, um, when it got to the end, one of the students who was by then graduating said you can't close it in the summer, people need it."

Code: Project background
P12, Founder and research coordinator

4.

"I don't actually, I mean, it started in 1997 or 1998, I think, so 27 years ago. And it was started by a woman called (name), who is a playwright. Whether she was somebody who knew (founder), I don't know. I came along a couple of years afterwards and then took it over, sort of after that. But as I said, when I started, it was just a few people just writing, you know, they would photocopy their writing and circulate it to each other, but that was it. And then they sort of started saying, well, you know, what's the point of doing this if nobody's going to read it? And their stories seemed quite important, and they cared about them, and it was hard work. It's hard work writing in any language, let alone, as I said, your fourth or fifth or sixth language. So they said, well what's the point in this if nobody's going to hear it. So we started looking around for opportunities and I was lucky enough to have a volunteer at that point who was quite well connected with the, um, people who ran the (refugee) sessions at the (Festival). So she got us into those and for quite a number of years, probably a decade after that, we used to send work to, to those sessions. And in the early days we actually used to travel up to the (Festival) to hear the work being read."

Code: Project background
P10, Writing project coordinator

5.

"Well, it was in a field because there were no rooms there, you know, it was all open air. So there was a goal post and it was an old 50s, 1950s kind of Butlins type camp. So there was a concrete goal post. And so I just put like theatre curtains like, um, net, you know, brightly coloured, made that like a bit. And then because of language, you know, I spoke French, which was good for the people, the French Council and the French charity workers so I could but um, there wasn't much communication with words, which is another thing that came to mind, so it was very objects based. So I'd have, the one example comes to mind, one box of sequinned fabrics. So I just put the box of sequinned fabrics in the by the goalposts, see what happens, you know. And so, you know, in that instance, you know, the women and their children would dress up and put their music on and we'd have a dance and a fashion, you know, fashion show. You know, and laughing again. And that's where the kind of health benefits come in. You know, people who don't laugh and play with their kids. It was lovely to see them laughing and playing with their kids. So that would be one. Or like little mask templates. And we all make masks and, and there's, because there's, it was a challenge and a barrier not being able to communicate through language. But, but I think it also opened possibilities up because if someone's drawing on a mask and making a mask and then they become something and then everyone's guessing what it is and this is drama. So they're "I'm this person". "I'm this thing." You know, and it's beyond language. It's, it's more, uh, the language can close things down sometimes, where actually kept things. The misunderstandings were part of the joy of it. Or they enabled things to happen. So, in short, came back to (city). Uh, googled women, refugees (city), and then went for 50 women in (city). And so went in there. They were looking for a yoga teacher. I said, I'm not a yoga teacher, but I'm a drama teacher, like one of my hats and, um. And they said, yeah, give it a go. So. And that was January 2016. So we've been doing drama every week since then."

Code: Project background
P9, Drama programme founder and producer/director

14. Personal motivation

1.

"I could see that people seeking sanctuary faced multiple barriers and stigmas including lack of financial aid, being cut off from family, friends or those who share their mother tongue and culture, the hostile asylum-seeking process in the UK, racism, Islamophobia, stigma

stoked by tabloid newspapers and the UK government. Over the years this has gotten progressively worse, with people we work with regularly being threatened with being “sent home”, including vulnerable children."

Code: Personal motivation
P23, Volunteer

2.

"But yeah, you're not addressing the systemic racism. You're not addressing the inequalities. You're not addressing everything else that there is. Right. But for me, social prescribing is a way of acknowledging that, even though that was not the initial intention, but it does acknowledge that. And that's what that's why I do it. Otherwise I wouldn't be there, because I don't agree with the ethos of the charity that I work for. The only reason I'm there is because I know it's, it's something worthwhile. And even though the initial intention wasn't that, um, so yeah, for me, social prescribing is, it's a holistic approach to taking care, which is what I need."

Code: Personal motivation
P7, Social prescriber

3.

"Uh, that's, uh, in 3-4 years, uh, I will after that, I will be retired, but still, um, working, still I am working, (Person 1: Yeah.) um, volunteering working. (Person 1: Yeah) Uh, which is, uh, when I see the people, I can help the people, I feel very, very good. (Person 1: Yeah.) In my heart, I feel, oh, I will help the people. And, um, I always do back home, up to still that time, my student is still with me. Uh, although I was just a teacher, when they paid, I teach them, but still they contact me, they still they're going to everywhere they have contact me. (Person 1: Mhm.) So um, because of I help them. (Person 1: Yeah.) They, they, they're human, so they need, they knows how to help others and, um, (Person 1: Yeah) uh, they knows, uh, what should I help them. (Person 1: Yeah.) So that's I think it's, uh, every human should be help each other. (Person 1: Yeah.) In short life, we have to help other people as well, which they need it."

Code: Personal motivation
P3, Youth and sport manager

4.

Person 2: "I see, but I think all our staff are so committed to supporting the community that they freely give their time. I’m privileged to work in a, in a setting where, where people are not just there for the money. They are there because they want it. Well, they wouldn't come for the money because, you know, we're paying pittance to everybody. Uh, but they do it because they feel very strongly connected with their communities.

Person 1: And they, and they see the purpose in what they do. Yeah. And, um.

Person 2: Helped building those relationships. Yeah. Yeah. Yeah. You know, and lived in those communities."

Code: Personal motivation
P18, CEO

5.

Person 2: "The joy.

Person 1: Yeah.

Person 2: Absolute joy. Um. I have never spent so much time because a lot of our work is with the women, um, for lots of different reasons. Um. And in the company of women, you

know, they are just so joyous and always laughing and, um. And I just think, wow, I, I didn't have that kind, I, um, I've been, um, yeah. Anyway, that's for me is one of the big things. It's purely joyous. But also, I think when the lady who had come to me a few times to say, can you help me book the hall and, and I said, okay, this is what you need to do. I will help you. You'll need to fill in the form, do the bleurgh. Um, when we got to the point where I said, okay, you can do this yourself now, you don't have to come to me. That is good, because she's now independent and can book the hall, and she knows she has to do it more than two weeks in advance. Yeah. Absolutely. Yeah. Um, and, and, and, and then you have the twinkle in the eye, the broad grin when you, um, at the end of the lesson, you know, and everybody has tried really hard to spell cat, at, but and you say, all right, what will we, what will we give you today? Is it ten out of ten? Um, and just that real sparkle of mischief. And one of the men in particular, as he gives himself 100 out of ten.

Code: Personal motivation
P13, Charity chair

6.

"And now through (charity). I've always thought this way. I've always looked for if something happened, I've always thought, well, well that's good, because. I can't think "Oh no that's terrible. My life is ruined." I'm not seeing my life ruined by circumstances, you know. Instead, it's "Okay. Okay. What can we do with this?" That's, that's been the way I've always worked is look for opportunity, look for possibility. And I've always tried to do that with other people because I know I can't do anything by myself, apart from my allotment at the moment. It's that kind of, it's collective you know, the best results come from working with other people, people who know most about the problem, you know. You know, students know most about living in this modern world. I don't. Bewildering, frankly. Refugees, they know most about managing in really hostile circumstances. Why would we not work alongside them to try and make it better? And that's, that's the strengths approach. It is that, it's the constant, it's the refusal to lose hope, not just optimistic, because optimism is like "Oh yeah, you'll be alright." It's the Rebecca Solnit, you know, because life's not a lottery ticket. It's an axe that you have to break down the door with, because it's easy to go "Oh there's nothing I can do". But the strengths approach, as a student once said to me, there's always something you can do."

Code: Personal motivation
P12, Founder and research coordinator

15. Decision-making and values

1.

"Um, so with that, I saw the role come up. I was interested in social prescribing because I'd had an introduction to it. Um, years back in America, where we were referring people to gyms from cardiac rehab. I worked in cardiac rehab. Um, and I thought that was a very interesting link, um, at the time. So I suppose I had that idea in terms of social health and social capital and all that for a long time. So when I saw this role came up, I applied for it and, um, and got it. That's my background and they're all great."

Code: Decision-making and values
P22, Social prescriber

2.

"So that's how I first talked to people about their experiences and really saw the trauma and the devastation and the really like the. Anyway and this is just my personal opinion, of course, just, I just had this feeling that there's just a lot of hypocrisy, um, in the systems that we build and how we talk about human rights and how we talk about social welfare. And because when you actually, at least in the UK and in many other European countries, but I

can only speak for this one, but you can see how there is a perceived sense of, oh yeah, we're taking care of the most vulnerable without actually focusing on how the law stops you from actually taking care of some of the most vulnerable, which are usually single mums of British children who have got no status, and they're living in one room rented out by someone who could easily exploit them. So and that's everywhere in (city). I mean, it's really not a minority at all. Um, so anyway, that's when I got in touch with that sense of, ooh, we're not really doing a great job as a society in many ways."

Code: Decision-making and values
P7, Social prescriber

3.

"I will start right from the very beginning, shall I? So, um, when I was a child, I grew up in quite a disadvantaged area. My parents were foster parents as well, but I was a home grown. Um, so I sort of had experience with lots of disadvantaged children, and my parents are very involved in the community. So I kind of grew up in an environment which was supportive of, like, other families and not just myself. So I think that's where kind of that embedded kind of duty of care stuff comes from. And that, that kind of, um, desire to help others."

Code: Decision-making and values
P2, Youth service manager

4.

"Um, I worked a little while in a local hospital at that stage, but, um, the Netherlands was still quite, I don't know whether I would call it behind, but in the Netherlands, the push for mothers to go to work was not very great. So the culture was very much such as, you know, when you have children, you become a homemaker. And that's a very valuable job. And you know, it's not oh, I'm only. So I then, I have four children, but after my third one was born, I couldn't really afford not to do some work, so I became a registered childminder and I was registered for seven children under the age of five, which was amazing. And it was during that time that I thought, you know what, I need to diversify. I didn't really enjoy, um, the work in the hospital as much as I had been doing when I was in the Netherlands. So I think the protocols here was still very stiff. Uh, and I was introduced to that. So during that time I did an advanced diploma in child care and education. So that would have given me the opportunity to work, um, as a manager in the child care setting, but, um, it took a slightly different role. So when I went back into work outside the home, I actually diversified."

Code: Decision-making and values
P18, CEO

5.

"And again, I say that glibly, but you know what I mean? And everyone said, oh, there's no women but I've always been a feminist researcher or feminist person. So, um, everyone said there's no women in the refugee camp in (French city). You, you won't find any women to work with. Of course, there were loads of women, but they were hiding a lot of the time, right in plain sight. Because of all the patriarchal reasons of violence against women. Girls, um, cultural difference, looking after children, all the things that. Yeah, that are aligned in a very different way. I don't want to make any false equivalence equivalency there, but in aligned ways to why women are generally invisible in, in the world, generally."

Code: Decision-making and values
P9, Drama programme founder and producer/director

16. Work experience

1.

"When I first started volunteering I would predominantly make soup for participants using freshly grown produce on-site. We would cook together over a campfire, sharing stories, joys and woes, singing songs and learning about different plants and cultures."

Code: Work experience
P23, Volunteer

2.

"So my background is in nursing. I was 25 years nursing, um, between Ireland, America and I lived in the Caribbean for ten years. Um, so that is my nursing background. I then moved into health care support teaching. Um, and I moved from there."

Code: Work experience
P22, Social prescriber

3.

"I was really, really worried about because this system, how to bring a fund? And also the English second language for me, is another barrier for me. And in sport, other things, we have something, they can have an instructor to instruct them what to do, but in that things wasn't anything for me. (Person 1: Mhm) Uh oh gosh, it was really hard time for me that time. And then I said, no, I'm going to make it. I said my mind, I say I'm going to make it. Otherwise even the people, the community, they're thinking, oh when I'm coming this close down. So no, I have to make it. So, uh, I said my mind, um, I learn. Uh one day I was in Refugee Council, so, um, their opportunity to learn how to do the fundraising. (Person 1: Mhm) I went there for one day, uh, training. I learned a lot, to be honest from there, a lot, a lot. I wasn't sure when you sent the, um, application how long it takes to process this. When I learned that I start from small grants, uh, to, uh, become big grants."

Code: Work experience
P3, Youth and sport manager

4.

"Um, so my background, I'm a trained musician. Um, then have worked in the arts, uh, for 15 years or something so of course different art forms and creativity and I guess, um, across all of that have been using music, drama, dance, all these, all these different art forms to work in communities, um, to hopefully at times facilitate, like community healing, um lifting up people's voices, um, in different ways. And so through all that work, um, you know I've worked with young people, worked with, um older people, done intergenerational projects. Then, um, worked with sex workers, worked in different places where to, like, advocate for people's needs using the arts. And then I guess as part of that, ended up working in projects that were working with refugees, asylum seekers. Um. And all of that, then later on led me to where I am now, where I, I still do a lot of that um, but also I trained as a therapist, so I am now dance therapist, um, and work for two different organisations as a dance therapist. Both of those organisations where I'm also working with a lot of refugees, um, and until a few weeks ago, I was working as a social prescriber as well, again, like looking at that, um well, working in the mental health sector, I suppose more broadly one could say that social prescribing is within the mental health sector."

Code: Work experience
P1, Social Prescriber

5.

"And so we were looking for more staff. I joined the organisation, initially as their volunteer coordinator, which I had for about the role for about six months. Then I moved on to being the integration manager, in which I run the ESOL classes, as well as other integration support, and most recently actually, um, I yeah, I mean, I've taken a number of different projects, like helping people on probation, um, helping people with different, um, um, homes, energy. I do quite a lot of different areas for the organisation."

Code: Work experience
P8, Integration manager

17. Migration history

1.

"And I came to this country late in 1994. So I was the one of, uh, their client as well, uh, because when I was coming to this country, I was struggling with the same issue that I providing to the other Afghans, fellow Afghans. So, uh, I received the support from this organisation when I came first to this country."

Code: Migration history
P5, Charity director

2.

"I was, uh, work back, um, at home, uh, um, International Olympic Committee as a professional person and coach. Uh, and I organised many, uh, sport, uh, tournament and uh, teams, great teams and the sorts of things. When I came here the first time, I started here with the (charity name) as a volunteer and, um, as a sport, uh, person, then, uh, became a chairperson then after."

Code: Migration history
P3, Youth and sport manager

3.

"Okay, so I'm Dutch. I lived my childhood till I was 23, in the Netherlands. In that time I became a paediatric nurse and I worked in a hospital, um, a paediatric hospital with, and my, my responsibility was for children, uh, 0 to 1 and a half, two. And then got married to an Englishman which meant I moved over to the UK in 79."

Code: Migration history
P18, CEO

4.

"I'm from the US originally, but I've um, been living in the UK since September 2020."

Code: Migration history
P8, Integration manager

18. Education

1.

"So, um, I well, actually, I studied music at (name) University a long time ago. Um, but was always interested in sort of the socio-political side of music and studied a lot of music from around the world and looked at music in protest. So I always had kind of international head on. Um, but then wanted to do something very practical, um, to help, help the world. So I went into teaching, actually did a PGCE, and went to primary teaching, um, with the long term goal of moving more into international development and education."

Code: Education
P19, Schools coordinator

2.

"I've lived, I lived in, in the UK for a long time now and studied here, but also worked and lived and studied elsewhere. Um, and I guess I have always and I've had, um, sort of weirdly a strange thing, from about the age of 16, 17, um, had a quite an international upbringing in that I ended up going to an international high school, um, and have just always felt quite comfortable in international communities. Also, living in a country that is not my country of

origin. Um, I have originally studied applied arts and theatre. Um. And that was a subject that was sort of, it spoke to me because it was a tool with which to work with people."

Code: Education
P15, Wellbeing manager

3.

"Um, and then, um, I went off to uni, um, but I didn't do education or teaching. I went into social work degree. And then I was placed in a youth service for my first placement. And then after my first year, I decided that I didn't want to do social work. I wanted to be part of the prevention side rather than the side where things got really bad. And then they get referred because that's when social workers normally come into play. So I did a, um, a degree in youth and community, um, which was amazing. And it was on the ground as well. And because I was an, a mature learner, it suited me better rather than living that whole traditional uni lifestyle with a bunch of 18, 19 year olds that were just getting drunk. Um, so I lived independently, worked 25 hours a week, and then worked my degree around that as well. So it was a really nice experience. I was so fortunate to have it because I never expected, like coming from where I came from. I think I'm like one of the 5% of like, um, students that go back and study from disadvantaged areas. So it was really nice that the uni loved that, and they gave me some extra grants so I could like live. And yeah, it was really nice. Um, it was [name of the University]. So when I started it was like a college and then it became university status after that. Um, and then I graduated, I think it was in 2004 and I went straight into youth work. So I, um, finished my diploma and then my BA, um, and I received a, a youth work BA, um, which was first class. So I was really proud of myself. Um, and then I was offered a postgraduate, um, course, actually, where the university paid for me to study for two years, and that was actually based around refugees. Um, and I really wanted to do it, but I was in debt. I was also hungry for work as well."

Code: Education
P2, Youth service manager

4.

"So during that time I did an advanced diploma in child care and education. So that would have given me the opportunity to work, um, as a manager in the child care setting, but, um, it took a slightly different role. So when I went back into work outside the home, I actually diversified. I did a degree in professional service in education, and I was specifically looking at, uh, disabled children and their parents and the, the reason why partnership working is so important. Because without doubt, you know, whatever you do in isolation isn't going to work. Um, and then I still don't know where I got the email from, but I got an email saying, if you're interested in doing a studentship, doing a master's by research, then, you know, apply, which I did. And it was funded by somebody in Ireland, an organisation, that were making adaptations for disabled children, especially with physical disabilities. So they funded my, my studentship. And, um, I looked at the advice-giving strategies that allied health professionals use with parents of disabled children. And, and I only finished up in 2014."

Code: Education
P18, CEO

5.

"I came over here initially to do a master's degree in international migration and public policy. So similar to you, I migrate for, well, partially for study, but also, um, and yeah, I did my master's and it was a one-year program afterwards."

Code: Education
P8, Integration manager

19. Language barrier solutions

1.

"Definitely. Google translate has been a life-saver, as have past practitioners and participants speaking multiple languages e.g. Arabic and Urdu. However we have not found language barriers get in the way of connection. The farm is a great place for people to practise their English, particularly as there are restrictions in place when folk initially arrive in the UK e.g. they cannot work or learn English straightaway, isolating them further."

Code: Language barrier solutions
P23, Volunteer

2.

"So some time uh but with the other job centre and other departments, I mean some time the people were uh, uh, unfairly, uh, pushed, uh, to find the work or why they are not, not working. Uh, despite they're, cannot speak English and no one can take, uh, people to work that can't speak, instead of encourage them to send them to courses with the colleges. So I have a, uh, meeting here, whether there is a job centre, there's a department that working with the Jobcentre that, uh, uh, been people were introduced to that department. I don't know what's the exact name of that department here, but people were introduced to find employment, um, that organisation. So some of some of the Afghan people, they are sending here as a volunteer until they find the job, um, uh, but uh, uh, we cannot employ, uh, the people also, they cannot speak English with the proper job, because all our jobs, even working with the, with the Afghans, some of administrative workers in training in English. So people has to speak English, write English and know uh, the, the rules. But people were sending here to find the job within the community. And I say working with the community is not, it doesn't mean that only they can speak partially, uh, out for native languages. It has to be, but yeah, as a volunteer to support in organising the event and uh, uh, supporting, uh, other, other such activities. Yes, but that we don't have a such, a rule to, to employ such people. But, uh, we can certainly can help them. Uh, learning basic computer, uh, using the computer. Uh, other, other, other things that they can get familiar with but cannot support. So I speak to those people how to, uh, I say, if someone comes to you and cannot speak English, so you're not welcome to employ them. So let these people, encourage these people to go to college and learn language first and then, and invest in those people first and then then go to proper job. So I had such meeting with the people with that department to not push too hard those people, especially, especially some of them are elderly, that, uh, very close to pension and then, uh, to learn English is also so difficult for those of such age. So, uh, yeah, I certainly recommend how to behave with, uh, with those people instead of pushing and sending them to work."

Code: Language barrier solutions
P5, Charity director

3.

"And those care homes have specific activities. So I suppose when, when I say we're not looking for those activities for the kids that can't get out of bed, we are, because we are encouraging them if they can't get out of the house to at least engage in those activities within the house, which are mostly kind of learning independent skills, cooking, budgeting, um, socialising, learning some English with the staff members, learning British culture if that's a thing, sharing their culture, um, and making friends with the other kids in the house who will have different but probably similar experience, experiences, at least with some overlap, they won't have all experienced the same traumas, but they will have experienced some of the same issues on their journeys. So we are, we always encourage that."

Code: Language barrier solutions
P16, Quality Assurance Teacher for post-16 education

4.

"But a lot of the girls we do have arrive pregnant, which is a horrible thing to think about, but they do. So physical health is a massive, massive issue, um, as well. And actually the first PEP I ever did when I started this job was for a young girl who was pregnant and she was 16 and she was unaccompanied asylum-seeking child, and the PP plus was brand new and no one knew how to use it. And we had way fewer restrictions on how we could use it. We could be really creative, which we're not allowed to do anymore, and we spent it on a Netflix subscription for her so that she could learn some English while at home with her baby in a way that was still maternity leave, and not asking her to work through her maternity leave. Um, so I think physical health conditions are probably under discussed and underrepresented in this conversation. And so is pregnancy, which I think should constitute a physical health condition, if it doesn't, I don't know what it is. So yeah, just that I think that's the only other thing. #01:10:59-6#"

Code: Language barrier solutions
P16, Quality Assurance Teacher for post-16 education

5.

"But on the, on the on the other hand, we have four community English classes that are run by volunteers every week. (Person 1: Mhm.) Um, so almost every day, every working day there's um, there's an English class. Um, and they are, they are depending on volunteers. They can be very social. They can be sometimes more about like they are not um, graded class. So you don't need to take any level exams to come in and you just walk in. Um, and, and sometimes they seem to be some teachers are more about eating cake than, than, than much else and um, uh, depending on the teacher. But um, but that's been going on kind of solidly for a long time."

Code: Language barrier solutions
P15, Wellbeing manager

6.

"And I think, um, I think that's much to do with the skill of facilitation we've got at the moment. (Person 1: Yeah.) Uh, Where we've got really skilled and very confident facilitators who, who know how to kind of throw a whole burst of energy into the room or into the group, um, make themselves be the silly person. Um, um, like if it's about trying something new, then you, you can be the first person to try and not necessarily try and fail and, and kind of make an example that anybody can try. Um, any outcome is okay. Uh, and then also people who have the skill of, like, then kind of step forward and step back where you kind of step forward and juggle balls in the air and get people talking and involved. And then, then you sort of step back and let people take ownership of the situation or carry on. Um, but there's, there's a real, um, art to that. And I think, um, I think we've got a team of three people who can all do that very well. And, and therefore we will often manage to engage all the participants on some level. Even if it's just touching base with everybody at some point. Um, "How are you? Everything okay?" Um, or in situations like we've had this sort of cooking together sessions where you will manage to come up with a tiny task for everybody, um, including the ones who don't straight away pounce onto the chopping stuff and, and or at least make people feel like, they feel comfortable being part of it, even if they didn't necessarily contribute to making a thing."

Code: Language barrier solutions
P15, Wellbeing manager

7.

"So we've been given the money and we, uh, we have now the first ethnic minority specific, uh, drugs and substance misuse website in the whole of the, uh, in England. Um, and the reason why we did is we made sure that whatever language was spoken and whatever

information we were putting on that, that, that was actually translatable because, um, yeah, I mean, you will know in German and I know in Dutch, so you use a lot of, of adjectives that, that, that just aren't in the UK. And whereas in the UK it would sound more like a command, whereas yeah, the Dutch are much more subtle about, I mean we're straight, we're straight to the point, but we're also very subtle when we want something or we're talking to children, for example. And so they just, the drug talk UK website was born okay, just it was launched in November and we're now looking how can we best, um, use the comms. So we've just set ourselves up with TikTok as well because that seems to be, you know, a really good way and just very short messages so that they're hungry for, for something more. And then, you know, the, the link is there to come onto the website, um, anything that is an editable PDF or, or literally written on the website, it can be translated instantly in over 100 languages."

Code: Language barrier solutions
P18, CEO

8.

"Um, so there were definitely plenty of people I was working with who whose English was, um, pretty good, um, and, uh, it wasn't a problem whether they were from, um countries like former British colonies and their English was, um, they spoke in English all their lives, or they were just they like, just been, like really quick learning the language, or they'd learnt it through TV or whatever, um, but then I think I mentioned earlier that, that I would often have to use translator services as well. Um, and that is really challenging (Person 1: Yeah?) even some translator services would use telephone, um, interpreters, do the language job. Um, and obviously, yeah, it's quite random who you get assigned, um, as an interpreter. It would also sort of create this like buffer. Um, you call the interpreter and sometimes you're waiting like ten minutes to get assigned to someone and you're there sitting with someone at times who's like already in distress. Again, you're not supposed to be seeing people who are in crisis, but often I did meet people who were like in extreme, um, difficult situations and felt in crisis. Um, and then with that added distance of the interpreter through the phone like building that human connection can be really, really difficult. And sometimes we'd get interpreters where, um, it didn't feel like that, like they could handle the distress as well that was happening. And they would say, like you know, please, further would be like, so can you please slow down? I can't, um, translate quick enough, we were talking over me or whatever. But in the room, I can really feel like the client the reason that they're talking over or the reason they're talking so much is because of the distress. You know, it's very understandable. Um, I actually had one interpreter who was like 'look, you're not letting me speak, so I'm going to go' and just left. (Person 1: Uh.)
And like, you know, I had some great experiences with attempted as well, but it was so, it can be so hiddenness (Person 1: Yeah) Um, and yeah when you have someone in distress who is also like maybe crying or other things. It makes the interpretation, like, so much harder over a phone, the sort of crackly phone, like all of these things. It's very, very difficult, whether or not you speak the same language, to be able to hear someone while they're crying and stuff. So um, yeah, using interpreters is always a challenge, but I'm also just, like so grateful that we had that service, that GP surgeries have that capacity. It's extremely expensive, I know that, um, and in other settings, other settings, even in my work as a therapist, it's very hard, it's like impossible to offer, um, interpreter services. So yeah, I'm glad that we could offer that."

Code: Language barrier solutions
P1, Social Prescriber

20. Cultural barrier solutions

1.

"It can be easier for people seeking sanctuary (and people generally) to access support in a non-pressured environment such as the farm. Sharing a traumatic life story feels less daunting when you’re sitting side by side planting potatoes - folk do not have to worry about eye contact, how they come across, and being outdoors can feel safe and expansive - EMDR therapy was founded in nature and I can see why.

Connecting with people over growing food and eating food. Gardening can be an emotional and cultural bridge."

Code: Cultural barrier solutions
P23, Volunteer

2.

"And I suppose this is an area of interest of my own is, you know, is time linear, is time… You know, something else. Um, so timing and arriving on time. But as I say, I lived in the Caribbean for ten years, so. Yeah, concept of time is, is difficult. Um, so, you know, arriving at something, you know, at the time that it's at a case is, you know, trying to convey the this is actually going on from 10 to 11. It's not going to, you know, continue for hours on end. Um, that's, that's an interesting issue. Um, I don't know if I'm answering your question. I find that people have stories and, you know, my whole career has been listening to people's histories and stories. Um, So, um. And, you know, when I, when I worked in places like the rape crisis and, um, um, women's refuge stories have gaps, you know, stories of holes as well, because people choose to tell you what, what they're going to tell you. So I think there's often an element of, you know, um, you're getting a partial story or, uh, yeah. And there's other there's other things as well. Like I might see counselling, I might value counselling, as, you know, an intervention for somebody if they've had a very traumatic, very, um, difficult past. And they're coming here sitting with that trauma in a new culture. And not everybody wants to revisit the trauma. And I think that's, that's been a learning for me, um, that, you know, people, people want to focus on their on their future and going forward. Uh, they, they don't really value the concept of looking back and, and um, and dealing with that with that trauma."

Code: Cultural barrier solutions
P22, Social prescriber

3.

"And he was the one who took it in the direction of being arts and culture led. So his argument was, yes, we need a week that is about refugees. But instead of it being sort of through with policy and to do with focusing on, like the themes used to be like, um, whatever, genocide, or torture, or whatever, because these are the things that, you know, activists just want to draw attention to and also to fundraise in relation to. And he said, well, let's spend a week talking about positive stories through arts and culture where people can represent their own experiences. We can get much more feeling of the full range of experiences, um, and also make points of connection with the wider public. Because actually people can only hear about these difficult things so much before they switch off. So that and that was really successful. People really welcomed that.”

Code: Cultural barrier solutions
P6, Producer

4.

"And, uh, and also with gender, that was another thing that we were very clear that if somebody was a muslim woman, it was likely that they would prefer to have a female peer support worker and probably somebody who speaks their language via Arabic. In reality, not everybody wants to. What we found out through the co-production process and through peer support work is quite often within a very small city that where everybody knows everybody within a certain cultural or linguistic group people don't want somebody from

within their own community to do the to do the interpreting, to work with them. Because they've had prior experience, lived experience of interpreters being selected from within the community and then passing information on, not treating that information as confidential. And they're afraid that of the shame of admitting a mental health problem or admitting that something's not going well will be transmitted to maybe their husband or to maybe their entire community. So that assumption is not a valid one. So we tailored. So we actually ask those questions of the peer. So the researchers are the people who make the first approach rather than a peer support worker and go through with them the metrics which are the baseline metrics for the study and also do an interview with them around what kind of person they would prefer, what their linguistic background is, and it just hasn't been possible to match them linguistically, language wise, and in fact has been incredibly illuminating that that isn't the key thing, that there is a way of making yourself understood. And the empathetic personality, the open listening personality, the person who allows a slow building of trust is far more important than somebody who's from the same cultural or language background."

Code: Cultural barrier solutions
P4, Senior researcher

5.

"And some schools do really well in supporting students who have English as a second language. So when I can think of in (region) of the city I visited for a young person who actually spoke very good English, but she was an elective mute, so she was actually put in the, put in the group with the other unaccompanied asylum seeking children, because she had similar communication needs, I think. So they were kind of trying to give her access to some of that. So I went to the centre and kind of had a look around and learned some of their strategies, and that was absolutely fantastic. Like they were doing 1 to 1 lessons, sending teaching assistants in with the young people into lessons to, not translate, but to give them a sense of what it was they were supposed to be doing so they could pick up some of that language, on top of which they had all of the unaccompanied asylum-seeking children accessing the centre at breaktime and lunchtime out of choice. Um, because there were fun activities in there, which meant that those who'd been here quite a long time and picked up quite a lot of English were kind of supporting, helping the ones who'd been here for less time and helping them translate some of their work and understand what was actually happening. Um, when I went, I sat there through lunchtime and there was this ongoing jigsaw puzzle. So you had like six massive teenage boys, like, crowded around this jigsaw puzzle of like, can’t remember what it was, some like, very famous building. It might have been, I've been, um, I can't remember, but some famous building, like finding the pieces, speaking a kind of mix of Arabic and English. That was quite cute. So that was that was like a really good system. But that said, there weren't any formal English lessons. Um, aside from the GCSE ones, most of the schools that I've known in my own area that I've worked in have had absolutely nothing."

Code: Cultural barrier solutions
P16, Quality Assurance Teacher for post-16 education

21. Mental health and trauma solutions

1.

"Um, the second issue I have is timing of interviews and appeals and rejections and all of that. So if we have a plan and then the interview comes up, there's this stress, like serious stress before the interview. Yeah, there's the day of the interview. There's a recovery from the interview because the trauma has all been triggered again. Um, and then there's the waiting and the fear. So there's a difficulty in concentrating on a plan. So what I tend to say is let's just try distractions. You know, you have all this trauma, you have, um, all this, you know, ongoing trauma now, in fear with, you know, whether you're going to be accepted or

rejected. Um, so let's try and look at it as a distraction. You know, we can go to the mother baby group or we can go to the, the, um, talk group, or we can go to a um, heads up group or whatever is on and try to concentrate in the moment, um, you know, on this as a distraction because, you know, it's hard to concentrate on a social event or a social activity when you have all this going on in the background. I think I've never been through the system, so I'm just trying to understand."

Code: Mental health and trauma solutions
P22, Social prescriber

2.

"Yeah. Yeah. That that is true. Yeah, probably I probably don't I'm probably not conscious otherwise. Um, when I'm speaking to somebody, but, um, obviously, um, for different religious beliefs and reasons, you know, some people don't want to be in a mixed, um, gender group. Um, some people would prefer to. I'm trying to think... some people prefer to listen. They don't feel like their story has value in the group. Yeah. Um, so, you know, sometimes if I sit with somebody, I might go to the first meeting. Yeah. Uh, say to a women's group, a chat group. And I find that the, the person I've come with doesn't feel like they have a contribution to make. Um, and it's trying to, you know, um, give value to their story and explain that, you know, their story and their background is actually very, um, very valuable here and interesting here. Um, um, sometimes there's a time can be different."

Code: Mental health and trauma solutions
P22, Social prescriber

3.

"We, we tend to be quite open in terms of how we can help people, so we're trying to deal with it in quite a holistic way. I think that's the way that it is with arts projects, and I think we're also aware that particularly not all the people we deal with are from a refugee background, but quite a few are. And so obviously, we're aware that for people who've experienced trauma in various ways and have, you know, ongoing trauma, it can be particularly hard to say, “right, I need some support in this point to do this, and then I'm off doing that”. Things can be much more complicated. In fact, I'm seeing someone today who wrote to me last week saying, “I'm sorry. I've been out of touch for a while. Things have been quite difficult. Could we speak again?” And one of the main roles, we always we say yes, we will speak again. It's not like I'm sorry, I'm too busy or kind of missed your chance. Absolutely doesn't work like that. And I feel that's right for all. Well, probably all kind of work, every kind of human interaction, ideally, but definitely for us in terms of work with artists and indeed organisations, that's a real point of, I say it's a point of principle. It's just a point of how we've always worked."

Code: Mental health and trauma solutions
P6, Producer

4.

"So, yeah, if they're not depressed, they'll benefit more or less. Um, we tend to find even the very, very depressed ones, if they were religious enough back home, will go to mosque. So mosque can be really, really helpful. And we've got good relationships with some of the local imams just around the emotional health and wellbeing of some of our kids who are doing nothing but are going to mosque. And they can be really, really supportive and helpful in getting those kids involved in their community activities. So that's good. Yeah, a lot of what they do is useful, even if they're not in college. There are community groups which will offer kind of, it's not the same, but it's more conversation classes and, you know, the kind of thing you do when you when you visit any country abroad and try to learn the language quickly. Um, so yeah, we'll try and get them, get them involved in things like that. We do, when we have their PEPs, we worry if a child is not doing anything outside of college. Just maybe not our British kids so much because they'll be out constantly with their mates and they don't

necessarily need an activity. But with a lot of our unaccompanied asylum-seeking children, they are either doing an activity or they're at home or they're being exploited. So, so we worry if they're not doing an activity basically. #00:23:49-9#"

Code: Mental health and trauma solutions
P16, Quality Assurance Teacher for post-16 education

5.

"We run a self-harm training program, which we developed with partners some years ago. We didn't particularly want to because we thought it should be a national responsibility because the gap was there and we asked repeatedly for the national college to do this. They didn't do it. We've now been running that for well over ten years called (name) program. And again, the model has a tiny team, is training trainers in other organisations and trying to sustain their networks. We've got a partnership with a lot of online reports if you're interested in, not particularly asylum seeker focused but with (name) University. So we now got, I think over a dozen self-harm trainers in a network that we've trained that they're now self-sufficient in self trainers. We've trained a network across all six education departments in self-harm and suicide prevention work with other partners as well. So it just gives you a flavour of the model that we're trying to grow skills and approaches and confidence to act in other agencies rather than doing all the sales, which just wouldn't make any sense. #00:18:21-2#"

Code: Mental health and trauma solutions
P20, Regional manager for health inequities

22. Trauma-informed practice

1.

"And people were really happy with the idea of individual conversations, interviews. But we haven't called them interviews. So throughout the study the co-design process showed that the term interview is quite traumatising because it sounds like a Home Office interview. So interview in the world of refugees in the UK means a Home Office interview. So when is your interview or mean when is the Home Office interrogating you about your right to remain? So we use the term conversations. So individual conversations with peers and focus groups with peer support workers have been part of the feasibility study. At the very beginning of the co-production process, we did conversations with people with lived experience and service providers, so that is how we gained the context for this work. The actual intervention has been run on those terms."

Code: Trauma-informed practice
P4, Senior researcher

2.

"Um, so our activities are very much focussed on trauma informed practice and um, and if we see somebody having a hard day, which is common, we will um, as always, it's not just in that case, but in every case, all the time. Give people lots of options. Um, if we're going for a walk, we might sort of see if there's a way of like not doing the whole thing. Um, is there a way of being, being part of the activity that requires doesn't require you to do much, but you can still be part of it. Um. Just options. Um. Checking. (Person 1: Yeah.) Do you want to be here and let me know? Um, yeah, I want to. Checking without kind of attaching yourself to a person because it's a group environment. Um. Checking in. Um, walking alongside. Um, that sort of thing."

Code: Trauma-informed practice
P15, Wellbeing manager

3.

"But it's not, it's not an environment for talking therapy. You might end up having really, um, meaningful big conversations, but that's not what the activity is for. There's, you know, um, but obviously, we are very aware that people react like people have a, carry a lot of trauma, and they, um, and they can react to certain situations in ways that you wouldn't expect. You know, they're kind of sort of, um, either really draw back or really kind of gets very protective, um, almost aggressive that like, and these are all just trauma reactions and responses that are coming from, um, that are just bubbling up from somewhere. Um, so we have a lot of understanding for behaviour that, um, might be triggered by something. Um however obviously we, we tend to know our participants from previous, um, already. Um, and, and if there's somebody who we feel is not able to, for safeguarding reasons and attend the group because we don't have the capacity to, um, care for them."

Code: Trauma-informed practice
P15, Wellbeing manager

4.

"So we've we've obviously all had as a team, um, some trauma led approach training, um, and how to deal with clients who have experienced trauma especially from conflict. Um, we've also um, been to the refugee buddy project for training, um, where they've delivered training on how to, um, understand and appreciate, um, people that have come over or fled, you know, the, um, countries that are in war or conflict. And, um, we've looked at different cultures and how to try and respect those cultures, but also support those people to, um, not necessarily integrate in our community because most of them want to go back home. If it's possible they want to return to where they come, you know, where they they lived before. Um, so, um, and I know a lot of our Ukrainian families have tried to go back home. So, um, what we've identified from our own experiences, from meeting with the children, young people, um, different levels of trauma and what their experiences are so obviously loss, witnessing, um, explosions and, you know, death. And um, seeing their towns flattened and things like that. So there's that like those extreme experiences. And we have come across a couple of young children that are mute, don't express their feelings. Um, so we're not trained therapists, um, or, you know, we don't work in in that way. We're social prescribers. So our roles are very light touch. Yeah, but we're absorbing, you know, what's happening to these children and young people. And we are trying to make the referrals. But with the social prescribing and the way that it was advertised from the beginning, parents didn't understand what that meant. So when you talk about health and well-being, they think that that's a financial thing. It doesn't translate. But when you talk about social prescribing, they feel that that's a health thing. And they're like, well, there's nothing physically wrong with me. So they don't identify mental health, emotional health and trauma as physical. You know, we try and encapsulate the whole thing. Well, you know, you can be unwell mentally and that's the same as if you've got a tummy bug or, cause we try and normalise mental health and, um, emotional health and well-being as is if you were poorly in other respects. But they don't they don't. There's no understanding there."

Code: Trauma-informed practice
P2, Youth service manager

5.

"I don't know how much in terms of trauma-informed practice and in terms of how they understand the role of trauma in how someone engages or doesn't engage or how, you know, for our social prescriber if people constantly miss appointments with her. (Person 1: Mhm.) And for me, if someone misses an appointment it's a flag for vulnerability. (Person 1: Yeah.) Usually I treat it in the same way I would if a child wasn't brought to an appointment. (Person 1: Yeah.) I'd look and see if they got care and support needs, language needs, got literacy needs doing have they got, you know, mental illness, what's going on for that person.

Do I need to proactively ring them? And on the whole I do, yeah. So Um, and then I don't punish them for missing an appointment with me. (Person 1: Yeah.) So, you know, and that all of that is kind of trying to develop that trauma informed approach, but I don't know. I mean, this social prescriber, she never rings if they've missed the appointment, I don't know what she does for an hour. (Person 1: Yeah.) So it's like how do you… I don't know. Yeah. I agree, I'd be really interested to know whether they have any training, particularly in engagement patterns."

Code: Trauma-informed practice
P14, GP and lead for local health stream initiative

6.

Person 2: "So, you know, so actually and I would say half the English language, you could communicate okay. Half you can't. So again you're dealing with both those things, so you have to make sure people aren't, well you don't have to do anything. But it's, it's sensible to make sure you're not excluding people with language. So it's having the different levels. And I'm a creative ESL teacher. That's part of the thing I did with the, um, when I was working with the teenagers. So again, using a lot of those creative ESL methods. So one thing that the first thing I did in the first ever session with balloons, with emotions on like, see, right, the, the word for an emotion with the balloon. And then people do that emotion and then you pass the emotions round. I did that last week with a new group I did. So there's some things that have stayed. And what I've done is I've changed that. Now, you know, when you do the contract at the beginning of the term, how we are going to work together, which is awful, boring, but you have to do it. Yeah. Um, and it's part of that, not a safe space because there is no safe space, but making a space that could be safer for everyone. We're going to try and make it safer together. So you, so you do with the emotion. And then I've thought because lots of the participants have been to lots of work, the arts workshops, and they know the answers. So it's like, what do we do when we work together? Oh, we trust, we respect, you know. And so actually what's that mean. So you do a statue. What is trust can be a statue of two people trusting each other. Give me a statue of two people respecting each other or listening or whatever. Then you put them on the balloon, the words. And then we try and pass all the balloons round. So you've got 15 women all passing the balloons and it's funny, haha. A lot of my work is about fun and funny.

Person 1: Yeah.

Person 2: Laughing. And then, uh, um, and then obviously some of the balloons drop, um, and they're, "Oh no, they've dropped and dropped". So then the learning point is, well, of course, when we're all together, of course some of those balloons will drop. Sometimes we might not be friendly or which balloon dropped. Oh the respect. Oh, maybe respect. We don't, but that's fine. We pick it up and we carry on. And so trying to show people, not tell them or help them experience the thing I'm trying to, we're trying to do. So that's an example of something that I did at the beginning. And it's slightly developed along the way."

Code: Trauma-informed practice
P9, Drama programme founder and producer/director

23. Enabling characteristics and benefits

1.

"Interesting. I hope everyone benefits. I think the people that self-refer tend to have, you know, an idea, um, of themselves. Sometimes it's a little bit more skewed towards employment. Uh, I'll come back to that. Um. I think the concept of meeting someone who can say to you, you're welcome. And now let's try and help you, um, you know, meet some

Irish people or meet some, um, get involved in an activity or get some help with your needs. Yeah, I think I think it benefits all groups."

Code: Enabling characteristics and benefits
P22, Social prescriber

2.

"So that's, I would say those are the kids who are the most likely to benefit if they, if we could get them to engage, but we can't get them to engage, mostly. So and time does quite a bit of that. Like time and boredom and often the visa process moving forward a little bit. There's quite a lot of that because a lot of those kids will get out of bed only for their visa meetings and interviews, and if they get the right to remain, they sort of breathe a sigh of relief. And sometimes then they will get out of bed and engage with some things and consider going to college. So I would say the kids who benefit the most are the kids with leave to remain because they are less panicked and frantic, and the kids who, um managing their trauma better."

Code: Enabling characteristics and benefits
P16, Quality Assurance Teacher for post-16 education

3.

"Yeah, I think definitely I think, um, often people would come in groups. So, you know, that obviously means that if you're on your own, you might not use, either you might not be familiar with the service or you, you know, it's a bit intimidating going to a random van outside of a pretty horrible place. So, yeah, you'd have to know about the service and kind of go there. Um, so I think definitely that, though it wasn't particularly advertised on site. So yeah. And I think also, uh, like recognising that you need to access the services as well. But yeah, some people will definitely benefit more than others or be more likely to go than others."

Code: Enabling characteristics and benefits
P21, Advocacy manager and primary care lead

4.

"But. Oh, that's a very interesting question too. So do certain types of people benefit from it more, um, I mean, the people who have seen. Um, we have people who participate in so many different ways. We have people and we totally accept people who come and they're quiet and, uh, and there's somebody who's trying to lead an activity and they are having a fag in the corner. Um, but it might have been a really big thing for them to even turn up."

Code: Enabling characteristics and benefits
P15, Wellbeing manager

5.

"Mhm. Um hmm. Oh yeah that is one of the questions. Um, who benefit the most? Uh, that's quite a difficult question. Um, because (...) sometimes I would feel like, God, I've done absolutely nothing for this person. Um, and I, I would feel like, you know, so useless and helpless in that situation. And then they would come back months later and tell me, like, just how helpful that one session had been, even though I did absolutely, well, to me, it felt like absolutely nothing. But I guess, you know, looking back on it, I was actually acting as a therapist, I suppose, you know, even though that wasn't the job. I was just someone who was there to listen, um, and I think that really is a part of the job often."

Code: Enabling characteristics and benefits
P1, Social Prescriber

24. Activity tailouring

1.

"It's harder for girls. Almost all of the community groups because of the numbers, and it is perfectly reasonable that there are more kids who are going to take it up. So that's what you'd put on. But most of the community groups are maybe not necessarily for boys, but they're football groups or rugby groups or cricket or baseball, they are very, very sport centred and a lot of our unaccompanied girls aren't particularly interested. Um, which sounds stereotypical, but it is just true as well. Um, and they also have, our girls have less community because there are fewer girls. So that can mean that they're really tight knit sometimes, but it also means that there are fewer of them around. It does mean that, our, our girls are more likely to be with carers than in residential placements, because I don't like residential placements generally, I think all of our kids, or at least the vast majority of our kids, should have foster carers who can take care of them individually, but our unaccompanied asylum seeking kids much prefer the residential placements because there are so many other people with their kind of culture, language, background, experiences present. So I think it's probably good for outcomes that our girls have foster carers because our kids with foster carers do better. But on the other hand, they're missing out in that community that the boys say they really value. So yeah. And so it's just quite a lot harder."

Code: Activity tailouring
P16, Quality Assurance Teacher for post-16 education

2.

"Um, we've provided things like laptops, uh, creative tablets. Um, we've worked with a few SEN young people, and we've also had, um, some children with, um, kind of long term health issues and disability. So we've paid for things like therapeutic pool sessions, you know, like a heated pool with a 1 to 1 tutor for therapy."

Code: Activity tailouring
P2, Youth service manager

3.

"And quite often, you know, we give bikes to, to whole families, to mum and dad and, and, and children as well. And also to see more, more children. Uh, yeah. Interestingly, it's worth mentioning just before I forget. I remember when the first couple of years of the project we did quite often, more often than not, you know, it was always in terms of families, it was always dad that contacted us and dad was looking for the way, you know. Yeah, maybe just or maybe for himself and the kids. And we would always naturally ask would mum, you know, would mum like a break as well. And then and dad you know would, would, would often speak on behalf of mum and say no, no, no mum's okay. You know, it's just I'm just looking for a bike, you know, just for me, you know, so we would always make a point to say, well, if mum changes her mind, you know, she'd be welcome to get in touch and we'd be we'd be happy to give mum a bike as well. Uh, and that, that started to change. So sort of through time, you know, after, after a couple of years and we've often reflected enough, you know, so maybe why, why it changed. But it's, no, it's not unusual where dad contacts us or even actually mum contacts us now, you know, looking for a bike for yourself and for, and for the kids. Uh, or we get, uh, a lot of, a lot of single, single women contacts as well looking to access bikes. So we have we have introduced lots of women to cycling for the first time, uh, of all ages."

Code: Activity tailouring
P11, Founder and CEO

4.

"So that's all about, um, poetry is a different form of, um, asylum testimony. Like how to, how do people tell their stories through a poem? And why is that better than the way the Home Office do it? So it's against that bureaucratic violence. Um, so that was a big strand. And we still do that. We still do the poems and that's great. Like an Eritrean woman who can't speak any English. She has a headache because she's so stressed. So her poem is just

like the word headache 20 times. And then the headache. And it's actually really when you perform that on the stage, it's really powerful.

Person 1: It's very moving. I can imagine that I can, yeah.

Person 2: It's like that repetition of the you know, it's like you get the... So again, it's the word echoing that feeling of like being driven, beaten down, you know. Um, so, so it's a practical, um, uh, method would you call it or response to the, the, the language challenges, but it also is effective aesthetically or the message would say, so those are the moments I'm looking for or I'm trying to find. Uh, and I suppose that's what my thesis is about. It's like all the, the different things we did and why they worked or didn't work, and, and it might be useful for someone, you know."

Code: Activity tailouring
P9, Drama programme founder and producer/director

5.

"I mean, right now, actually, um, and just it all goes back to funding. We actually did receive some funding specifically to support Ukrainians. And through that we ran a class that was, it was, most of our classes are mixed nationality, but we do have a class specifically for Ukrainians. And it's still even though the funding has finished, we still have running it or running the ESOL class in particular. So I mean there's definitely arguments to be made about that. Um, the fact that the majority of our students are Afghan, I mean, we have um, in some cases that's the design, whether you.. like, intentional or not. I mean, it's certainly we might look into doing in the future, but is challenging because like, um, we can't we don't want to like exclude people. And we also want people to like, not stay in their own, like, little country cliques if that makes sense.

Code: Activity tailouring
P8, Integration manager

25. Delivery model

1.

"And what does a day look like? So what we do when I see someone is, when someone is referred to me, I see them either at a GP practice or I call them if they can't come to the GP practice or if they prefer a telephone appointment. And we go through a holistic assessment. So we start talking about all different areas of their life essentially, it's very, very holistic. So we start with housing, we move on to uh, finances, benefits, employment, uh, family, community connections and anything really. And mental health, physical health, children’s safety. We always have to keep an eye out for any safeguarding concerns as well. So we just go through all these categories just having a chat with them. Um, very, very humane, trauma informed, type of approach depending. I mean depends on who the social prescribing person is. But this is how I do my job, because of my background as well. But that's the that's what we do. We do a holistic assessment. And then from that, as you can imagine, all sorts of things come out, that they were not expecting to share, maybe just because it's a safe space that the primary goal of the session is really to offer a safe space that they so that they can talk through anything that's really causing the stress or the, um, distress, for which they've reached out to the medical professional in the first place. So we look at all the social determinants around the health problem that they've been complaining about, which usually involves mental health, stress, anxiety, PTSD with migrant populations. It's, it's pretty much always, not always, but very often PTSD initial. And yeah, so that's what a session looks like. So that's what a first session would look like. And then we offer up to six or uh we could offer more sessions. But the basic sort of service is six sessions. And then when someone when you feel someone's benefiting from it then we can extend it."

Code: Delivery model
P7, Social prescriber

2.

"(This region) Health Board has got six local authorities, six health and social care partnerships, um, slightly nightmarish if it comes to the dynamics of all of that. So my tiny team tries to support six health and social care partnerships and the alcohol and drug side. You've got this thing called alcohol and drug partnerships, ADPs. So we can try to support six of those, um, currently with a maternity leave and a vacancy, we've got a team of essentially two people, and they've got a team covering every subject, the whole age range, plus a bit of my time. So that gives you a flavour of the complexity. So rather than just describing you a problem the only way we can really help is to operate, scale wise, is to work through networks of other colleagues. So we create, we've got a number of regular network meetings. We have an alcohol and drug prevention network for the whole of (region) group applied, which meets roughly quarterly with some extra things that we do from time to time. We do a lot of communication mail outs, we create a lot of resources and so on. And, and we work through local health improvement teams and other partnerships in the local patch. So trying to add value to what they're doing rather than trying to go out and do lots of community based things, which is, what is to some extent, I'm less involved these days directly in working with individual community projects. We don't have the means of doing that, with a few exceptions."

Code: Delivery model
P20, Regional manager for health inequities

3.

"So like the the six meetings, the one to one, the kind of casework support load, um, and then out of that would come activities signposting, you know, all those sorts of things, um, that come out of the support for social prescribing. Um, but we quickly realised that, that it wouldn't work in that way. Um, um, you know, the families weren't very responsive to that kind of working. They just wanted to get, you know, smiles on their the faces of their children again and get them into activities as soon as possible. So it was a case of we would have one meeting, find an activity for them, put them in that activity and then kind of monitor their journey. And that's kind of how we've been working ever since really."

Code: Delivery model
P2, Youth service manager

4.

"Um, and the there are like criteria about who we're allowed to see or who we're not allowed to see, like I said before, in terms of who was supposed to be seeing, it's generally supposed to be people with like, long term, um, challenges that the doctors are like struggling to, I guess like find like work out how to help them. Um. But the reality is, like I say, sometimes it's more like pressing issues and or more like social issues. Um. It's very much not supposed to be a crisis service. So the waiting list often it would take maybe like five, six weeks before, so if a GP wanted to refer someone, then the next appointment probably be 5 or 6 weeks down the line. So definitely not supporting people in crisis, um, and not supposed to be. Um and regardless of the weight, um, we shouldn't be supporting people, where, um, there's like a high level of distress or, like, really problems that need to be supported by secondary care or something like that. Um, I say that and yet often I would get referrals from people who really needed extra support beyond what I can offer, um, yes."

Code: Delivery model
P1, Social Prescriber

5.

"So, anyways, so, you know, our classes are free. Um, we have a number of teachers, I don't actually teach itself, I don't have a qualification, but I have a number of teachers. Um, and actually very recently, I mean, I'm the integration manager, I recently brought on one of my existing ESL teachers to be a coordinator in the project. So she does a lot of the day to day logistics so I can work on other things. And yeah, so we have um, yeah, in person classes. Um, with often I would say up to maybe 15, 20 students. It can sometimes be less than that though, and especially for the Pre-entry entry one level, we tried it, so it doesn't get too big. We try to have like co-teachers or teaching assistants in some cases."

Code: Delivery model
P8, Integration manager

26. Recruitment and referral

1.

"I wasn't expecting as much support from statutory professionals, due to time-constraints and burnout. Some would even accompany folk to the sessions and arrange for interpreters to attend with them. This has ebbed and flowed over the years. I would say we have many allies across (city) that have helped our project to thrive, mainly through referrals and word of mouth."

Code: Recruitment and referral
P23, Volunteer

2.

"Um, I get my referrals from, you know, the, the primary health care team, doctors, ots, physiotherapists, um the (name) hospital maternity unit, psychiatric. Um, so a lot of different primary health care teams. Um, but I also get referrals. Not very many. I would like to get more from, uh, managers of reception of the centres. And there is one role in particular that I'm, you know, that has been very useful, but it isn't in every direct revision or I call centre, it is called a reception officer. Um, it is um. It's part of the whole aim to have the IPAS centres and monitored and reviewed by what we call the health, um Health and Quality Organisation, which monitors um hospitals and nursing homes. So that role has been very useful from one IPAS centre, because the role isn't in every one. So that that has been a very useful link. And then, um, I also have clients who have been living in (country) for 15 and more years who are still, you know, looking to integrate, still looking for help, um, to be included and integrators and just having some of that difficulty. Still having difficulty with the English language. Uh, so."

Code: Recruitment and referral
P22, Social prescriber

3.

"Um, and I see people that are referred to me because they have gone to their doctor and they've had an assessment with their doctor and the GP, or the nurse has realised that there is an underlying social concern behind the health concern that they've come to see them with. Um, a lot of the times that they are patients that are on the frequent attender list, which means they are in most cases is because they're extremely isolated and their only human contact in many occasions is through the health care system. Um, and a lot of these people are immigrants, migrants who are no longer scared. So there's two categories. So in this or in the social prescribing model within the health care system can be not amazing sometimes. Um many migrants if they are hiding, if they are at a state where they cannot make an application, they will be terrified to go to. They have the right to go to a GP practice. They don't have to pay anything and they don't have to have a visa. But they're terrified because they think they're going to contact the Home Office, which I mean, it is, it's a justified fear because it has happened in the past. Not that GP practices have, um, called the

Home Office, but all the services do contact the Home Office, like social services will have a direct link to the Home Office and they will tell them when they encounter someone that has no status and that could trigger removal procedures for deportation. So that's why migrants, um, of certain categories might not approach GPs, which means there's a risk to their health. Of course, also, they just will not access a service like social prescribing, which could link them up with other support services that they are entitled to. And they you know. So yeah. Who do I see? So that's, that's who I see. And what does a day look like? So what we do when I see someone is, when someone is referred to me, I see them either at a GP practice or I call them if they can't come to the GP practice or if they prefer a telephone appointment"

Code: Recruitment and referral
P7, Social prescriber

4.

"So obviously because we were seeing people on a one-off kind of stealth basis, we had to refer back to the onsite medical team, but also make safeguarding referrals and also make raise concerns about suitability for the site. And so the Home Office has suitability criteria. So technically only people who, who fit that suitability criteria should be housed at (town). Um, so but that we found that that wasn't the case. And really, if they were applying that criteria correctly then most people shouldn't that are in (town) shouldn't be there. Um, because it's about like, um, uh, serious health needs, mental health. Um, a lot of the people wouldn't fit that criteria anyway, um, histories of torture and things that they shouldn't. According to the Home Office's own criteria, they shouldn't be housed there. And but we found a lot of people that were accommodated there shouldn't have been. So kind of raising that was difficult. The on-site medical centre didn't do suitability checks when they should be doing it. They also, um, challenged the Home Office, challenged their um, allocation of accommodation policy to kind of request a higher burden of evidence if people were requesting to be moved. Um, which meant that often the evidence said we would write a letter. So the clinician, um, in the service would write a letter outlining the concerns and why this person isn't suitable to the site. That meant that they needed kind of more evidence than that. So I think it's two GPs or yeah, additional evidence, which meant that it became difficult for us to be able to advocate for people to be moved from the site. Um, so yeah, the medical team didn't engage with that as an issue. Um, they just really, you know, we heard a lot from people as well visiting the service that they'd just be given sleeping tablets or antidepressants like short term solutions, routine prescriptions, everyone kind of having paracetamol, sleeping tablets, antidepressants, um, without any kind of long term, um stuff. Um, and particularly with safeguarding it was an issue would refer we'd make a lot of safeguarding referrals but then um through the accommodation provider but then not hear anything back. And it took a long time to kind of build those processes and make sure that they were, um, being taken seriously and acted on."

Code: Recruitment and referral
P21, Advocacy manager and primary care lead

5.

"Yeah, I think that's actually… I've got a list here. I think that's it's just a lot of networking, a lot of signposting. Um, a lot of meeting up schools and colleges, um, and a lot of rolling things out. Like I said, we had this Syrian theatre thing that went to four schools. Um, and then funding was obviously a big aspect. Um, but just meeting lots of organisations too. And I got a meeting, (city) council later to speak about needs across the city that they've seen in different schools, how we can meet them tomorrow. I'm seeing the uniform bank in (town) who give out free school uniforms. Um, and how we can get them more, better known in in schools. Um, a lot of services and charities. Somebody when I got the job, somebody said you're a bit like the sand in the cracks. #00:23:36-3#"

Code: Recruitment and referral
P19, Schools coordinator

6.

"I'm imagining a situation where I was to send off a client to, um, but there's an organisation, I think they're linked to link to lots of health services, and you come to them from services and they, uh, (charity name in city). And they, um, I think must be like, partially council funded, almost all of it. And when they run things like kind of therapeutic, um, allotment and um, so like supported gardening kind of group. Um and say I was to send refer somebody there… How do I ensure that the other participants aren't casually racist? (Person 1: Mhm) Um. How do I ensure that the when I'm assuming that, uh, um, because I've worked in this field for a long time, I've probably, you know, a couple of the people, um, leading the sessions. But, um, how do I ensure that the facilitators aren't, um, casually racist or they are, they understand the needs of the person who I'm sending to them. Um, I mean, I feel like I must have a certain amount of responsibility to, to, um, introduce this person to the group and to just get a sense of, of how this, whether it works and I think in I, I mean my way of doing it basically is that I utilise volunteers and this is where my befriending work comes in. Um, that I think, I think is sort of false, often irresponsible to send somebody alone, um, if the person is a client and they are vulnerable. Um, I also think it's just it's just silly because nobody ever goes. Yeah, there's no point. I get to maybe tick the box that I refer to somebody somewhere, but they're never going to attend. (Person 1: Yeah.) There's, there's zero outcome from a one-sided referral. (Person 1: Yeah.) Um, because they will never, ever actually take it on unless it's organised, unless I attend it with them, unless there is a volunteer who has gotten to know this person, and they've built a level of trust, it's, basically it's about trust. All of it. It's a trust building exercise. Um working with vulnerable people is, is one big trust building exercise. And do they trust me? That is the bottom line. If they trust me, then they might trust that the place I'm sending them to is good. So they might even give it a go. Um, but that's quite a big step for me. What they, the only way I can guarantee that people will turn up to anything, and like in the years that I've been working there, the only way people have turned up to anything is anything kind of outside of, um, this is, unless they, there is like a personal connection or they know guy who already goes, it needs to be "I will meet you at this place at this time. We will go together.""

Code: Recruitment and referral
P15, Wellbeing manager

7.

"So, um, sometimes it can feel a little bit like a dumping ground for the GP. Um, if you're just not too sure, they'll, they'll see a patient and they might say, "oh, gosh, you know, I don't know what to do. Go speak to the social prescriber" and then we'll get it and we'll kind of go, "oh gosh, what do we do here.""

Code: Recruitment and referral
P17, Social prescriber

27. Methodology for project

1.

"It's the seed approach where you scatter your seeds. And I think over the last three years I've been working out what my seeds are. And also there's more organisations that know about us that can signpost people our way to the council, signpost people away, and that's the kind of bottom up. Um, so I think I'd go into (city) with my seeds and, and get to know the council and various key charities first. Um, and then, yeah, approach schools through the seed of sanctuary. #00:55:40-2#

Person 1: What do you think your seeds are? Sorry. Because you've said you've been figuring out what they are, what these are. #00:55:45-1#

Person 2: Um, I lived so that when I'm thinking about seeds, I'm thinking of the things that schools are like, “Yes, I want that immediately. Um, rather than please do this event that we've put on”. Um, it's people with lived experience who are sharing their story. Um, and that has been the trickiest seed to, um, germinate, create in the analogy."

Code: Methodology for project
P19, Schools coordinator

2.

"Um, it's again, it's the beauty of social prescribing is that we don't necessarily just cut off that support. We will always say to someone, um, we're being quite open and honest, we don't feel like you're engaging or, you know, they might just not go back and contact. But whenever you are ready, you can get back in contact. We always let a client know that we do require them to be proactive. Um, so I tend to say, you know, if it's just opening links or letters that I send over to you, that's a good enough start and we can build up from there just so they don't feel the pressure and get automatically put off by what the social prescribing kind of wants them to do."

Code: Methodology for project
P17, Social prescriber

3.

"Listen, okay. I don't know if you're familiar with the human givens. Um, Ivan Tyrrell, um, who wrote a book set up, um, a college, which produced the magazine and trained practitioners in the human givens approach. And I think that's a very helpful construct because it assumes that as humans, we have already got what we need, for health, wellbeing, productivity, and that when something goes awry, often it is that we need to recognise and work with that. I’m not talking about, you know, major head injury or, you know, losing a leg or something like that, you know, but it’s like, it’s like, it’s like, let’s start from the basis that we are from, that’s not a deficit basis. You know, we are all different. We are each of us uniquely capable of flourishing. You know Robin Wall Kimmerera? Her latest book, just at the front, it says all flourishing is mutual. That’s what I absolutely believe, which is that if we fulfil what I call that moral ambition, if we do what Audre Lorde talks about us finding our work and doing it, then not only is the world around us a better place, but that we are, we are likely to grow and thrive and be, you know, increase our own vitality. So if you start from that place, which is that vitality is about recognising the value of life itself and wanting to enhance it, then you kind of, it commits you to certain kinds of actions. Which is not comfort seeking. Which is not acquisition. It is, it is about being the best, having the best quality relationships we can with each other, with the planet and with ourselves."

Code: Methodology for project
P12, Founder and research coordinator

4.

"The talk I’m giving in Glasgow on Tuesday, we use seeds. The seed, the seeds are the promise of life and they are the gifts of nature, you know. Take pumpkin seeds for my allotment. And, you know, one pumpkin produces about 200 seeds. And that promise of life will only be realised in relationship with us. You know, humans who are stewards of the land. So, you know, you have to, you have to nurture it, you have to grow it. It's a bit like at (charity) you have to nurture students. You have to nurture refugees. You know, a bit. Just enough so that life can grow and thrive and then be passed on elsewhere. The work in Uganda is with (charity name), an organisation that trains subsistence farmers in organic methods. And then they pass that on to their neighbours, and their neighbours, and their neighbours. So it’s that growth economy, that’s not about financial growth. So that’s the, the kind of my understanding."

Code: Methodology for project
P12, Founder and research coordinator

5.

"And now through (charity). I've always thought this way. I've always looked for if something happened, I've always thought, well, well that's good, because. I can't think "Oh no that's terrible. My life is ruined." I'm not seeing my life ruined by circumstances, you know. Instead, it's "Okay. Okay. What can we do with this?" That's, that's been the way I've always worked is look for opportunity, look for possibility. And I've always tried to do that with other people because I know I can't do anything by myself, apart from my allotment at the moment. It's that kind of, it's collective you know, the best results come from working with other people, people who know most about the problem, you know. You know, students know most about living in this modern world. I don't. Bewildering, frankly. Refugees, they know most about managing in really hostile circumstances. Why would we not work alongside them to try and make it better? And that's, that's the strengths approach. It is that, it's the constant, it's the refusal to lose hope, not just optimistic, because optimism is like "Oh yeah, you'll be alright." It's the Rebecca Solnit, you know, because life's not a lottery ticket. It's an axe that you have to break down the door with, because it's easy to go "Oh there's nothing I can do". But the strengths approach, as a student once said to me, there's always something you can do."

Code: Methodology for project
P12, Founder and research coordinator

6.

Person 2: "So we started we did drama for life, which was one class you could go to, and drama for performance, which was the other class.

Person 1: Okay. Yeah, yeah.

Person 2: So that's kind of the model of, that's kind of the model I sort of proposed in my PhD, actually. This is what I found actually, if you were going to set it up from scratch.

Person 1: Yeah.

Person 2: Yeah, but they're not like hierarchical. They sit next to each other because sometimes someone from drama, from performance might be, having got some bad news, having a rough time. They can bounce back to drama for life. And a lot of the techniques and games we play in, the methods and everything are interchangeable. You know, it's not like different. It's just, yeah, the drama for performance is higher stakes, public facing, you know, so a bit more resilience required. Drama for life, actually, it's fun. Actually, if you miss three sessions a term it's not too, it's not the end of the world. Just let me know. You know, it's, it's a softer, more forgiving, forgiving. It's all forgiving. Hopefully. So yes, that would be my answer. And, uh, it's quite hard to do practically because certain women are only available because the two sessions are two hours, then there's a half hour break and then the other two hours. And of course, some of the women from drama performance could only go in the morning because they've got to pick their kids up from school. Childcare is a major issue, um, It's all the time. And another thing about performances. Um. And everything."

Code: Methodology for project
P9, Drama programme founder and producer/director

28. Activities delivered

1.

"An abundance of activities, included but not limited to:

Planting and tending organic produce
Watering

Weeding
Tree grafting
Saving seeds
Planting fruit bushes
Nurturing and tending our bespoke forest garden
Nature art
Forest school activities
Campfire cooking
Cooking and sharing food from different cultures
Green woodworking
Sowing seeds
Foraging
Using herbs for health
Making elderflower cordial
Making rosehip syrup
Making firecider
Building/fixing raised beds
Chopping wood
Wreath making
Creating a sunflower circle
Finding creative ways to label veg
Apple pressing using a traditional apple press
Meditation
Gentle movement"

Code: Activities delivered
P23, Volunteer

2.

"So we had group work for single moms. We had a group of moms who came every Tuesday to have, um, lunch together and do activities. They had a choir to sing together, and then there was a creche for the children. And then while they were in the group, they might also have a brief meeting with their legal advisor at the same time while they were there, because the travel expenses were, were covered for the day, because they were attending the group. So I would be like, give me the paperwork, so it was very holistic… which is beautiful. And I think that's the key for any project."

Code: Activities delivered
P7, Social prescriber

3.

"Uh, these days we are uh, uh, big in need for uh, uh, advice in term of the immigration or other general advice, housing, welfare. So these things are now as a priority for the organisation to get these people out of the issue or for our elderly, uh, generation to take them out of isolation. Uh, other issue that to support them in their daily life or paperwork, so other things. So these are the priority"

Code: Activities delivered
P5, Charity director

4.

"Um, then we've got um, uh, in terms of wellbeing activities, we go out every week and one day a week on Mondays until we take a group out and, um, and that's a changing, ever changing group of people of depending kind of who turns up and, and the activities range depending on our partners and the time of the year. Um, so that might be, it might be a walk, it might be a cycling trip, it might be the canoeing trip, very excitingly, um, and the summer, it might be a train trip to a sort of outside (city), to a village, um, with partners who were

running a workshop, you know, sort of nature based workshops with and, um, etc.. Yeah, it might sometimes in the summer, um, sort of in the winter when it's raining and sometimes we might go, we've been, gone to the cinema... but yeah, it's, um, social group activity, essentially."

Code: Activities delivered
P15, Wellbeing manager

5.

"Uh, yeah. I mean, I mean, we, within the hotel, we, one of the things that we do offer there is a led, a cycle, cycle group, cycle rides. Actually, we just commissioned, you know, someone to do that once, once a week. Actually, just came to the end because the funding came to the end. But we're trying to put, I'm trying to put some new funds in place so we can re-establish that again, you know. But that was because lots of people within the hotel, you know, were saying, you know, they had access to all these new shiny bikes. Yeah, a lot of people were saying, I don't know how to cycle. I don't have the confidence to cycle. You know, uh, I'm, I'm not used to cycling on, on, you know, the roads and (city), on this side of the road. So we've tried to respond, we've tried to respond to that. That's really the first thing that we've done because unless somebody asks us specifically, you know, somebody chased us saying I don't know how to ride a bike. Then we'll do what we can, you know, to teach that person. But actually, you know, we have very, very little time and resources to do that. Because if we're doing that, they were not fixing not bikes and working through a waiting list. So we need to find, you know, so we, a lot of what we do might be signposting."

Code: Activities delivered
P11, Founder and CEO

29. Stakeholders involved

1.

"We've got about 300 activity providers on our books. Cross County. Um, and we started off with, uh, quite a large pot of money for each client. Um, we managed to get around £1,000 for some of our clients for the year, which was great. As the numbers got bigger and bigger last year. We had to reduce that down to, I think around 600, and then next year it's going to be even less because there's a smaller pot of money now. So we've got to look at how we do it. Um, but we've placed children and young people into activities, um, such as horse riding, tennis, swimming, uh, afterschool clubs, um, uh, all sorts of arts projects, residential forest schools, um, circus schools, uh, gymnastics, dance, drama, you name it. Like, you know. All the sports."

Code: Stakeholders involved
P2, Youth service manager

2.

"It's all pretty random, actually. I mean, I as I said, I met (name) at a dance class our daughters were both attending. Um, she then introduced me to a woman who runs another theatre company called (theatre company). Yeah, I another very talented singer songwriter composer, um, who worked with us on this lost and found musical narrative, came because she I think she applied for the deputy job once, and she was quite clearly way, you know, qualified in all the wrong ways for this job. But she was so engaging and so creative and so talented that we just met up afterwards. And so she that sort of relationship came out of that. There was another one who just happened to be somebody that I knew did some work in the neighbourhood and we, you know, so sometimes it's, it isn’t planned. It's not at all systematic, how these things happen."

Code: Stakeholders involved
P10, Writing project coordinator

30. Volunteers

1.

"But the, the way we survive and, uh, by working, I mean, there was a time, there was a time that we had no funding at all, but we didn't. We committed to, uh, serve the community as volunteers. And we, we recruited more volunteers, and, uh, we survive those times as well. And now, uh, we are as an organisation as such, uh, right now., so that if I cannot say we're a big organisation, but a middle class or middle, uh, stage organisation that supporting, uh, un uh, uh, serving, uh, thousands of community members every year. Uh, so, uh, the, the funding, uh, are still a challenging, uh, time for the organisation because some of the funds are continuing to supporting but their reducing the funding. Uh, so we try to, like I said, instead of full time working with a part time, uh, staff, uh, some of the work we doing as well, using our volunteers to do, uh, some of the activities, uh, being donated by the community members, such as, for example, such as the Saturday schools. Uh, the parents are supporting some or part of the, uh, expenses, like the paying to the teacher or, uh, pay for the hiring, the classes. Uh, so when we get the funding, we ask them not to support, we saying, oh, we have something to, now to cover, But some time is coming that we ask them that the funding is ending, so until we get a new one, so they are supporting and they keep running the classes."

Code: Volunteers
P5, Charity director

2.

"But on the, on the on the other hand, we have four community English classes that are run by volunteers every week. (Person 1: Mhm.) Um, so almost every day, every working day there's um, there's an English class. Um, and they are, they are depending on volunteers. They can be very social. They can be sometimes more about like they are not um, graded class. So you don't need to take any level exams to come in and you just walk in. Um, and, and sometimes they seem to be some teachers are more about eating cake than, than, than much else and um, uh, depending on the teacher. But um, but that's been going on kind of solidly for a long time."

Code: Volunteers
P15, Wellbeing manager

3.

"Dad was a uh a police captain in the arm and he had two houses. Definitely was not coming here for, for the, for the privilege of more money. Anyway he then made sure very early on he would learn the language. But then when he used to come to us, he would volunteer. So he would sit in a coffee morning and do a little bit of interpretation with people that were just coming for the coffee mornings that needed somebody to talk to."

Code: Volunteers
P18, CEO

4.

"I think that, um, especially hard for refugees, they have because of the way that they work, they, they have different services that they provide. And therefore there is a lot more opportunity for volunteering. So they get to clean the beaches, for example, and they make a really big thing to say, you know what? They're actually contributing to our society. They're not plundering our society. They're actually contributing. And no, they're not taking away from you."

Code: Volunteers
P18, CEO

5.

"This was during lockdown, and the tutor asked if I would do 1 to 1 with her because she needed more help. So I as I say, I've worked with her for a long time, built up a relationship. Um, at Language Cafe, she was still quite shy. Um, but she has become more vocal over time, which has been lovely. And she also, um, I worked with another volunteer to do her speaking for the B1 test, the, um, citizen test. So I, for her to have the confidence to work with somebody else, um, was quite, quite big really. So yeah, that's one story of just her journey from being quite shy and unconfident to much more confident. Um, I think obviously the, the, the families I'm working with at the moment who have no English and very little opportunity. Yeah. Um, you know, so I think that's very important for them to not only have the English language, but actually to have a known face that they can come and ask questions of."

Code: Volunteers
P13, Charity chair

6.

"Um, so I think, I mean, one of the other examples is that I have observed is that there's an organisation that's sort of related to transition. It's actually the same woman who was founded. The transition group started this other thing called (project name), which works with people in the southern part of the (city), and they are people who are what's described, I think generally as hard to reach. You know, they're people who don't tend to volunteer for community organisations. They're not even very visible necessarily. A lot of them are women, um, of Bangladeshi heritage or South Asian heritage. And they tend to stay at home and be very self-effacing. And often they don't have very good English. But my God, they can cook, you know, so and, and many of them want to so, so I mean that that organisation started a kind of shared meal thing with these women and they would start to cook for other people in the, in the estate where they lived. They would, they use the um tenants and residents of (housing association) and every Thursday they would have a meal, and then that expanded into, you know, it turns out that some of them could sew, so they would do they bring a little bit of children's dresses that they were making or they would do mending or they would do repairs for people. And then there was another estate for also in the south of the (city) um where another um charity that works with women, mostly from that um cultural heritage planted a community garden. And so then they discovered that, you know, many of these women were very, very skilled gardeners. And also, obviously, it was enormously, an enormous pleasure and delight for them to be able to realise that they could actually grow the vegetables, that they couldn't find the market here that they remembered from home, you know, so they were growing goods and vegetables, various other things that they needed for their cooking. Um, and so that's, what does that do? First of all, it brings them together with each other. Secondly, it brings them together with people in, you know, the native English-speaking, as it were, community who probably can't cook particularly well or don't know how to repair a dress or but never grow vegetable in their lives. And the third and the most important thing which is related to that is it means that it gives them something to give. You know, because as a refugee, as an asylum seeker, you're, you’re always on the receiving end. You're always begging. You're always expected to be grateful. You’re always told that you're be lucky to be here. And you have to express that feeling of being terribly lucky. It's kind of wearing and, and it's annoying. And it must be humiliating, actually, on a very deep level. And so when people actually can say, here's this thing and I've made it for you and, you know, I'm giving it to you and somebody else has to be grateful to them for a change. That's an enormous thing, I think, to be able to offer people. So I would say that those, those are the examples I have observed where this kind of activity has been very beneficial to people who are refugees and migrants."

Code: Volunteers
P10, Writing project coordinator

7.

"think, probably it was, and then you get into all sorts of other annoying problems, which is that when we, when we do recruit mentors again, we try very, very hard to find people who are not middle aged, middle class and white. We almost never succeed because if they're young, um, and particularly they're young, minoritized, or from a culturally diverse background, they're probably not particularly financially stable. They probably can't afford to know to donate five hours every fortnight, and particularly not to know that they're going to be able to do that for the next two years, which is a sort of minimum level of commitment you need. So, in an ideal world with no other constraints, I would make sure that there was money to pay the writing facilitators so that they could be people from a different range of, uh, cultural backgrounds and ages, and so that they could do it as part of a job rather than relying on volunteers. I think that's the biggest change I would make, and I think that would make an enormous difference to how it felt. And it would, it would very much reduce that sort of slight sort of white saviour element, which I feel concerned about in what we do at the moment. I mean, it's never said, and people have never complained about it, but I think that inevitably there is a little tinge of that just because, as I said, we can't afford to pay people to, to, to do the volunteering."

Code: Volunteers
P10, Writing project coordinator

31. Partnerships in delivery

1.

"So we are the, uh, have that, uh, the leadership of uh, uh, running and working with other 11 to 12 Afghan organisations who are working differently in different (areas). So, uh, we are leading on that to bring all those community together, especially running the social and cultural event together. So these are the people, unfortunately, was not attending each other meetings, now working together under the leadership of the (charity). So that's that's, uh, bring a lot of advantage to the organisation as well that we've been capable of bringing all these 10 to 12 organisations. We have an event on 13th, uh, that uh, involving all those organisations as well, so every time we have such a event, uh, despite the lack of their participation of those organisations as well, we just introduce them as partners and they are so happy to get them involved. And, uh, they learn something from the event and then, uh, the, the credibility of the organisation, that can work with, uh, everybody if they are working for the community or the, for the community members. So we are ready to work with them as well. So, yeah. Uh, yeah. That association is another part, uh, within the (national organization), uh, the (NGO) group comes to the UK and I am the member of trustee there and that group. So about nearly 50 communities. We are working together within the community with the (national organization). And that is one member of trustee that I am a member of."

Code: Partnerships in delivery
P5, Charity director

2.

"But what we then put together for the counsellors was a series of site visits and project visits, which kind of took 1 or 2 weeks to organise. I organise along with the (area) Integration Network, which is the community response to the, the needs of asylum seekers in particularly the north of (city). And it came about, I think there was a contract many years ago where essentially, it's crude language, but some of these persons were simply dumped in really bad quality flats, lots of demolished, uh, in the north of the city notorious (name) flats, for example, were some of the worst housing in Europe at that time and were earmarked for demolition. They eventually were blown up. These 60s flats, of course, like many cities across the UK that were badly built and were deeply mouldy, leaking after a few years and became

crime hotspots, all the rest of it. Anyway, so after a period when, when very little work was done and these asylum seekers simply appeared in the community without any backup and support, rather than rejecting them, the community turned their anger, I think, against the authorities, but also then said, let's find out a better way to support these groups of folk and work with them. And so after several years they were quite mature. By the time we organised a site visit and really spent a couple of hours with them, um, and they got some public money, but most of it is their own fundraising and so on and, uh, tremendous amount of work. A lot of it is around trauma and distress that you might imagine. And the integration would really, was the watchword. They would bring together a children's project, so it wasn't just the children of asylum seekers or unaccompanied asylum seekers, they were the local kids could take part in these play schemes of various kinds of cross-cultural food and community events, arts events, poetry, you name it. Women's support projects and so on."

Code: Partnerships in delivery
P20, Regional manager for health inequities

3.

"And, and then the other thing that is really unique about our team at the moment is that we work incredibly closely with the um, mental health services. Um, and we've got the only asylum seeker and refugee mental health team, NHS mental health team, um, in the country. (Person 1: That's a big deal.) Yeah. Based here in (city) and, um, and we brought up one of the things that the activities, oh, it's not so much an activity service that we, we run um, it's called Health Point where for sort of half a day of week, um, our centre is taken over by health services and is run by volunteer GPs and mental health practitioners and um, and also, um, one of the, one that, a person who used to be in the activities team um, who has now qualified as a counsellor, has moved on to the um NHS mental health team, um, to work essentially with the same clients but in a clinical setting. Um, but he still comes and joins our activities one day a week, so he still sort of directly facilitates activities for, for our clients who often end up in his clients, um, and his patients as well."

Code: Partnerships in delivery
P15, Wellbeing manager

4.

"Um, and we're quite, um, straight with our partners now is that this is, you will accept any form of participation. Um, we, um, will not have anybody kind of be like, no, no, no, no phones down. Like, we need to sit down because this is about my workshop. Because it's not about the workshop. Um, uh, if you can show interest to people and engage people in conversations, fantastic. Do that. But, um, it's. Yeah. And we often, because there's often a lot of laughter and a lot of, like, jokiness and a lot of just fun. Um. But not everybody, like, directly participates in that."

Code: Partnerships in delivery
P15, Wellbeing manager

5.

Person 2: "It was really difficult. And I had an hour-long conversation with the, the, the the manager of the social services team. And we got on well in the most part, but I just wasn't. The young people were putting pressure on their workers and their social workers. They wanted to get these memberships. They wanted these bikes. I can't go to college if I haven't got a bike. All of this. And then her social workers were putting pressure on her. And then she was putting pressure on me. And I was explaining to her why. And then she was not having any of it. To them we were just putting a barrier up, to their support, what they could give to their, you know, their kids. But (name) was right with saying what he said because he's worked with refugees all of his life. He said he took the refugee out from like Ghana or somewhere, and they were cycling the wrong way down the A27 and not even using the cycle path. You know, no helmet, nothing and no awareness of how to be safe on the roads.

Um, you know, they come from a country where there wasn't proper roads, it was dirt tracks and, you know, so.

Person 2: And that that matters to under duty of care. Of course it does.

Person 1: 100 percent. And I'd say this to them and they'd be like, yeah, you know, well, that's our job. That's our job to keep an eye on their safety, not yours. And, you know, stuff like that."

Code: Partnerships in delivery
P2, Youth service manager

32. Staffing in delivery

1.

"It's really easy that our colleagues or our staff all, uh, know that, uh, which, uh, of our staff can help in which, which, uh, uh, direction. So if someone is coming from the women's group, the women's group just make a, uh, our immigration advice aware of that issue or give their details or ask for any appointment if is available, which they because he is working only two days a week. Uh, and other three days with the other firm, uh, legal firm. So we just hired him just for two days a week. Uh, uh, due to our financial circumstances. So if anything's happening like that, we know that, I mean, even the woman's group coming to immigration advice during his, uh, immigration issue and asking something that related to a women's group. So he direct the, the client to the woman group that the, uh, that I can help in this regard or, or to the education part that we can direct to education or anything over there, that they asking them to speak to us, uh, to something like general things."

Code: Staffing in delivery
P5, Charity director

2.

"The attachment issues our kids have, um, they're um, so if they're not depressed, usually they're quite enthusiastic. And usually they get on quite well with professionals. And will listen to professionals. And if professionals say, like there is this community group that you'd like, or there's this basketball team that you'd really like that, you'd find really fun, they'll give it a try."

Code: Staffing in delivery
P16, Quality Assurance Teacher for post-16 education

3.

"Well, um, we have some volunteers, but sometimes, honestly, even, um, we are struggling for the cost of, uh, (Person 1: Yeah.) these volunteers (Person 1: Yeah), uh, which is, uh, the funding. Uh, sometimes they have their own criteria, their own things they said. That's the problem. Sometimes we recruit from the sport. They say, okay, that's you our sport, that's our, um, happy, we need to do if, we don't have to wait for someone to help us. If nobody helps, so we need to help self. We we going to use them sometimes to, um, do volunteering work. (Person 1: Mhm) Um, but that's only for the sport things. We cannot ask them to support men to come to do something different project which is they are not happy with that. (Person 1: Yeah). Yeah, we can use those people even sometimes we cannot pay their expenses as well. They trying to um, I mean we're trying to bring them too and they spend their money."

Code: Staffing in delivery
P3, Youth and sport manager

4.

"They've employed me, um, as their worker for schools, and colleges now because I've adopted colleges, um, to do 20 hours a week. And we have a director as well who does 20 hours a week but that-, that's, that's it. We're very, very small."

Code: Staffing in delivery
P19, Schools coordinator

5.

"So all of our wellbeing team must work part time. Um, so we have, uh, we have a rota now where we, um, we sort of at the very core of our activities... but the thing is that this is kind of the way we see activity is often like the outdoor activities that we put a lot of extra effort into. But on the, on the on the other hand, we have four community English classes that are run by volunteers every week. (Person 1: Mhm.) Um, so almost every day, every working day there's um, there's an English class. Um, and they are, they are depending on volunteers"

Code: Staffing in delivery
P15, Wellbeing manager

6.

"And I think, um, I think that's much to do with the skill of facilitation we've got at the moment. (Person 1: Yeah.) Uh, Where we've got really skilled and very confident facilitators who, who know how to kind of throw a whole burst of energy into the room or into the group, um, make themselves be the silly person. Um, um, like if it's about trying something new, then you, you can be the first person to try and not necessarily try and fail and, and kind of make an example that anybody can try. Um, any outcome is okay. Uh, and then also people who have the skill of, like, then kind of step forward and step back where you kind of step forward and juggle balls in the air and get people talking and involved. And then, then you sort of step back and let people take ownership of the situation or carry on. Um, but there's, there's a real, um, art to that. And I think, um, I think we've got a team of three people who can all do that very well."

Code: Staffing in delivery
P15, Wellbeing manager

7.

"We have a Ukraine advisory panel and we meet each week and it's hosted by the county council and all the professionals that are working with the Ukrainian um visitors, um, and Ukrainian people come together and they select members on the panel. And then there's visitors to the panel. Yeah. And, uh, I normally attend or the team normally attends each week, and then we get feedback stuff from the, um, from the Ukrainian panel. And they let us know how we should be doing our jobs basically for them, you know, right. So that's another way of, um, learning about the different cultures and breaking barriers."

Code: Staffing in delivery
P2, Youth service manager

33. Priorities if starting new project

1.

"So how I would design a project, I would make sure that the priorities are in the right place. So I think sometimes we create systems, we create projects to help people, but actually the priorities are not the right ones. So that's what I think is wrong with the world. Like we are, we're all like, oh yeah, climate change and mental health crisis and isolation crisis and food crisis and all these crises. And we're like trying to take action or try to change those things through politics and voting and activism and the like. No, but the, the priorities set by those who have the power to change things are not the right ones. If the welfare of everyone and the survival of all human beings and the human race as a civilisation was the priority, this

would not be the system. So it's really again, it's about narratives and perspectives and how we're seeing the human experience and what we think about what the human experience is and what the priorities for those living that experience are and the priorities is survival, food, shelter for everyone. And if we look at that and you start a project, you would think, okay, right, we're all equal, okay. And these people don't think that because they feel worthless. How can I create an environment where the narrative is changed and they know viscerally that they are seen, that they are accepted, not just like, oh, everyone is welcome. No no no no no, really. Let's get to the bottom of it. You really are equal to me. And we are all really, really here trying to, to live the best way we can. And we're all trying our best with the circumstances. And yeah, conditionings and narratives that we've been fed and we're all here, and all we have to do is support each other. And everyone deserves support and everyone can support. And just having a different narrative and the culture of the project and the organisation is so important."

Code: Priorities if starting new project
P7, Social prescriber

2.

"But honestly, just more money. More money. Give them enough money to get some clothes, like some actual clothes that, um, like two pairs of jeans and two t shirts, like a proper wardrobe and money for, uh, so we'll fund their laptops through the PP Plus, although that just mean that we then don't have PP Plus for activities. And if we don't college will and if they don't, you know, there's an educational bursary they can access, so they'll usually have laptops or they can get laptops. But it doesn't necessarily mean they know how to use them. And it doesn't mean they can fix them if something goes wrong with them. So they need IT skills as well."

Code: Priorities if starting new project
P16, Quality Assurance Teacher for post-16 education

3.

"I feel like, you know, being just really real with who are the most, who are the most vulnerable people in our society, uh, that for me, I say the people that are the most vulnerable or the people who don't have recourse to public funds, don't have immigration status, have all of this uncertainty. And for me, with sex workers, you know, are in this another kind of legal grey zone where technically the work they do is legal, but then everything around it is illegal. So, um, I think that those kind of projects which are really looking at, okay, who does need the most support and finding ways to support them that kind of stuff really excites me."

Code: Priorities if starting new project
P1, Social Prescriber

4.

"I think you, you want to find a yeah, a group of people who are going to do it to make sure that you've got a group of you who could support each other. Um, I and I would say make contact with, um, the people at the council who are already dealing with new Scots if there are such people. Um. And yeah, really draw on any social connections you've got in a community. Um, because you need a bit of a structure I think, on which to um, build. In terms of making contact with new Scots, um, I guess we, because we kind of came at it through the resettlement team. Um, maybe you just need to if you don't have a, a more, um, formal approach system then I think having uh, welcoming space with tea and coffee. Um, and encourage people to come along. And listen, chat, food, very important. Um, but be aware that, um, they might not, you know, people might not eat the food that you supply. Um, and, you know, so, um, Kitkats seem to be okay. You know, it's the very basic things, like, you know, the basic biscuits that are acceptable. Um, and then soon, hopefully, the, you know, people will bring their own, which are far more tasty. Uh, you know, food, definitely.

Um, what else would I say? If you're just setting up, I would say do some training on, um, cultural difference. Um, just so that you have some understanding of the basics. Um. Uh, what else would I say? Find other groups. And yes, even if they're not in the same area, but, you know, groups like ours and talk to them. Um, remember… Right, I suppose, be clear about what you're wanting to do."

Code: Priorities if starting new project
P13, Charity chair

5.

"It might be about refugees, it might be about old people, it might be about people in poverty. It might be people, you know… this, the model that we used for (charity) isn't about refugees. It's about dispossessed groups that don't get services and it's about how you bring together people who care and work on how to do something. So I would start, I've talked to different places around the country to try to get this to happen. So I would go and see people, that's what I would do. I'd meet people, I'd talk about, I'd listen to what they thought was important, what their ideas were, and I, I would just do whatever I could to support them to think it through, how to do what they wanted to do, where they were, with what was there."

Code: Priorities if starting new project
P12, Founder and research coordinator

34. Lessons learned from own project

1.

"Over the years we have moved away from the focus being on getting people into work, as we recognise this is not what everyone needs (and that people are worth more than what they do; everyone is inherently valuable) - many people attending our sessions simply need somewhere to be, share, learn and connect, but of course the sessions impact their lives in a myriad of ways. An overriding value is that people are welcome as they are."

Code: Lessons learned from own project
P23, Volunteer

2.

"So what I tend to say is let's just try distractions. You know, you have all this trauma, you have, um, all this, you know, ongoing trauma now, in fear with, you know, whether you're going to be accepted or rejected. Um, so let's try and look at it as a distraction. You know, we can go to the mother baby group or we can go to the, the, um, talk group, or we can go to a um, heads up group or whatever is on and try to concentrate in the moment, um, you know, on this as a distraction because, you know, it's hard to concentrate on a social event or a social activity when you have all this going on in the background. I think I've never been through the system, so I'm just trying to understand."

Code: Lessons learned from own project
P22, Social prescriber

3.

"Okay. It's very complex. And there's also, there's a term in psychology. Um, my clinical supervisor, he always mentions it, and I never remember the term, but he, he always mentions that something happens when you are in a position where you want to help someone and you feel that you should help them because they're coming to us for help and they're expecting to be helped. And then there's nothing you can do because it's a systemic problem, but then you're left with that feeling of, ah, I just, I didn't do it, I didn't do it, and I am, I am in a way benefiting because of the colour of my skin, because of the place where I was born. And I am benefiting from this system. And yet I can't help this person. Even though

obviously it's not my personal fault in that moment, but there is some psychological burden that goes with this feeling of helplessness or hopelessness where it's my fault. Constantly having to, to tell people, because in many cases, at (charity), we had to say there's nothing we can do. Like you would see someone who is homeless, street homeless. Hasn't got a chance to make an application. Doesn't meet any of the rules. Not a refugee. No. Nothing. And even though they've been here for, for ten years, they've got their life here. Whatever life they've been able to build with the conditions that. Having no status gives you in the UK. And you have to tell them there's nothing we can do. You have to continue. You have to either go back to your country to the abuse that you were running from, or you can stay here homeless without any rights. And it's, it's very draining psychologically. And there's no uh, now there's a little bit more, but there's not a lot of mental health support for charity workers. Either it's it’s front line, that's one of the things."

Code: Lessons learned from own project
P7, Social prescriber

4.

"So sometime even I'm laughing the community members coming to the office and they say, uh, I want to go to this area, which but I'll go into this area. And I said, I'm not the, I'm not the traffic. So because, because they're so confident and so happy to come to the office, even sometime we are laughing between each other. And I say because of the behaviour and the attitude we are showing to the community members, and now they're coming to the office and asking for bus direction as well, so which bus is going to which area. And I say this is not our job. If you just go to the bus station, even I don't know myself, because I never use the bus. But because of that relationship, they're they they're so happy to come to the office just to ask for the bus direction as well. So that bridging the relationship with the community leaders."

Code: Lessons learned from own project
P5, Charity director

5.

"Another barrier was which I didn't say was, is that they didn't want to look, that they were taken from our government. They didn't want to look, that they were taking money. They were they were very guarded. They didn't want to look like they were benefit scroungers or coming over here. That was they were fully aware of the stigma around coming to the UK. And how they were received as refugees. Because they were being put up by these lovely families in rural areas and, and a lot of them had really good jobs back home. They were like lawyers, doctors, you know, therapists, teachers, you know, professions, you know, mortgages, cars, all that sort of thing. Um, they they were in hardship because the war put them in hardship. But they weren't they didn't live in hardship before then. So it was very alien to them. And they they didn't ask for help. They would never ask for help. We had to, like, put ourselves on them and say, this is here for you, just for you and for your child, because we want them to be happy while they're here, and we want them to enjoy themselves while they're here. And that's the only way we could get around it. Um, and then obviously the studies [inaudible 46:15] had to be used. But we said it was a formality. And um, you know, this was part of the, you know, the process. But you know they did find it difficult."

Code: Lessons learned from own project
P2, Youth service manager

6.

"Oh, so again, that can be challenging. These are the patients where we tend to say to them, we don't feel like you're ready for this. Um, we'll have patients that will come to us. And again, it could be an elderly lady who's very isolated. Um, she might, you know, have had a fall. She might have lost a confidence, or we're trying to get her out into the community to

reduce her isolation, to go to strength building classes to reduce those falls. And then we're just met with “Oh, I'm not a sociable person. I don't I don't like mingling.” So then we kind of have to dig into that and we'll say, oh, you know, tell me about how you met your husband or what? “You know, I actually met him at the local dance so many years ago.” Well, you know, you're sociable. Then what happened? So and I will often say to people, “what are the barriers if you tell me, what is stopping you?” So to hear it from their perspective, and then I'll put a spin on it and I'll say, well, you've said this, this is what I'm hearing. Can you just let me know if that's correct?"

Code: Lessons learned from own project
P17, Social prescriber

7.

Person 2: "I think the only thing is that, is that we live in a world where people are scared. So we are scared of losing our job. We’re scared of climate change. Violence, robbery, war, you know, there’s a lot to be scared of, and I think we have to acknowledge that, and not pretend that we can make things safe. So a friend of mine talks about how her father used to tell her there are two kinds of people. There are life enhancement and death avoidant. And you know, probably there are more than two, but I think what I want to say is to trust. Trust ourselves. Trust the environment. Trust each other. You know, it won’t always work. Lots of times, lots of times that trust will be, you know, a bit like, a bit like that research involvement. But it’s not going to stop me trusting somebody next time. It didn’t stop me trusting you, even though you’re a researcher.

Person 1: That's. That's true. Yes. I'll take that one. Yes. Yeah.

Person 3: Yeah. And trust that something is possible. You know, doing the same thing as everybody’s one because it’s safe, isn’t safe. It’s clearly not safe. So let’s try something else."

Code: Lessons learned from own project
P12, Founder and research coordinator

35. Learning from work

1.

"But one of the things that I've learned over the years is that people present very differently from what the reality might be, and I think this is one of the most striking things when you're first working in any way or meeting people who've had probably any traumatic experience. But, and it's particularly noticeable, I think, with the young people, young guys who, like, seem like these great young people, really up for things, really well and healthy and happy. And then you see what of the… I don't know if it is quite the right word, but you know, you would just, you know, obviously understand this of where that trauma sits. And so the awareness of that, going back to what I was saying about giving people time, about being wary of pressure."

Code: Learning from work
P6, Producer

2.

"But, um, I think what the learning has been is that it's very easy to. Well, first of all, building a relationship is fundamental, and that takes a bit of time and a lot of trust. Um, and that people who have very severe post-traumatic, they're not meant to be referred to the project with very severe mental health problems. But it's not always apparent, I think, when they are referred, how much their mental health is affecting them and people who are not able to leave their accommodation, who have a lot of social phobias, which is quite common for refugees who've experienced displacement, it’s quite hard to work with them. Because what

we're encouraging through the peer support work model is for people to basically go out and about to build community contacts to build social connections, to find out about the services that can support them and build relationships with those services to perhaps have contact with the wider local population through maybe sporting activities, social activities, whatever it is that matters to the person following the what matters to you conversation that they have with them. If that person has extreme anxiety and extreme social phobia then it's very hard to work with them, I think that's one of the cases that's been very difficult. Not, not, not saying that they don't get benefit from having that contact from the peer support worker, but to do the work, the signposting and the accompanying work and moving them on is challenging. Um, I think also the other finding has been around where the person is in their displacement journey. So if they're newly arrived, we won't we don't take people who are, you know, literally just arrived. But we take people who've had the sort of basics taken care of. But nevertheless, in those two different contexts, the two different sites, one of the sites is seems to be, um, less able to deal with those basic what you might call case worker issues, which are at the bottom of Maslow's pyramid. You know, the, the housing, the food, the, you know, shelter, the, you know, and has actually kind of co-opted the peer support workers to do casework for them, which wasn't, it's not explicitly stated, but that is in effect what's, what's happened. And so the peer support workers have found themselves involved in lots of filling in forms and, um, as a by-line that also happens in social prescribing. You know, obviously, I work in social prescribing for (city), and that's not uncommon at all."

Code: Learning from work
P4, Senior researcher

3.

"But I had this sort of revelation as well that I don't need to go to the world because the world is in (town). I've got more countries here than, than ever. And actually I can be more useful, um, supporting people arriving here. I understand our education system here. So it was a bit of a sort of head flip rather than me going over there to be helpful. Actually, let's be more helpful here. Um, and this job was perfect because it combines my interest in international, um, affairs and politics and all of that, um, with teaching and education in the UK, which are the two massive things I was interested in. And it kind of mushed together in, in this job, um, which I inherited from somebody who'd been in it a couple of years who hadn't been a teacher, actually. And I do think coming into this job as a teacher helps you see it through a very different lens, um, because you understand the restrictions and the challenges sort of first hand. You've got the lived experience of that in schools so you can pitch things differently. You can relate differently. You couldn't just. I guess. Yeah, you've lived through the pressures, so you understand why some things are harder to do than others."

Code: Learning from work
P19, Schools coordinator

4.

"You can't just say oh Ukrainians. Um yeah. You know, you know it's like saying the British, you know, there's Welsh, there's a Scottish, there's people from Cornwall, Londoners, you know, it's you can't look at them in a, in a generalist way. And we've, we've learnt a lot about the culture as we've, as we've gone along and we try and open ourselves up to lots of learning with that as well. So, um, training courses and, and speaking with our Ukrainian contacts and our translators and our we have an advisory panel."

Code: Learning from work
P2, Youth service manager

5.

"I mean, I learn something every day from, from a different community. I mean, from this, from the notion of, uh, of a Chinese older lady having been on, um, you know, in the ICU

during the pandemic, um, to her still being on a drip because she wouldn't drink, uh, the stand on that. And our staff is saying, well, what do you give her to drink? Well, there's a jug of water. Yeah, but Chinese people don't drink cold drinks in this environment. Can we just give you a flask? Put the hot water in it. And within a day, she was off the drip."

Code: Learning from work
P18, CEO

36. Environment

1.

"They have a slow cooker group. Um, they have, like a trip to the park to do laps around the park group. And actually they find lots of the mums who are too scared to go out, particularly when the Afghan mums were there a few years ago who were in the hotel and just the school gate was their safe place. That was it. Um, they used to take them to the park to just walk around and introduce them to the park, which gave them the confidence to do it. But it was also physical exercise but also help build community."

Code: Environment
P19, Schools coordinator

2.

"Um, it is also great when people get very practically inducted and, um, integrated into their new city by just way of showing people areas and paths and cycling routes. Um, we're very lucky in (city). We live right by the ocean and with lots of green spaces around us. Um, um, with a sort of gorgeous coastal path. Just lovely walking path running along. Um, so there's a lot to explore and there's a lot to take people to. Um. And also we just, we.. there's sort of one side of it is that we want people to feel like they, they gain a tiny bit of ownership of their new city. They learn an area where they can spend time in. On the other hand, it's also sometimes good to have a good day. Um, and it's, um, it's really valuable to have a memory of a good day, and you can then anchor yourself in that."

Code: Environment
P15, Wellbeing manager

3.

"So yeah getting them outside and and appreciating the outdoors and um, having um physical activity like bikes, like walking, like, you know, Duke of Edinburgh's Award or that sort of thing. Um, yoga, uh, foraging. We live we live on the dams and we're by the sea. We're really fortunate in our environment, but getting the kids out there is really difficult. So I think those projects really work. And it's just nice to see, you know, um, happy kids outside enjoying themselves. We've got a youth centre and it's all very insular. And my aim is over the next year is to get everybody out again. You know, out in the community. I think Covid has a lot to answer for that as well. Um, so yeah, that's one of my favourite things in youth is the importance and the impact that outdoor outdoor education has in formal education."

Code: Environment
P2, Youth service manager

4.

"Simple things. Like a bicycle. Yeah. You know, uh, just being, being outdoors and being, being, being active. And the evidence and research is there. You know, the evidence is research is very, very, is very black and white, and I know this, you know, uh, from my public health background, uh, you know, I'm familiar with a lot of research."

Code: Environment
P11, Founder and CEO

37. Donations

1.

"Uh, so we need to have, uh, like, uh, find the businesses like other people to help. Like, uh, I'm, uh, um, uh, starting volleyball. I talked to, um, uh, two, three businessmen. I said, we're going to support you, we're going to, uh, you have to be sponsor a bit with these sorts of things so we can, um, you put your details into sort of things in media and that, so there is more people they can buy your product, these sorts of things. We have champion here, if you want a personal support, you can do this one. We have to go bit openly, otherwise if we stay one thing, if it's nothing, then we stopped."

Code: Donations
P3, Youth and sport manager

2.

"Um, then I took him to a place where it's called the (name) Food Club. So you pay £8, and then the next week you get a massive big crate with lots of food, lots of, uh, fresh produce, but also some other things. But it's, you know, and it was twofold. It's so good food doesn't go to waste. And so I said oh, it's a bit far. Are you able to go? Oh, I'll just take the pram with the baby and I'll just, you know, so we have people like that."

Code: Donations
P18, CEO

3.

"Um, there was, uh, a particular charity who run the (area name) Social Prescribing service, um, have like a Christmas fundraising drive every year, and they get loads of donations, um, at Christmas from organisations. Um, so yeah, this year we had loads of like Argos gift vouchers and then like toiletry boxes and just random stuff, like really random stuff. Amazon gift vouchers. Um, and so for me, that was also a giant relief that for some people that I can't go and access certain funding streams or whatever for them. I was like, here's a £10 Argos voucher. I know it's not much, but I actually meant like loads to some of the clients. So I'm thinking about some of the asylum seekers that I was working with and yeah they'd be like, okay, what can we do? Let's let's google it now, let's see what we can go and buy on Argos at the moment for a tenner. Actually I'll give you two vouchers that will, what can you buy for £20 on Argos? And you're like, oh my god, perfect. We've found this, this thing that is exactly what you need at the moment. So like that was brilliant, but again, it's just like totally random, to be honest. (Person 1: Yeah) Totally random."

Code: Donations
P1, Social Prescriber

4.

"So bikes are mostly donated by individuals within, within communities. We also we also received bikes, uh, abandoned bikes from, from universities and colleges. We get, we've had some bikes from Police Scotland in the past, from housing associations where bikes are also abandoned. We have to be selective in terms of the bikes that we that we accept that we take because we have limited time and resources, you know, so, you know, we always say to people, the better the condition of the bike, you know, the better, because we have, uh, you know, as I mentioned, we have a significant waiting list. So the, the quicker that we can, the quicker that we can get bike fixed up and into people's, people's hands, the better, you know, so we're not able to just accept everybody and any bike, you know, that is donated to us."

Code: Donations
P11, Founder and CEO

5.

"And that's why we rely a lot on like, you know, community fundraising. We've organised some fundraising events, like we did a walk recently. But also and I mean, since the crisis. And it's interesting because when the crisis in Afghanistan happened in 2021, like we were receiving, uh, because we were like, but clearly like the leading refugee organisation for Afghans, we received actually a lot of unrestricted funding, sometimes from like trusts, some from like the community, but then of course, like once that and then similarly when the war in Ukraine happened. But, you know, uh, once the attention is off those areas like.. slow down."

Code: Donations
P8, Integration manager

38. Stakeholder strategies

1.

"So yeah, in terms of, of finding the services that will help the person, that's, that's where the role becomes really important, because part of the social prescribing role is to do that research and to have that relationship with community organisations. So there's a huge part of it of that, it's about looking at, okay. Right. So this is the local area. What is available within the local area. If it's a very specific problem and like immigration for example, you're not going to find free immigration advice next door. You might have to move okay. What's outside of (area of city). What can we do? There's a whole, um, amount of research we have to do to find as a team and we've got meetings with third party organisations or new projects coming through, we're constantly bombarded with new projects in here, just to keep track of everything."

Code: Stakeholder strategies
P7, Social prescriber

2.

"Um no it's, it's actually quite nice because it's quite organic. Um, so depending on, on each case, um, you would research for different organisations and then talk, I would just call them and say, okay, can I just have a chat with you and would just have a teams chat? Okay. What do you offer? What do I offer? Okay, great. This is fantastic. That is a very functional, very okay, how can you help my client. And then we just, yeah, we just make lists of everything that's available. And then whenever we have a question, we, we talk to them and we have a monthly third party meetings that we invite, um, different organisations that, um, we, we have come across that look interesting. We invite them to talk to us about their services. And we get to know them a little better. We also have an outreach, outreach team um, that goes out into community centres and food banks and gets in touch with very grassroot projects and they bring them to third party meetings. So there's a constant barrage of emails coming through all the time with these services. So yeah, it's usually quite, we don't have time to build hugely like, I don't know, like a strong connection, like, oh, let's just talk. But we do the minimum to establish a strong relationship so that they would accept referrals. And it's the same for them because it's also good to, to know what we can do and how we can refer people to them and what type of people we, we will get that can go to their services. So yeah, yeah, it's ongoing, constant influx of relations with new projects that come through because some projects come and then they go and then it's constant. It's just so vibrant in terms of services and community projects."

Code: Stakeholder strategies
P7, Social prescriber

3.

"Um, I've been much more blatant about our funding needs on the newsletters, um, and to organisations we work with. So for example, the council, (name) Council has the education access team, who are the team at the council who are responsible for when Asylum-seekers come to (city) when they go to school, or for Roma Gypsy, the groups that all have sort of access um issues there. So I work quite closely with the refugee um, guys who do that and have said to them a few times because I spoke a bit about how schools are Sanctuaries have different models and different regions. In (city), for example, schools of sanctuary are embedded within the council. Um, it's embedded within the EO Department. So the EO lady also oversees schools of sanctuary and rolls that out in schools kind of together. Um but (city) council doesn't have much capacity. And it's notable from having, I used to work in (city) so they don't have any, you know, any consultants that would work on that. Um, so point being, I have occasionally I've tried to say, oh, if you ever want to kind of, you know, fund some of our work or adopt it, um, we could do it that way, I guess, just looking at different models of how we could be sustainable, um, and, and how we could sort of embed ourselves within other organisations who might fund us even or be looking at."

Code: Stakeholder strategies
P19, Schools coordinator

4.

"And for example, for Refugee Week, uh, I contacted them because they have funding to provide meals at lunchtime. Where we don't so, um, we said, can we borrow a couple of your volunteers that have got their, um, you know, their, their food hygiene and can they come to us and cook for us? We're just looking at doing, um, um, there is a, there is another award that is around partnerships, and we work really, really well together. So we're both going to apply and saying, you know what, what actually, what that partnership looks like, how we support each other."

Code: Stakeholder strategies
1007AF_fina

5.

"And there was also a community centre, again thank God. In my first few weeks I tried to go and, you know, run around before I was staying client. So I was like running around trying to just connect with services like the neighbourhood forums or whatever. And I found that there was a community centre, um, over the like a few minutes walk from my surgeries, um, that majority of my clients had never heard, in fact, I think most of my clients all hadn't heard of. But it has so many amazing things going on there, the majority of which are free, um, including a lunch club where people come together and cook together and stuff, but also a gardening club where people also like growing the food that they can cook."

Code: Stakeholder strategies
P1, Social Prescriber

6.

"And we've noticed one of the things that people will also sort or will also arrange within that WhatsApp group, will, you know, arrange to meet up with each other, you know, so during the summer, you know you know, uh, to, to go cycle. So we can just offer that and, you know, somebody will write and say I've got a puncture. I don't know how to fix it. You know, before we jump in and say, oh, bring the bike to us, you'll find that another refugee will jump in and say, I know how to fix a puncture. I'm coming to fix it for you. So it's, you know, so it's quite interesting. I mean, as I said, there's about 300 people in that group. Uh, and it's become a bit of a, you know, a bit of a peer support forum and group as well. And people are self-organising"

Code: Stakeholder strategies
P11, Founder and CEO

39. Supportive relationships

1.

"I would say supportive. Okay. Yeah, yeah. So everyone has been supportive. So within the company I can refer to the Community and Nutrition Development group, which do a cooking, uh, 6 to 8 week cooking class."

Code: Supportive relationships
P22, Social prescriber

2.

"We're in many ways lucky in this country, despite the fact that the refugee sector has been defunded. There's an amazing network, a lot of voluntary groups as well. So like the (refugee) network and Refugee Week does give an amazing focal point for that network. So I would feel quite confident that this person, if they're able to pursue it, will find an op-, someone willing to “yeah, let's do that for Refugee Week.” It just makes it so much easier. Of course it could happen any time, but it just brings people. It just gives you an easier way.”

Code: Supportive relationships
P6, Producer

3.

"We have a lot of, uh, social or cultural events that purely been supported by the Afghan business, uh, men that working, that have businesses within the community. So they are supporting, they are raising their businesses in the event as well, as well as they're sponsoring the events. So it's nothing for us to pay for that. We are just organising that sort of, uh, social and cultural event, uh, being supported on, sponsored by the business holders within the community."

Code: Supportive relationships
P5, Charity director

4.

"Our kids don't really access a lot of the charity stuff. They tend to access more statutory services. So they'll access college, they'll access kind of social care. They'll go to the youth zones. Actually, they're much more likely to go to the youth zones, which are fantastic in (city). Actually, I know you have to anonymize this, but like the services in (city region) are really, really good and have amazing support, so they'll go there a lot of the time. Yeah. Um, yeah. They do tend to kind of group together, but they have homes where they're grouped together and they have colleges where they're grouped together."

Code: Supportive relationships
P16, Quality Assurance Teacher for post-16 education

5.

Person 2: "Anything else? I’d say the food bank. I'm absolutely in love with the food bank. I went and did, you know, um, little volunteer stints at the food bank to get a sense of, like, what it's like there and also just really love the team and happy food bank, um what yeah (Person 1: Yeah) Incredible place, incredible place. I was issuing food bank vouchers all the time, so yeah. And that is a service that I could do for, uh, asylum seekers and refugees. Thank God."

Code: Supportive relationships
P1, Social Prescriber

40. Staff burnout and secondary trauma

1.

"So after a while, like, you know what? I don't want to be fighting with the Home Office for most of my working hours, which is what immigration casework is, is just constantly fighting with caseworkers and racism and just seeing the ugly truth of things, that are just incredible racist and sexist refusal letters and interviews that they have with clients were just, yeah, the ugly truth of the world we live in. And you can only do that for so long. Before you, I mean, this is my experience, I know people, some people can do it forever, but I just many of my colleagues and me included, were burned out very quickly because a lot of the huge emotional labour."

Code: Staff burnout and secondary trauma
P7, Social prescriber

2.

"There is no acknowledgement of how hard it is and how much emotional burden goes into it, emotional labour goes into it and how draining it can be because you're switching. First of all, that you're making decisions all the time about what you think is right for that person, what services could work. Then you're switching contexts all the time, which for the brain is like exhausting. So you're going from one crisis to another to another to another, and at the same time trying to give hope, bring some positivity. Make them feel welcome, like you see them, you hear them and then have a problem with it. It's a lot right. So because that's not, um, reflected in the job description."

Code: Staff burnout and secondary trauma
P7, Social prescriber

3.

"And they tried to kick him out both years. He'd not made any progress. I got our EP to go in who wrote up a report that said something like, he would really benefit from some 1 to 1 support, and they therefore kicked him out, saying that they couldn't meet his need because they didn't want to offer 1 to 1 support. They didn't ask us because in our homes, we do have workers who will give 1 to 1 support. They didn't ask if we could put anything in place. They didn't apply for any HCP, even so that he could maybe go somewhere that was a bit more specialist to support him. They did nothing. They just kicked him out, like they will be actively cruel. I do think it's probably compassion fatigue, like I've, a lot of my colleagues used to work at this college and know the people I'm talking about and will say they used to be kind, pleasant, caring people, and they're not anymore. And I think its numbers and I think its second-, secondary trauma, but it's very, very difficult. And I've been fighting for three years now to keep all of our kids, not just unaccompanied asylum-seeking kids in education and with college understanding them and with support in place for them. And it's getting harder and harder every year, and they are withdrawing more and more from us every year."

Code: Staff burnout and secondary trauma
P16, Quality Assurance Teacher for post-16 education

4.

"Um, but again, if you've got, you've got these competing forces, so you've got that desire to be more trauma aware and trauma sensitive, but then still got lots of vacancies, clinical staff under pressure, budget cuts and so on. So you've got two very different competing forces at play. And uh, um, yeah, I don't think that it makes for an easy mix. Um, um, what people would like to do, uh, in terms of the practice and what they often force to do because the career of the staff, they're, they're short staffed. The vacancies are then held for months, sometimes just to save money before they're recruited to. So you've got the theory and the practice, which are two different things"

Code: Staff burnout and secondary trauma
P20, Regional manager for health inequities

5.

"You know, we've struggled recruiting at times. Just, uh, just, just because people that previously would have been working since the pandemic, they've either decided they want a totally different kind of job because it is very demanding what we do and, and it's quite emotive as well because we're working now with lots of, um, asylum seekers that have become refugees."

Code: Staff burnout and secondary trauma
P18, CEO

6.

"I think a lot of people felt really real pressure to be getting through, um. People not taking any breaks or, you know, working through their lunch times, um, squeezing in extra patience and actually seeing more than seven patients a day, all this kind of stuff. Um, and whether or not that was actually what was needed, that it felt all the time, like we are being pressured to do this. Um, and it could be really hard to push back to the GP surgeries, um, and be like, 'no, I need, I need some time to get catch up on admin'. And do all that, you know, measuring impacts or like data entry or whatever it is. So yeah, that that kind of challenge."

Code: Staff burnout and secondary trauma
P1, Social Prescriber

7.

"It's really up to each person. But if you have seven clients a day, that's like seven hours, you have a one-to-one appointment, right? (Person 1: Yeah.) But that one-to-one appointment has to include time to write up the your notes ready. So each person is going to use that differently. (Person 1: Yeah.) Um. But then like I say sometimes you're just, I mean, I would even have GP's double book appointments in there and squeeze in extra patient. So I was like, okay, I don't have an hour appointment now. If somebody's got a half hour appointment and I still need to do all the other bits around it. So yeah, if you'd have an hour-long appointment, which would infuriate GP's as well, because they have ten minute appointments, right? (Person 1: Yep) Um, so I mean, it's amazing for clients that, I think one of the reasons, that they really value the service is because often they're like, whoa, you actually sat down and listened to all of my problems rather than, like this tiny little fraction. Um. And so even if you're having like a half hour, 45 minute appointment and then writing up your notes, it still feels really valuable to the client. But often I think like the GP is a sort of like, why do you need all that time? We only have ten minutes, why do you need a lot of time? And, um. I think just different expectations on the notetaking and all this kind of stuff and what we're offering. Um, that could be quite challenging."

Code: Staff burnout and secondary trauma
P1, Social Prescriber

8.

"Things are just, you know, a little bit, like, I mean, one I remember and it always happens on Fridays, for example, like we had one time we had a this was social prescriber. It was actually a police station. It was like I think it was like 3 or 4 p.m. on a Friday. We had a police station call us about like a domestic violence case of a woman who, like, she was like wanting to go back to her husband. But the husband was, like, threatening to kill her. It was like a really severe case. And she's like, I need someone from like, the Afghan community who could, like, talk to her. And so and at the time, it was like, I think there was an event or something going on. Most of our stuff is where our office is very short staffed at the moment. Yeah, there's like nobody else around. And honestly, we did not have anyone who could, like, speak her

language available at the moment. And I said, tend to say to her, I'm very sorry, like we can't help at this moment. She was understanding, but it was tricky and I felt really bad."

Code: Staff burnout and secondary trauma
P8, Integration manager

41. Working conditions

1.

"Yeah. So there is a model. Uh, the idea is it's a short-term service. It's a signposting facilitating service. And it's short term, the idea that I think 6 to 8 weeks, I don't stick to that strictly. And I haven't had a review of the service yet. Um, it's probably something I should have said from the very beginning. Um, so this, this service hasn't been reviewed. So anything you hear is news is me, um, and my supervisor."

Code: Working conditions
P22, Social prescriber

2.

"Uh, so we try to, like I said, instead of full time working with a part time, uh, staff, uh, some of the work we doing as well, using our volunteers to do, uh, some of the activities, uh, being donated by the community members, such as, for example, such as the Saturday schools. Uh, the parents are supporting some or part of the, uh, expenses, like the paying to the teacher or, uh, pay for the hiring, the classes. Uh, so when we get the funding, we ask them not to support, we saying, oh, we have something to, now to cover, But some time is coming that we ask them that the funding is ending, so until we get a new one, so they are supporting and they keep running the classes. So that sort of communication and supporting each other within the community as well."

Code: Working conditions
P5, Charity director

3.

"But it’s very little for 1.2 million population for (region). So it's, uh uh, yeah, it, uh, the numbers are slightly scary. I know the ratios of specialist skill to, to, to challenge."

Code: Working conditions
P20, Regional manager for health inequities

4.

"And we have two social prescribers and a coordinator on the team. Um, and the social prescribers are split in between the two different age cohorts. So one social prescriber commits to primary school age and one to secondary school age. And that's how we kind of divvy up. And then they share the workload. Um, like, um, it's quite even, to be honest, actually, there's, um, I'd say going into the third year of the project, uh, slightly more secondary school age, because those primary school children, some of them have moved up to secondary school, but they obviously stay with their original social prescriber because it just makes sense to you because the relationship’s there. Um, and then the social prescribers work with them across the whole of (area name), um, and support them all the time, their living or going to school in this county."

Code: Working conditions
P2, Youth service manager

5.

"And, you know, I suppose we are, uh, we are a charity that isn't very flush when it comes to, uh, management. Just myself. And, and we now have a full-time office manager. And then that's it. Uh, so I tend to work like, I've got meetings now. They have to happen during the day. So then I haven't looked but from last night, there were already 25 emails that we send

in the evening. But those are the people I liked, I felt and I properly, you know, and I also come in on a Saturday if need be, on, on the Sunday. So I probably work about 60 hours a week at the moment. Every time I say, listen, I'm going to work my hours in four days, something happens and it doesn't happen. I see, but I think all our staff are so committed to supporting the community that they freely give their time. I'm privileged to work in a, in a setting where, where people are not just there for the money. They are there because they want it. well, they wouldn't come for the money because, you know, we're paying pittance to everybody. Uh, but they do it because they feel very strongly connected with their communities."

Code: Working conditions
P18, CEO

6.

"So it tends to be a permanent contract from my understanding with (charity), that is a year rolling contract. So that does tend to be a bit of anxiety, I think at the end of the years for the social prescribers to see whether the PCN is going to fund them back, but because I'm part of a PCN, I'm, um, kind of protected in that, so I don't have to wait for that. I'm just permanently employed by them."

Code: Working conditions
P17, Social prescriber

7.

"Um, but she came to me with sort of tears streaming down her face and said, "Can you help me? Because, I'm in this terrible way and I don't know what to do and I don't know where to turn. And the GP says it will be 12 weeks before I can get a referral". And I just had to, I mean, luckily because of the age I am of the demographic I'm from, I have friends who are therapists. So I then had to go and spend some of my own time the next day, frantically ringing around trying to get some help for this woman. You know, which isn't my job. Shouldn't be my job. It should have been possible to get her some help and support from the organisation itself because, like so many of the others, she's done masses of fundraising and reading and performing and writing and publishing for the organisation in the past. And I felt very, very bad saying to her, "Yeah, I'm sorry, they're not going to help you". Um, so I felt I absolutely had to find another way around it. So, you know, there are situations in which people's mental health becomes extremely unstable and it does weigh on us sometimes, because there aren't other places for them to go. Um, but that's just part of the job, really. And unfortunately, with the way things are with the asylum system in this country, it's only going to get worse."

Code: Working conditions
P10, Writing project coordinator

42. Emotional response

1.

"Do you know, people are amazing that they are just, they never cease to amaze me what people achieve and the attitude they have. And, you know, the sense of humour and, um, you know, all those things to survive… It's difficult, living."

Code: Emotional response
P22, Social prescriber

2.

"It's really moving. Actually, I am, quite often get quite emotional on like appraisal days when I realise just it's so much more than reading and writing. And that is, you know, the goal

ultimately because you want everyone to get jobs, but it's so holistic, the care that schools give."

Code: Emotional response
P19, Schools coordinator

3.

"At the end of the day I don't look at and I, and I maybe this is a bit of a naive way to look at it, but I look at every single child as an individual and what their needs are and their trauma is no worse, or they're no more or less or more important than somebody else that's experienced trauma to me. They are a child, they need help. I don't care where they've come from or what country they've come from or how they've got to come on my project. If they're a child and they need help and they're experiencing trauma, then that's what I want to do. And we want to make, you know, a positive intervention for them. And, you know, I don't want to get caught up in all of this. You know."

Code: Emotional response
P2, Youth service manager

4.

"I'm always amazed, and this is going to get really stupid. But I'm always amazed by how beautifully they dressed compared to most British people. I mean, they do not come with stains on their clothes. They don't come wearing baggy jeans, they don't come wearing sort of some old kind of hand-me-down. They, they, they come looking as though they're sort of fresh from the cleaners. I don't know how they do it, I genuinely don't, but, but there's something about I think there's some connection with whatever sense of dignity and self-worth got them through it, whatever feeling of I'm worth, you know, my life is worth saving. I am worth surviving. I'm worth fighting for. That somehow presents itself in this this demeanour of sort of, well, as I said, they just, they just, they just look, I'm always amazed by how incredibly smartly they turn themselves out, given that many of them are living on £35 a week or £8 a week or whatever it is, they get what you know from, from the measly handout from NAS."

Code: Emotional response
P10, Writing project coordinator

5.

"And I think that for us to be able to actually surprise people with a different view of what an asylum seeker or refugee or a survivor is, and to say, you know, these are people who've given up everything they ever had and everything they ever knew and left behind everybody that they ever cared about and taken a completely risky, dangerous journey and arrived absolutely empty handed. And look at them now. I think that that is something that is really important to do, to, to counter the, the narrative of, of the sort of beaten down, broken, defeated person who needs our help and actually present them, as you know, these are people who can add, you know, they don't just add their cooking skills or their gardening skills or whatever it is, you know, they enormously enrich, you know, they're coming here offering themselves, offering all the riches and boundaries that they contain within themselves to us. And we're just like, you know, no, we don't want that. You know, we're going to keep you in a state where you're completely immobilised and you're paralysed and you're immiserated and you're impoverished and you, you can barely sort of draw breath, barely keep, keep your belly, have the courage even to carry on. We're going to keep you in this state for as long as you possibly can, you know, until, with any luck, you'll go away again. It just seems to me absolutely insane. And I think the more you can actually, I don't understand why our government doesn't tell the truth. I don't understand why nobody is saying refugees and migrants bring much more to the economy than they take. They bring much more to the economy than most native people do, actually, to be honest. And all they want is to be given an opportunity to give back and to add to the community and to enrich

our, the plat of our cultural life and, and, and bring to it the special thing that makes them who they are. And I get so mad that that isn't acknowledged. And I suppose that in whatever small way we're able to contribute to that and change that narrative and present these people as the sort of shining stars they are. Uh, that's what I feel our work is all about."

Code: Emotional response
P10, Writing project coordinator

43. Transport

1.

"And then while they were in the group, they might also have a brief meeting with their legal advisor at the same time while they were there, because the travel expenses were, were covered for the day, because they were attending the group. So I would be like, give me the paperwork, so it was very holistic."

Code: Transport
P7, Social prescriber

2.

"So I had a social worker on the phone. Incredibly cynical and patronising. No. Mum has support systems in (area). She can go there. She can stay there the night. She hasn't got money for transport. She's got a month-old baby and she is a child herself. She's 19."

Code: Transport
P7, Social prescriber

3.

"So having to walk, um, to get the toilet, um, having security on the gate as well. So if you did want to leave, you'd have to, you know, pass the security on the gate and then it's on a country road. So walking to the nearest town village, so it's in (town) village. Um, it's about half an hour. You've got nothing to do there. You get £8 a week, you know, not, um, not great."

Code: Transport
P21, Advocacy manager and primary care lead

4.

"And people were, you know, people were, uh, you know, walking to this project, you know, you know, there were maybe walking 2 or 3 more, more, more hours a day just so they could volunteer on this project. So I after the project was launched on launched on Facebook, uh, I started to take bicycles through from (city) to (city) and then, and hand them out to the volunteers within, within my friend's project, that were asylum seekers. And an interesting thing happened. We noticed that, hey, that, you know, some people who, who had maybe been volunteering a day or a couple of days a week, uh, because they were travelling, you know, from, from quite far. And (city) was a big city. And a lot of people host, you know, in sort of outlying, uh, schemes in the city. But we noticed that people started to volunteer, maybe not once or twice a day, but they started to volunteer or 2 or 3, four times a day because they had a, you know, of free, a free means of, of, of transport, transport."

Code: Transport
P11, Founder and CEO

5.

"If you're in a hotel, yes, you're getting a roof over your head and you know, and you know, and, and your access and, you know, sort of a sort of free meals within hotel, but you're given a financial allowance of £8 a week, not a day. £8 a week. So that's the only cash that you have in your pocket. So public transport is as out of question. You know as I think (city’s) £5, or 7. You know. So a bus pass you know that would be your weekly allowance, not, you

know a daily, a daily living, living allowance. So people are really, you know, really isolated really, you know, socially and economically disadvantaged."

Code: Transport
P11, Founder and CEO

6.

"I mean, we always I mean, luckily since day one, you know, we've had, we've had a waiting list, you know, and, and that waiting list has just continued to, to grow, you know, so there's a, there's a massive, you know, sort of level of unmet need for access to, to free transport. Transport poverty, is, you know, is a significant issue, you know, amongst, amongst asylum seekers and, and refugees, you know, so not surprisingly, you know, those are quite a significant demand. I think at the moment we maybe have over, I don't know, three, 400 households, not individuals, but households, you know, on the list to access, access bikes."

Code: Transport
P11, Founder and CEO

44. Stressors for refugees

1.

"We need a better visa system. We need better a better asylum system. We need them not to be terrified they're going to be kicked back to the place they came from. None of these are things that we can address."

Code: Stressors for refugees
P16, Quality Assurance Teacher for post-16 education

2.

"Um, and also often people, um, um, people are really affected that, by the kind of realities of everyday, the stresses of hearing from, um, their home office case, um, stressors related to housing, um, hostile living environment, lack of opportunities, etc., which talking therapy just cannot address."

Code: Stressors for refugees
P15, Wellbeing manager

3.

"Obviously, you're a new person, a new town that doesn't speak the language. It's hard to fit in to the culture of the (town), I suppose, but then you also have the ignorance of other people that are willing to be open to that sort of change, or that new sort of person to sort of find out who they really are. So again, that can be a catch 22 within itself. So you've got two different sort of scenarios at once, I suppose that can clash. Um, it all is down to this asylum seeker or, you know, the refugee. They are in the middle of that and they are, unfortunately, the ones that are getting the backlash of that when they are just trying to survive, which can be quite hard."

Code: Stressors for refugees
P17, Social prescriber

4.

"So with this psychoeducation workshop, like the ambassadors have been really clear that at the beginning of it, we really have to label the point that we have no connection to the Home Office, like, you know, and it feels like it's like, why do I need to say that? Like, of course I don't have a connection with the Home Office, but yeah, we have to bring it and force it and say, like everything you say here is not going to be taken back. You know, if it's, um, really it is, it's a very and that's like, it's so much more basic than you could ever kind of think that, that where that conversation needs to start. (Person 1: Yeah.) Um, and yeah, just how fearful people can be of, of services."

Code: Stressors for refugees
P14, GP and lead for local health stream initiative

5.

Person 2: "Yeah, as I've been saying, there's a lot of good services and support out there. But how do people access it? You know, if you yeah. You know, if you're stuck isolated at a hotel, some hotels are miles away. I mean, I'm aware of a hotel. There's a hotel which accommodates asylum seekers down and just down under (other city). And I think it's about I don't know, that's seven or eight miles outside. It's in the middle of nowhere. Yeah.

Person 1: Yeah. And you're not going to be walking in or, you know, you'll walk into town from there if you're really desperate, but you're not going to do that for fun every day or to access something.

Person 2: No, no, not for fun. And you know, you you'll do it if you have to. You know you'll do it. Of course. You know if you're having to. You know, if you're accessing legal support, you know, and you're, there's an expectation as well, you know, that you have to check in with the Home Office of every so often, you know, so that's, that's a requirement. So you'll do it then if you're accessing, you know, sort of specialist health services, you know, mental health services. Uh, no, I mean, you're doing it out of, out of necessity, mostly. Mostly. And, you know, and that's difficult, during the winter, you know, with, uh, you know, with small children and, you know, and families and buggies and so."

Code: Stressors for refugees
P11, Founder and CEO

6.

"They will have incredibly complicated, chaotic lives. A lot of them have very bad physical health. A lot of them have not brilliant mental health. A lot of them are still being dicked around by the asylum system, or if not that, by the NHS or by, you know, the benefit system or whatever it happens to be. So their lives are a very stormy sea on which it's amazing they manage to keep afloat and let alone afloat enough to paddle to our workshops. So we're quite astounded, actually, often what they managed to do."

Code: Stressors for refugees
P10, Writing project coordinator

7.

"And especially at that time, some of them are in hotels and really like cut off from anything. They're very bored."

Code: Stressors for refugees
P8, Integration manager

45. Physical health

1.

"Um, and then they most of them work in cleaning jobs because they can't speak English, and they work in cleaning jobs for so long that they get all these allergic reactions to products. They get arthritis. They get medical conditions that are extremely complex. And then when they can't physically make themselves work, they feel guilty because they feel like they're taking advantage of, and those who are fine and they keep working, and I hear based in the group a lot, is they sort of share experiences and those who have never had to stop working because they never had a health issue and whatever they feel so proud was like, no, I'm still contributing to the economy and I'm not. Yeah, I'm not on benefits. I'm not one of those."

Code: Physical health
P7, Social prescriber

2.

"Um, so there needs to be a certain threshold. Sometimes it's physical. Like we need to be able to walk up a hill and down a hill. Um but sometimes it's, it has been mental um in that somebody is so unwell that they just, we wouldn't be able to ensure their own or others safety. Okay. Okay."

Code: Physical health
P15, Wellbeing manager

3.

Person 2: "Um, a lot of, um, refugees and asylum seekers understandably can't afford to go private. Um, so it's about sort of, again, working together to see what they are. So, um, just going back to like the sort of dentist things they can't really afford, they can't get on the NHS, um, dentist because of the waiting list. So I'll always speak to them about the alternative, which is University of Liverpool Dentistry School, um, provides free um dental and…

Person 1: Dental care appointments.

Person 2: Um, from the students there. And if they're open so that that is an alternative for them."

Code: Physical health
P17, Social prescriber

4.

"Yeah, and also things like the ins and outs of, um… I, uh, tried to get a lady, so she's got refugee status, but she's, she's living in, uh, three flights up and she's got a little, little baby. She's got significant back pain, and she's fled domestic violence. Um, and she's being sorted, supported by local domestic violence charity. Um, and they very, they came, um, to an appointment with her and were advocating and this lady was just like, I need to change house, I need to change house. I can't go up these stairs with this little baby on my back. And, and so I was like, I don't understand the ins and outs of housing. Let me book you in with the, um, social prescriber. But then the social prescriber was like, um, she was like, oh, but I don't I don't really understand. She was going… it was something about her refugee status, and I think at some point it, um, at some point it was written on the notes that she was an asylum seeker and she was going "But I don't really understand whether this is going to entitle her to any housing and all of that kind of the entitlement side of it as well." Um, and then sometimes people have like, sometimes people have a visa, which is a form of, um, like family reunification visa and kind of where does that leave them in terms of their entitlements to various different benefits or housing, whatever it is. Um, and that can be quite confusing I think."

Code: Physical health
P14, GP and lead for local health stream initiative

5.

"Another saved a woman's life because she got neglected septicaemia, and had, had kept going to the doctor's surgery, but not with an appointment, you know, not speaking English, complaining about stomach pain. You know, inevitably, you know, this was a "hysterical nuisance." #00:12:02-9#

Person 1: Yeah. Of course. #00:12:04-2#

Person 2: Yeah. And the students just insisted that they use language line. And the diagnosis happened, and she was admitted to hospital, so, and she had five children, so it would have been a disaster. You know, quite apart from the personal."

Code: Physical health

P12, Founder and research coordinator

6.

"They will have incredibly complicated, chaotic lives. A lot of them have very bad physical health. A lot of them have not brilliant mental health. A lot of them are still being dicked around by the asylum system, or if not that, by the NHS or by, you know, the benefit system or whatever it happens to be."

Code: Physical health
P10, Writing project coordinator

46. Mental health and traumatic experience

1.

"Lived traumatic experiences come up a lot - often we will support people who are distressed one-to-one, to make sure everyone in the group feels comfortable and that no one is triggered. We also have to be mindful of when children are there. It feels like an honour to be able to hold people's pain as well as their joy, and to offer acceptance and understanding."

Code: Mental health and traumatic experience
P23, Volunteer

2.

"I would say that the reality of leaving your home and leaving your country, um, arriving in a new country with a history and a culture that you know nothing about. Um. living in a room, you know, with your entire family. Uh, um. Probably feeling trapped in that room because of language barriers. Um. Other barriers. Even though you're living with people of your own culture, you know, living in a, in a group setting like that, I just I just couldn't imagine what the, what that would do to your mental health. So I think it's just it's just a reality that your mental health is going to be affected by this situation, by all that has happened on top of, you know, the trauma that you've experienced that led you to that in the first place. Um, so mental health is a, is a real issue. A lot of people are, you know, have are connected with their GP or um have if they're, if they're lucky enough to get into a mental health team, they're supported by that. Sometimes they're waiting. Um. They're often taking medications. Um, and. And then you have people who are here in the international protection system, you know, there are international protection applicants, but they also have responsibility to family back home. You know, and I'm just thinking of a client in this recently who was a man, I, I and I say that because I have 25% men and the rest are women, that the, the gender breakdown. Um, so but he's here earning or sorry, getting €38 a week. Um, feeling responsible for, you know, his mom and six siblings, um, in his country of origin. That's a that's a lot of stress. It's a lot of pressure."

Code: Mental health and traumatic experience
P22, Social prescriber

3.

"Everyone we, we speak to, pretty much everyone is in a crisis of some kind and with some pretty heavy mental health and problems, which seems like everyone. It's again, that's something that I find really important and something I would change at the job is changing the perception that, oh, that, that mental health, um, or anxiety or stress or isolation is something weird, something that, an anomaly that, um, just a few people struggle with and it's their fault because they're not making an effort. So this is the narrative, and it's a very capitalistic, um, like this meritocracy system where people are valued according to how much they produce and how much they do, rather than just by being the human being. So a lot of people are getting burnt out because of that system and the weight that they put on themselves to be someone in life, and then when they crash and open up about it, there's

just this whole load of, again, like self-loathing and like, it's my fault. It's my fault. So isolation, mental health, all the problems that I hear from the people I see, they think that it's, again, it's like what I was saying about migrants and how they see themselves because of what the system says."

Code: Mental health and traumatic experience
P7, Social prescriber

4.

"Um, but we felt one to one meetings for however long each time to talk about feelings and needs, and we felt that that wasn't really useful. We knew when they came to us what they'd experienced. We knew that for many of the children, it would be they don't want to talk about that. You know, they don't want to express themselves. And also, we're not qualified therapists. It was a level of trauma. It's not like a traditional social prescribing approach where you go to a year six and seven kids in a school that are on Pupil Premium and you go, right, okay, so your parents haven't got money to for you to play football or something like that, or you might need some help with behaviour. That, you know these uh this is like like really traumatic stuff. Intensive. Um and we knew that we were, we were not qualified as delivery providers to give that level of support. But what we could do was place them in fun activities to help repair the trauma that they'd experienced. And that was the approach that we took."

Code: Mental health and traumatic experience
P2, Youth service manager

5.

"Yeah I think mental health is a massive big issue. So, so a lot of them don't go to sleep to 4:00 in the morning. And then that means that, and you know, they ask me to make a GP or a GP appointment or the GP appointment is for 10:00 and then they don't turn up because they're still in bed. So we have people like that as well. They literally can't cope, but other people do come to us and they say, uh, I'm not good"

Code: Mental health and traumatic experience
P18, CEO

6.

"Um, obviously I was coming to the job as a trained therapist, so I felt like, um, when it came to supporting people who have had yeah really traumatic experiences, um, I felt, I guess, well equipped, um, to be able to like hold space for them and with there's a lot of people, um, in social prescribing who have a therapeutic background, um, but not all. So, I feel like there's, yeah, you're going to get different experiences with different social prescribers. Um, and definitely there were some, uh, refugees that I was working with where there was very little I could do practically, but I was like someone that they could talk to. And that was kind of all that I was offering, even though that's not what I was supposed to be offering necessarily. (laughing) I just have to make sure that in each appointment I was like, 'right, I'm gonna suggest a thing. You're probably not going to do it, but I'll suggest the thing'."

Code: Mental health and traumatic experience
P1, Social Prescriber

47. Cultural differences

1.

"There are certain women who are in quite a strict Muslim upbringing. And they don't speak here, so it's often the men talking for them and stuff like that. We've had fathers with teenage daughters and, you know, a bit of a light goes flashing in my head. So we did say, you know, for health reasons, we need to interview you, uh, separately. Just to make sure. And

because initially they were sharing a bedroom and I said in this country we have rules about that. So once a person, once a child is over the age of 9 or 10, we expect them to have their own bedroom. So and then there were two families, both or two men, both with their teenage daughters. So we were able to negotiate the girls would have a room together. And that said, at least we would just still have separate bedrooms. But the girls could go together. As a result of that, um, you know, the girls blossomed."

Code: Cultural differences
P18, CEO

2.

"Um, and again, I feel like a lot of people within the (region) itself can be quite ignorant to culture. Um, you know, we've all heard it. We've, I've had conversations on phone, on the phone to people that are from this, this, um, area, always, been born here. And, you know, we'll be talking about, um, benefits. And they seem to think, oh, you know, all of “if I came here on a on a boat, you know, I'd be in a five-star hotel eating five square meals”, because they do not have that exposure that I have. They don't see what it is actually like. Um, so I think the culture can work in both ways. Obviously, you're a new person, a new town that doesn't speak the language. It's hard to fit in to the culture of the (town), I suppose, but then you also have the ignorance of other people that are willing to be open to that sort of change, or that new sort of person to sort of find out who they really are. So again, that can be a catch 22 within itself."

Code: Cultural differences
P17, Social prescriber

3.

"Um, I think that's one of the things that we all really, really enjoy. Yeah. Um, learning about the cultural differences. And we have found that the, the Syrians, um, because are more than delighted to share and they don't take offence when you make mistakes. You know, um, so it's been a lovely journey just learning more about different cultures. And we have, from time to time, got people to do little presentations on, you know, aspects of their culture. Um, which is really nice."

Code: Cultural differences
P13, Charity chair

4.

"So it's like, yeah, culture. We've all got culture. You know, British culture's kind of not one thing. And you know, it's like, it's how do you work with people where they are, as they are in a way that is open, inclusive. There’s a chapter in a book I wrote with a colleague about creating commons, and the cultural kitchen is a commons and the allotment is a commons and it’s, it's the, it's always been, you know, this is some organisations kind of, uh, find themselves focusing particularly on a certain cultural group. Uh, I think the best example of how (charity) doesn't is that it has even when all the Ukrainians came, and that was a large number of people. They actually had a discussion about how to manage that presence, so it didn't overwhelm for the people who were already there. Um so it didn't do, cultural kitchen didn't do a cultural evening. The race equality did one and people were told yeah you can come. It's for everybody. So you know if you want to come to kitchen that's great. Come, come as you know it's for everybody. It's not a denial of culture but a recognition that it isn't, it doesn't mean one thing."

Code: Cultural differences
P12, Founder and research coordinator

5.

"I think, I mean, one other thing I would say about why they stay is that, as I'm sure you can imagine, I mean, you won't have heard, but there was a, there's been a big thing in the news

it for this a couple of days in this country about a big, um, terrorist plot from that was uncovered by some Iranians, um, who were plotting to kill other Iranians, you know, a bit like the sort of Skripal poisonings. And then that's something that we have, it's a very extreme example of something that we encounter a lot, which is that as a sort of outsider, you might expect that people from Congo or from Iran or from wherever it is would hang out with other people from that country. But actually the reality is that those are the people who might be from, you know, they might be from the other political party, who are the people that tortured you, or they might be, uh, government spies, or they might be deadly enemies of your family, that there are all sorts of reasons why people from your own cultural background might actually be the last people you'd want to could consort with. But on the other hand, in the group they encounter and work with people who have very different cultural backgrounds but accepted same emotional and psychological experiences, so they don't have to explain themselves. They don't have to make excuses. They don't have to behave in a certain way. Everything is understood and everything is forgiven and everything is supported. And I think that becomes really important. And I think it's what makes, it's what makes a group feel like a sort of second family for a lot of people."

Code: Cultural differences
P10, Writing project coordinator

48. Language barriers

1.

"The language aspect has been the most interesting thing. We had an assumption. Well, we knew from the co-production workshops all the issues around the interpreting services and how difficult that is and how hard it is to have a cultural, and that was again built into the study was cultural humility, cultural sensitivity, trauma informed practice. How is that understood within the services and how do they how do they provide that? Because it's not just about the language, it's about understanding the idioms of distress, you know? So the way somebody who's from a certain culture expresses mental health issues and it often somatises, often a stomach ache, a headache. What we've come to understand is that those differences are very personalised and that it's there isn't a way of saying everybody who's Iranian will say they've got a stomach-ache. It does so happen that Iranian people are more likely to somatize and say it's a stomach ache. You know, we have Persian speakers who've told us that. However, it's not everybody who will do that. So it's very much about being flexible and understanding, when people talk about not sleeping, having a headache, what that is to do with. The language itself is much less important than being open to those different cultural reference points."

Code: Language barriers
P4, Senior researcher

2.

"And I think especially when there are a lot of issues or if there's conflict, if I'm arguing with college, for example, that their child shouldn't be kicked out. Getting a word in edgeways is quite difficult. So you're trying to cram your bit in, which means you're not leaving any like, pauses for the translator. The kid has absolutely no idea what's going on. Um, yeah. And you kind of have to. It feels like you have to prioritise the professionals talking about the kids, because that's what's going to keep him in education or like prevent something difficult from happening to him. But it's a difficult decision to make. And I'm sitting there going, wait, can I just, can I just get a word in to challenge the fact that they are considering his absences unauthorised, when a doctor said he had tuberculosis and couldn't leave his bed? Or do I, instead of doing that pause and get the translator to translate that he's going to get kicked out of college because he wasn't attending when he had tuberculosis, like, and I'm almost

always gonna pick fighting with college. So yeah, it's just really difficult for the kids. They have no idea what's going on. They can't defend themselves."

Code: Language barriers
P16, Quality Assurance Teacher for post-16 education

3.

"And also sometimes, uh, when they go into the different clubs, the first, the, um, the first of all language barrier. They don't know how to (Person 1: Yeah.) ask them, how to do the help or they feel shy if they, oh, I don't have money, (Person 1: Yeah) uh, can you give me a free chance or something? They know, they're not feeling well to do that. (Person 1: Yeah.) I mean, uh, even, uh, I don't know, uh, when I came here, I said, okay, uh, I was practising it all before I start the service. (laughing) I said, I'm not going to go anywhere, I feel shame, if I go to ask them can I join free? or something like that. And honestly, the benefit they get is not covered sometimes those sorts of things."

Code: Language barriers
P3, Youth and sport manager

4.

"There's a lot of questions around sharing and “Am I happy?” Um, the emotion, that questions “Do I feel relaxed?", all those sort of things. And then you ask the same questions at the end of the project and then, you know, they rate them from 1 to 5? I think it is. Is it 1 to 5? No. Yeah. 1 to 5. And then there's supposed to be a difference at the end of that. So I mean with the older ones I think they were just telling us in the beginning what we want, they thought we wanted to hear. So they just rated high on everything even to start with. It's like, oh, we need to give you high answers, which wasn't the case. Um, so we in our findings, we kind of explain that, you know, we think that there was a communication language barrier, you know, a lack of understanding of what the scale means. And a lot of, um, you know, kind of lip service from the older ones thinking that that's what we wanted to hear, even though we did try and tell them that they could be as honest as possible and it didn't matter. You know, um, there were few honest answers. I mean, we did get a really good, um, selection to be able to, you know, put our, um, reporting across of good journeys travel. There wasn't a lot of, um, data that we were able to use."

Code: Language barriers
P2, Youth service manager

5.

"But with the social prescribing and the way that it was advertised from the beginning, parents didn't understand what that meant. So when you talk about health and well-being, they think that that's a financial thing. It doesn't translate. But when you talk about social prescribing, they feel that that's a health thing. And they're like, well, there's nothing physically wrong with me. So they don't identify mental health, emotional health and trauma as physical. You know, we try and encapsulate the whole thing. Well, you know, you can be unwell mentally and that's the same as if you've got a tummy bug or, cause we try and normalise mental health and, um, emotional health and well-being as is if you were poorly in other respects. But they don't they don't. There's no understanding there. So um, so it was really hard trying to explain to them what we wanted the project to do in this kind of therapeutic, um, way, um, and improve wellbeing because it just didn't translate. So we call it [name of the NGO] because positive translated really well and activities translates as it is. And we talked about, you know, um enjoyment and happiness and fun and those sort of, um, words to show the parents that's what we wanted for their children. We wanted them to have fun. We we we want we felt it was important for them to enjoy themselves while they were here. And out of that, from a reporting point of view, ticked the boxes for, um, recovery."

Code: Language barriers
P2, Youth service manager

49. Barrier characteristics

1.

"100%. That is number one, the one of, I would say, the number one thing that I loved about what (charity) have to offer. Because what I, many of us as advisers, found was that when people came to us. First, many of them were just terrified they would, so we were talking about people that had been hiding from the Home Office for years, and when they couldn't take it anymore, then they came to us or when they found out about us. So in many cases, they would, it would be the first time they find a safe space in the time that they had been in the UK, sometimes in years. I had clients that had been there for 20 years, and they wait 20 years so that they could apply for a visa, because this is a rule specifically for people who haven't got children, no family, no British relationships of like any kind, so they don't fit the family rules. They have to wait 20 years to be able to claim to apply for a visa. If they're not meeting the refugee convention or if they're not meeting anything else, and they have been physically present in the UK for 20 years, then they can apply for a visa. In the meantime, they're homeless. We had a lot of people like that. Imagine the trauma. And when we were dealing with their cases, we would see their files from previous solicitors because in many cases, they would have tried to apply for many years to claim asylum, to apply under another category, anything. And we would see the applications that other solicitors made in the past charging them. So we would never charge fees for the service, right? So solicitors would charge incredible fees, even though they knew that the case was not going to succeed. So they had been distressed, mistreated, abused financially by solicitors in years. So they didn't trust legal advisers at all."

Code: Barrier characteristics
P7, Social prescriber

2.

"So I think to go back to where I started, if you have a language that is less frequently spoken, it is harder for you to make relationships and it's harder for you to engage in community activities and any community activity that is designed for an accompanying asylum seeking children or refugees or whatever will do a really good, like good job of incorporating action and showing, not telling and so on. So it is possible, but I would, I can completely understand why kids will look at an offer and go, I'm too tired. Like I'm too tired to go and try and understand what people are saying to me for another two hours a day after college, like."

Code: Barrier characteristics
P16, Quality Assurance Teacher for post-16 education

3.

"Oh, so again, that can be challenging. These are the patients where we tend to say to them, we don't feel like you're ready for this. Um, we'll have patients that will come to us. And again, it could be an elderly lady who's very isolated. Um, she might, you know, have had a fall. She might have lost a confidence, or we're trying to get her out into the community to reduce her isolation, to go to strength building classes to reduce those falls. And then we're just met with "Oh, I'm not a sociable person. I don't I don't like mingling." So then we kind of have to dig into that and we'll say, oh, you know, tell me about how you met your husband or what? "You know, I actually met him at the local dance so many years ago." Well, you know, you're sociable. Then what happened? So and I will often say to people, "what are the barriers if you tell me, what is stopping you?" So to hear it from their perspective, and then I'll put a spin on it and I'll say, well, you've said this, this is what I'm hearing. Can you just let me know if that's correct?"

Code: Barrier characteristics
P17, Social prescriber

4.
"so I, I very much think that, uh, definitely from a GP angle, but also from my research is that, um, in terms of kind of mental health, well-being, um, it is so stigmatised in some cultures and it is so wrapped up in the social determinants of health that any support mechanisms to allow people to access things that will support them with their wider determinants is definitely beneficial. Um, so a lot of them do not want medication. They do not necessarily perceive themselves to have a mental illness, if they're distressed because they feel distressed because of the situation that they find themselves in, um, and any human would feel distressed having lost X, Y, Z and being in such a hostile environment. So, um, it is when we think about mental health, then the medical model is just not, is not the answer in any way. They may come to a medical model or approach a medical person because that's where, they don't know where else to go. (Person 1: Yeah.) Um, uh, and there's so much in terms of kind of somatisation as well, like, you know, the number that present and this isn't just from asylum seekers and refugees, it’s all quite, lots of different cultures. They may present with unexplained tummy pain, headaches, chest pain, fear, sleeplessness, you know, all of those kind of things. And most of it is a somatisation of that distress of where they find themselves. So, um, I, you know, well-being is 100% is, you know, is related to all those other aspects, but particularly I think, in this group, from what I've learned, is a lot to do with culture, connection, spirituality."
Code: Barrier characteristics
P14, GP and lead for local health stream initiative

5.
Person 2: "I think there are people who are seriously traumatized, with mental health issues, which could have even predated their journey. You know, people with Autism, people with learning disabilities, people with, or people with acquired head injuries, those you know, those sorts of experiences kind of make it very difficult to work alongside people who are there trying to support you."
Code: Barrier characteristics
P12, Founder and research coordinator

50. Local communities

1.
"I like the concept of social prescribing in that it recognises that, you know, loneliness and um, social exclusion and difficulties are being included and difficult. The difficulty, like a client of mine, joined, uh, a dance class. She loves to dance and she took two buses and went to a dance class, you know, on a very, on a very limited income. And she called me that evening and said I was the only black face there. You didn't tell me, you know."
Code: Local communities
P22, Social prescriber

2.
"It's horrendous because it's, it's, um, it's as if society puts a voice in their head that constantly shouts at them how inadequate they are and how they should be thankful for what they've been given. And it's just so important to challenge those narratives from whatever, uh, organisations, charities, whatever, um, movements there are, because it's, we're talking about the UK, right? We're talking about the country that colonised however many other countries and stole from other countries, and then they get people from Ghana and Nigeria and then, no, sorry. No. Yeah. We screwed your country, but you can't come

now. And then the people that come then are told that here, and yeah, they believe it. And they think that this culture is better than theirs, and they think like they adopt certain ways of doing things because they think this is the right way. There's no spaces where they can be told that no, no, no. There. Talk to me about your culture. I want to know. I want to know how do you do things in Nigeria. How like there's so much richness in, in in that, but there's no space for them to share that it's, there's this imposed narrative that is the Western way of doing things is the most advanced and this sort of like narrative of progress and how we're ahead of all the countries rather than this is just not okay."

Code: Local communities
P7, Social prescriber

3.

"And if you look into it, as I have done on many occasions, trying to find some kind of loophole, you'll find that although colleges are assessed by Ofsted, the DfE doesn't really have any control over them. They're independent businesses. Um, they don't have to, the way schools do, prove that they've tried techniques to support children before kicking them out, because it's not statutory. Post 16 education is statutory in that a law has been passed saying that you must be in education, employment or training post 16. Um, but it's, there's no way to apply that, there's no way to hold anyone responsible if children aren't and colleges are not local authority run and they're not run by the government, and we can't do anything, and if they want to kick a kid out, they'll kick the kid out. And they don't have to have a reason for it any more than an employer. No, in fact, less than an employer. An employer might be sued for discrimination. Yeah, theoretically so might a college. And I have asked my manager to do this, to take the, uh, the college I'm talking about to court multiple times, but the local authority won't take a college that it is part funding to court. So, it's not a possibility. What we really need is a parent of a refugee child, a professional qualified parent of a refugee child who knows the British legal system somehow, an education system to take them to court for discrimination. Because we can't do it or won't do it or whatever because but, but it's actively racist. So college I would say."

Code: Local communities
P16, Quality Assurance Teacher for post-16 education

4.

"Um, but, but I get it. We have had direct, um, hositility. Um, we have had hostility from parents before. Okay. So there's a school in (city), um, who a big secondary on a very white working class council estate in (city). And, um, we started working with them, they sent out a letter or put it on the end of a newsletter saying we are working towards a School of Sanctuary accreditation. And honestly, they had parents writing in the next day, “but I'm going to take out my child if you dare become a school of sanctuary”. I mean, this is the school as well that had, um, a far-right parent group that were handing out flyers to the year elevens to say, boycott the prom because it was being held at (name) Hotel, which is where some of the Afghan families were staying. So that is the hostility, I think, from the school. I mean, school staff on the whole are very nice and open and liberal and leftie kind of. Um, yeah. But it's the parents they're working with and the children that bring in the attitude but mean children again, I don't think have as strong opinions as parents, but it's often the parents, I'd say bring the hostility."

Code: Local communities
P19, Schools coordinator

5.

Person 2: "Yes, we have done from time to time. Okay. Um, for I don't know whether you because if you're living in Germany, you may not have been made aware, but, um, three little girls, were.

Person 1: I know about it.

Person 2: All right. It was so bad. People that didn't even live there then came marching. They came to the (town). So then had the police saying, you know, the community police. I had their direct number, mobile number. We took a load of measures to make sure that people couldn't see into the building. We were very quiet on social media. Yeah, we, we didn't say we closed. We didn't say we were open. But because staff have to, uh, work mobile, so people were still being supported like that. And we, uh, very much an emergency thing that if, if for any reason people were, you know, because we're right on the, on the main road where the other organisations aren't, we were. Um, so we, we covered all the, all the glass with, with, with paper or curtains or whatever, so they couldn't look into a building at all. Uh, and we had a plan as to where we needed to be if something was going to happen."

Code: Local communities
P18, CEO

6.

Person 2: "But, but like I say we're lucky in (city name), like in (other town) there's an organisation called (charity name) who's been going for years and years. And they're an amazing organisation, not just for people seeking asylum, but um, for, you know, there's lots of Eastern European migrants, economic migrants in um, in (sub-region). It's a very, very deprived area. (Person 1: Mhm.) And they do community lunches, English classes, loads of advocacy work, loads of legal advice. They're just brilliant. They've been really well-established in the community. But since Reform came in, um, they've not been able to help hold their community lunches. They've put CCTV in their staff's houses because of negative people following them home.

Person 1: Oh my word.

Person 2: For an organisation that that supports migrants. Um, so, you know, it is toxic in places. It's, it's horrific. Um, because, you know, the CEO of (charity name) came to a refugee and asylum seeker research event in (big city), and she was just saying, you guys have no idea how lucky you are to be able to hold your community lunches and do what you keep doing to support the community, because we're having to do it on the on the, on the on the sly."

Code: Local communities
P14, GP and lead for local health stream initiative

7.

"But unfortunately, um, the theory was that the work would be read by distinguished writers who would read it more beautifully than our writers possibly could read it for themselves because of their accents and their pronunciation and everything. And that what ended up happening was often that it was read by people who were also not native English speakers, and who hadn't even bothered to practice, so often it wasn't actually read any better than they could have done it themselves and they weren't given… I mean, they were allowed to sit in the audience, but there was no possibility for them to meet the writers or to be acknowledged or to answer questions. It was quite demeaning in a way."

Code: Local communities
P10, Writing project coordinator

51. Challenging relationships

1.

"So I had such meeting with the people with that department to not push too hard those people, especially, especially some of them are elderly, that, uh, very close to pension and then, uh, to learn English is also so difficult for those of such age. So, uh, yeah, I certainly recommend how to behave with, uh, with those people instead of pushing and sending them to work. Yeah, some of them are not physically fit for work, and they're still pushing those people to work. And I said, even if I, if I have a business, I'm not going to employ even he's Afghan and I'm Afghan because I can, I can, I can help them give them one week £100, but I cannot employ them because my job is a physical, uh, job. So he cannot be physically fit for the job. So I had such meeting with those people to, to behave differently with the community members, uh, or approach different, differently instead of pushing to find the job. No one going to give the job to a 60 year-old man without even, uh, physically fit for the job and not only English, but body, but physically. Uh, yeah, we, we do such things. But, uh, something is up, a lot of lot of time. We speak to the job centre and, uh, specifically about the specifically cases that I said, yeah, this guy is looking for work. Uh, and I'm aware of that. He is looking for work, and you have to believe that. But if you find such a job for the guy, so he going to work, but he can't find the job because we don't have the job sometime. Yeah. Uh, I complain one day on the, on that meeting that I just mentioned. That the job centre sending people to us to find the job. So that's the job centre. What's the meaning of job centre? Because you are the job centre. Yeah. I mean all the jobs are there… So why are you sending people to find job for the organisation. We are not the job centre. We are social, social organisation. So the people were laughing. I said, why are you sending people out to find the job? So instead we have to send people to you to find a job because you call, called yourself the job centre."

Code: Challenging relationships
P5, Charity director

2.

"And I think the other one that I said I think was yeah gatekeeping organisations who for whatever reason feel they have to hold the ties very strongly. And that I think, is to do with all those factors we've just talked about, about funding, about the vulnerability of the population they work with, with very difficult experiences around services in the past, the voluntary sector feeling like you're ignored or not supported by statutory organisations. And I think there's another element of that which is around the power that some, some people hold within voluntary sector when it's with this population that they feel the need to protect them, um, in a way that becomes quite paternalistic actually, which they may not themselves identify, that they're holding that sort of paternalistic role and actually are filtering, filtering on behalf of people who, who perhaps don't have the ability to speak for themselves, um, and who perhaps just feel very challenged by, by the research process and, and how it might reveal that virtually."

Code: Challenging relationships
P4, Senior researcher

3.

"I find it very difficult with colleges. I've found it very difficult with colleges. Colleges I think are frustrated because they set their course level at how many children are here in September and by, you know, January, you've got hundreds more kids who want to be on the course. So then if you see a child not engaging for whatever reason, they're looking around them going, we've got 60 kids or 100 kids or 200 kids who would want to be doing this and you're not attending. So what's the point? But I have found in the past that the biggest college in our area, the college that almost all have children I've had to go to is actively cruel

in the way that it treats their unaccompanied asylum-seeking children, and because it has a whole separate campus for ESOL and separate course leads for ESOL and therefore separate rules for ESOL, what you've got is this kind of de facto discrimination, where kids who are white and British and studying construction, um, might have their attendance explained away, they'll have attendance meetings and they'll have support put in place and they'll have… whereas our unaccompanied asylum seeking children, it's disciplinary one, disciplinary two, disciplinary three, you're gone. You're gone and you can't apply again next year. The end."

Code: Challenging relationships
P16, Quality Assurance Teacher for post-16 education

4.

"Um, but, but I get it. We have had direct, um, hositility. Um, we have had hostility from parents before. Okay. So there's a school in (city), um, who a big secondary on a very white working class council estate in (city). And, um, we started working with them, they sent out a letter or put it on the end of a newsletter saying we are working towards a School of Sanctuary accreditation. And honestly, they had parents writing in the next day, "but I'm going to take out my child if you dare become a school of sanctuary". I mean, this is the school as well that had, um, a far-right parent group that were handing out flyers to the year elevens to say, boycott the prom because it was being held at (name) Hotel, which is where some of the Afghan families were staying. So that is the hostility, I think, from the school. I mean, school staff on the whole are very nice and open and liberal and leftie kind of. Um, yeah. But it's the parents they're working with and the children that bring in the attitude but mean children again, I don't think have as strong opinions as parents, but it's often the parents, I'd say bring the hostility."

Code: Challenging relationships
P19, Schools coordinator

5.

"Absolutely. So there's been a few barriers from some activity providers around, uh, I would say blatant racism and negativity towards the refugees. Um, comments made like, well we want them to go home, don't we? Um, so they're willing to support and they've got all these lovely things and then they're like, but really we want them to go home, don't we? So there's that kind of like, I don't know if I want my young person to go on your project when you're talking like that. So that was really difficult. Um, and I'm not um, but then I would try not in a, an aggressive way, but I would come back and say, well, they do want to be at home, but unfortunately their towns are flattened and this is where they're trapped. Um, so I was pretty clear about that. Um, and some have kind of shaped up."

Code: Challenging relationships
P2, Youth service manager

52. Rural location

1.

"They're also at the stage of doing the feasibility trial, and the learning from those two different sites was how different things are, how contextually, in realist terms, it makes a very big difference how the context is and to how peer support workers can work and how things can move forward. [county A] County Council heard about the work we're doing in [city I] and the work we were doing in [city B], and was very interested because suddenly they were dealing with asylum hotels in small villages or market towns where the local population had no experience of this, where the services had no experience with this, didn't know what to do. And so they were really interested to be involved in a future funding bid.

That maybe will roll out the peer support model to other sites. So that's ongoing at the moment."

Code: Rural location
P4, Senior researcher

2.

"So it's a military site in, in or an ex-military site in a very remote location in rural (region), um, which was opened as kind of asylum accommodation, um, not strictly detention because people technically can leave, um, but it's very isolated. So by, by nature people are kind of trapped in, in the site."

Code: Rural location
P21, Advocacy manager and primary care lead

3.

"So they put a little package together. Um, and a lot of them are rural, so it's about transport a lot of the time. Some host families would be willing to drop them and pick them up. Um, sometimes the, uh, parents have access to a car, but sometimes that's not available. So it's about looking at what's around and looking at the school just to put, um, the children into activities as well."

Code: Rural location
P2, Youth service manager

4.

"People who don't, well say, well, they don't speak English and they sit in their hotel. They may go a little bit out of the way. And sadly, that hotel is very close to an absolutely gorgeous beach. But a lot of the kids are so traumatised with their journey, they don't want to go anywhere near the beach because they remember, you know, the, the trauma is extensive, so there are those people, but then there are also people who don't want that."

Code: Rural location
P18, CEO

5.

"Um, but yeah, so we tend to the way that our structure works is we will have a referral meeting on a Monday. Um, we will go through all the referrals that have come through. Um, we'll have a little chat about them. We'll have a chat with the team to say, you know, um, do you feel comfortable with this person or we'll give each other advice if it's someone that we know, um, personally, because the (town) is quite small."

Code: Rural location
P17, Social prescriber

6.

"So one is called (charity) and one is called (charity). Um, so they can help a lot with, you know, um, health checks. They can help with immigration law, employment. They have activities on um. The issue is if the person is already in contact with those services, there is a lack of sort of support in the (region) for what else they need. So there's no point me, um, referring, you know, um, um, a mother who was recently given birth to a, um, a baby and tots group, a mom and tots group, if she can't speak English and is going to feel more isolated in a crowded room. Um, so it can be a little bit challenging in that sense if they, if they have already accessed and aware of those, um, places in, it's a bit well, where do we go from here? But alternatively, if they don't know of those services it can be great."

Code: Rural location
P17, Social prescriber

7.

"Um, if someone is an asylum seeker outside that town, um, it could be like a two hour walk. So this lady in particular had back issues. She couldn't afford to get the bus. So again, she was quite isolated in terms of she couldn't access that support. So and it would be things such as, um, food banks that will have a food bank in there, particularly halal food for people that need those sorts of things. But she could not get there to access that sort of support. Again, it's where does social prescribing fit into this, and I personally find the hardest thing is having to turn around to someone and say, unfortunately, there's nothing I can do. Um, it's something that I personally struggle with because a lot of people are in a social prescribing role, are quite empathetic, and want to do their best. Um, so yeah, accessing um services when you are further away is particularly harder."

Code: Rural location
P17, Social prescriber

8.

"Yeah, as I've been saying, there's a lot of good services and support out there. But how do people access it? You know, if you yeah. You know, if you're stuck isolated at a hotel, some hotels are miles away. I mean, I'm aware of a hotel. There's a hotel which accommodates asylum seekers down and just down under (other city). And I think it's about I don't know, that's seven or eight miles outside. It's in the middle of nowhere. Yeah."

Code: Rural location
P11, Founder and CEO

53. Narrative critique

1.

"I think endless signposting can feel overwhelming and impersonal. Some social prescribers hand folk a leaflet and call it a day. I also do not rate time-limited services that offer short 'recovery' courses, and believe people seeking sanctuary often need months if not years to cultivate a sense of safety and confidence, especially in our current migrant-hating racist climate, peddled by the rich and powerful wanting to deflect from the issues that actually affect us."

Code: Narrative critique
P23, Volunteer

2.

"This is and this is very, very specific to migrant populations because they are not integrated in the system because we don't live in a society that supports integration of migrants. And this is the way it is. And it's, and they feel, the problem is that they feel they are the problem. This is where the psychological harm camps within systems that are abusive and racist, is that they really make people believe that there is something wrong with them. And they come to us with shame, with feelings of inadequacy, with feelings of I don't deserve. And we see that when people are, so I see a lot of people who claim PIP which is the benefit health disability benefit here. And they, they feel ashamed. So when they have to stop working. So migrants and asylum seekers and refugees, they have these feelings that they have to give back to the UK because the UK has given them the luxury of living a safe life. And it's like, whoa, uh. It's not, they don't feel entitled to any support. They don't feel entitled to anything because they feel like they're stealing. Like this narrative of, you're stealing our jobs. You're still like, this racist… All these constant, um, things that they're bombarded with, they believe them. Not all of them. The most they do. And when they become very ill and they have to claim benefits because they can't keep working, they feel worthless. They feel like they're taking advantage of the system that, no, I don't want to I don't want to do that. You see. No no no no no. I've always worked."

Code: Narrative critique
P7, Social prescriber

3.

"It's horrendous because it's, it's, um, it's as if society puts a voice in their head that constantly shouts at them how inadequate they are and how they should be thankful for what they've been given. And it's just so important to challenge those narratives from whatever, uh, organisations, charities, whatever, um, movements there are, because it's, we're talking about the UK, right? We're talking about the country that colonised however many other countries and stole from other countries, and then they get people from Ghana and Nigeria and then, no, sorry. No. Yeah. We screwed your country, but you can't come now. And then the people that come then are told that here, and yeah, they believe it. And they think that this culture is better than theirs, and they think like they adopt certain ways of doing things because they think this is the right way. There's no spaces where they can be told that no, no, no. There. Talk to me about your culture. I want to know. I want to know how do you do things in Nigeria. How like there's so much richness in, in in that, but there's no space for them to share that it's, there's this imposed narrative that is the Western way of doing things is the most advanced and this sort of like narrative of progress and how we're ahead of all the countries rather than this is just not okay."

Code: Narrative critique
P7, Social prescriber

4.

"For an organisation like ours, but also at the same time, we don't want it only to be that. And the other the history of this subject, of migration and displacement, you know, the, the infinite going back, you know infinite… As long as mankind's history, history of this... so it is also really important to engage because this is not just about people that've just arrived on a boat from the channel. That's a kind of political framing. It's not accurate. So it's also important for us to bring that and also to say it's not just about people who have come and claimed asylum. It's also we can connect this subject to broader conversations about migration, about diversity. About difference about race and then also about climate. Um, about mental health. All of these things are points of points of connection and I think make particular sense for a creative organisation. So that's what we also, are mindful of not, of being quite open with our language."

Code: Narrative critique
P6, Producer

5.

Person 2: "Yeah, I often, I often actually just approach the press and I'm like, can I write 500 words for you? This school has done amazing things with these Ukrainians, and then always link in the fact that it's a challenging time for the Ukrainians with this and, you know, trying to meet, I try to do quite a lot of work with media, prepare the game.

Person 1: I was about to say, you're pushing back an alternative narrative, aren't you, when you do that?

Person 2: Absolutely. Yeah."

Code: Narrative critique
P19, Schools coordinator

6.

"And I think that for us to be able to actually surprise people with a different view of what an asylum seeker or refugee or a survivor is, and to say, you know, these are people who've given up everything they ever had and everything they ever knew and left behind everybody that they ever cared about and taken a completely risky, dangerous journey and arrived absolutely empty handed. And look at them now. I think that that is something that is really

important to do, to, to counter the, the narrative of, of the sort of beaten down, broken, defeated person who needs our help and actually present them, as you know, these are people who can add, you know, they don't just add their cooking skills or their gardening skills or whatever it is, you know, they enormously enrich, you know, they're coming here offering themselves, offering all the riches and boundaries that they contain within themselves to us. And we're just like, you know, no, we don't want that. You know, we're going to keep you in a state where you're completely immobilised and you're paralysed and you're immiserated and you're impoverished and you, you can barely sort of draw breath, barely keep, keep your belly, have the courage even to carry on. We're going to keep you in this state for as long as you possibly can, you know, until, with any luck, you'll go away again. It just seems to me absolutely insane. And I think the more you can actually, I don't understand why our government doesn't tell the truth. I don't understand why nobody is saying refugees and migrants bring much more to the economy than they take. They bring much more to the economy than most native people do, actually, to be honest. And all they want is to be given an opportunity to give back and to add to the community and to enrich our, the plat of our cultural life and, and, and bring to it the special thing that makes them who they are. And I get so mad that that isn't acknowledged. And I suppose that in whatever small way we're able to contribute to that and change that narrative and present these people as the sort of shining stars they are. Uh, that's what I feel our work is all about."

Code: Narrative critique
P10, Writing project coordinator

54. Media

1.

"Um, so yeah, the media coverage that we had was, was fairly positive because it was drawing attention to the fact that, you know, there's a medical service on site and we're still outside in this van. Um, but obviously others, yeah, not so, not so positive, kind of interviews with the local residents and saying, you know, got people wandering around and doing all sorts and stuff. And, you know, that's obviously not true."

Code: Media
P21, Advocacy manager and primary care lead

2.

"And when ITV approached us, um, in Refugee Week, they wanted to do a piece on a school that just become a school of sanctuary and had established a new, new to English provision and had a massive impact on pupil outcomes and whole school culture. Um, and you're just never sure when the film crew come in, what angle, you know, but they didn't flip it around. They interviewed lots of people, interviewed me, but it was a really positive, like four minute, 6:00 news piece on the benefits that, um, having new to English speakers bring to the school. And the important thing I said to them was like, you can come in, but the, the key people you need to interview, yes, you need to interview the new English students that have come here, but you've got to interview some of the very local (city) born white people essentially, and have that voice, because that voice is often missing from the narrative. And they interviewed this girl. That was just brilliant. She couldn't have been more (city), but who spoke so fondly about how wonderful it had been to have this person and this person from this country then in (city)."

Code: Media
P19, Schools coordinator

3.

"But for example. So public health wanted to showcase, I presume, uh, the money that they're giving us for the migrant community wellbeing team. They interviewed a staff

member and they interviewed a service user. They took a picture and both were happy with that picture. So that was then published and that came onto social media. There was a lot of negative. So and you're saying you need to say, you know, really, you could almost feel the hatred in those people. You know, the way they were responding. But we never respond to stuff like that because in two days there's something else that's happening. And it's died down again."

Code: Media
P18, CEO

4.

"I suppose. I mean, again, it's a sort of cliché. It's been said before, but I do think that in all the conversations and the endless reams of, um, press and everything about refugees and asylum seekers, you know, you very, very rarely, you very rarely hear their own voices. And when you do, you know, you tend to, there tends to be a sort of cliché that perpetuated that, you know, the asylum seeker or refugees, they're sort of sad, helpless person with no agency and no dignity, sitting in a sort of bus shelter, staring out at the endless rain and, and you know their lives are sort of hopeless, you know. And I think that what we try to do is we try to present, we try to say, these are people who've survived things that would have killed most of us. These are people who have survived things that we would, that would, that we would, the most of us, you or I would probably not have survived and have gone on to become, you know, fighters for their future and people who are joyful and generous and dignified."

Code: Media
P10, Writing project coordinator

55. Politics and system critique

1.

"I could see that people seeking sanctuary faced multiple barriers and stigmas including lack of financial aid, being cut off from family, friends or those who share their mother tongue and culture, the hostile asylum-seeking process in the UK, racism, Islamophobia, stigma stoked by tabloid newspapers and the UK government. Over the years this has gotten progressively worse, with people we work with regularly being threatened with being “sent home”, including vulnerable children."

Code: Politics and system critique
P23, Volunteer

2.

"There are currently no safe routes to the UK for the Palestinians to escape the genocide in Gaza - this sends out a clear message that Palestinians are not welcome, and has a devastating impact on those we already support, because they can feel the hostility towards difference and diversity, even in a diverse city such as (city). The government is our biggest barrier to us supporting some of the most beautiful and wonderful people I have had the privilege of spending time with. Sometimes we build a relationship with someone and help to connect them in the community, only for them to be moved to another city hours away against their will."

Code: Politics and system critique
P23, Volunteer

3.

"When I, because I see when I was at (charity), I used to see people who were just about to get status because I was sorting out their paperwork, people who had been without status for a very long time. But now I'm, I'm getting to see the other side of it. And people at

(charity), we all feel very happy when someone got status and we thought, okay, that's it. They're sorted now because their legal status is so linked to. Yeah, welfare access to housing and access to everything. So we're like oh that's it. But it's over. And the thing about how humans work and it's not that easy. Your situation, your material situation will change for sure. But mentally, psychologically and from a trauma perspective, they're stuck. They feel isolated. They're not able to open up. Um, still, I mean, obviously more than before, but it's still a huge process of integration that, or they don't get much support. They it's just about, well, just get on with it. Just go work. I mean, be a citizen, right? It's like, well no actually. And not only go be a citizen, but go be a citizen and be grateful that we let you be here, right? And renew your visa every two and a half years and pay the Home Office £2,000 every two and a half years to renew your visa until you get the right to apply for a permanent residence. And then after that, you can apply for citizenship and maybe you feel proud to be British. The whole thing is just so abusive, the whole migrants' journey. Yeah, it's so inhumane. And they internalise that and they don't feel worthy of anything. This, of course, is not all migrants feel like that. But this has been my experience working with people for over eight years, that there is a huge sense of unworthiness and I don't deserve, which leads them to burn out. What I see a lot now because I mean, in a healthcare setting, I see people with chronic anxiety, depression and PTSD. They don't know what to do. They've all always worked because they felt that was the way to prove to the UK that they were not here to steal anything, I'm here to contribute to the economy, and I'm here to show you that I am worthy of your acceptance."

Code: Politics and system critique
P7, Social prescriber

8.

4.

"So I think that's why, that's what makes me so passionate about schools. I feel like schools are filling the gaps. Yeah, that community has been lost. And where, you know, small businesses, small cafes, perhaps have struggled in Covid and disappeared and community centres have disappeared… um, you know, the school where I am governor, they open their kitchens every holiday to let families who are living in hotels, use their cooking facilities because they can't cook. And three out of five of the weeks that are the summer holidays, they're running, um, holiday, sort of free holiday clubs. So in the most deprived area, taking them on beach trips and the whole families too, they're taking the families to like, Whitby, um, which is amazing. They've applied for a grant to do it, and that's how they're doing it. But it's still teachers getting holidays and getting paid a bit."

Code: Politics and system critique
P19, Schools coordinator

5.

"But if they're a single male or a single female, yeah, they have to be out of the premises within, uh, four weeks. And we all know within four weeks you will not have your universal credit and you definitely won't have any property. And the criteria that are being used to determine whether a person is vulnerable is very different from what we call vulnerable. Because we feel that people like that are much more prone to modern slavery. Uh, to sex trafficking, you know, all of those things, because if somebody is offering them a a bed for the night when it's freezing outside. They will say yes and then they own them. So we're very much aware of those things as well."

Code: Politics and system critique
P18, CEO

6.

"I think yes, it is, uh, a good thing to have this idea of being able to connect to different services because, um, the GPs can't do that. There's no way. Um. I think the problem is like the inequity of the service that, um, like I said, you got each person coming, each social prescriber coming with their own background, their own knowledge, their own interests. Um, and it means that you'll, you'll be getting a very different experience depending on which social prescriber you go to. Um, and you might go to a social prescriber who's like, oh, this there's nothing that we can offer you. You go to another one and they're like, oh, I actually know about this very very niche thing that, um you know, go try this thing out. Because ultimately no one can have all the knowledge. Um. So there is an inequity there, I suppose that maybe that's, you know, it's just a frustration of something that is very hard to fix."

Code: Politics and system critique
P1, Social Prescriber

7.

"Yeah, yeah. And yeah. And it takes lots, you know, lots of lots of different kinds of, of, of of support. Um, You know, we, you know, we just have to be realistic. We're only here because, you know, because the system is broken. You know, because the system doesn't, you know, the system doesn't, doesn't work, doesn’t support people effectively enough. You know, that's, that's the reason why we're here. That's the reason why, you know, many other, uh, community, uh, refugee support projects exist as well. You know, to be honest with you. We would all rather know it's not be here where we'd rather that people where were much better, much better supported. You know that, uh, you know that we'd rather that transport poverty, you know, you know, wasn't such a big, such a big issue, you know, amongst, uh, amongst asylum seekers. Uh, you know, so in, in many ways, uh, you know, with recognise and particularly myself coming from that public, public health background as well, you know, I very much recognise that that's what's, what's really required and needed, you know, is that more upstream kind of kind of approach. You know, the what we are providing is, you know, it's very much a more of a Band-Aid kind of approach, like, like, like many other services as well. But then, you know, if you're not, if you're not doing."

Code: Politics and system critique
P11, Founder and CEO

8.

"And I think that for us to be able to actually surprise people with a different view of what an asylum seeker or refugee or a survivor is, and to say, you know, these are people who've given up everything they ever had and everything they ever knew and left behind everybody that they ever cared about and taken a completely risky, dangerous journey and arrived absolutely empty handed. And look at them now. I think that that is something that is really important to do, to, to counter the, the narrative of, of the sort of beaten down, broken, defeated person who needs our help and actually present them, as you know, these are people who can add, you know, they don't just add their cooking skills or their gardening skills or whatever it is, you know, they enormously enrich, you know, they're coming here offering themselves, offering all the riches and boundaries that they contain within themselves to us. And we're just like, you know, no, we don't want that. You know, we're going to keep you in a state where you're completely immobilised and you're paralysed and you're immiserated and you're impoverished and you, you can barely sort of draw breath, barely keep, keep your belly, have the courage even to carry on. We're going to keep you in this state for as long as you possibly can, you know, until, with any luck, you'll go away again. It just seems to me absolutely insane. And I think the more you can actually, I don't understand why our government doesn't tell the truth. I don't understand why nobody is saying refugees and migrants bring much more to the economy than they take. They bring much more to the economy than most native people do, actually, to be honest. And all they want is to be given an opportunity to give back and to add to the community and to enrich

our, the plat of our cultural life and, and, and bring to it the special thing that makes them who they are. And I get so mad that that isn't acknowledged. And I suppose that in whatever small way we're able to contribute to that and change that narrative and present these people as the sort of shining stars they are. Uh, that's what I feel our work is all about."

Code: Politics and system critique
P10, Writing project coordinator

9.

"it runs through everything we do. Yeah, because. #00:42:07-5#

Person 1: It's always in the room. #00:42:08-1#

Person 2: Everything. It's always in the room. And everything about the women's lives is affected by, um, violence. So yeah, because of the way they're treated in this country, the way they're, they're spoken to by housing official. So much is an act of violence against them. So it's a constant act of resistance to push back against them."

Code: Politics and system critique
P9, Drama programme founder and producer/director

56. Refugee allowances

1.

"But one thing that I found when I moved from (charity) to (charity) is that migrants are pretty much invisible to the (charity). So this is, I mean, how I see it, and I even complained to the CEO about it, not complained, but like raised the issue with the CEO. So (charity) is a huge charity, um, that covers hundreds of locations across the whole country in the UK, and it's their own family support workers mental health support. Great services. But then and they have a grand scheme as well. And there's a lot of financial support. But none of their grants are for NRPF which is no recourse to public funds. So anyone that hasn't got a visa or even people that have a visa, that they have any type of condition on their visa, that means they have no access to public funds, which includes benefits in housing. So I asked at some point in one induction or I don't know, I had a chance to ask questions to the CEO, and I said, well, have you got any services for the migrant community? And he said, no, it's not needed. And I thought, what do you mean it's not needed? It's like, well, we don't have that speciality, there's other charities specialising in that. And I'm like, well, you say you're taking care of the most vulnerable families in the UK. I'm pretty sure the most vulnerable families are migrant families. And that was it."

Code: Refugee allowances
P7, Social prescriber

2.

Person 2: "I mean, I could keep going for hours, as you can see, but, um, there is something that I think is important and that in, in terms of health care and migrants and social prescribing, because it is in a health care setting, what you will, what I find a lot is migrants being denied secondary care. So in the UK, um, migrants have access, undocumented migrants sorry, um refused asylum seekers, they have access to primary care without them, but they don't have access to secondary care. They have to pay for them with even if they're homeless and destitute. So what you will find is either people with huge debts. So I would find, uh, the single mums that I would help from Nigeria and Ghana. They would have £10,000 in debt from their births, giving birth. So that's a huge problem that we as social prescriber will encounter that, um, 100%. Because if you're at a GP, some, you will see someone that's undocumented and open, because they feel safe, they will open up to you and you have to… and they will come with debt and they're destitute and they cannot pay

that debt. And then that debt is taken against them in their immigration case. So the Home Office can refuse an application if someone has debt with the NHS. But it's, it's a cycle of violence that's absolutely horrendous.

Person 1: So yeah.

Person 2: Um, that's something that I think it's important, um, to be aware of in, like the connection between social prescribing, healthcare and migrants. There, there's always that, um, that scenario that people don't know what to do with. And it's important that they refer people to migrant charities because there's some things that can be done. There's ways to advocate for those people."

Code: Refugee allowances
P7, Social prescriber

3.

"I just have a patient now, one of the patients I have, she's Colombian, she's got breast cancer and she's been refused treatment. Treatment has stopped because her, um, she doesn't have status. So the NHS said, oh, we can't. Either you pay or we don't operate and she doesn't have money, so. And she can't go back either. There she goes, she's just being told to stay there without, without treatment. In the UK. So she's been told by the there's a team within the NHS called the overseas team, which is the one that issues the bills."

Code: Refugee allowances
P7, Social prescriber

4.

"So if you did want to leave, you'd have to, you know, pass the security on the gate and then it's on a country road. So walking to the nearest town village, so it's in (town) village. Um, it's about half an hour. You've got nothing to do there. You get £8 a week, you know, not, um, not great."

Code: Refugee allowances
P21, Advocacy manager and primary care lead

5.

"I, I think that the one thing I haven't spoken about is the way that the government has dealt with the process. I don't agree with the visa system at all for our Ukrainian refugees, the state of a lot of their places to tell them that they will never have settlement here, like permanent settlement, they are just seen as visitors and that is that. I think, you know, some children were born here. Some children came here when they were two years old. They're not going to know their home country if they aren't going back any time soon, they're at school here. They've made friends. They're settled. If you if you if you offer to house refugees, then you have got to have an expectation that they that you support them and you don't just chuck them back at the first time that they can go back because, you know, um, like anyone that travels to a different country and stays there for a long time, it ends up becoming their home or their second home. And I don't I don't feel that we have the right to do that to people, and I am against it. Um, and that's with any refugee that comes to this country. Um, I feel that they have they should have an opportunity to be able to apply for permanent residency if they want to stay, because they're not all going to want to stay. You know, the ones that are from Kiev and like other big cities that have a, you know, they're going to want to go home. You know, you don't need to place that restriction on them and you don't need to make it hard for them. There are all these deadlines that were coming up that were like, you've got to get that form in, otherwise you're going to, you know, where they're going to go. They can't go home. Where are they going to go? So I think it's really unfair and it's and it's unethical and it's I think it's racist. And I think that's, you know, just how the government, you know, reacts in that way."

Code: Refugee allowances
P2, Youth service manager

6.

Person 2: "That the women or even the men, who ever just cooking in the house can go in the adjacent room and we've got sliding doors, so they are still with the children, but it's safe. Um, they do the cooking and they can eat as a family at least once a week. Because, yeah, you know, we're looking at health. You know, those kids are being brought up on fast food.

Person 1: Oh, well, yeah. You know, it's the question of what is possible, then in a travel lodge room, for instance, right? Where you have no cooking facilities.

Person 2: The only thing they're allowed to do is having a kettle. Some like, for example, sneaked in toaster and then they smelt it and they're in trouble. And then what do they expect these families to do. Yeah, exactly."

Code: Refugee allowances
P18, CEO

7.

"So they have the isolation they're only living on about, you know, £30 a week, um, to get through to them. Um, so a lot of the referrals that we have do have common sort of, um, needs, food, um, you know, if you've got issues with accessing, um, things for the children, accessing clothes, accessing community support, to which, whether that be their religion or someone that speaks their language to um, and even accessing things such as the NHS itself, um, is quite challenging."

Code: Refugee allowances
P17, Social prescriber

8.

Person 2: "What a lot of people don't realise is that if you're an asylum seeker, uh, regardless of your, your skills and your qualifications, uh, you're not allowed to work, you know, so you can't seek, uh, employment. Uh, people also don't realise that you also can't claim state benefits. So you are given an allowance, a daily allowance of I think it moving somewhere between seven, seven and eight pound.

Person 1: And that's what I wanted to say as well. It's, it's horrendously low. Yeah.

Person 2: Yeah. And that's all your, all your daily living expenses. So much for your food and your clothes and your toiletries and expenses, the public transport costs. So in (city) I think are a day bus pass is between £5.50 and and £7.7. So that's almost your daily allowance, you know. So we we're certainly aware of many people who are having to make difficult choices on a daily basis about whether, you know, they're able to feed themselves and their families or afford the expense of public transport costs to access basic, essential health services, and, you know, and, and community activities. So eh yeah, so, yeah, (name) was inspiration behind, uh, behind the project."

Code: Refugee allowances
P11, Founder and CEO

57. Hostile environment

1.

"I mean the child I spoke to you about before, I will be careful not to identify him, but I will say that he had tuberculosis that went undiagnosed. His extremely low attendance was

considered to be by his doctor, down to the fact that he had undiagnosed tuberculosis. I still had to actively fight for them not to kick him out. And we had to pay for our educational psychologist to go and do an assessment, because he'd been there for two years and not made any progress. And they tried to kick him out both years. He'd not made any progress. I got our EP to go in who wrote up a report that said something like, he would really benefit from some 1 to 1 support, and they therefore kicked him out, saying that they couldn't meet his need because they didn't want to offer 1 to 1 support. They didn't ask us because in our homes, we do have workers who will give 1 to 1 support. They didn't ask if we could put anything in place. They didn't apply for any HCP, even so that he could maybe go somewhere that was a bit more specialist to support him. They did nothing. They just kicked him out, like they will be actively cruel."

Code: Hostile environment
P16, Quality Assurance Teacher for post-16 education

2.

"And, and then we work with people who are, um, new to the area, the system, the country, um, they are vulnerable. They are made to feel like they don't belong in the society. They have been told they don't belong in society. Um, I mean, they have, they have a piece of paper to prove that they don't belong in the society. And then yet we often treat these people in the way that we're like, let me just send you off to a place and assume that you're confident enough to walk there and take yourself there and attend this meeting of this group of people or whatever it is, um, as if you belong. And, and like our clients, are the definition of people who don't respond to a poster on the wall. Um, because people who respond to a post on the wall are not like they are not our target group. They are people who already feel like that thing belongs to me. I am confident enough to go there. Um, I understand where what, when. Um, so that, that's immediately not our target group."

Code: Hostile environment
P15, Wellbeing manager

58. Role of funders

1.

"Obviously, we have to do all these reports for funders. Which can be helpful, sometimes, they help you kind of crystallise a viewpoint and maybe even understand some of the impact that you might be having."

Code: Role of funders
P6, Producer

2.

Person 2: "And then they produce what's called, county council have adopted the Sterling scale measurement tool. Um which is a well-being scale. Um but we struggled with it. It doesn't translate very well and it's aimed at children aged eight and above. Okay. So those really little ones, it didn't really make much sense to them. So we try to get around it because we had to use it. And we still have to use it as a measurement tool for reporting.

Person 2: As part of the grant arrangement.

Person 1: Yes, yes. In our contract terms, that is how we measure."

Code: Role of funders
P2, Youth service manager

3.

"Um, there was also, again, because of those numbers of how many people need to go through the door for the funding, uh, in terms of what's been said by the funders, um, someone said, like, you know, you're supposed to have a certain number of appointments that are just one off appointments, so that you just get the numbers. (Person 1: Mhm) I don't know exactly what whether there is a specific number, but I guess there was a kind of drive towards trying to get at least a certain number, like percentage of your clients, where it is just 1 or 2 appointments, like sort something out quickly and then go. Um, and then there will be other people and you would get a sense of it, of like, okay, this is a client where there is a little quick fix-ish that we can do, and there's other clients where it's like, not quite sure how to support, like, like, let's monitor this over time and see what happens."

Code: Role of funders
P1, Social Prescriber

4.

"You know, it's it's difficult for me, you know, and and it's, it's really, really difficult to put funding in place for, for social research for that type of evaluation as well. You know, funders are just not interested in giving you any, I can't kind of think of actually, in the last 7 years, uh, very, very rarely, you know, will there be funding, funding out there to fund any kind of social research or evaluation. It's just, which, which is a big gap and a big, a big problem because particularly, because every funder, of course, naturally, if somebody gives you money, you know. You know, and you know, it's an aid to the project, you know, you know, people will want a six-month report and some need of funding report. People want to know what difference you know, they their funding has, has made. And that's absolutely perfectly, you know reasonable. However, I think a lot of funders have not most funders have quite unrealistic, unrealistic expectations. You know, and you know, I just think they don't entirely appreciate, you know, that good research, a good evaluation takes, takes time and resources, but they're not giving it to you. You know, they might give you a grant for a small grant of of £20,000, you know, but they have big expectations about, you know, uh, you know, sort of, uh, evaluation and case studies and, you know, and, and that's and that's difficult and that's and that's valid."

Code: Role of funders
P11, Founder and CEO

5.

"So and we're very lucky because we're not beholden to a whole lot of, um, uh, what's it called, um, precarious funding. We can actually say to funders, we're not going to apply for that because your evaluation, we don't agree with your deal or that doesn't align with our values or whatever. So in theory, normally the benefits should always be for the women, not the organisation or the, you know, so the benefits of any activities should sit with the women, therefore what we measure is the benefit to the women."

Code: Role of funders
P9, Drama programme founder and producer/director

59. Reflection on evaluation

1.

"Um, I do this and I do, um, I do SIKA. Yeah. Um, which is, you know, I have to fill out forms to, to, um, to justify the funding or to. Yeah, whatever the word is. Um, to be honest, a lot of the time I forget to do those things because people are telling a story, you know, and it's a long, complex story. And oftentimes it's, you know, it's a lot of trauma. Yeah. Um, and so I try to let it flow as much as I can and then focus in on maybe a, you know, a concern, or 1 or 2 concerns from what I found."

Code: Reflection on evaluation
P22, Social prescriber

2.

"It's an ongoing question, and in some ways I might, in all honesty, say not. We don't do it very well. (Person 1: Okay.) But my mitigation of that is that it's a very hard thing to do and people who appear to do it well, I wouldn't necessarily believe what they say, um, because I've seen a lot of evaluation. I mean, virtually no one reads evaluations, in my experience. I've seen a lot. And what they really tell you, um, we obviously capture data of kind of people who come, we gather feedback in various ways. Um, you know, we do case studies. We, you know, we have to report back, um, on kind of facts and figures. Um, and that is all obviously relevant"

Code: Reflection on evaluation
P6, Producer

3.

"And, and it's not the same as proving training to ex quantity of people leads to x reduction in deaths. So that it's not, that's not that simplistic is it? But, uh, I think we've got really good evidence that that network of training approaches build the confidence to intervene in a really positive way. And uh, and uh, and, you know, apply that to, you know, the, the (name) uni work's been written up. It says in many reports that the, the growth of the training capacity and some, some feedback from their colleagues as well. Being aware where our energies is going. And of course, it's not a static resource. Sometimes it's growing, sometimes it's shrinking. So you try to keep adapting all the time."

Code: Reflection on evaluation
P20, Regional manager for health inequities

4.

"Meaningful evaluation, picking up your question, right? Um, yeah, I would say with sufficient time and, um, the space as well as time to do some of this reflecting on what you're trying to do. You would build in not just a technical evaluation of the work you're doing with whatever partners, but also build in the evaluation of the system around it that makes the decisions about fund making, that makes decisions about mainstreaming of activity, about how mainstream services then interact with what you're trying to do. But you often don't have that. It ought not to be a luxury, but it is luxury or you often don't have anything like that, the ability to, to look at the system impact on what you're trying to do."

Code: Reflection on evaluation
P20, Regional manager for health inequities

5.

"Um, there were definitely some social prescribers who are clearly sending it out a lot, and then a lot who weren't. Um, I think, again, this is part of the challenge of the job. It's just like back-to-back clients all day. Very little time for admin. And for a lot of practitioners, the sort of evaluation processes just completely fell by the wayside to be honest. (Person 1: Yeah, well it's just -) The thing you could get from, like, the records that we're keeping (Person 1: Yeah) um, again, in terms of like what other firewalls that are coming in and for what, stuff like that, um, but obviously that requires everyone to be keeping extremely, um, diligent things and again that can be hard when you've got such high client numbers and the ideal would be that you're getting all that data from the NHS systems, from um, the EMIS system. But unfortunately, our service isn't able to draw the data from the NHS system. So we were having to replicate all the data from NHS system onto our own system. And so, I'm sure, like there will be, it just won't be as detailed or like we won't be able to really get the rich data that we might be able to do something with, to be honest… And I think we need to research the ethics around data collection, um, and I felt really strongly like if we're collecting this data, we need to be doing something with it, right? (Person 1: Yeah). Um, and so I was, I would be saying like how is this then going to ever get communicated back to the clients?

What do we do with this data? Who who's looking at it? Um, so and again, when there's all this, these time pressures, it's very difficult to work out who's going to have the time to go and sit down and do all this stuff. So I know that I was like really flying the flag, ‘so let's do something with this data’. And, you know, and coming up with ideas of what we're doing with it. Um, but, uh, I think that's always the challenge. And I think that's probably across a lot of these sectors. Probably too much data collection going on in some places and too little going in other places, or rather just like the wrong data is being collected. Um, all I think there's a lot that needs to be done.""

Code: Reflection on evaluation
P1, Social Prescriber

6.

"Um, um, and with the evaluation there's always the tension of, again, quantifying. We helped 1 million women in (city) or we helped one woman in south (city). But in fact, actually, you could you could be supporting three women really, really meaningfully and effectively. Yeah. But that for a lot of funders there's just three in a box, you know, whereas if you could put 150, yeah, that would be better. But actually, what, what, what, what is what does better look like? What is what is the better that we're measuring? So and I'm really lucky at the organisation I work for that people are really, um, willing to engage with that level, you know, to, to be critical about evaluation, which is, which is good and also to be quite brave. In that quite often in funding reports, we say actually this didn't happen, this thing happened instead. And in fact (institution) asked us to come in and they wanted us to be part of a big evaluation day and be trained and, and so this was my old boss, (name), and she said, well, why could some of the women from my drama group come in and run? Because we've been I've been doing some facilitation training with them, some of the women who've been coming for ages. And so in fact, (institution), um, they weren't sure about it, but um, but in fact, the day went really well. Um, some of the women from the drama group facilitated some of the discussions and it was great and everyone loved it, you know, because it was it was actually doing the thing. Yeah, they were all saying, how do we do it? And you're like, well, this is how you do it. So again showing, not talking yet. Showing not, not yeah."

Code: Reflection on evaluation
P9, Drama programme founder and producer/director

60. Informal

1.

Person 2: "where we're trying to understand the reach of our programmes and in but even in like obvious ways, so in (event), are there more organisations taking part? Have we got more press coverage?

Person 1: Yep.

Person 2: That can kind of tell you, and we… and to a certain extent, it's then on what people say, are we getting the feedback that people are saying this in the refugee network, has this been effective?

Person 1: Yeah.

Person 2: And I think if people, if we start and not so many people are coming or people are saying, well, this didn't quite work. So I think openness to conversations is actually really important. Having lots of meetings when we're encouraging people to say what they think and being responsive to that. And that's a bit of a partner level and a wider level."

Code: Informal

P6, Producer

2.

"I have done specific bits of feedback for training, for example. So when I go to training, I always bring forms with me there and then and say, here you go, fill it in now. Um, yeah, that seems to work better than an email, which people just don't really, really do. Um, last year, two years into the project, I did my first sort of impact report. So I do have a spreadsheet of data where I like, this, we've done this many assemblies, you've done this like numerical, kind of like, um, how many we've done of this. And I keep also just a word document of any quotes that anybody has said to me throughout the year when they did an assembly or something was done, or that Syrian refugee was brilliant about that, or somebody with lived experience who's been on our advisory panel who talked about the value that I keep. Quotes. Yeah. And then the impact report basically looked like sort of figures and more quotes. Um, that's as far as I've got really um, I do think, yeah. It's exhausting with funding applications, having to constantly project outcomes. Um, and then I do find when I wrote my end of grant reports, there's enough of I have enough data to be able to feed back. And then I've kind of anecdotal stories about, you know, we supported this Afghan boy in Catholic school. The teacher quotes. So teacher quotes are very helpful."

Code: Informal
P19, Schools coordinator

3.

"Um, we collect obviously participant data. Who was there, how many people were there? Um, we collect anecdotal quotes. Um. And then we sometimes write up case studies of person who attends on week one and or attends for many weeks, and is very reserved and quiet, and then, and then, um, all of a sudden is somewhat confident in a canoe."

Code: Informal
P15, Wellbeing manager

4.

Person 2: "Um, yeah, it is mostly, I think, feedback from the volunteers and from, you know, and we actively ask people, um, on, on a regular basis and I, you know, I think we try really hard to listen and pick up on any even slight suggestions because sometimes there's just a little comment and if you grab it quick enough and dig, you can get a bit more. Um, so I think it's just being aware of listening for what people are thinking and feeling about it, so that I would see we haven't really done, um, uh, formal evaluations. Yeah. Um, but no, I did write something down and let me have a quick. What number was that again?

Person 1: Gosh, that's under three, titled measuring impact. Yeah.

Person 2: That's it. Um, so yeah, observation and feedback would be the main things. But, um, I think the fact that that we, you know, we have a core group of people who keep returning. Uh, including one gentleman who works away at sea for months at a time. When he's at home, he always comes. Um, so I think, um, we're doing something right. Um, and we, we did a, we project, side project from the Language cafe. We planted some trees in a local park. Um, and, you know, the we have, uh, a language café WhatsApp as well as the regional one. So, um, you know, people were posting, um, photos and, you know, they obviously had a really good time. So I think things like that, that kind of engagement."

Code: Informal
P13, Charity chair

5.

"but yeah, we also use it, you know, we can, we can reach out to people and say, you know, yeah, yeah, you know, we want to do a wee bit of, a wee bit of evaluation and find out how

people are getting on with, with their bikes and how you're using your bikes and what you're using your bikes for. You sort of get some feedback, feedback that way. I mean, you'll see on our website some really nice, nice quotes there, which highlight, you know, just the yeah, you know, the wide-ranging outcomes. You know that was sort of the thing to do before we do some you know, we've developed some, some case studies as well. But again, I mean, to be honest with you. Good evaluation takes, takes time and resources."

Code: Informal
P11, Founder and CEO

61. Formal/validated scales

1.

Person 2: "Um, um, we use the ONS4, which is a wellbeing questionnaire. Yeah.

Person 1: Okay.

Person 2: But it's, uh, it's not representative of the reality. It might be, um, it's quite a tick box type of thing, so. It's just for questions about, oh, um, do you feel anxious now? How anxious did you feel yesterday? How are you with your life? And then you would ask the same questions at the end. Um, but the reality of it is none of us get to ask those questions at the end, because the end almost never comes, because people kick back, because there's a housing crisis, there's a welfare crisis. There's a mental health crisis. There's the loneliness crisis. It's unlikely that people will come to the service and then say, I'm happy."

Code: Formal/validated scales
P7, Social prescriber

2.

"Yes. I think that is definitely something. I think that's why something like the, where the core screening kind of is less useful, I guess, because it's, although it's talking about like frequencies of how these things occur, doing that through with the telephone interpreter on the phone, who's also not there in person is really difficult. And some of the wording of the questions is one in particular, which is like, I can't remember what it is now. Um, but there's one question. It will come to me, which is basically you can't, it doesn't really make sense in English. And even when you translate it, it's really… I'm going to have to look it up because that's going to annoy me very much. Yeah, it's a question for “talking to people has felt too much for me”. So “over the last week, how often has talking to people felt too much” being like, what does that mean? Like, if someone asked me that I'd be like, oh, I don't know, like that's… So then that being translated like the interpreter doesn't really know how to translate that, so a lot of those questions yeah are quite strange. Like some of the more kind of physical ones like, oh I've had difficulty sleeping then. Yeah that's easier to answer. But yeah, the others are a little bit tricky especially. Yeah when being translated"

Code: Formal/validated scales
P21, Advocacy manager and primary care lead

3.

"It's a really interesting one, isn't it, because I'm going to talk about this nationally. Um, so (name), for example, has a school, the sanctuary Survey, that she asks all schools when they've completed the award to complete, but she gets about 30% completion rate, um, just by local leaders badgering, badgering, badgering, badgering. It's very hard to get feedback"

Code: Formal/validated scales
P19, Schools coordinator

4.

"And then they produce what's called, county council have adopted the Sterling scale measurement tool. Um which is a well-being scale. Um but we struggled with it. It doesn't translate very well and it's aimed at children aged eight and above. Okay. So those really little ones, it didn't really make much sense to them. So we try to get around it because we had to use it. And we still have to use it as a measurement tool for reporting."

Code: Formal/validated scales
P2, Youth service manager

5.

Person 2: "When I came into service, I could see, so basically, the contract, um, determines that we need to be getting ONS4 scores. That is the, um, that's one of the key measures of impact. And what is the journey across? Do you know the ONS4?.

Person 1: I, I don't I, I will look it up. But you, please also give me a definition. I'm very happy to have one. Yeah.

Person 2: Um, one of the things that's great about it, is it's just full statements. Um. Because, you know, other mental health measurements that might be like the core ten or even the core 20, like much longer things. And in social prescribing, especially when you're seeing people and all these different levels, that might be very short term or whatever, having to ask loads and loads of questions, not possible, not possible. So ONS4 just four questions. Um. What are they? I've already forgotten. One is like, um, how happy did you feel yesterday? Maybe. Or how anxious did you feel yesterday? (Person 1: Okay) To what extent do you feel the things that you do in your life are worthwhile?"

Code: Formal/validated scales
P1, Social Prescriber

62. Project impact or changes brought about

1.

"I mean, one of the reasons that, uh, the women's group activities are running so successfully because of the relationship these women are made with the other staff, women, women, group staff, uh, to, to, uh, to be clear and honestly, that these women, despite they working two days a week, uh, they've been in contact with women 24 seven. Uh, my colleagues are telling me that some of the women that are calling us at 7:00 on the morning as well, and we are not refusing to take their calls. Uh, and because of that relationship, even, uh, when we say that, uh, the (name) College reducing their fund and they're cutting off the fund, they're asking the student to come to the college, and they can’t provide such activities, uh, in a college for them as well. They didn't go because our colleagues were not there, so that mean the reason they are coming to our activity just because of the relationship they made with our staff and the confidence and, uh, uh, the confidentiality that we are we are holding."

Code: Project impact or changes brought about
P5, Charity director

2.

"Um, you know, we have one, um, one school two years ago that had 12 Afghan families in 2021 come to their school when, um, people fled to Kabul and one of those dads ended up becoming school governor. Um, and that was just really powerful because he could just, his English was really good, but he could translate messages and not just physically, but the meaning of things and why, um, to the Afghan population."

Code: Project impact or changes brought about
P19, Schools coordinator

3.

"Uh, because it's obviously, you know, that's how you recover from trauma is to then, you know, do something fun and, you know, become happier in your new environment. So, I think a lot of parents were caught up with housing, finances, what was going on with them, making sure their kids were placed in school. That activities bit, that went around the outside wasn't a priority. So we kind of took that away from the parent and made it a priority. And then that meant that they didn't have to deal with it. So I think that was really important for them as well and they're all so grateful."

Code: Project impact or changes brought about
P2, Youth service manager

4.

"Uh, yesterday we said goodbye to one of our staff. Yep. He came into this country is from Afghanistan, so to this country. So he already spoke some English. We volunteered in UN, another charity that was specifically just for asylum seekers and refugees. Um, he then did some work. I don't know whether you've heard of the (charity). So he, he then, uh, himself and I was part of a of a judging panel, if you like. So we then invited him to do an activity with us. He then applied for a job, and then he ended up, uh, while he was volunteering, ended up with a job, and then he's just left because he's now comfortable that he is setting up some of his own business. So we have people like that as well."

Code: Project impact or changes brought about
P18, CEO

5.

"Um. I happen to be working, somehow I happened to get loads, uh, queer, um, asylum seekers come to me in my job and I've spoken to my colleagues about this and 'you know that so random, we've got none'. Um, and I'm like, 'I bet you do have some'. But I think because I'm also queer, um, there's probably a degree to which I don't know that the clients were on a on a very, like human level feeling that or knowing that and would come out to me. And as soon as I know that, then I'm like, okay, I've got some very specific services I can signpost to or refer you to. Um, and that was actually really powerful. And generally, those were the times where I felt like, 'okay, I'm actually gonna do something', um, because the, uh, queer asylum seekers, there are some really good services out there, and the there's lesser waiting list as well. So that was good."

Code: Project impact or changes brought about
P1, Social Prescriber

63. Individual journey

1.

"A gentleman in his mid twenties, referred by an Occupational Therapist was extremely withdrawn and vulnerable when he arrived. We slowly built a relationship with him, encouraging him to come to sessions however he is feeling, as sometimes he felt he couldn't come if he was depressed and anxious. One time he messaged me to say he made it to the gate, but felt overwhelmed by his depression so walked home. Knowing this, I have since sent encouraging messages to him each week, and he now attends even when he is struggling.

Once he felt safe, he shared that he travelled to the UK by boat. Devastatingly, the boat capsized and he was in the water for hours without help. He watched every other person he was on the boat with die. As a result, he experiences nightmares, flashbacks, anxiety and depression, and is undergoing intensive weekly therapy and is on antidepressants.

Being at the farm has greatly helped him. Before the farm he was very isolated and didn’t feel able to socialise with others. We have seen him increase in confidence and he has told us that he feels happy and at home during our sessions. He can struggle to eat so we always make sure he is sent home with any surplus healthy lunch we have cooked. We have provided him with a certificate of participation, emphasising how much we value his presence and contributions to the farm, which will hopefully support his leave to remain and future employment."

Code: Individual journey
P23, Volunteer

2.

"So I'll do a pretty, um, simple, but not simple client. Um, so I met a woman from the (city) or, sorry, from the Democratic Republic of Congo. So I did my master's, um, um, rape services in, um, DRC. Um, compared to the (city) Rape Crisis Centre. That was that was my thesis. Um, so, um, I suppose I, I have an interest in, in, um, in that area. So I, uh, met a client, um, she was living in a she's living in a, in an IPAS centre. Um, it's a it's a comfortable, um, well-run centre. Uh, that was a hotel. Um, but clients cannot cook. So what they do, what they're, what's happening is they're, they're given a voucher. They buy pre-cooked or ready meals, um, reheat them and, uh, microwaves. That's all that's available at the moment. Um, she has eight children. Some are adults, some aren't. Um, and she was, um, captured, taken to the bush, um, you know, gang raped, repeatedly raped for a long time, and then eventually, you know, got away. So I met her at her time where she wanted to become. Her English was, um, little, and she wanted to become involved in, um, the community, but she didn't really know what that looked like. Um, so she was, I have to say, she was a little different in that she had a lot of family support. I don't often see that. Um, so she, um. Yeah, I think that family support was, was quite significant. She has a partner and she's very well supported. They were all separated, all got together again, which is a good outcome in terms of her story. Um, because that doesn't often happen. We have a lot of people who are, um, who have lost family. Yeah. Um, and so we, we met, we talked, she joined the English classes, she joined a women's group, she joined a jewellery making group. And she has then gone off on her own route. So she, she was she had never been to school. So that literacy problem. Um, so she also did a English and basic computer skills course here, and she was thrilled with herself. But in the midst of all of that, we talked about (city) Rape Crisis Centre and getting some support from there. And she said no, but she wanted to have better English. Um, she wants to be able to express herself more from the heart. Um, and that she would consider it later in life so that. Yeah, that was a journey uh for her. Other people's journeys aren't as you know, aren't as complete."

Code: Individual journey
P22, Social prescriber

3.

"So I have a couple of I have one client who's like the perfect social prescribing client who's just so brilliant and has just done so well. Um, his journey. So he came to me for help. You see, like, the reason why they are referred is like the, the tip of the iceberg. And then when you meet them like, boom. So he was referred to me for help with the PIP form, for him, for benefits advice. And then when I did the holistic assessment and we were chatting for like the whole session about what, what's really causing his depression and then loads of things came out. So essentially this is a 57-year old man, chronic pain, severe anxiety. Couldn't look at me in the eye shaking, um, very isolated. Wouldn't leave the house. Um, problems with hoarding as well at home, like different manifestations of mental health, entrenched mental health problems, um, no relationship with his daughters. Just very deep feelings of unworthiness. And he, he just came for the PIP form. So we sorted that out. But I was like,

listen, I can offer up to six sessions. You want to come next time? We can have a chat about all the things. He was really apprehensive like oh, no, I don't want this, this is a 57 year old year old man from a Caribbean heritage who's like, I don't care about systems and done with life. Like this is all. Like very closed off to, like, very cynical about the system and very not really believing that therapy or anything like that would help because he had a really difficult life. So. Okay, fine. Cool. I'll come for the next and then we, session by session, we, I tried to it's a very intuitive thing as well. And that's why the emotional support side of it is really important, because you sort of get a feeling of what the person is willing to try and not. And you have to, um, patience in terms of like, they're not ready to push. So like you have to offer things and then see how they feel about it and then know and then do they engage, then now maybe look at all the things. And in in his case, we had like 12 sessions, once a month. Um, and he went from that setting like that, that um, presentation that I just gave you to now where he sees his daughters pretty much every day. He's cleaned up his apartment. He he's claiming benefits. He's doing okay. He has a great relationship with his mom. He talks to the mum of her children, and they have an amicable relationship. He's volunteering and he's getting training to get into employment, at some point. He's getting mental health support. He's bright, like he comes in, he jokes. Like, I see him around. I used to be very close to (area of city). I would see him around like, hey (name), he's just joyful. He feels like he can. I mean, obviously he's not going to be like, oh, okay, I'm completely fulfilled by my life. But he engaged with services so well, just by that, having that hand-holding through the process and that space where it feels like there was nothing wrong with him, um, there's always options and there's always support. So the perfect case scenario in a social prescribing case is engagement with services like holding on to people and seeing what's possible for them and reflecting that back to them."

Code: Individual journey
P7, Social prescriber

4.

"We've had some wonderful like I remember now, actually, one chap who was initially a long time ago referred by the (refugee) team, um, to activities and this very quiet, um withdrawn, didn't ever like social engage with the rest of the group. Um, but he came week after week. He always turned up um, um, and once we went canoeing with him and he was terrified and, uh, and then he decided that he would go with the instructor and another person and sort of sit in the middle. Um, um, um, and it was quite a big thing for him to, like, step off the pontoon onto the canoe, into the middle. And he's just sat there holding the edges. And then he came again afterward, and he just, like, had blown himself in the canoe and took a paddle, um, towards a paddle. Um, Uh. So sometimes the most visible kind of difference you can see is in people who are maybe, um, less extroverted."

Code: Individual journey
P15, Wellbeing manager

5.

"Spirituality is a really difficult one, though, because I don't know how many social prescribers would be confident saying, I don't know, a big thing for you is like asking about spirituality, or asking about faith or, you know, like one of our project ambassadors was just moved by the, um, Home Office, um, from (city). He was really gutted to go, but he's moved to (city in another region). And I said to him, because the whole time he'd been here, he said, I just, I do. I do really miss my people. I miss my church. Yeah. Uh, and (city) is a small place. They don't have an Ethiopian Orthodox church, obviously. Sure. But yeah, he texted me and said, you sent me a picture of, um, uh, it was a, like a I don't think it was like a traditional meal. Yeah. They found in an Ethiopian restaurant in (new city) and he said, “And I found my church.” And he's like, I'm so happy. Yeah, you know, and I guarantee a lot of his like, you know, mental distress is going to be lowered because he's got that connection."

Code: Individual journey
P14, GP and lead for local health stream initiative

6.

"I mean the ones who really take to it, they develop a completely new personality. You know, they yeah, they I mean, this is a story I tell endlessly, but it just seems to me to be so sort of emblematic. We have a man who came to the group from a country in sub-Saharan Africa, a francophone country. So he arrived in this country with literally not a word of English and no money, and sort of met somebody at the mosque and slept on his sofa for a while, and eventually was referred to (charity). A very bright man. He'd been a teacher, but a science teacher in his own country and, um, absolutely passionate about it and no idea about literature and no idea about creative writing. He'd never done any creative writing, even at school. And so the first piece of writing I asked him to do, I said, can you describe your ideal day? And his ideal day consisted of, um, marking homework and planning the next day's lessons, and it was written in a very plain style. And so time went on, and a few weeks later we did a workshop with, I think about poetry, and I think the, the model that we use was Kipling's If, something like that, which was great, "If you could do, if you can do that", it's very easy to kind of grasp and follow. And so he wrote a poem in response to that, and he was like, "Oh, is that me? Does that come out of me? Is that in there somewhere?" Yeah, it was just amazing. And he then became this extraordinary poet. And it was like, it was like watching him growing into a new being. It was like midwifing a whole new side of his personality into being. It was such a privilege to, to help that happen."

Code: Individual journey
P10, Writing project coordinator

7.

"Oh, yes. There's a woman who started last term, and, I mean, she must be late 70s, doesn't speak any English at all. Been in the UK quite a long time, but under the radar, so doesn't have a status that we're just the legal people at the charity sorting that out for or trying to sort that out. So she's been invisible. And then and I was like, oh gosh, this is going to be difficult tonight. Yeah, that was very uncharitable of me to think that. But I was like, oh, you know, um, uh, well and then but actually she's, she's spectacular. You know, it took a few weeks and very silent, very just silent. And then, um, and again, that's this, this husky voice, you know, when someone doesn't use the voice, literally just not used, so we do a lot of, like, well, with the blowing up balloons, that's also about the lungs and the air and all the drama stuff. And then an amazing dancer and mover.

Person 1: Great. Yeah.

Person 2: And now so started coming all sort of shrouded up you know. And so the clothes, the clothing becomes more colourful, more personalised, you know, so this is what happened with this older lady. You know, she now she, she came last week. She had her hair done. It was all uh, and she was, she was, um, she's Orthodox Christian. And, and she wears, she's at the end of the last time she wore this big, it's like, amazingly cool, you know, like big chunky wooden beads like this with a big cross, like, you know, the kind of, oh, I don't, forgive my ignorance, like, oh yeah, yeah, a designed cross and then these cool trainers and like there's, and I swear to you look like she should be at the Met Gala ball. You know, I mean I was like, (name), you look so cool. Yeah. It's just like. But, you know, like, free in the movement and just, um, she still doesn't really say much, I think. I think she's not quite sure about me.

Person 1: Okay.

Person 2: She's like, I don't know who you are, lady.

Person 1: Yeah.

Person 2: Yeah, but we kind of have fun in this class, so I'm going to keep coming, you know? And then and she performed in the, um, the Christmas show for the other women. And she performed something about him. It was about mothers. Uh, um, it was like, I miss my son, you know, I've missed my grandchildren, you know, so learned very. And then we did that as a person with other women. So she was part of that."

Code: Individual journey
P9, Drama programme founder and producer/director

64. Social prescribing definition

1.

"Well, I'd have to read it. Um. Social prescribing is the provision of non-medical supports for people to enable them to improve their health, well-being through structured referral process and the establishment of close connections with both health care practitioners, community organisations. Link worker will create a person-centred assessment of the person's needs, agree on a co-created plan, connect people to local supports, meet the person's social, physical and emotional needs that may not be met by their GP. The social prescribing model is an integrated model of care that aligns with the social determinants of health. So how I see it is, is all of that. Um, I, I'm a I'm strong believer in social capital and social health, uh, and that having those enhance your health. Um, I, I understand the difficulty of making social connections, I suppose from a personal point of view, because I've, I've travelled like you've got to, you know, join new communities and it's not easy. And, you know, the, the idea of leaving your room in the IPAS centre and going down into a village nearby may sound very easy, but it's, it's actually a huge step."

Code: Social prescribing definition
P22, Social prescriber

2.

Person 2: "I think, I think it's a role that's evolving and has changed a lot throughout the years. So when it was created, it was a way to remove the load from GPs. That's how I understand it. It was created so people would come to the GP because they're isolated, they're bored. They don't know what to do with their life. That's the, the idea. And then GPs don't have time to deal with that. Um, and then this role was created so that people would have access to activities and community groups and, um, exercise things, but that, that's not true, at least not in (city area)."

Code: Social prescribing definition
P7, Social prescriber

3.

"And I would say also in the pathway of social prescribing, when we think of it as a non-medical intervention, supporting social determinants of health, helping to helping to address health inequalities."

Code: Social prescribing definition
P4, Senior researcher

4.

"I mean, I'm not a total expert on social prescribing at all. I just yeah, I in my work in primary care, I kind of naturally come across it, in my understanding of it is that it's kind of a holistic approach to reducing health inequalities. So referrals to other services that support people's

health and wellbeing and a lot of that actually can be linked to our work, particularly our advice line, because although the main aim is to get people registered with the GP, if people come with other issues, then we will refer to other organisations um, as well. So I guess, yeah, it's like a non-clinical part. Um, and yeah, referring to support and a practical support but also kind of more wellbeing, emotional mental health support as well."

Code: Social prescribing definition
P21, Advocacy manager and primary care lead

5.

"Yeah, I think is it sort of prescribing activities like exercise or gardening or such? So a more holistic approach to improving health. Yeah. And mental health. Um, physical, physical health. Yeah. Um, yeah. Things that aren't medicines necessarily."

Code: Social prescribing definition
P19, Schools coordinator

6.

"How do I understand it? I would understand it, as I said, sort of, um, as a model utilised by public health services, um, to send people off to another place to do things they can't do in an office."

Code: Social prescribing definition
P15, Wellbeing manager

7.

"Well, social prescribing, as I understand it, is that I know that there's this standard thing and I can't think of what it is now, even though it's on all of my reporting. Um, but it's a way of supporting, um, a client or a family with the assessing their needs and finding what out what their needs are and then obviously and their interests. And then through a range of tools we can signpost, refer, place, um, you know, get them to a place where they need to be. Normally when somebody is referred to you for social prescribing because they need help in some way. And it's about ensuring that their needs are met and if they can't be met by us, which generally isn't because it's a light touch, sort of, um, navigation like community navigator, that's a similar role, um, so our social prescribers, they, they provide a needs assessment, look at the whole family approach and yeah, just help the child or young person or client to be placed where they need to be to help them, you know, um, improve their well-being. Um, and just, um. Yeah, sort of meet the needs. So it's like a person focussed approach. And, um, needs based."

Code: Social prescribing definition
P2, Youth service manager

8.

"Okay, what it means for me is, because of my organisation. So that is that, um, sometimes going to a GP and getting tablets for being referred to somewhere else is not really the answer. You know, especially when we talk about mental health. And so a lot of our surgeries now have a social prescriber there. And so then it's almost like a brokerage service. You know you go to your GP and they say oh you know what. Um I don't necessarily want to put you on medication right now, but what you're telling me when we talk to so-and-so and so-and-so has a good map of everything that's available and they refer to organisations or, um, you know, wherever, people are, they're being supported in ways that isn't strictly clinical. Um, but we're supporting them for their wellness. So probably in a nutshell, how it is."

Code: Social prescribing definition
P18, CEO

9.

"Gosh, I mean, I get asked that question quite a lot, and sometimes it's hard to sum up. Um. It's, it is about having that conversation with the patients. So I always say it's about finding out what matters to them and giving them the time to talk about those issues. Um, it's a guide. It's almost like I'd say “friend” in quotation marks, because sometimes the lines can blur. Um, you know, some patients can become a bit too friendly. Some of them can become a bit too reliant. Um, but it's about having given that patient that time to have someone really listen to them that isn't a friend, that isn't a family member, and someone that is not going to judge them, and someone just to say that “that sounds hard Do you want to tell me a bit more or would you like some and support and guidance on this?” So I think that's probably how I would sum it, sum it up."

Code: Social prescribing definition
P17, Social prescriber

10.

“So it's not something I'd ever heard of. Um, I'll be honest with you. I haven't really researched it. Um. It's not, it's not a term I use. Um, or our organisation uses."

Code: Social prescribing definition
P9, Drama programme founder and producer/director

11.

"To be honest, I don't really know the official definition."

Code: Social prescribing definition
P8, Integration manager

65. Familiarity with social prescribing

1.

"Yeah, we haven't really thought about it in that way. And I don't think as an organisation or myself, we really know a lot about social prescribing and how it works."

Code: Familiarity with social prescribing
P6, Producer

2.

"Um, that, that social prescribers may be doing a really good job, I would have to see some of the, um, evidence, you know, of what they're saying is working well, but I don't necessarily, I've not ever been asked, could they come and do a survey with us? So it's those kind of things I think is lacking."

Code: Familiarity with social prescribing
P18, CEO

3.

"Really, all I know about it is, uh, what I read on Google."

Code: Familiarity with social prescribing
P13, Charity chair

4.

Person 2: "And, you know, I'm not aware of it happening, um, in the area, but I don't know, but I think it's a really good idea. Um, I, I like because, uh, for example, the lady yesterday who came and asked me to help her with the library, she had, she had a note from the doctor or the nurse, somebody, um, because it had NHS at the top and they had written, um, go to the library and get some books. Read them with your child.

Person 1: Okay. That might have been a, that actually might have been a social prescription from exactly what you've just said.

Person 2: There you go. That’s my first one."

Code: Familiarity with social prescribing
P13, Charity chair

5.

"I mean yeah, yeah I'm not going to say it's a bad thing. #01:08:47-4#

Person 1: No no no, but. #01:08:48-5#

Person 2: I don't know enough about it to comment."

Code: Familiarity with social prescribing
P9, Drama programme founder and producer/director

6.

"Kind of another example actually. And this, this particular prescriber context is quite a lot, but sometimes they're a bit, like, I wouldn't necessarily say a bit, you know, I don't know if the word is insensitive, but more just like, a bit much, like, for example, she once contacted us about the subject was like urgent action required. And it was like about, I think an Iranian client who was, like, suicidal and needed, like, urgent help. And it was just a little bit insensitive, inappropriate because it was like super urgent and just kind of out of the blue. And we didn't have that sort of capacity to help them."

Code: Familiarity with social prescribing
P8, Integration manager

66. Alignment with project work

1.

"Um, and then this role was created so that people would have access to activities and community groups and, um, exercise things, but that, that's not true, at least not in (city area). I mean, social prescribing is like full on caseworker role or you just have to like, get deep into social welfare, racism or all sorts of complexities come at you from left, right and centre. So I think there's a gap between what the NHS thinks social prescribing is and what the reality of this, of it actually is. So I think I mentioned that before. There's something about when, when you work with people in a helping decision, in a charity or frontline work, um, there's always a gap between the people that make the decisions and the people that do the work. And there's very little communication between both. So what the role is according to the NHS, it's not the truth of it."

Code: Alignment with project work
P7, Social prescriber

2.

"And I would say also in the pathway of social prescribing, when we think of it as a non-medical intervention, supporting social determinants of health, helping to helping to address health inequalities. All of that matches onto the [project A] project. I would say the journey in that you are referred by somebody, although you don't need to be referred by a health service professional, you know, um, uh, and not all social prescribing schemes require that either. So there we go. Um, you meet with somebody who's empathetic, who has a listening conversation, a guided conversation with you, which is very broad, which takes into account your whole person and, and and where you are and how you want to move forward and then meets with you a number of times. Looking to move you forward on some of the things that you've identified, perhaps signposting you, perhaps taking you to things. All of those things line up with social prescribing. I think the difference would be, um, how it's done in the NHS

in England, which is around, you know, quite often a health service referral, quite often a system level referral that goes on to medical records. We don't do that type of record keeping and that would be completely anathema in [project A], because people are so wary of records and where they're kept and why they're being kept and why questions being asked, and the whole traumatising nature of some, the way that some statutory services are called to work with them. So that's very different."

Code: Alignment with project work
P4, Senior researcher

3.

"Um, so I would say that social care, social workers, designated teachers at colleges, we at the (name) school, leaving care workers, carers, key workers from their home, all of these people are doing the social prescribing bit, presumably charities too. I know my kids don't have much interaction with them, but presumably charities are too. But I would say that health professionals, no actually CAMHS workers do it as well."

Code: Alignment with project work
P16, Quality Assurance Teacher for post-16 education

4.

"As you were talking, I was like, oh schools are a bit like social prescribers. So, I mean, got a school that became a school of sanctuary a few years ago, I'm actually now a governor for. They were the ones with the Afghan families, actually. They have, um, a hub, at the hub, which is separate sort of building next to the school, but run, run by the school. And, um, they have a women's English group. They have a slow cooker group. Um, they have, like a trip to the park to do laps around the park group. And actually they find lots of the mums who are too scared to go out, particularly when the Afghan mums were there a few years ago who were in the hotel and just the school gate was their safe place. That was it. Um, they used to take them to the park to just walk around and introduce them to the park, which gave them the confidence to do it. But it was also physical exercise but also help build community. Um, because they, they were also saying it is a very, very deprived areas, one of the most like high level, highest levels of unemployment and lowest life expectancy areas in the whole country. There's no, um, there's no community cafe. So they were saying, you know, school parents drop off kids and then want to talk, but there's no space to build those relationships. So the school had the hub where you can drop in for coffee afterwards and you can you can meet other people. And that for some parents, it's been a lifeline of meeting other people and then doing, doing activities. So yeah, I guess that's some kind of informal, it's social prescribing in a way, because it has really had a massive impact on because we, I was talking about whether that, that has an impact on pupil outcomes, which is the ultimate goal and actually does because it breaks the barriers down between them. Then they might be more likely to go into a reading session in school, you know, where parents are invited in because they've built confidence in the hub, which then has the impact on whether they might read to the child at home, and that improves outcomes very much."

Code: Alignment with project work
P19, Schools coordinator

5.

"No, but there is one that we use organizationally. But I don’t use social prescribing because obviously we've changed how…

Person 2: Well that, and that is exactly, that is what I was going to come to next. Is it a term that describes the work that you do?

Person 1: Well, it's under the social prescribing contract. But obviously we've had to change the way it's delivered. And I attend social prescribing um meetings for the county and a lot of

social prescribing projects are through the NHS services. They're based in doctor surgeries or through HE or hospices or places like that. Um, so I came on board and I set about [name of the NGO] and they were like, well, why is she here? It is social prescribing, but we've had to adapt the way we deliver. Although the the meaning of it is there, um, we still deliver a whole family approach. We still do a needs assessment. We still find out what those needs are and how best to, um, support people either back into the community or improve their health and wellbeing. But it is done in a different way."

Code: Alignment with project work
P2, Youth service manager

67. SP aspects - recommendations for improvement

1.

"I think, um, the only thing I probably would say is. I think social prescribing is a great model. I think it's a great framework. And I think the concept behind it is very, is very, um, it's very valuable. I think there is and also, uh, probably should look at maybe a key worker role as well as social prescribing role. Um, it's quite short term and I have I probably should have said this to I have referred some clients, um, or they have referred themselves. So we're, we're going to kind of cap it at three. But yeah, there are times when it could go on longer. I mean that that requires some reflection on my part as well in terms of, you know, discharging and, you know, keeping to the framework, but also the need if, if somebody is re referring to, uh, twice or three times, then there's a gap in service somewhere and maybe, maybe we should start looking at a key working role as well."

Code: SP aspects - recommendations for improvement
P22, Social prescriber

2.

"It's a space for you to feel safe and be human. So that's needed for everyone, really. All of us need it. But if someone's gone through such huge amounts of trauma in their past, gosh, I mean, it's so necessary. So in my view, I would make sure that there are services like this one outside of healthcare settings that people can access without feeling threatened. So food banks for example are a great example because the people might feel safe to get to food bank. But then how do they access the food bank without someone, do you see what I mean? So there's a link to service um access that, at some point someone has to hold space for those people to feel safe to access services. And if, if health care is not one of those settings, then there's going to be a community. Like, for example, many asylum seekers are allocated accommodation under section 4 or section 95 in hotels that are inhumane, like the conditions that people are made to live in, I have no words. In those settings, there are um staff in those in those hostels who could provide some signposting, who could easily say, look, you have the right to go to the GP, there's no harm, it's safe, you can go, but it doesn't happen because again, it's epidemic right. So there are tracks where we could have people signposting and provide information that would make access to services very easy, make people feel safe to do it, but we don't utilise it because again, it's, it's not that big of a problem to the right people who have powers make the decisions."

Code: SP aspects - recommendations for improvement
P7, Social prescriber

3.

"Yeah, I think first of all, we should have less people because it's for the workers. It's not sustainable because again, the way it's designed is like, oh, it's a very medical mindset, because it's a medical setting. Then they look at it like, oh, here's some slots, and then you see them, and then you let it go. But it's not like that. It's, there is emotional, there's a strong emotional connection that the patient makes with the worker, inevitably, because it's a

space where they're opening up about something emotional, something that matters to them. So it's not going to be, oh, here's this slot. You tell them the problem, you sort it out, signpost them and there you go, bye bye. That's how the timetable is designed to be. But the reality of it is not like that. So you have to balance that out. But you have to somehow work with a system that is not really reflective of the reality of the job. And that means you're seeing too many people, way more than what should be. Because in therapy or counselling, how many people do you see per day. Not 7 or 8. Not that many"

Code: SP aspects - recommendations for improvement
P7, Social prescriber

4.

"so I think specialist training is really important. And I also think this whole idea of cultural awareness, cultural sensitivity and understanding trauma, I understand trauma informed practice is something that social prescribing generally. A lot of people are trained in that because it's present throughout many populations. But I think for this population, the triggers, the triggers of trauma and, and, and what provokes, you know, mental health crises or mental health discomfort are quite specific. And if they if they don't have those, if they don't have that awareness and training and, and support really to work with those populations, I think that would be very challenging."

Code: SP aspects - recommendations for improvement
P4, Senior researcher

5.

"I think for a particular support side of things, if you have trained your social prescribing systems and the people that do that to be aware of and connected into a much broader range of community supports, including in a multicultural dimension and so on, then you've got the more mature response. If you've only got a sort of standard list that hasn't thought that side of it through, then you don't have that wider range of assets to draw from. So I think it comes down to how, how richly have you drawn your system up in the first place? Are you constantly looking to expand that? And, um, you know, I know that colleagues, sometimes they're really simple ideas are some of the strongest."

Code: SP aspects - recommendations for improvement
P20, Regional manager for health inequities

6.

"I think, I think often what can happen is that, um, that the health service that's involved in social prescribing will prescribe the activity much like a medicine, where it's sort of almost written on a paper, um, or in an email and that's that and there's little involvement and check-up of what it actually is. Whereas really if you want to, then you need to do what kind of what we've got going is that the person from that team comes and joins us. Like you really want to ensure that there's, you know, what you're getting into and people are benefiting from it, then you need to be there."

Code: SP aspects - recommendations for improvement
P15, Wellbeing manager

7.

"I mean, I think there are ways of improving it. Um, having better like directories of services that are, uh, shared across teams and stuff, um, so that everyone has a better awareness of what is out there. And requires, uh, it requires that a separate person who's probably not so like they're doing the clinical work to be out there, like researching all the stuff all the time. Um, and that's something that the current model does not, the funding model doesn't support like there's no even funding for management essentially. So to get funding for like an admin person, it just doesn't exist. Um, so yeah."

Code: SP aspects - recommendations for improvement

P1, Social Prescriber

8.

Person 2: "And it's so, um, I'm involved separately in a, in a social prescribing for, um, pregnant women living in, in the 20 most deprived areas of (region). Um, and so we've had to kind of learn about how social prescribing is, um, commissioned and provided across the whole of (region). And it's taken months to work out, um, you know how… Because it's really confusing. #00:12:41-6#

Person 1:Is it fragmented in the sense…

Person 2: Yeah. Yeah. Really fragmented. Yeah, yeah. And also, like, there's such a broad range as well, because I don't, I don't really need a social prescriber to just signpost. (Person 1: Yeah.) Um, because I could probably do that. I mean, I don't know all the organisations, but I, a lot of the time I need them to case manage a little bit. (Person 1: Yeah.) Um, and particularly in a group which is disempowered and main probably doesn't have any awareness of what social prescribing is. (Person 1: Yeah.) That's not going to happen in one project, in one appointment."

Code: SP aspects - recommendations for improvement
P14, GP and lead for local health stream initiative

9.

"Um, and I just sort of think with the social prescribing as well, like, I don't know how much in terms of trauma-informed practice and in terms of how they understand the role of trauma in how someone engages or doesn't engage or how, you know, for our social prescriber if people constantly miss appointments with her. And for me, if someone misses an appointment it's a flag for vulnerability. Usually I treat it in the same way I would if a child wasn't brought to an appointment. I'd look and see if they got care and support needs, language needs, got literacy needs doing have they got, you know, mental illness, what's going on for that person. Do I need to proactively ring them? And on the whole I do, yeah. So Um, and then I don't punish them for missing an appointment with me. So, you know, and that all of that is kind of trying to develop that trauma informed approach, but I don't know. I mean, this social prescriber, she never rings if they've missed the appointment, I don't know what she does for an hour. So it's like how do you… I don't know. Yeah. I agree, I'd be really interested to know whether they have any training, particularly in engagement patterns."

Code: SP aspects - recommendations for improvement
P14, GP and lead for local health stream initiative

10.

"So, you know, if, if I think if, uh, if you know, funders, you know, really value the role of the third sector in terms of social prescribing, then it needs greater, greater priority in terms of, in terms of funding for a particular type of, of funding as well, you know, so that has to be a longer term, longer term funding. You know, we, the, the third sector is just plagued with insecurity, uh, and a lot of burnout as well, because there's a constant pressure of, you know, having to having to put new funds in place, you know, sort of year on, year on year, you know, so particularly if you're a, uh, an employer as well. So, yeah, you know, that, that greater, you know, that, that, that greater political priority and, and investment bit of funding which is, you know, which is which is longer term, you know, at least three years, you know, there's a couple, a couple of, of examples in recent years where some funders are starting to get that message, and there's some three and five year funding pots about, but they're very, very few, and far, and far between. And actually usually what, what happens is when people start to offer three and five year funding at this time, it tends to be the same amount of

funds, but they're just offering it over, yeah, you know, so, so actually, yes, you might get three years funding, but every year you get smaller, smaller amounts of funding over that three years. Yeah. Uh, so it's just. Yeah. So it needs to be a greater investment, longer term funding also particular type of funding, not just project funding but also unrestricted funds. And, and core, and core funding as well, which is, which is really crucial, which are, you know, uh, many if not all social sector organisations really struggle with, you know, who are very much, you know, an important part of that social prescribing landscape. Uh, so, yeah, you know, that would be my, that would be my, my shout out. That's what I would be, uh, asking for, demanding."

Code: SP aspects - recommendations for improvement
P11, Founder and CEO

68. SP aspects - what needs to change

1.

"Yeah, I think that, I think that's it, I think um you know travel, travel costs uh support with travel leave cards. Um, um yeah. I mean I think if you're going to be seriously behind the idea of helping someone to leave their room. And this is my simple concept that is helping someone to leave their room and join a group and feel, feel valued and worthwhile in that group. Um, you need to have, you know, you need to have good sleep. You need to have proper nutrition. You need to have, um, good support. You need to have money. Um, it's a very temporary type of living, but it's long term, you know, so it's, you know, people are people are in IPAS centres for three or 4 or 5 years more, I believe it's more, um, yeah. And it's just the, the, the hits on your mental health and the hits on your self-worth. Yeah. Um, it's it's an enormous price to play today, really."

Code: SP aspects - what needs to change
P22, Social prescriber

2.

"So what I would change about the job is, I wouldn't change, I mean, obviously I would see less people so that we would have more time to actually help people and provide that emotional support without burning out, because all of us, like people, leave the job very quickly. You can, we're always recruiting and always leaving and always recruiting. So it's quite a dense, it's as I said, it's like five different jobs in one. Because you're all these things. You're also looking for activities. You're relating to third parties, all of this stuff."

Code: SP aspects - what needs to change
P7, Social prescriber

3.

"And when you have that type of job, and then a big part of the job is to signpost to services. And then you also have the same person that's doing all that emotional labour, do the research and create their own system to… And then be in contact with the agency is like whoa, whoa whoa whoa, hold on. Here's, like five different jobs. So I would if I were creating a social service for migrants, I would create a huge database with anything and everything available for, I would have one job post just for someone to research charities constantly. Everything that's out all the time. Everything that shuts down, shuts down. Everything that's created, everything that changes, referral procedures. That's your job. Research all that so that these people that are doing the emotional labour can have a clear brain like clear, at least in front of them, and just send people through to the thing. That, that's another thing."

Code: SP aspects - what needs to change
P7, Social prescriber

4.

Person 2: "But money is the big thing. And I heard a story about 30 GPS in (city) being practices being set up to do social prescribing a few years ago. That would have been a huge multi-million-pound investment. There was literally nothing given on the delivery side. For the social prescribing. Well that's not going to work, because there is not capacity out there at all.

Person 1: Correct.

Person 2: We're in a situation where there is no spare capacity for anyone. It really is at that point. Um, of course you might just happen across oh, there's this program happening. You can join it. But that it just doesn't work that anymore. Councils are not funding things. No one is funding things. So without money, it doesn't happen."

Code: SP aspects - what needs to change
P6, Producer

5.

"so I think specialist training is really important. And I also think this whole idea of cultural awareness, cultural sensitivity and understanding trauma, I understand trauma informed practice is something that social prescribing generally. A lot of people are trained in that because it's present throughout many populations. But I think for this population, the triggers, the triggers of trauma and, and, and what provokes, you know, mental health crises or mental health discomfort are quite specific. And if they if they don't have those, if they don't have that awareness and training and, and support really to work with those populations, I think that would be very challenging."

Code: SP aspects - what needs to change
P4, Senior researcher

6.

"However, the only flaw I would say is a lot of the training for social prescribing is around, you know, mental health, isolation, loneliness, which all does fit under, um, what people that are asylum seekers can, um, experience. But the training itself, there isn't a lot on culture. And I guess, you know, there are so many different cultures. Um, unfortunately, the two surgery, the two services I did speak about, I did try and get in contact with them to sort of have a bit more of a stronger relationship. Um, I haven't been successful in breaking down that barrier. Um, although I have attended one of the services. But it may be the case if, you know, having, like an open day, go in there, having that conversation and being like, this is a two-way street, you know, we can come to you with these people. You can come to us."

Code: SP aspects - what needs to change
P17, Social prescriber

7.

"I think so, I mean, yes, like, right now how it usually works is, like, we have, like, a general admin email. I think it's the same way you've reached out. (Person 1: Yeah I did yeah) We also have a landline number and we usually direct them to like a referral form that's on our website. Um, and it usually works, but um, and it's mainly one of my colleagues who checks the form, but, um, you know, there are cases some times when, like, these organisations will get frustrated, we don't reply quickly enough, or I mean, in some cases we if in some cases if we just cannot help that particular case, we try to tell them. But yeah, no, it is challenging. Yeah I think there needs to be be a bit more patience on, patience on their end."

Code: SP aspects - what needs to change
P8, Integration manager
_final

69. SP aspects - less fitting

1.

Person 1: "Does it always feel that way, that it is possible to match people up well to their needs, or do you see kind of cracks and gaps there as well that need to be filled.

Person 2: Yeah, there are not. You mean whether there are services to send people to?

Person 1: Exactly. How do you send them on?

Person 2: So that's, that's a whole other like complication. So sometimes there are services that are great. Sometimes there's nothing. Or sometimes there is a service that we all know is amazing, but it's, it's at capacity. And they're not going to take anyone else on like (charity) for example. They're always at capacity because it's a great free service. But yeah, the demand is way bigger than, than what's available."

Code: SP aspects - less fitting
P7, Social prescriber

2.

"And I would say that's one of the issues of personalisation, that it sort of draws us towards thinking about an individual rather than thinking about an individual within their network and what the you know, what the arrangements are between them and their family and their wider community. But, I think when a whole family has just arrived and dropped on your doorstep, I think that's incredibly challenging for a social prescriber to deal with. Unless they have extremely good connections with the voluntary sector organisations who might be able to support. And then also those voluntary sector organisations don't have capacity to suddenly deal with, you know, 250 young men or, or their families or you know, so I think I think that makes it different in that it's and then it's massively challenging."

Code: SP aspects - less fitting
P4, Senior researcher

3.

Person 2: "I went to the GP recently in the local authority where I live, and it was interesting that there are quite a lot of leaflets around that could kind of inform and support social prescribing. I don't think any of the GPs have ever had the time, taken the time to stop and read them, but they do. They are there. They're there on the tables. But remember, we're talking about kids who don't speak very much English and don't read very much English, so it's not very helpful for them.

Person 1: We could also pose the question of who, who engages with brochures and leaflets, right? We can also ask that question.

Person 2: Um, yeah, professionals do. I do, because I'm a professional and I want to go and see what's on offer here, that isn't an offer in the area I work in. That's, that's it basically."

Code: SP aspects - less fitting
P16, Quality Assurance Teacher for post-16 education

4.

"But I think it's more beneficial if you can, you can build those links, back to your overall theme of social prescribing, and you have people who know to how to navigate that. And so this one work from, um, different parts of the UK, I think (city area), is on this and the fascinating work about almost like a dose response of if you give community members multiple choices about the support they can get and the idea that you can try lots of different

things until you find something that's close fit for you, rather than be told, we're going to prescribe you X and if you don't like it, that's, you've had your, had your, your, your say um. But again, if your community isn't well resourced and strong enough to give people choices, then I think you can draw, my biggest concern in this whole territory is false negatives. You know, the kind of reductionist mentality that says, we've got the social prescribing project. We give communities one option that didn't work for some people. So this is, this whole thing is a waste of time. I think that's just bad science and bad thinking."

Code: SP aspects - less fitting
P20, Regional manager for health inequities

5.

"Um, the problem I have with it is that there's often very little quality control and you can run an activity for refugees and asylum seekers that is deeply harmful. Um, and, um, but nobody checks on that. If you say you're running football for refugees. You know immediately uh sort of like “wow, well isn't that great”. Um there is no there is no quality standard of how that's run, and how inclusive the practice is, how professionally it's done, or is it the sort of like backhanded offer? Um, is it is it therefore sometimes quite retraumatizing for people. Because, because they will always be treated as, as the second-class citizen, that's getting the second class offers. Um. Uh, so I think there's, there is a lot to be said about social prescribing in that it needs to be it needs to be controlled. Like, I wouldn't send my clients to other organisations unless I really know what's going on there."

Code: SP aspects - less fitting
P15, Wellbeing manager

6.

"Invariably, the social prescribers are white British. But, um, you know, for our community, um, and that I think that has been very evident in the hospital asking for ethnic minority um advocate, for public health providing that service. It's because, you know, and we work with people like (charity), we’ve worked with really well because they already had those positions in, in the hospital, but they were saying, listen, we're not reaching anybody and I'm sure there must be people in hospital that need support. But you know, we're not we're not able to support them. So that's why because the hospital advocacy projects, so it's more and more recognised as well. And I think, you know that social prescribing again, I think the pandemic has given lots of really positive examples that social prescribing works way better, outside in the community."

Code: SP aspects - less fitting
P18, CEO

7.

"Um, and then because there are just so many services that, um, you can't access if you don't have recourse to public funds. Um in particular, so with my service, (area name) social prescribing that was, it's a part of, um, wide wider charity (charity name) who offer periodic grants all the time. But none of those grants are open to people with no recourse to public funds. Um, and (charity name) itself doesn't have any, uh, services for refugees and asylum seekers, which I think is a massive oversight and it doesn't make sense to me. Um, I don't know how you put that into the your research. Um, but yeah, it just I can often just feel this frustration of working in organisations where we're like, we're here to help the most vulnerable families, and we're here to help the most vulnerable whatever's, um, but we also don't support people who don't have access to public funds. (Person 1: Mhm) I'm like, cause yeah, there's just a real gap there (Person 1: Mhm), um, across many, many different services."

Code: SP aspects - less fitting
P1, Social Prescriber

70. SP aspects - especially fitting

1.

"So there's the degree of invisibility amongst migrant populations. Any category that a service like these could address and does address when, when the time comes, when they, they feel brave enough to come through the GP or they feel safe, they have some level of status, or they're either waiting for asylum, claim to come through, or they're waiting on a case and they feel a little bit safer, legally speaking. And then they go to the health care setting and then, then there's a degree of visibility and feeling heard and seen and knowing what's available, because most of them don't know that there are services available for them, there are advocacy services available for them. There are food banks that they can access. There's a lot. But if you don't come across those, those services that can point you in the right direction and signpost you or refer you then."

Code: SP aspects - especially fitting
P7, Social prescriber

2.

"So obviously the whole family approach is really useful because when you do a needs assessment, you ask certain questions, um, that you're trying to do as a social prescriber to draw out any needs around the whole family. And they might not know that they've got access to things. So you'll do things like a benefit check. You know, um, uh, to see if they, they're entitled to benefits or refer them for an assessment if their child's got SEM or refer them to a support agency to help them into work. So there's all these connections that social prescribers do in a traditional way that really work well and put people in touch with staff, signposting, referrals, all that kind of thing. Um, and without that, they wouldn't have access to a lot of things that the families do have. Um, um, so in that respect, it works really well. Um, that kind of, um, light touch, supportive role works really well."

Code: SP aspects - especially fitting
P2, Youth service manager

3.

"Um. And then, yeah, being able to signpost to services that, of course, a person is not going to know about whether, because, um, they don't have access to a phone or a computer to be able to do that research. Um, whether they've just, they've just landed here so that they don't have any contacts, how on earth would they know about like, you know, this random refugee, I mean, like (charity name), for example. No one knows about (charity name), this very, you have to get referred in and it's like it'd be impossible to find that by yourself. Um very niche kind of group like that, you know. Um. And I had something else in mind as well. Um. (...) Like I said, actually, a lot of the services do do also require referrals. So you do need those those link people"

Code: SP aspects - especially fitting
P1, Social Prescriber

4.

"Um, it's again, it's the beauty of social prescribing is that we don't necessarily just cut off that support. We will always say to someone, um, we're being quite open and honest, we don't feel like you're engaging or, you know, they might just not go back and contact. But whenever you are ready, you can get back in contact. We always let a client know that we do require them to be proactive. Um, so I tend to say, you know, if it's just opening links or letters that I send over to you, that's a good enough start and we can build up from there just so they don't feel the pressure and get automatically put off by what the social prescribing kind of wants them to do."

Code: SP aspects - especially fitting

P17, Social prescriber

5.

"So just going back to the again, the young um, lad that I supported, he um, he messaged me because he needed something else and he was like, can you please ask the doctor this or can you give me that support? Unfortunately, that time frame, he'd actually moved surgeries. Um, so I was trying to explain so, "You are no longer under my support. Um, Unfortunately. But there is a social prescriber that covers your surgery, so you will have that support there." Um, but otherwise he would just suffered in silence and pain of whatever was happening to him. So because there are so many of us based over the GPS, it is all interlinked. So if someone was to move for any reason, the asylum seeker still has that sort of continued support."

Code: SP aspects - especially fitting
P17, Social prescriber

6.

"Advocacy? Uh, definitely. I've seen, I've seen letters that social prescribers have written to advocate for, um, uh, so and I'm talking more about asylum seekers when they, when they have um, at the been struggling with, um, housing, um, like, um, actually and refugees as well when they've struggled with, with social housing, writing letters of support. Um, but also for things like reporting requirements for the Home Office. Yeah, we've seen that as well. So I think they can be really strong advocates and they have a bit more time than, than health professionals to really understand that person."

Code: SP aspects - especially fitting
P14, GP and lead for local health stream initiative

7.

"So and I think, I think supporting people to exist in a really shitty situation, to exist in poverty. You know, to know that they can get free bus tickets or that they can get free prescriptions or that. Um, you know, I, you know, I can't change what the Home Office does to you. I can't change your accommodation necessarily. I can advocate for you. But I do think, you know, social prescribers could really do some small things that really support that person to exist in a really tough situation."

Code: SP aspects - especially fitting
P14, GP and lead for local health stream initiative

71. SP evaluation - SP as useful

1.

"And I think, um, I think social prescribing has great worth if, um, if the other supports are there to facilitate. So I said language. I said, um, parent support, parenting, I said, and the stress and the trauma of um, of interviews. But I think also there is uh, I forgot what I was going to say. No, no, you you, you know, there's, you know, there's, there's a lot of difficulties. Really."

Code: SP evaluation - SP as useful
P22, Social prescriber

2.

"What I would say is that I haven't ever seen a case where a GP has suggested an activity to a child or anything to a child, apart from medical treatments. And I think that if you got in a room with social care and with health, health would argue that it wasn't their responsibility and social care might agree. But health I don't say would pick it up. No, not at all."

Code: SP evaluation - SP as useful
P16, Quality Assurance Teacher for post-16 education

3.

"Useful? Um, I think it depends a little bit, if people don't speak the language. Okay, because it's great sending them there and then they may get, they used Google Translate or whatever. And it's not necessarily as helpful as some of the people want. You know, sometimes they want to be put in touch with other people from the same ethnic backgrounds. And, you know, because they feel very isolated. And understand from their perspective, you know, where it's safe to go. And, you know, they've, they've got a, the police sign on their website. Does that mean that they tell the police? And you know, it's those kind of thing for our, for our service users. Uh, it's a big, big issue. You know, there is a mistrust and, and so in those cases, it's not always as helpful."

Code: SP evaluation - SP as useful
P18, CEO

4.

"Oh, I know, yeah. To have someone to, I think I said that already, like, have someone to just acknowledge, like, how difficult it is, um, and not just fully be in the like. Okay, well, social prescribing is a weird role because you are there to be providing practical support, but you're in this like little zone where you can, yeah, acknowledge some of the, the challenges and the the trauma that the person is coming with and… whilst also offering practical support. (Person 1: Yeah) I think that's really needed."

Code: SP evaluation - SP as useful
P1, Social Prescriber

5.

"Yeah, I imagine it's better in, you know, places like (large city) or somewhere like that. Yeah, it might be quite a different, um, you know, the social prescribers might be upskilled in, um, in these areas, um, and, and things like the, you know, one of the commonest stresses is, is legal support. (Person 1: Yes.) Uh, and again, uh, you know, yeah, uh, I know that that one of our local charities is now offering legal drop ins, but I'm probably the only GP that knows that and I'm trying to get that shared. And probably I don't think any of the social prescribers would know that. So if someone comes to them and goes "I've got to appeal my asylum claim. I'm really stressed out by this. I don't know where to go." they're not likely to say, go to this drop in at (charity name) on a Thursday."

Code: SP evaluation - SP as useful
P14, GP and lead for local health stream initiative

6.

"So and I think, I think supporting people to exist in a really shitty situation, to exist in poverty. You know, to know that they can get free bus tickets or that they can get free prescriptions or that. Um, you know, I, you know, I can't change what the Home Office does to you. I can't change your accommodation necessarily. I can advocate for you. But I do think, you know, social prescribers could really do some small things that really support that person to exist in a really tough situation."

Code: SP evaluation - SP as useful
P14, GP and lead for local health stream initiative

72. SP evaluation - other approaches as useful

1.

"Person 1: Yeah. Good, good, strong answer. Um, and do you see it as a useful approach when working with refugee populations and or do you think there are other approaches that might be more fitting or more sustainable?

Person 2: I think it is. When it's done well, I think it could be absolutely transformative. Um, but, uh, I think it's not done. It's not, that it's not done well, it's just not done for, for this group. That I have seen. But I would be really interested to know where it's done better. But I think, I think once people get to the right voluntary sector organisations that can be really support people's wellbeing."

Code: SP evaluation - other approaches as useful
P14, GP and lead for local health stream initiative

2.

Person 2: "I'd love to know that, I was genuinely quite interested. I mean, I was cautious because of all the reasons. You know, I am cautious. And at (university), um, where I'm studying, uh, my supervisor is health and wellbeing, and we do a lot with the doctors, training the doctors with the arts, low level that, you know. Um, but it's also and that's where again, I'm coming to learn more about, um, how all the arts might be brought in to cure people with all their ailments and their mental health. And I am, I am cautious about that because arts practice is arts practice. It's not drama therapy. You know I did one year's training as a drama therapist the first year and then I knew I didn't have the…

Person 1: Okay. It wasn't for you.

Person 2: Um yeah I… Yeah. No because it, it, it was too um upsetting. It was, I didn't, I couldn't keep that distance. You know, it was the arts, you know, arts and wellbeing space was. I felt comfortable with that, you know, but I couldn't go deeper and dig deeper into to be a drama therapist. You know, it was it was it was a lot for me and the empathy side of it, I couldn't. You know, it was a long, um. And so I think it's you have to be really cautious about actually the arts who are already underfunded and, and, um, literally on its knees as a sector actually shouldn't be brought in where in fact there should be investment in the NHS. So that's what I feel is like actually the NHS should be employing mental health professionals, um, to be tackling some of the issues and the arts are very happy to, we're very happy to join in and support that, but we can't, it would be dangerous for us to tackle some of some of the issues that, if I've understood social prescribing correctly."

Code: SP evaluation - other approaches as useful
P9, Drama programme founder and producer/director
